# Single-Cell Analysis Uncovers Osteoblast Factor Growth Differentiation Factor 10 as Mediator of Vascular Smooth Muscle Cell Phenotypic Modulation Associated with Plaque Rupture in Human Carotid Artery Disease

**DOI:** 10.3390/ijms23031796

**Published:** 2022-02-04

**Authors:** Karim J. Brandt, Fabienne Burger, Daniela Baptista, Aline Roth, Rafaela Fernandes da Silva, Fabrizio Montecucco, Francois Mach, Kapka Miteva

**Affiliations:** 1 Division of Cardiology, Foundation for Medical Research, Department of Medicine Specialized Medicine, Faculty of Medicine, University of Geneva, Av. de la Roseraie 64, CH-1211 Geneva 4, Switzerland; karim.brandt@hcuge.ch (K.J.B.); fabienne.burger@unige.ch (F.B.); daniela.baptista@unige.ch (D.B.); aline.roth@unige.ch (A.R.); rfdasilva.ufmg@gmail.com (R.F.d.S.); Francois.mach@hcuge.ch (F.M.); 2 Department of Physiology and Biophysics, Institute of Biological Sciences, Federal University of Minas Gerais, Belo Horizonte 6627, Brazil; 3 Swiss Institute for Translational and Entrepreneurial Medicine, Freiburgstrasse 3, 3010 Bern, Switzerland; 4 Ospedale Policlinico San Martino Genoa—Italian Cardiovascular Network, 10 Largo Benzi, 16132 Genoa, Italy; fabrizio.montecucco@unige.it; 5 First Clinic of Internal Medicine, Department of Internal Medicine, Centre of Excellence for Biomedical Research (CEBR), University of Genoa, 6 Viale Benedetto XV, 16132 Genoa, Italy

**Keywords:** vascular smooth muscle cells, atherosclerosis, carotid artery disease

## Abstract

(1) Background: Vascular smooth muscle cells (VSMCs) undergo a complex phenotypic switch in response to atherosclerosis environmental triggers, contributing to atherosclerosis disease progression. However, the complex heterogeneity of VSMCs and how VSMC dedifferentiation affects human carotid artery disease (CAD) risk has not been clearly established. (2) Method: A single-cell RNA sequencing analysis of CD45^−^ cells derived from the atherosclerotic aorta of Apolipoprotein E-deficient (Apoe^−/−^) mice on a normal cholesterol diet (NCD) or a high cholesterol diet (HCD), respecting the site-specific predisposition to atherosclerosis was performed. Growth Differentiation Factor 10 (GDF10) role in VSMCs phenotypic switch was investigated via flow cytometry, immunofluorescence in human atherosclerotic plaques. (3) Results: scRNAseq analysis revealed the transcriptomic profile of seven clusters, five of which showed disease-relevant gene signature of VSMC macrophagic calcific phenotype, VSMC mesenchymal chondrogenic phenotype, VSMC inflammatory and fibro-phenotype and VSMC inflammatory phenotype. Osteoblast factor GDF10 involved in ossification and osteoblast differentiation emerged as a hallmark of VSMCs undergoing phenotypic switch. Under hypercholesteremia, GDF10 triggered VSMC osteogenic switch in vitro. The abundance of GDF10 expressing osteogenic-like VSMCs cells was linked to the occurrence of carotid artery disease (CAD) events. (4) Conclusions: Taken together, these results provide evidence about GDF10-mediated VSMC osteogenic switch, with a likely detrimental role in atherosclerotic plaque stability.

## 1. Introduction

Cardiovascular diseases are the leading cause of death in developed countries [1,2], with atherosclerosis being the predominant underlying cause. The disruption of the atheroprotective layer of vascular smooth muscle cells (VSMCs), which forms the fibrous cap that covers the atherosclerotic plaque [3], induces acute thrombo-occlusive events, such as myocardial infarction and stroke [4] Evidence has demonstrated that the different embryonic ascending and descending aortic domains exhibit distinct phenotypes [5] which impact disease progression [6]. Site-specific development of atherosclerotic lesions is observed in both murine models of atherosclerosis and in humans [7]. The regions susceptible to the development of atherosclerosis are called “atherosclerosis-prone”, whereas areas less prone to atherosclerosis are referred to as “atherosclerosis-resistant” [7]. Apoe^−/−^ mice exhibit lesion formation in the aortic arch and root (AA&R) and the abdominal aorta [7]. The observed spatial dissemination of vascular diseases could also be explained by differences in hemodynamic and vessel structure [8,9]. Respecting the site-specific predisposition of atherosclerosis, we undertook a precise approach to reveal disease-associated cell populations, genes and molecular determinants by performing single-cell RNA-sequencing (scRNAseq) of atherosclerosis-prone sites separately from atherosclerosis-resistant sites. 

VSMCs are not terminally differentiated and undergo complex phenotypic changes during atherosclerosis in response to environmental triggers, including growth factors, extracellular lipids and lipoproteins, and various inflammatory mediators in the injured artery wall [3]. VSMC phenotypic switch results in the transition toward a synthetic phenotype of a dedifferentiated state, characterized by the decrease or loss of VSMC-specific cytoskeletal proteins [3] and the acquisition of markers of macrophages, mesenchymal stem cells and myofibroblasts [3], as well as osteoblasts and chondrocytes [10,11]. A study combining single-cell transcriptomics identified VSMC-lineage cells expressing the multipotent progenitor marker Sca1 in atherosclerotic plaques which downregulate contractile VSMC genes expression [12]. Tcf21 was shown to promote VSMC transition to “fibromyocytes”, causing increased fibrosis, fibrous cap stabilization and subsequent lower risk of acute cardiovascular events [13]. Klf4 has been extensively studied as a key regulator of VSMC phenotypic modulation and atherosclerotic plaque pathogenesis [11,14,15]. A recent study demonstrated that Klf4 regulates VSMC transition to Lgals3^+^ osteogenic cells with a likely detrimental role in atherosclerosis [14]. However, the exact link between KLF4 and osteogenic-like cells is not fully revealed and the osteogenic cells such as differentiation of VSMCs may require the involvement of specific osteogenic mediators.

The diversity, heterogeneity and complex phenotypical modulation of VSMCs, as well as the factors and the molecular determinants controlling this process at the disease-relevant vascular regions during early and advanced atherosclerosis, are incompletely revealed. By investigating the complex interplay of factors operating at atheroprone versus atheroresistant sites, a more complete understanding of the overriding mechanisms governing the initiation and progression of atherosclerosis may be achieved.

The present study aimed to comprehensively characterize the transcriptomic profile of phenotypically-modulated VSMCs and identified mediators of VSMC transdifferentiation and their link to plaque rupture in human atherosclerosis.

## 2. Results

### 2.1. Hypercholesteremia-Associated Transcriptional Signature

**Figure 1:**
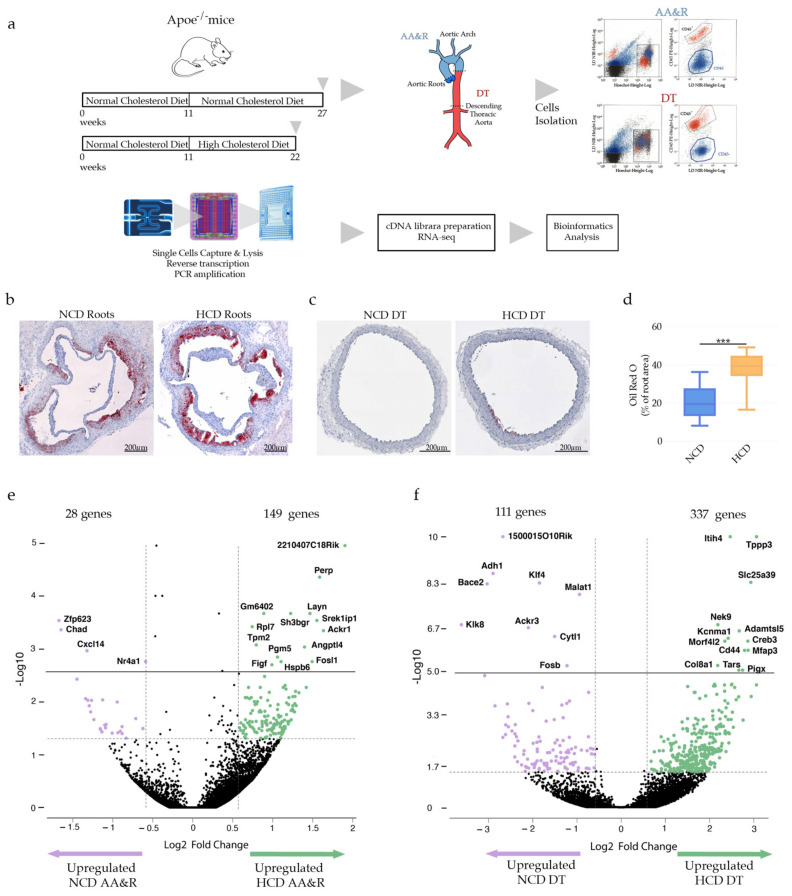
(**a**) Experimental setting of scRNAseq of CD45^−^ cells. (**b**) Oil Red O stained atherosclerotic lesions of Apoe^−/−^. (**c**) Oil Red O stained DT aorta of Apoe^−/−^. (**d**) Bar graphs represent the mean ± SEM of atherosclerotic lesion quantification with *n* = 6–8/group and ***p < 0.001. (**e**) Volcano plot showing relative gene expression of CD45^−^ cells derived from AA&R of Apoe^−/−^ on NCD versus HCD. Each dot represents a gene within the performed comparison (p-Adj < 0.05, Log2 > ±0.58), *n* = 6 mice/group. (**f**) Volcano plot showing significance versus relative gene expression of CD45^−^ negative cells derived from DT aorta of Apoe^−/−^ on NCD versus HCD. Each dot represents a gene within the performed comparison (p-Adj < 0.05, Log2 > ±0.58), *n* = 6 mice/group.

We investigated the gene expression profile of cells separately derived from the atheroprone AA&R and the more atheroresistant DT aorta. The adventitia was carefully excised by sharp surgical dissection in a clearly defined plane to leave a naked media composed of VSMC and endothelial cells (CD45^−^ cells). Fluidigm C1 platform for RNA sequencing of single cells was used to reveal the transcriptional profiles of viable individual CD45^−^ aorta cells isolated from the AA&R and DT aorta of Apoe^−/−^ mice fed either an NCD for 16 weeks or HCD for 11 weeks (Figure 1a). Cells with more than 1.5% of reads aligning to mitochondrial-encoded genes were discarded. As a result, the scRNAseq profiles of 680 cells passed the quality control with respective reads mapped to genes, respective number of genes per cell and unique molecular identifier (UMI) per cell, as indicated in Supplementary Figure S1a,b. HCD-fed Apoe^−/−^ mice showed significantly elevated levels of cholesterol and LDL-C (Supplementary Figure S1c,d) and larger plaques in the aortic roots (Figure 1b,d). Apoe^−/−^ mice fed HCD did not exhibit plaques formation in the DT aorta, as evident by Oil Red O staining (Figure 1c). To better explain the broader mechanisms that control the initiation and progression of atherosclerosis, we investigated how hypercholesteremia impacts the gene expression profile specifically in atheroprone AA&R. The volcano plot and the heat map illustrate the gene expression profile under HCD compared with NCD specifically of AA&R-derived cells of Apoe^−/−^ mice (Figure 1e, Supplementary Figure S2a). In total, 177 genes were differentially expressed (p-Adj < 0.05, Log2 > ±2), of which 149 genes were upregulated under HCD and 28 were downregulated (Figure 1e, Supplementary Figure S2a and Supplementary Table S1). As expected, among the significantly upregulated genes in AA&R of Apoe^−/−^ mice on HCD were genes involved in apoptosis, cell adhesion, cell survival, VSMC growth, migration, atherogenesis, foam cells formation and inflammation (*Perp* [16], *Pgm5*, *Layn* [17], *Srek1ip1*, *Rpl7* [18], *Ackr1*, *Ackr1* [19] *Tpm2*, Angptl4 *Fosl1* [20], *Angptl4* [21], *Figf* [22]) (Figure 1e). In contrast, genes with a probably important atheroprotective function, such as regulator of vascular inflammation (*Zfp623*) [23] and inhibitor of VSMC proliferation (*Nr4a1*) [24], were downregulated. In total, 448 genes were differentially expressed in the DT-derived cells of Apoe^−/−^ mice on HCD compared with NCD (p-Adj < 0.05, Log2 > ±2), of which 337 genes were upregulated while 111 genes were downregulated (Figure 1f, Supplementary Figure S2b and Supplementary Table S2). Even though we did not observe plaque formation in the DT aorta of Apoe^−/−^ mice fed an HCD (Figure 1f), the gene expression profile of these cells was consistent with a profound response to atherogenic stress. Significantly upregulated were gene regulators of VSMC differentiation, triggers of cell apoptosis, inflammation and coronary heart disease markers (*Itih4* [25], *Tppp3* [26], *Slc25a39* [27], *CD44* [28]) in parallel to atheroprotective genes such as *Morf4l2* [29]. In contrast, genes inducing vascular damage, such as *Adh1* [30] and Klf4, a key regulator of VSMC phenotypic modulation and atherosclerotic plaque pathogenesis [11,14], were downregulated in the DT-derived cells of Apoe^−/−^ mice fed an HCD (Figure 1f). The hypercholesteremia clearly triggers atherosclerosis-associated vascular damage gene expression profile, not only in the more atheroprone AA&R but also in the atheroresistant DT aorta-derived cells, in line with the findings, showing that atherosclerosis of the descending aorta is a useful predictor of cardiovascular events [31].

### 2.2. Atherosclerotic Disease Site-Specific Transcriptional Signature

Even under an NCD, a major difference in the gene expression profile of atheroprone AA&R cells compared with more disease-resistant DT aorta-derived cells of Apoe^−/−^ mice was seen (Supplementary Figure S3a), with a total of 1908 differentially expressed genes (p-Adj < 0.05, Log2 > ±0.58), of which 1364 were upregulated in the AA&R and 544 in the DT aorta-derived cells (Supplementary Figure S3a). Interestingly, the gene expression profile recapitulates different aspects of VSMC phenotypic switch, such as VSMC proliferation, migration, lipid accumulation, calcification, adipogenesis and osteogenic development (*Dpep1* [32], *Scara5* [33,34], *Smoc2* [35], *Clec11a*, *Dpt* [36,37]) (Supplementary Figure S3b). In contrast, the genes upregulated in the DT aorta of Apoe^−/−^ mice on an NCD exhibited an atheroprotective gene expression profile linked to cardiac and skeletal muscle development, vasodilation, cholesterol efflux and VSMC phenotype preservation (*Ptprz1* [38,39], *Ramp1* [40], *Hdac9*n [41], *Sh3bgr* [42,43]) (Supplementary Figure S3b). The vascular site-specific transcription signature reveals molecular determinants of atherosclerotic disease susceptibility or protection even before the onset of hypercholesteremia-induced atherogenic stress. AA&R compared with DT aorta-derived cells of Apoe^−/−^ mice on an HCD showed differential expression of 1558 genes in total (p-Adj < 0.05, Log_2_ > ±0.59), of which 1071 were upregulated in the AA&R and 488 downregulated in the DT aorta (Supplementary Figure S3c). In response to hypercholesteremia, the AA&R showed upregulation of genes regulators of cholesterol efflux (*Abca8a*) [44] and blood coagulation (*Entpd2*) [45] (Supplementary Figure S3d), while DT-derived cells expressed genes with a potentially important role in cell fate preservation (*Des* [46], *Mira* [47]) (Supplementary Figure S3d). A notable observation is that the genes upregulated in the DT aorta may be of fundamental importance in the protection against atherosclerotic disease pathology and cell fate preservation. Interestingly, genes associated with cardiovascular disease mortality (*Clec3b* [40]), as well as VSMC transdifferentiation such as *Igfbp6* [48] and *Dpep1* [49], were upregulated in AA&R versus DT aorta-derived cells of Apoe^−/−^ mice, independently of the diet (Supplementary Figure S3b,d), implying that these genes may be key determinants in atherosclerotic site-specific predisposition.

### 2.3. Signaling Pathway Highlighted during Atherosclerosis Progression

**Figure 2:**
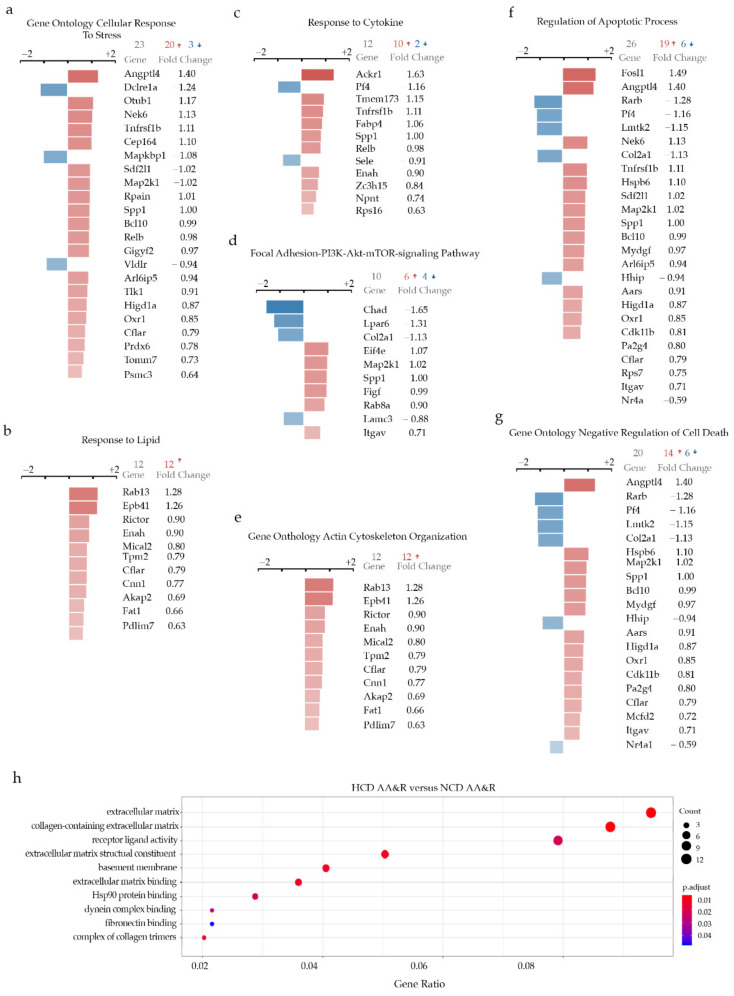
HCD-induced signaling in AA&R (**a**) Gene Ontology Cellular Response To Stress; (**b**) Response to Lipid; (**c**) Response to Cytokine; (**d**) Focal Adhesion-PI3K-Akt-mTOR-signaling Pathway; (**e**) Gene Onthology Actin Cytoskeleton Organization; (**f**) Regulation of Apoptotic Process; (**g**) Gene Onthology Negative Regulation of Cell Death; (**h**) Bubble plot of selected GO terms enriched in AA&R of Apoe^−/−^ on NCD versus HCD. Dot size is proportional to the number of genes overlapping 222with each GO term, and the adjusted *p*-value is color-coded from red to blue.

In response to hypercholesteremia, the cells derived from the more atheroprone AA&R exhibited induction of gene sets and pathways typically showing atherosclerosis progressions, such as cellular response to stress, lipids, cytokines, apoptosis and cell death (Figure 2a–c,f,g). Interestingly, the focal adhesion-PI3K-Akt-mTOR-signaling pathway (Figure 2d,e) and actin cytoskeleton organization gene set resulted in the reorganization of the actin cytoskeleton facilitating changes in cell shape, motility was all induced upon HCD feeding in AA&R-derived cells. Importantly, it was shown that selective inhibition of the Akt/mTOR signaling pathway can promote autophagy and the stabilization of vulnerable atherosclerotic plaques [50] Therefore, actin cytoskeleton organization may appear as an important target whose abrogation could be beneficial in atherosclerosis. In parallel, the observed changes in the extra cellular matrix (ECM) structure, collagen-containing ECM and ECM structural constituent (Figure 2h) gene sets and pathway activation could be explained by a hypercholesteremia-induced VSMC switch from contractile to synthetic state as a major process in atherosclerosis progression.

**Figure 3:**
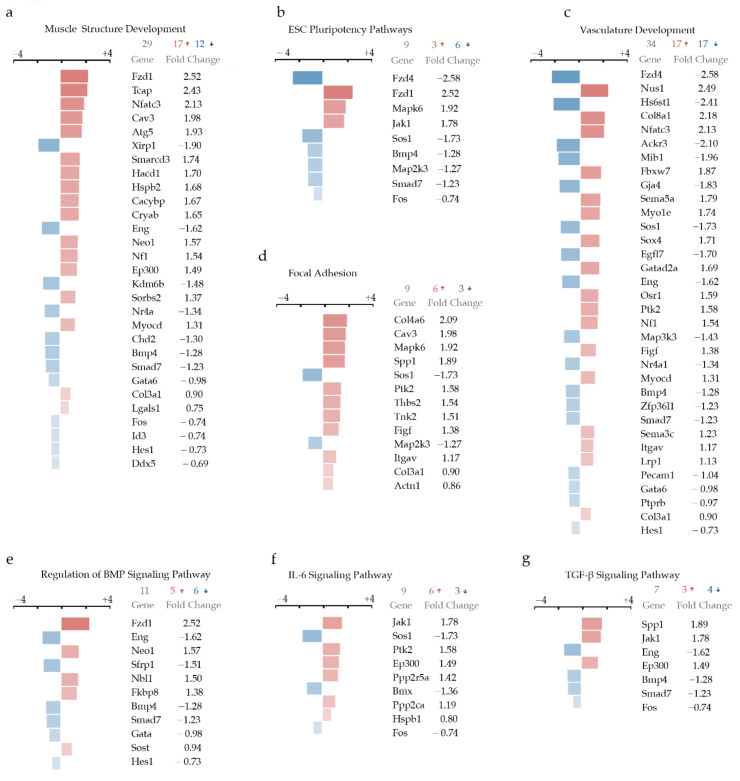

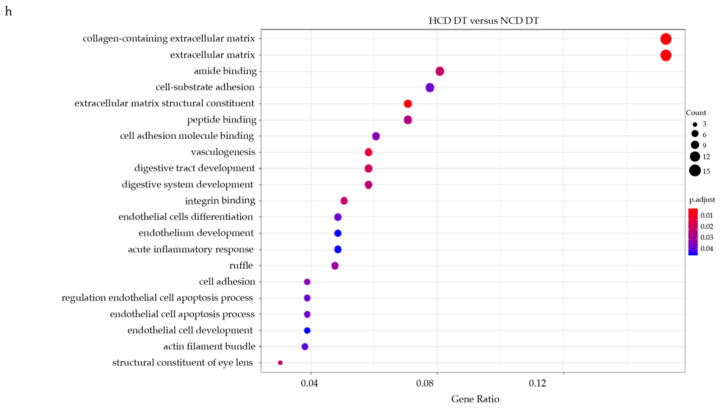
(**a**) Muscle Structure Development; (**b**) ESC Pluripotency Pathways; (**c**) Vasculature Development; (**d**) Focal Adhesion; (**e**) Regulation of BMP Signaling Pathway; (**f**) IL-6 Signaling Pathway; (**g**) TGF-β Signaling Pathway; (**h**) Bubble plot of selected GO terms enriched in DT aorta of Apoe^−/−^ on NCD versus HCD. Dot size is proportional to the number of genes overlapping with each GO term, and the adjusted *p*-value is color-coded from red to blue.

In contrast, hypercholesteremia in the DT aorta-derived cells promoted the regulation of gene sets linked to muscle structure and vasculature development (Figure 3a,c), preserving the VSMC phenotype. In addition, embryonic stem cell pluripotency and focal adhesion were also regulated in cells derived from the more atheroresistant DT aorta under an HCD (Figure 3b,d). Interestingly, the pro-atherogenic stress promoted regulation in BMP (bone morphogenetic proteins) signaling (Figure 3e), as seen by a 2.5-fold increase in the expression of the Fz1 (Frizzled 1) gene shown to repress the induction of alkaline phosphatase mediated by bone morphogenetic protein [51], which seems to be a mechanism protecting against phenotypic changes in DT aorta-derived cells resulting in vascular calcification. In addition to IL-6 (Figure 3f) were TGF-β signaling (Figure 3g) genes of ECM structure, collagen content, ECM, ECM structural constituent, adhesion and endothelial cell development, differentiation and apoptosis (Figure 3h). The observed signaling pathways clearly indicate microvascular damage in the DT aorta, which is in line with previous findings, showing that patients with atherosclerosis of the thoracic aorta have a higher probability of coexisting coronary artery disease [52,53]. Moreover, plaque composition and vulnerability rather than the degree of stenosis defined by the lesion size have emerged as crucial factors leading to plaque rupture, which underlies the great majority of infarctions [54].

### 2.4. scRNAseq Analysis Reveals Seven Distinct Cell Clusters

**Figure 4:**
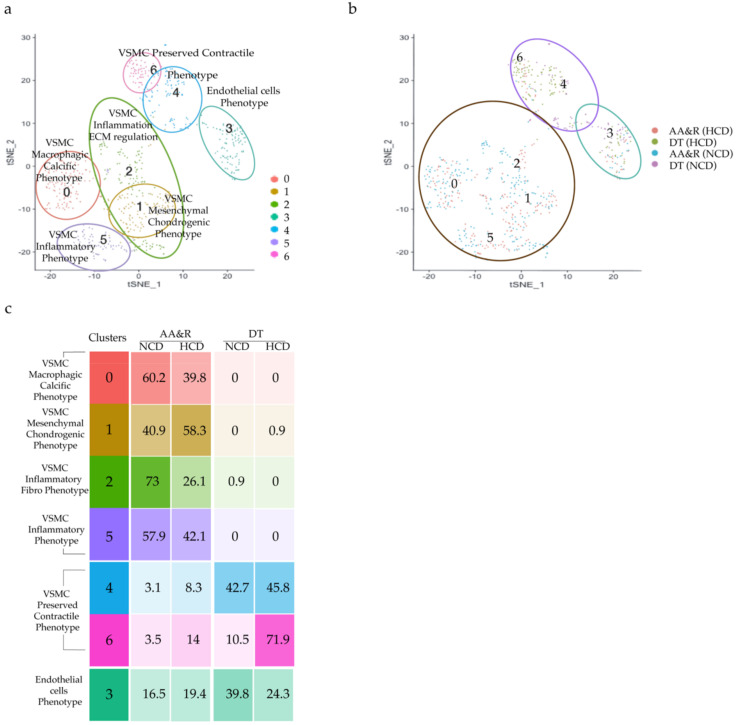
Clusters of AA&R and DT aorta cells t-distributed stochastic neighbor embedding (tSNE) plot showing: (**a**) all seven identified clusters; (**b**) AA&R and DT aorta clusters of Apoe^−/−^ mice on NCD or HCD; (**c**) Relative frequency of cells derived from AA&R and DT aorta of Apoe^−/−^ mice on NCD and HCD composing the 7 clusters.

An unsupervised clustering algorithm was applied to investigate the aortic cell phenotypic diversity and revealed distinct cell clusters typical for either atheroprone AA&R or more atheroresistant DT aorta of Apoe^−/−^ mice. Seven clusters of CD45^−^ cells were identified, as shown in the tSNE plots (Figure 4a,b and Supplementary Figure S2c–l). Clusters 0, 1, 2 and 5 were composed of cells derived almost exclusively from AA&R cells of Apoe^−/−^ mice on an NCD or HCD and expressed genes of macrophage foam cells (Supplementary Figure S2g), VSMC phenotypic transition (Supplementary Figure S2h), vascular inflammation and calcification (Supplementary Figure S2i,j) in parallel with a pronounced downregulation of VSMC contractile genes (*Myh11* and *Acta2*) (Supplementary Table S3). Most cells of clusters 4 and 6 were derived from the DT aorta (Figure 4c) and exhibited gene expression profile of VSMC with the preserved contractile phenotype (Supplementary Figure S2k), with upregulation of VSMC contractile markers such as *Tpm2* (tropomyosin), *Tagln* (Transgelin), *Cnn1*, *Myh11* and *Acta2* (Supplementary Table S3). Cluster 3 contained cells derived from AA&R or DT aorta of Apoe^−/−^ mice on an NCD as well as HCD (Supplementary Figure S2l) and expressed a set of genes corresponding to endothelial cells as well as vascular inflammation (Supplementary Figure S2l and Supplementary Table S3).

### 2.5. Gene Signature of Atherosclerotic Phenotypically Modulated VSMC Clusters

**Figure 5:**
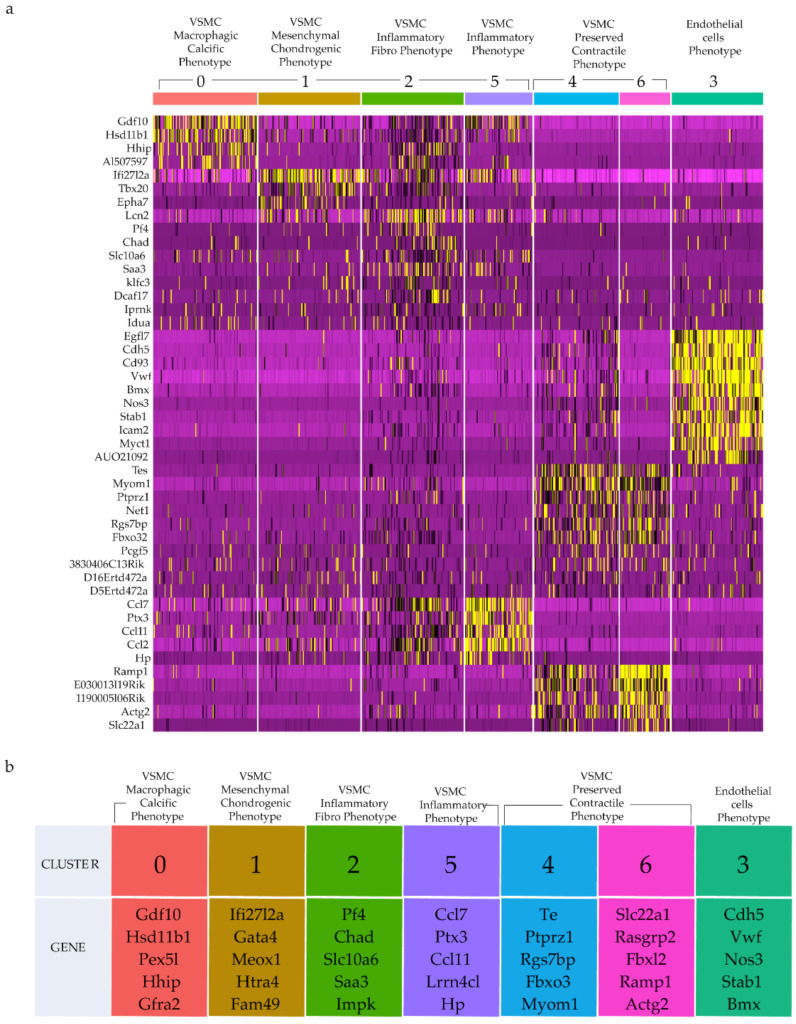
Clusters Gene Expression Signature. (**a**) Heatmap illustrating the top differentially expressed genes among all detected clusters. (**b**) Top 5 differentially expressed genes detected in each cluster.

The gene expression profile of cluster 0 reveals the gene signature of VSMCs undergoing transdifferentiation to a unique population of mixed macrophagic calcific osteogenic-like cells (Figure 5a,b). The top differentially expressed genes of cluster 0 include osteoblast factor *GDF10* (BMP-3b), shown to regulate cell differentiation, skeletal morphogenesis [55], ossification, osteoblast differentiation [56,57,58], and linked to increased Alkaline Phosphatase activity [56]. In addition, cluster 0 expressed *Serpinf1*, involved in ossification and osteoblast differentiation [59], Hsd11b1, shown to exacerbate atherosclerosis [60], Pex5l, expressed in macrophages [61] and Gfra2 in monocytes [62], as well as cystatin C, associated with coronary artery calcification [63] (Figure 5a,b and Supplementary Figure S4a). Differentially expressed genes of cluster 0 are linked to cholesterol efflux (*Abca8*) [44] and induction of chondrogenesis (Itgbl1) [64] (Supplementary Figure S4a). Moreover, cluster 0 also expresses Svep1, which was recently shown to promote atherosclerosis in humans and mice, which is expressed by VSMCs within the atherosclerotic plaque cause proliferation and dysregulation of key differentiation pathways, including integrin and Notch signaling [65]. Furthermore, the GO terms analysis of cluster 0 showed induction of ossification, canonical and noncanonical Wnt-signaling (Supplementary Figure S5a). Both canonical and noncanonical Wnt ligands contribute to pathological calcification of the aortic vasculature [66]. This particular gene expression profile links this cluster to macrophagic calcific osteogenic-like cells phenotype. Cluster 1 represents a distinct population of cells expressing genes involved in inflammation (*Ifi27l2a*) [67], hyperlipidemia (*Gata4*) [68], VSMC phenotypic modulation (*Meox1*) [69], calcification and bone formation (*Htra4*, *Meox1*) [70,71] (Figure 5a,b and Supplementary Figure S4b ). The GO term enrichment analysis of cluster 1 shows activation of mesenchymal cell differentiation, inflammation, ECM organization and chondrocyte differentiation, as well as *osteogenic* as ERK1/2, a critical inducer of cell proliferation [72] (Supplementary Figure S5b). The observed gene expression profile of cluster 1 clearly shows a population of phenotypically modulated VSMC acquiring mesenchymal chondrogenic phenotypes potentially involved in vascular inflammation and calcification. The gene expression profile of cluster 2 revealed another VSMC-modified phenotype associated with vascular inflammation and ECM regulation, as evidenced by expression of genes encoding pro-atherogenic chemokine (Pf4) and a key determinant of TLR-induced innate immunity (*Ipmk*) [73] (Figure 5a,b and Supplementary Figure S4c). The GO term enrichment of cluster 2 shows ECM proteins and ECM structural constituents characterizing VSMC with a synthetic phenotype in addition to cytokines and chemokines activity (Supplementary Figure S5c). Interestingly, cluster 5 exhibited an inflammatory phenotype gene expression profile characterized by the expression of *CCL7* (*MCP-3*), promoting monocyte mobilization and VSMC proliferation [74]; *Ccr11*, induced in VSMC upon arterial injury; and *Gpr133 with* immunoregulatory properties [75] (Figure 5a,b and Supplementary Figure S4e). In parallel, the GO term enrichment analysis of cluster 5 confirmed the phenotype of this population as a mediator of inflammation, as evidenced by the induction of leukocytes, granulocytes and neutrophil migration, defense response, cytokine production, inflammatory response genes and cellular response to IL-1 (Supplementary Figure S5f). AA&R-derived cells show impressive phenotypic plasticity and diversity of phenotypically modified VSMC, highlighting the complex atherosclerotic mechanisms orchestrating VSMC transdifferentiation into multiple cell states/types attuning to atherosclerosis progression.

### 2.6. Gene Signature of Clusters with Preserved Phenotype

Clusters 4 and 6, composed predominantly of cells from the thoracic part of the aorta (Figure 4c), exhibited a gene expression profile of VSMCs with preserved contractile phenotype, as evidenced by the expression of genes essential in preserving the VSMC phenotype and in control of VSMC contractile function, such as *Rgs7bp*, *Fbxo32* [76], *Myom1* [77,78], *Fbxl22*, *Actg2*, *Tpm2* (Tropomyosin), *Tagln* (Transgelin), *Cnn1*, *Myh11*, *Myl6b*, *Asb2*, *Tmsb4x* [79]; *Tpm2*, *Acta1* and *Acta2* (Figure 5a,b and Supplementary Figure S4e,g). Among the differentially expressed genes were atheroprotective genes such as *Rasgrp2*, an inhibitor of thrombus formation [80], *Ramp1*, associated with vascular resistance [81], (Figure 5a,b) and *Itga8*, an inhibitor of VSMC proliferation and migration [82] (Figure 5a,b and Supplementary Figure S4e). In line with the gene expression profile of VSMCs with preserved phenotypes, the GO term enrichment analysis of clusters 4 and 6 revealed enrichment of muscle processes, heart and smooth muscle contraction, cardiac muscle development and differentiation, actin filament organization genes, muscle cell differentiation, regulation of muscle contraction and genes of heart and muscle contraction (Supplementary Figure S4e,g). The clusters of atheroresistant DT aorta cells delineated a transcriptional profile of VSMC-preserved contractile phenotype with the induction of genes linked to vascular pathology protection, cholesterol efflux, as well as positive regulation of the genes suppressing inflammation, apoptosis, and thrombosis. Cluster 3 showed a gene expression profile typical of endothelial cells, including *Cdh5* (VE-cadherin), *Vwf* and *Nos3* (eNOS) [83]; *Stab1*, involved in angiogenesis, lymphocyte homing and cell adhesion; *Bmx*, promoting ischemia-induced inflammatory angiogenesis [84] (Figure 5a,b and Supplementary Figure S4d); genes of endothelial activation, function and proliferation, and neo-angiogenesis such as *Pecam1*, *Egfl7*, *Ptprb* and *Mmrn2* [85]; and genes such as *Cdh5* promoting vascular permeability and leukocyte transmigration [86] and *Tie1*, involved in atherogenic shear stress and inflammation in atherosclerosis [87] (Figure 5a,b and Supplementary Figure S4d) The GO term enrichment analysis of cluster 3 is in line with the endothelial cell identity of this cluster and shows enrichment of endothelial cell differentiation, endothelium development and endothelial cell migration genes, the establishment of an endothelial barrier, cell migration and junction, which appears to facilitate cell–cell contact, and functions as a paracellular barrier that dysfunctions in response to inflammation and pathology-associated gene activation of endothelial cells (Supplementary Figure S5d).

### 2.7. GDF10 Promotes VSMC Phenotypic Modulation

**Figure 6:**
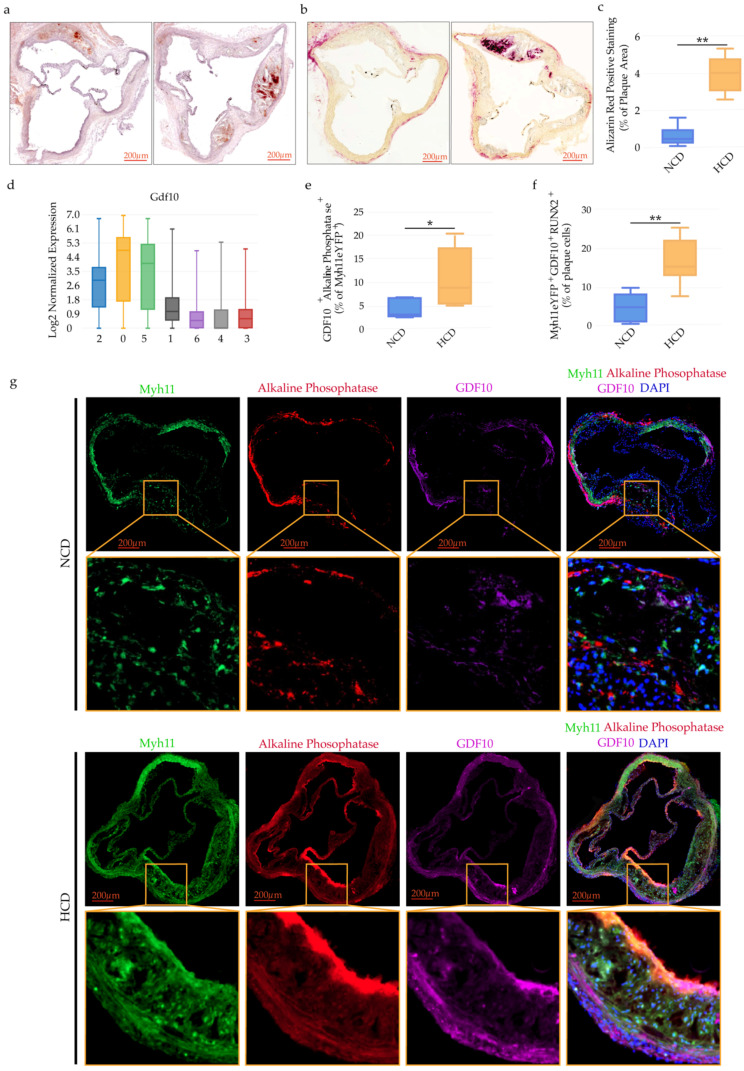

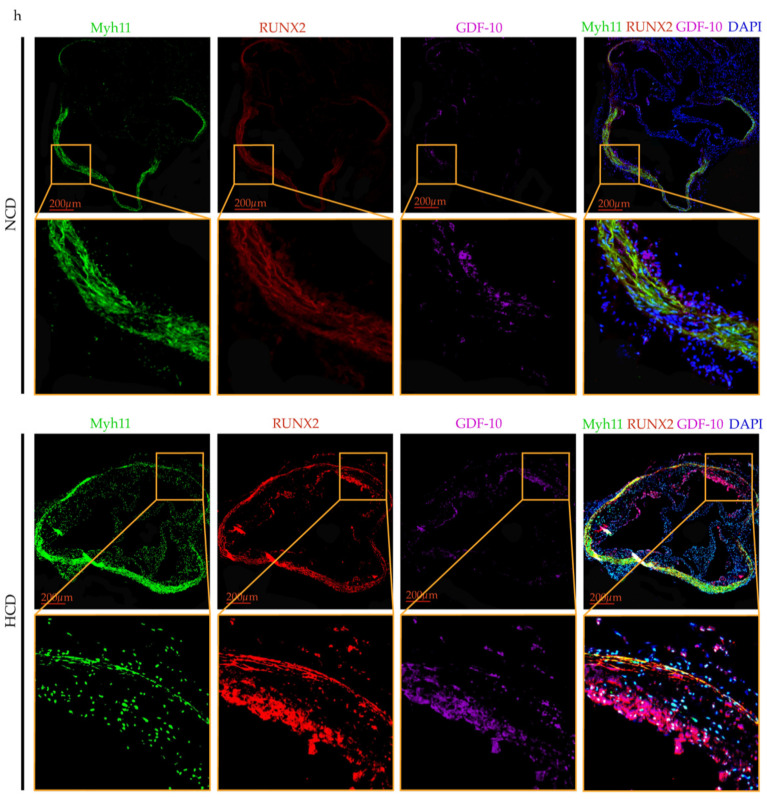
Vascular calcification and GDF10 expression in osteogenic-like VSMCs in mouse atherosclerotic plaques. Vascular calcification in aortic roots (**a**) as shown by representative images of Alizarin red staining of Apoe^−/−^ mice on NCD and HCD, scale bars: 200 μm and (**b**) representative images of Alkaline Phosphatase staining of Apoe^−/−^ mice on NCD and HCD, scale bars: 200 μm. (**c**) Bar graphs represent the mean ± SEM of the percentage of Alizarin red positively stained areas in the total atherosclerotic plaque area of aortic roots, *n* = 6–8 mice and **p < 0.01. (**d**) GDF10 expression among the seven clusters, *n* = 6 mice/group. (**e**) Bar graphs represent the mean ± SEM of GDF10^+^ Alkaline Phosphatase^+^ cells expressed as percentage of the total Myh11eYFP^+^ plaque cells, *n* = 6–8 mice and *p < 0.05. (**f**) Bar graphs represent the mean ± SEM of Myh11eYFP^+^ GDF10 ^+^ RUNX2^+^ cells expressed as percentage of the total plaque cells, *n* = 6–8 mice and **p < 0.01. (**g**) Representative immunofluorescence staining of GDF10 (purple) and Alkaline Phosphatase cells (red) and Myh11eYFP (green) expressing aortic root cells in Apoe^−/−^Myh11-CreERT2, ROSA26STOP-floxeYFP^+/+^ mice fed NCD or HCD. (**h**) Representative immunofluorescence staining of GDF10 (purple) and RUNX2 (red) and Myh11eYFP (green) expressing aortic root cells of Apoe^−/−^Myh11-CreERT2, ROSA26STOP-floxeYFP^+/+^ mice fed NCD or HCD.

**Figure 7:**
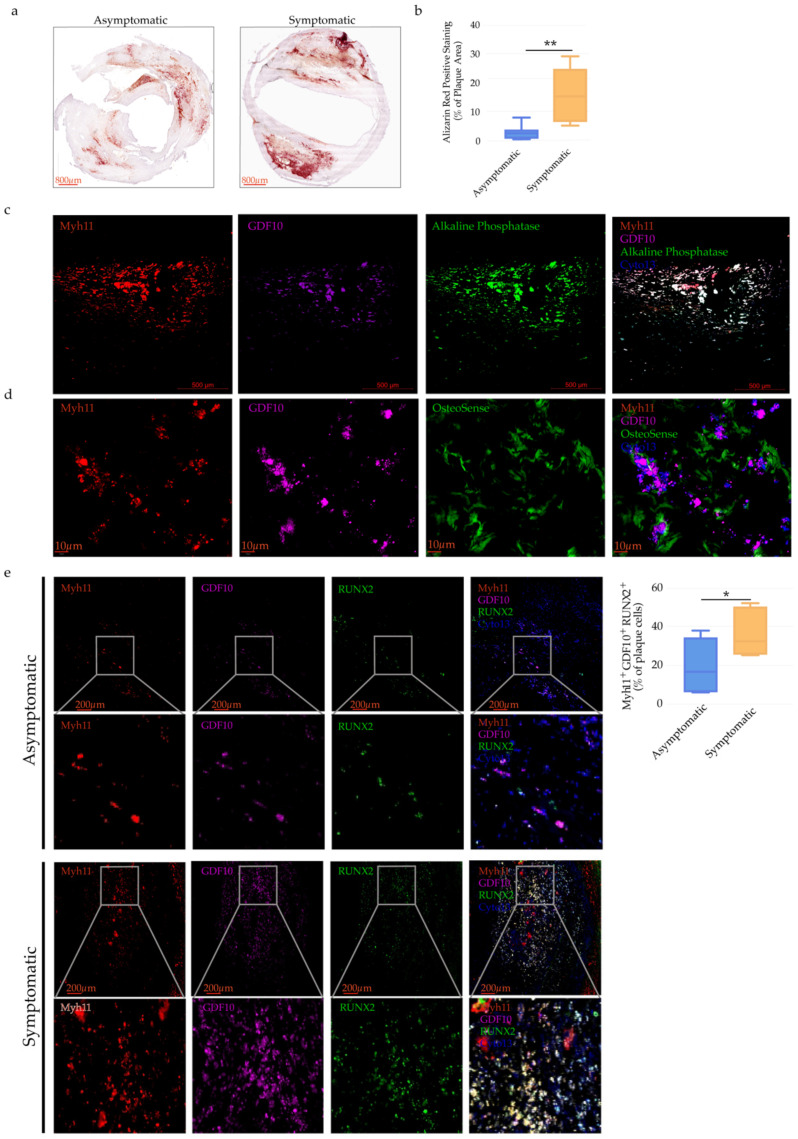
GDF10 expression in osteogenic-like VSMCs in human atherosclerotic Plaques. Vascular calcification as shown in the (**a**) representative images of Alizarin red staining of human atherosclerotic Plaques of asymptomatic and symptomatic CAD patients, respectively, scale bars: 800 μm. (**b**) Bar graphs represent quantification of vascular calcification of human atherosclerotic plaques expressed as the mean ± SEM of Alizarin red positively stained areas in the total atherosclerotic plaque area of asymptomatic and symptomatic CAD patients, respectively, *n* = 8 and **p < 0.01. (**c**) Representative immunofluorescence staining showing Myh11, GDF10 and Alkaline Phosphatase positive cells in area of vascular calcification in human atherosclerotic lesions of carotid artery tissue of CAD patient; Myh11 (red), GDF10 (purple) and Alkaline Phosphatase staining (green). (**d**) Representative immunofluorescence staining of Myh11 (red), GDF10 (purple) and OsteoSense (green) in area of vascular microcalcification in human atherosclerotic lesions of asymptomatic and symptomatic CAD patients. (**e**) Representative immunofluorescence staining of Myh11 (red), GDF10 (purple) and RUNX2 (green) expressing cells in human atherosclerotic lesions of asymptomatic and symptomatic CAD patients and bar graphs represent the mean ± SEM of Myh11^+^GDF10^+^RUNX2^+^ cells of asymptomatic and symptomatic CAD patients, respectively, *n* = 8 and *p < 0.01.

Atherosclerosis and vascular calcification remain the leading cause of death worldwide and there is a huge need to investigate mediators of osteogenic differentiation of VSMC as a major pathological process. The progress in the area is dependent on targeting cellular and soluble mediators promoting vascular calcification. In the search for a gene with a potentially causal role in VSMC osteogenic differentiation, the present study identified growth differentiation factor 10—GDF10 (BMP3b)—as a highly differentially expressed gene characterizing a population of VSMC with the macrophagic calcific phenotype (Figure 5a,b). GDF10 (BMP-3b) was shown to be involved in the regulation of cell differentiation, skeletal morphogenesis [55], ossification, osteoblast differentiation [56,57,58], as well as increased Alkaline Phosphatase activity [56]. Moreover, since GDF10 appeared to be one of the top differentially expressed genes of VSMC with the macrophagic calcific phenotype of cluster 0 (Figure 5a,b), we hypothesized that GDF10 is a gene with a potentially causal role in VSMC osteogenic phenotype differentiation. Calcified lesions were observed in the aortic root cryosections of Apoe^−/−^Myh11-CreERT2, ROSA26STOP-floxeYFP^+/+^ mice fed an NCD or HCD, (Figure 6a,b), with increased calcium deposit in the HCD fed mice as quantified by Alizarin Red staining (Figure 6c). Interestingly, we observed GDF10 expression in all phenotypically modified VSMC clusters 0, 1, 2 and 5 (Figure 6d), with the highest levels of expression in cluster 0 of VSMC with a macrophagic calcific phenotype. The reprogrammed osteogenic-like VSMCs (Myh11 (*eYFP*) in the aortic root cryosections of Apoe^−/−^Myh11-CreERT2, ROSA26STOP-floxeYFP^+/+^ mice fed an NCD or HCD co-expressed GDF10 and Myh11 (*eYFP*), in addition to known markers of VSMC osteogenic switch, such as Alkaline Phosphatase and the osteogenic transcription factor RUNX2 (Figure 6e–h), playing an important role in VSMC calcification [88]. In line with our findings, it has been shown that GDF10 which belongs to the BMP family, affects the transcriptional network in which the transcription factor RUNX2 plays essential role [89]. The number of osteogenic-like VSMCs co-expressing GDF-10 and Alkaline Phosphatase, as well as GDF10 and RUNX2, were significantly increased in the aortic root plaques of Apoe^−/−^Myh11-CreERT2,ROSA26STOP-floxeYFP^+/+^ mice fed an HCD versus Apoe^−/−^Myh11-CreERT2, ROSA26STOP-floxeYFP^+/+^ mice fed an NCD (Figure 6e–h). To explore a possible association of GDF10 and human vascular calcification and plaque destabilisation, we used human atherosclerotic plaques of symptomatic patients with ipsilateral ischemic stroke, as well as of asymptomatic patients (no history of ischemic symptoms) undergoing carotid endarterectomy (CEA) for severe carotid stenosis. We observed calcification in the atherosclerotic lesion of the CAD asymptomatic and symptomatic patients, (Figure 7a), with increased calcium deposit in symptomatic CAD patients (Figure 7b). Moreover, Myh11^+^GDF10^+^ cells were detected in areas of human atherosclerotic lesion microcalcification, as demonstrated by Alkaline Phosphatase (Figure 7c) and OsteoSense-positive staining, indicative of advanced calcification and colocalized preferentially with osteopontin-positive cells [90] (Figure 7d). Moreover, osteogenic-like VSMCs expressing Myh11^+^GDF10^+^RUNX2^+^ were detected in human atherosclerotic lesions, with a higher percentage of these cells present in endarterectomy carotid artery tissue of symptomatic patients who had experienced an ipsilateral ischemic stroke in comparison to asymptomatic patients (Figure 7e). Importantly, the present data shows that GFD10 expression in a population of osteogenic-like VSMCs was associated with CAD event occurrence in human atherosclerosis.

**Figure 8:**
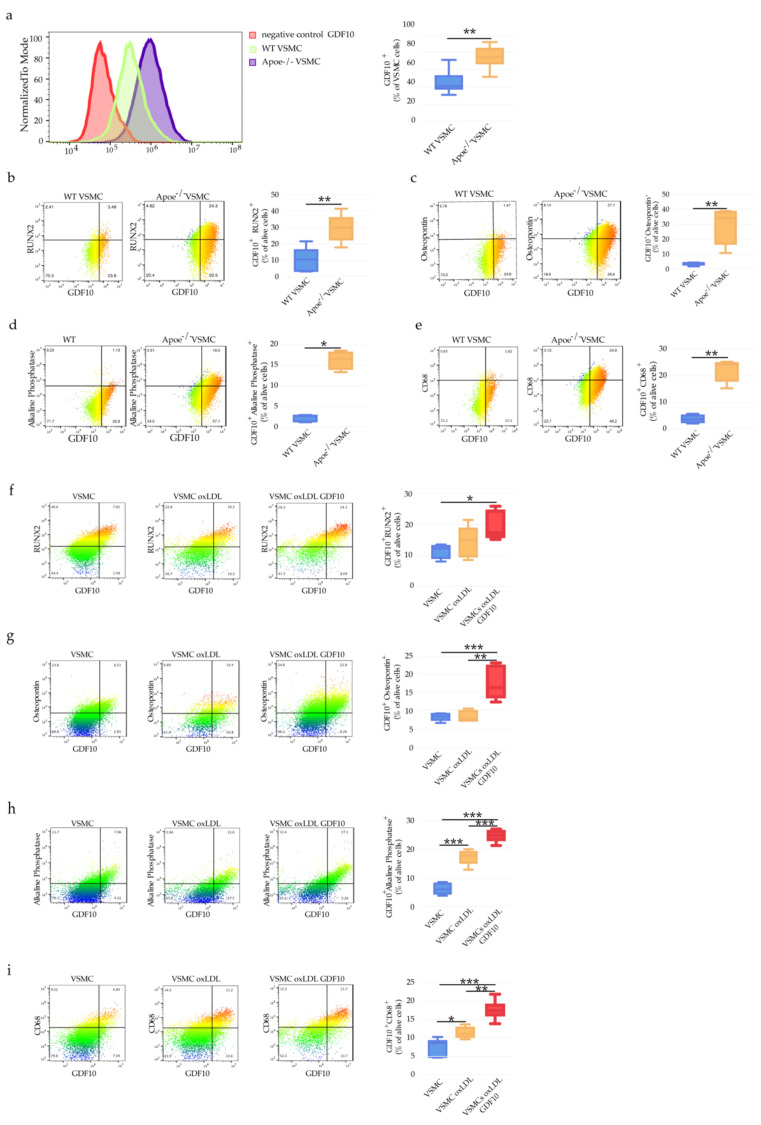
GDF10-associated VSMC phenotypic transition to osteogenic-like cells. (**a**) Representative flow cytometry histogram showing GDF10 staining in VSMC and bar graph representing the mean ± SEM of GDF10 expression in WT VSMC and Apoe^−/−^VSMC cells, *n* = 6 and **p < 0.01. (**b**) Representative dot plots and bar graph representing the mean ± SEM of GDF10^+^RUNX2^+^ WT VSMC and Apoe^−/−^VSMC cells, *n* = 6 and **p < 0.01. (**c**) GDF10^+^Osteopontin^+^ WT VSMC and Apoe^−/−^VSMC cells, *n* = 6 and **p < 0.01. (**d**) GDF10^+^Alkaline Phosphatese^+^ WT VSMC and Apoe^−/−^VSMC cells, n = 6 and *p < 0.05. (**e**) GDF10^+^CD68^+^ WT VSMC and Apoe^−/−^VSMC, *n* = 6 and **p < 0.01. (**f**) GDF10^+^ RUNX2^+^ VSMC, VSMC oxLDL and VSMC oxLDL GDF10 cells quantified by flow cytometry, *n* = 6 and *p < 0.01. (**g**) GDF10^+^Osteopontin^+^ VSMC, VSMC oxLDL and VSMC oxLDL GDF10 cells, *n* = 6, **p < 0.01 and ***p < 0.001. (**h**) GDF-10^+^Osteopotin^+^ VSMC, VSMC oxLDL and VSMC oxLDL GDF-10 cells, *n* = 6 and ***p < 0.01. (**i**) GDF10^+^Osteopontin^+^ positive VSMC, VSMC oxLDL and VSMC oxLDL GDF-10 cells, bar graph represents the mean ± SEM of GDF10^+^Osteopontin^+^ VSMC, VSMC oxLDL and VSMC oxLDL GDF10 cells quantified by flow cytometry, *n* = 6, *p < 0.05, **p < 0.01 and ***p < 0.001.

Interestingly, we demonstrated that VSMCs expresse GDF10 (Figure 8a) and co-expressed known markers of VSMCs transdifferentiation to osteo/chondrogenic phenotype like osteochondrogenic markers—Runx2, osteopontin and Alkaline Phosphatase [10] (Figure 8b-d). VSMCs isolated from Apoe^−/−^ mice showed pronounced co-expression of GDF10 and RUNX2, Alkaline Phosphatase, osteopontin as well as macrophage marker CD68 in comparison to VSMC derived from WT mice (Figure 8b–e). Furthermore, to demonstrate the causative role of GDF10 as an inducer of VSMC phenotypic switch to VSMC with macrophagic calcific phenotype, we used oxLDL and GDF10 to treat WT VSMCs. The supplementation in vitro exclusively of GDF10 in combination with oxLDL resulted in a significant increase in the percentage of GDF10-positive cells expressing RUNX2, a master transcription factor of VSMCs osteoblast differentiation versus unstimulated VSMCs (Figure 8f). The percentage of GDF10 positive cells expressing osteopontin, Alkaline Phosphatase and CD68 was also augmented upon GDF10 and oxLDL stimulation (Figure 8g–i). Thus, under hypercholesteremic conditions, GDF10 may serve as a paracrine mediator working in an autocrine manner and promoting VSMC transition to osteogenic-like cells.

## 3. Discussion

Cardiovascular diseases (CVD) are still the predominant cause of death and morbidity in developed countries [1,2]. The atherosclerotic environment triggers complex phenotypic changes in VSMCs, leading to the acquisition of multiple phenotypes with distinct roles in atherosclerotic lesion pathogenesis, some beneficial and some detrimental [13,14]. Taking into account the atherosclerosis site-specific predisposition, we revealed disease-associated cell populations, genes, pathways and molecular determinants with a potential disease-causative role.

An HCD promoted profound cellular response to cell stress, lipids, cytokines, apoptosis and cell death, actin cytoskeleton organization involved in VSMC migration in response to vascular disease [91] and with a potential role in VSMC phenotypic modulation, whose abrogation could be beneficial in controlling VSMC phenotypic modulation. Hypercholesteremia clearly triggers an atherosclerosis-associated gene expression profile independently of the predisposition to atherosclerosis development. However, it appears that the fine balance between atheropromoting and atheroprotective molecular mediators determines the progression of the disease and the identified genes may emerge to be critical determinants of atherosclerosis disease susceptibility or protection.

The transcriptional profiles of the identified seven clusters mirrored the site-specific predisposition to atherosclerosis. AA&R clusters showed impressive phenotypic plasticity and diversity of phenotypically modified VSMCs that acquired VSMC macrophagic calcific phenotype, VSMC mesenchymal chondrogenic phenotype, VSMC inflammatory and fibro-phenotype and VSMC inflammatory phenotype from an early stage of atherosclerosis. The results of the present study greatly extend the characterization of the disease-relevant gene signatures of phenotypically-modulated VSMCs by emphasizing the complexity of VSMC phenotypic transition during atherosclerosis progression. In contrast, the clusters derived from the atherosclerosis-resistant DT aorta exhibited transcriptional profiles of VSMC-preserved contractile phenotype, with the induction of genes linked to vascular pathology protection, cholesterol efflux, as well as positive regulation of the genes suppressing inflammation, apoptosis and thrombosis.

Vascular calcification is a dynamic pathophysiological process, principally driven by VSMCs and linked to an increased risk of heart disease, stroke, atherosclerotic plaque rupture, vessel stiffness, systolic hypertension, diastolic dysfunction and heart failure and regarded as a prognostic marker of cardiovascular morbidity and mortality [10]. The presence of vascular calcification was proven predictive of all-cause mortality in a 15-year follow-up study of a cohort of 9715 adults, all-cause mortality was 3% in patients without coronary artery calcification in contrast to 28% in individuals with high levels of coronary artery calcification [92]. However, human plaques have complex features of calcification [93,94] and accumulating evidence from clinical and preclinical studies point out that the extent and the type of calcification determine beneficial or detrimental effects. For example, microcalcifications have been linked to higher degrees of inflammation and it could be found in thin fibrous caps and areas of macrophages accumulation and was associated with plaque rupture, while calcified nodules lead to thrombosis [95]. It is considered that presence of calcium (small, fragmented, spotty) is a predictor of unstable plaque while plaque with heavy calcium (diffuse, fibrocalcific plaques, sheet of calcium) is predominatly a predictor of plaque stability [95].

Current vascular calcification therapies only modulate those factors associated with the development of the disease, and there is currently no efficient therapy to directly target mediators of VSMC transdifferentiation from a contractile into an osteoblast-like phenotype. Therefore, progress in the area is dependent on targeting cellular and soluble mediators promoting VSMC phenotypic switch that subsequently causes myocardial infarction, stroke or heart failure through vascular calcification, vessel stiffness and plaque destabilization.

The present study was able to identify GDF10, linked to skeletal morphogenesis bone formation [55,56], ossification and osteoblast differentiation [56,57,58]. Interestingly, in genome-wide association (GWA) studies, single-nucleotide polymorphism of GDF10 was linked to blood pressure loci and shown to influence lipid metabolism following a high-fat meal [96,97]. The present study further extended the knowledge about GDF10 and its role in cardiovascular pathology showing that GDF10 could mediate VSMC transdifferentiation to osteogenic-like cells, with a likely detrimental role in atherosclerosis plaque stability.

Importantly, GDF10 was expressed in areas of vascular calcification and by VSMCs transdifferentiating to osteoblasts such as cells as indicated by the co-expression of GDF10 with Alkaline Phosphatase (the phenotypic marker for osteoblastic differentiation) and RUNX2 (the transcription factor for osteoblastic differentiation and bone formation) [98,99,100]. Furthermore, it appears that GDF10 is not only a marker of cells fated to become VSMC osteoblast-like cells, but promotes VSMC phenotypic modulation to osteoblasts-like cells associated with a higher risk of CAD events.

Interestingly, a recent study has demonstrated that the predominant and the earliest conversion of VSMCs was to osteoblast/chondrocyte lineages [101], while Aryl hydrocarbon receptor pathway activation diminished atherosclerosis disease-related VSMC switch to chondrocyte-like cells [102]. Those and the finding of the present study contribute to our understanding of how the VSMC response to the atherosclerosis disease triggers is regulated. Those studies emphasize the importance of VSMC phenotypic modulation to osteoblast/chondrocyte lineage and demonstrate its complex mechanisms of regulation and the involvement of a multitude of specific mediators. The finding of the present study identified osteoblast factor GDF10 as one of the specific factors implicated in the regulation of the VSMCs osteoblast/chondrocyte switch with likely detrimental implications in atherosclerosis disease severity. In conclusion, our results show that osteoblast factor GDF10 has a likely detrimental role given our finding that GDF10-positive VSMC osteoblast-like cells are present in areas of human vascular calcification and are associated with CAD events in human atherosclerosis. The genes and signaling pathways revealed by the present study are a valuable resource for research and should help to further explore and target distinct dedifferentiated VSMC populations or mediators with implications in atherosclerotic disease susceptibility, diagnosis, and treatment. Our findings may open a new area for the development of novel anti-calcification therapeutics.

## 4. Materials and Methods

### 4.1. Animals

Eleven-week-old male Apoe^−/−^C57Bl/6 mice or Apoe^−/−^Myh11-CreERT2, ROSA26 STOP-floxeYFP^+/+^ mice were fed an NCD (4.6% fat, 21.1% protein, 4.5% Fibre, 6.4% ash, Special Diets Services, Essex, UK) for 16 weeks [103] or an HCD for 11 weeks (20.1% fat, 1.25% cholesterol, Research Diets, Inc., New Brunswick, NJ, USA) [104]. To facilitate VSMC lineage tracing, injection of tamoxifen was used to induce Cre recombinase activation in male Apoe^−/−^Myh11-CreERT2, ROSA26STOP-flox eYFP^+/+^. A series of ten intraperitoneal 1 mg tamoxifen (Sigma, St. Louis, MO, USA) injections from 8 to 9 weeks of age, an average body weight of 25 g for the 2 weeks prior to the high cholesterol diet was performed and triggered permanent labeling with eYFP fluorescence of all VSMCs and their progeny independently of any phenotypic modulation [105]. Whole blood was collected and total cholesterol, low-density lipoprotein-cholesterol (LDL-C) were measured. Animals were sacrificed by exsanguination after anesthesia with 4% isoflurane. Experimental protocols and procedures were approved by the Institutional Animal Care and Use Committee of the Geneva University School of Medicine. Animal care and experimental procedures were carried out in accordance with the guidelines of the Institutional Animal Care and Use Committee of the Geneva University School of Medicine. All procedures conform to the guidelines from Directive 2010/63/EU.

### 4.2. Tissue Processing, Cell Staining and Flow Cytometry

After intracardiac perfusion of Apoe^−/−^C57Bl/6 mice on NCD or HCD, the aorta was surgically excised (*n* = 6 mice per group). The aorta adventitia was carefully excised by sharp surgical dissection in a clearly defined plane, to leave a naked media over the length of the AA&R (the aortal segment from just left of the branchpoint for the brachiocephalic artery to just right of the branch point for the left subclavian artery) and DT aorta (the straight aortal segment from after the arch to the renal aortas) were separated. The segments obtained from the AA&R and the DT aorta were digested separately at 37 °C in DMEM containing Collagenase P, dispase and DNaseI. The cell suspension individually obtained from the AA&R and DT aorta was passed through a 70 μm cell strainer with anti-mouse CD45-PE (Biolegend, clone 30-F11), LIVE/DEAD Fixable Near-IR Dead Cell Dye (Thermo Fisher, Waltham, MA, USA) and Hoechst 33342 (Thermo Fisher, Waltham, MA, USA) fluorescent dyes to exclude cell debris. CD45^−^ cells were then selected from the total viable AA&R and DT aorta cells using Beckman Coulter’s MoFlo Astrios EQ for scRNA-sequencing. The aortic roots of male Apoe^−/−^Myh11-CreERT2, ROSA26STOP-flox eYFP^+/+^ mice fed an NCD or HCD were embedded in OCT and serially cut into 7 μm sections.

### 4.3. scRNAseq

The total viable CD45^−^ cells from the AA&R and DT aorta were loaded separately on C1 Single-Cell mRNA Seq HT IFC chip (10–17 μm) to automatically isolate individual cells to separate reaction chambers. After labeling each cell and microscopy imaging the C1 chips to confirm cell count of about 70% capture efficiency, each cell was lysed for RNA amplification and cDNA synthesis using the C1™ Single-Cell mRNA SeqHT Reagent Kit (Fluidigm, South San Francisco (HQ), CA, USA). The Illumina Nextera XT DNA Sample Preparation Kit (Illumina, San Diego, CA, USA) was used for library preparation. Ten million reads from individual cells were acquired for the relative quantitation of mRNA expression on an Illumina sequencer.

### 4.4. scRNAseq Data Analysis

De-multiplexed FASTQ pairs were generated, UMI-tools were used to assign cell and UMI barcodes to each read, and reads were trimmed using Cutadapt [106]. Quality scores were assessed using FastQC [107]. Reads were aligned to the Mus musculus genome build mm10 using STAR [108]. Individual sample reads were quantified using HTseq [109]. Alignments were de-duplicated with UMI-tools on the gene level, and such reads were grouped together if they shared the same UMI and gene. Cells with more than 1.5% of reads aligning to mitochondrial-encoded genes were discarded. Resulting counts were analyzed using Seurat 3.1.1. Counts were normalized using “SCTransform” and analysis used 10 and 7 dimensions, respectively for the “FindNeighbors” functions. Clusters were visualized using t-SNE. The Rosalind™ RNA-seq assay was used (https://rosalind.onramp.bio/, accessed on 20 January 2020), with a HyperScale architecture developed by OnRamp BioInformatics, Inc. (San Diego, CA, USA). Reads were trimmed using Cutadapt [106,107]. Quality scores were assessed using FastQ^20^. Individual sample reads were quantified using HTseq [109] and normalized via Relative Log Expression using DESeq2 R library. Read Distribution percentages, violin plots, identity heatmaps, and sample MDS plots were generated as part of the QC step using RSeQC [110]. DEseq2 was also used to calculate fold changes and p-values. Clustering of genes for the final heatmap of differentially expressed genes was carried out using the partitioning around medoids method using the fpc R library [111]. Functional enrichment analysis of pathways, gene ontology, domain structure and other ontologies was performed using HOMER [112]. Several database sources were referenced for enrichment analysis, including Interpro [113], NCBI [114], KEGG [115,116,117], MSigDB [118,119], REACTOME [120], WikiPathways [121]. Enrichment was calculated relative to a set of background genes relevant for the experiment. Additional gene enrichment is available from the following partner institution: Advaita (http://www.advaitabio.com/ipathwayguide, accessed on 15 November 2019).

### 4.5. Human Samples

Specimens of internal carotid plaques of a previously published cohort study [122,123] from CAD symptomatic patients with ipsilateral ischemic stroke, as well as of asymptomatic patients (no history of ischemic symptoms) undergoing CEA for severe carotid stenosis were used for immunofluorescent analysis. CEA was performed due to extra cranial high-grade internal carotid stenosis (>70% luminal narrowing) using the criteria of the North American Symptomatic Carotid Endarterectomy Trial [124]. The indication for CEA for asymptomatic patients was based on Asymptomatic Carotid Surgery Trial [125] while for symptomatic patients, CEA indication followed the European Carotid Surgery Trial (ECST) [126] and the North American Symptomatic Carotid Endarterectomy Trial (NASCET) [125]. After surgical excision, the internal carotid plaque specimens were cut perpendicular to the long axis through the point of maximum stenosis to obtain the atherosclerotic plaque upstream to the blood flow. The upstream internal carotid plaque specimens from symptomatic and asymptomatic patients were embedded in optimal cutting temperature compound (OCT). The study was approved by the Medical Ethics Committee of San Martino Hospital in Genoa (Italy) and conducted in compliance with the Declaration of Helsinki after participants provided written informed consent.

### 4.6. Histological and Immunohistochemical Staining

Embedded in OCT serially cut into 7 μm sections of AA&R and the DT aorta of Apoe^−/−^Myh11-CreERT2, ROSA26,STOP-floxeYFP+/+ mice on NCD and HCD were stained with Oil Red O staining for lipid content quantification, as previously described [127]. The sections were counter-stained with Mayer’s hemalum solution and rinsed in distilled water. Quantification was performed using the Definiens Tissue Studio software (Definiens Inc., Munich, Germany). Data were calculated as the percentage of the stained area from total lesion area. Embedded in OCT serially cut into 7 μm sections of aorta of Apoe^−/−^Myh11-CreERT2, ROSA26, STOP-floxeYFP+/+ mice on NCD and HCD were preincubated with 5% normal serum and then incubated with primary antibodies for 16 h at 4 °C. The sections were then reacted with Alkaline Phosphatase antibody from Vector Laboratories for 2 h at 4 °C. These sections were visualized with an Alkaline Phosphatase substrate kit (Vector Laboratories, Burlingame, CA, USA) according to the manufacturer’s instructions. Levamisole (Vector Laboratories) was used as the inhibitor of endogenous Alkaline Phosphatase. To detect calcium, sections were rehydrated in water for 2 min, stained in 40 nM Alizarin red Staining Solution (Sigma Aldrich, St. Louis, MO, USA), pH 4.2, for 6 min, rinsed in distilled water followed by 3 changes of phosphate-buffered saline, pH 7.4, rinsed in Neoclear (VWR International, Radnor, PA, USA) 2 times and Slides were then air-dried and mounted in Neo-Mount (VWR International).

### 4.7. Immunofluorescent Staining and Quantification

Internal carotid plaque specimens from symptomatic and asymptomatic patients and the aortic roots of male Apoe*^−/−^Myh11-CreERT2*, *ROSA26*,*STOP-floxeYFP^+/+^* mice on NCD and HCD were embedded in OCT serially cut into 7μm sections. Cryosections were fixed in 1% paraformaldehyde and then washed with 1xPBS and incubated with blocking solution, consisting of 5% BSA in PBS for 30 min, then permeabilized with Triton X-100 0.1%. Aortic roots cryosections of Apoe^−/−^Myh11-CreERT2,ROSA26, STOP-floxeYFP^+/+^ mice (*n* = 8 mice per group) were stained with primary rabbit anti-GDF10 (Thermo Fischer, Waltham, MA, USA), RUNX2 (Novusbio, Centennial, CO, USA), and cell-permeant SYTO Orange Fluorescent Nucleic Acid Stain (Thermo Fischer, Waltham, MA, USA). The secondary antibody used was Alexa 647 anti-rabbit (Thermo Fischer), DyLight 405 followed by mounting with ProLong Glass Antifade Mountant (Thermo Fischer, Waltham, MA, USA). Immunofluorescent images were acquired with Axioscan Z1 microscopy and analyzed with QuPath software. Endarterectomy specimens from asymptomatic and symptomatic patients (*n* = 12 samples per group) were stained with primary rabbit anti-GDF10 (Thermo Fischer, Waltham, MA, USA), RUNX2 (Novusbio), cell-permeant SYTO 13 green fluorescent nucleic acid stain (Thermo Fischer, Waltham, MA, USA), anti-mouse Myh11 (Thermo Fischer) and OsteoSense 680EX Fluorescent Imaging Agent (PerkinElmer, Waltham, MA, USA) for micro-calcification visualization or Alkaline Phosphatase substrate kit (Vector Laboratories). The secondary antibody PE anti-rabbit (Thermo Fischer), Alexa 647 anti-mouse (Thermo Fischer, Waltham, MA, USA), DyLight 405 and mounting with ProLong Glass Antifade Mountant (Thermo Fischer, Waltham, MA, USA) were used. Immunofluorescent images were acquired with Axioscan Z1 microscopy and quantified with QuPath software.

### 4.8. Isolation and Culture of Primary VSMCs

The aorta adventitia was carefully excised by sharp surgical dissection in a clearly defined plane, to leave naked media over the length of AA&R derived from male C57Bl/6 mice and Apoe^−/−^C57Bl/6 mice. VSMC were isolated from the AA&R via digestion at 37 °C in DMEM containing Collagenase P, dispase and DNaseI. VSMC phenotype was confirmed by flowcytometry analysis (smooth muscle α-actin^+^, Myh11^+^, CD31^−^ (endothelial cell marker) and CD90^−^ (fibroblasts cell marker) data not shown). These cells were in SmGMTM Basal Medium (CC-3181, Lonza, Basel, Switzerland) supplemented with SmGMTM-2 SingleQuotsTM (CC-4149, Lonza, Basel, Switzerland).

### 4.9. VSMC Osteoblast-like Cell Transdifferentiation

VSMC derived from AA&R of male C57Bl/6 and Apoe^−/−^ C57Bl/6 mice were stimulated with either 40 ng/mL oxLDL or/and 150 ng/mL of GDF10 for 7 days. Quantification of VSMC osteoblast-like cell transdifferentiation was performed via flow cytometry analysis of RUNX2 DyLight 405 (NOVUS), Alkaline Phosphatase APC (NOVUS), osteopontin PE (R&D), GDF10 Alexa 700 (Thermo Fisher) and CD68 PerCP/Cyanine5.5 (Biolegend, San Diego, CA, USA), excluding dead cells via LIVE/DEAD Fixable Near-IR Dead Cell Dye (Thermo Fisher, Waltham, MA, USA). Samples were acquired in CytoFLEX (Beckman Coulter, Brea, CA, USA) and analyzed with FlowJo software (TreeStar, Version 10.5.3, Ashland, OR, USA).

### 4.10. Statistical Analysis

Statistics were performed using GraphPad Prism 8. for Mac OS X (GraphPad Software, Inc., La Jolla, CA, USA). For the comparison of 2 groups of continuous variables with normal distribution and equal variances, 2-tailed unpaired Student *t*-tests were performed with a significance threshold of *p* ≤ 0.05. For multiple group comparison, we performed 1-way ANOVA. DEseq2 was used to calculate fold changes and *p*-values and perform optional covariate correction.

## Data Availability

Data are contained within the article or supplementary material. Additional data that support the findings of this study are available from the corresponding author upon reasonable request.

## Supplementary Materials

The following supporting information can be downloaded at: https://www.mdpi.com/article/10.3390/ijms23031796/s1.
