# Supplementary material for: Single-Cell Analysis Uncovers Osteoblast Factor Growth Differentiation Factor 10 as Mediator of Vascular Smooth Muscle Cell Phenotypic Modulation Associated with Plaque Rupture in Human Carotid Artery Disease
Source: Int J Mol Sci. 2022 Feb 4;23(3):1796. doi: 10.3390/ijms23031796 (PMC8836240; doi:10.3390/ijms23031796)
Supplement: Supplementary file 1 [file ijms-23-01796-s001.zip › ijms-1539603-supplementary materials final.pdf]

Supplementary Materials

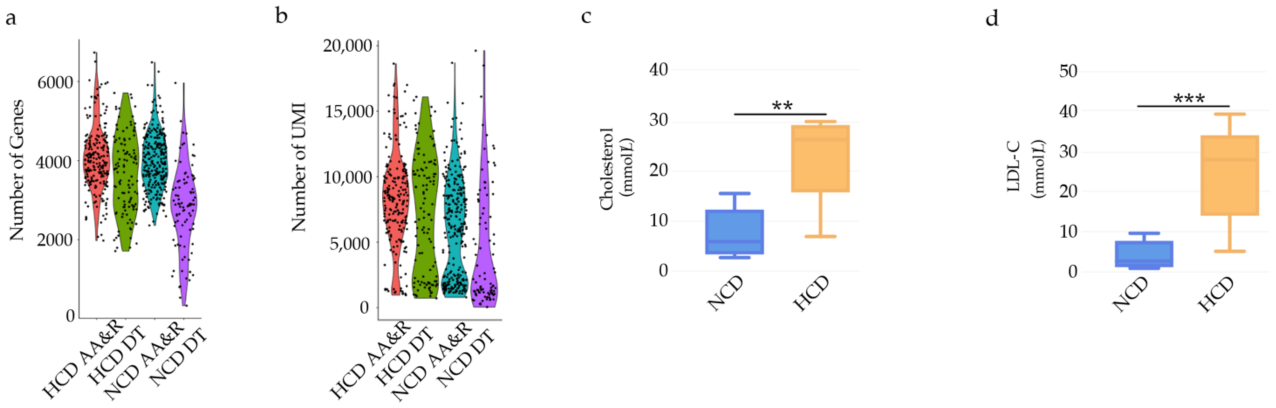

Figure S1. Quality control of the profiled scRNAseq transcriptomes of CD45$^-$ cells derived from atherosclerosis-prone AA&R, and atherosclerosis-resistant DT aorta of Apoe$^{-/-}$ mice on NCD and HCD and cholesterol levels. (**a**) Violin plots showing the quality control metrics of a number of genes per cell and (**b**) unique molecular identifiers (UMI) per cell, $n = 6$ mice. (**c**) Bar graphs represent the mean ± SEM (**c**) cholesterol; (**d**) LDL-C, $n = 6$ mice/group, $^{**}p < 0.01$ and $^{***}p < 0.001$.

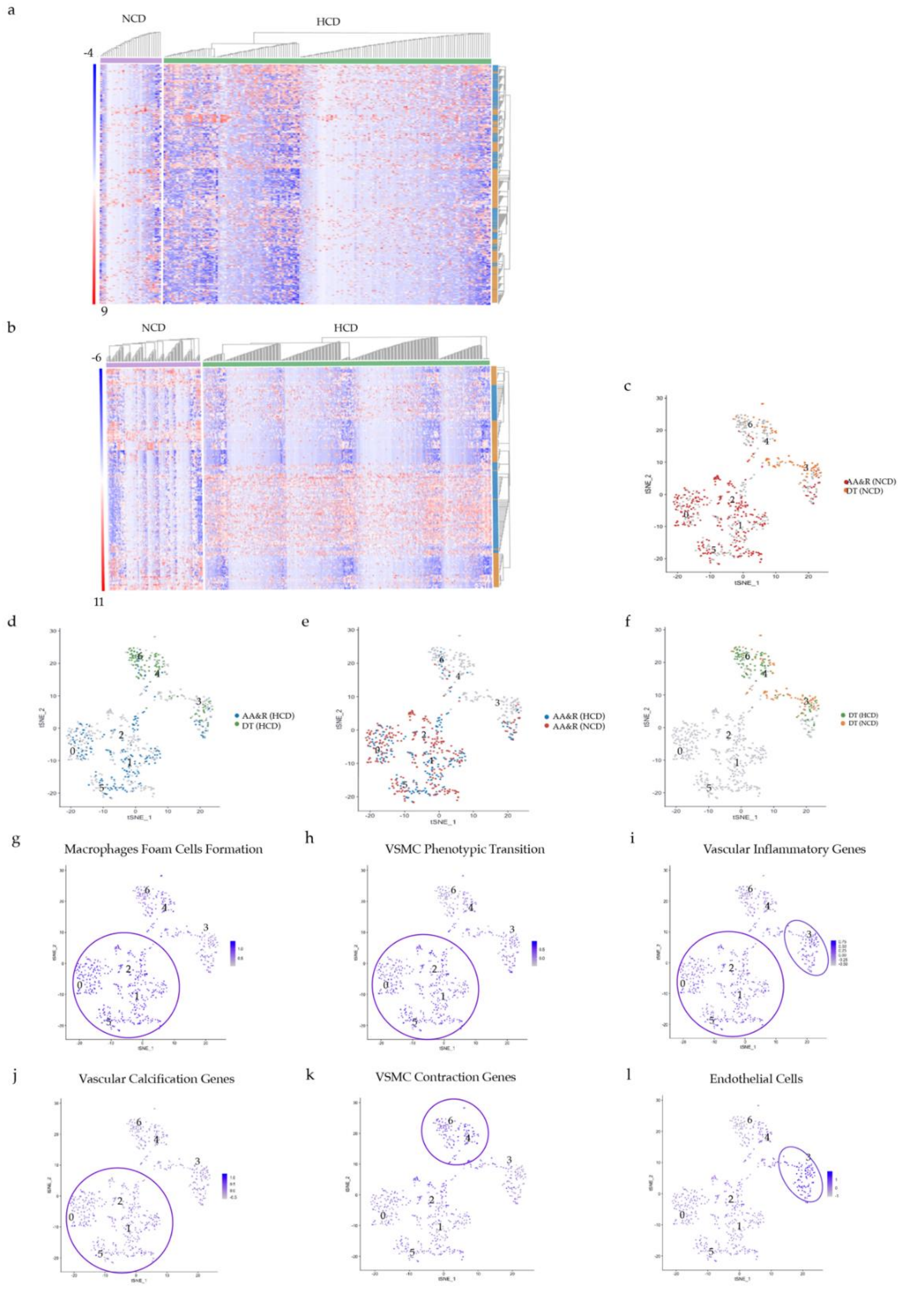

Figure S2. (**a**) Heatmap showing the expression of genes CD45^{-} cells derived from AA&R of Apoe$^{-/-}$ on NCD versus HCD (HCD, red side strip or NCD, purple side strip) (p-Adj<0.05), *n*=6 mice/group. Rows represent genes and columns represent cells. (**b**) Heatmap showing the expression of genes CD45^{-} cells derived from DT of Apoe$^{-/-}$ on NCD versus HCD (HCD, red side strip or NCD, purple side strip) (p-Adj<0.05), *n*=6 mice/group. Rows represent genes and columns represent cells. t-distributed stochastic neighbor embedding (tSNE) plot showing: (**c**) AA&R and DT aorta clusters of Apoe$^{-/-}$ mice on NCD; (**d**) AA&R and DT aorta of Apoe$^{-/-}$ mice on HCD; (**e**) AA&R clusters of Apoe$^{-/-}$ mice on HCD and NCD; (**f**) DT clusters of Apoe$^{-/-}$ mice on HCD and NCD. Predefined gene sets were overlaid on single cells on a tSNE plot to identify the cell identity of clusters with an enrichment of indicated gene sets (**g**) Macrophage foam cell formation; (**h**) VSMC phenotypic transition; (**i**) Vascular inflammation genes; (**j**) Vascular calcification gene; (**k**) VSMC contraction genes; (**l**) Endothelial cell.

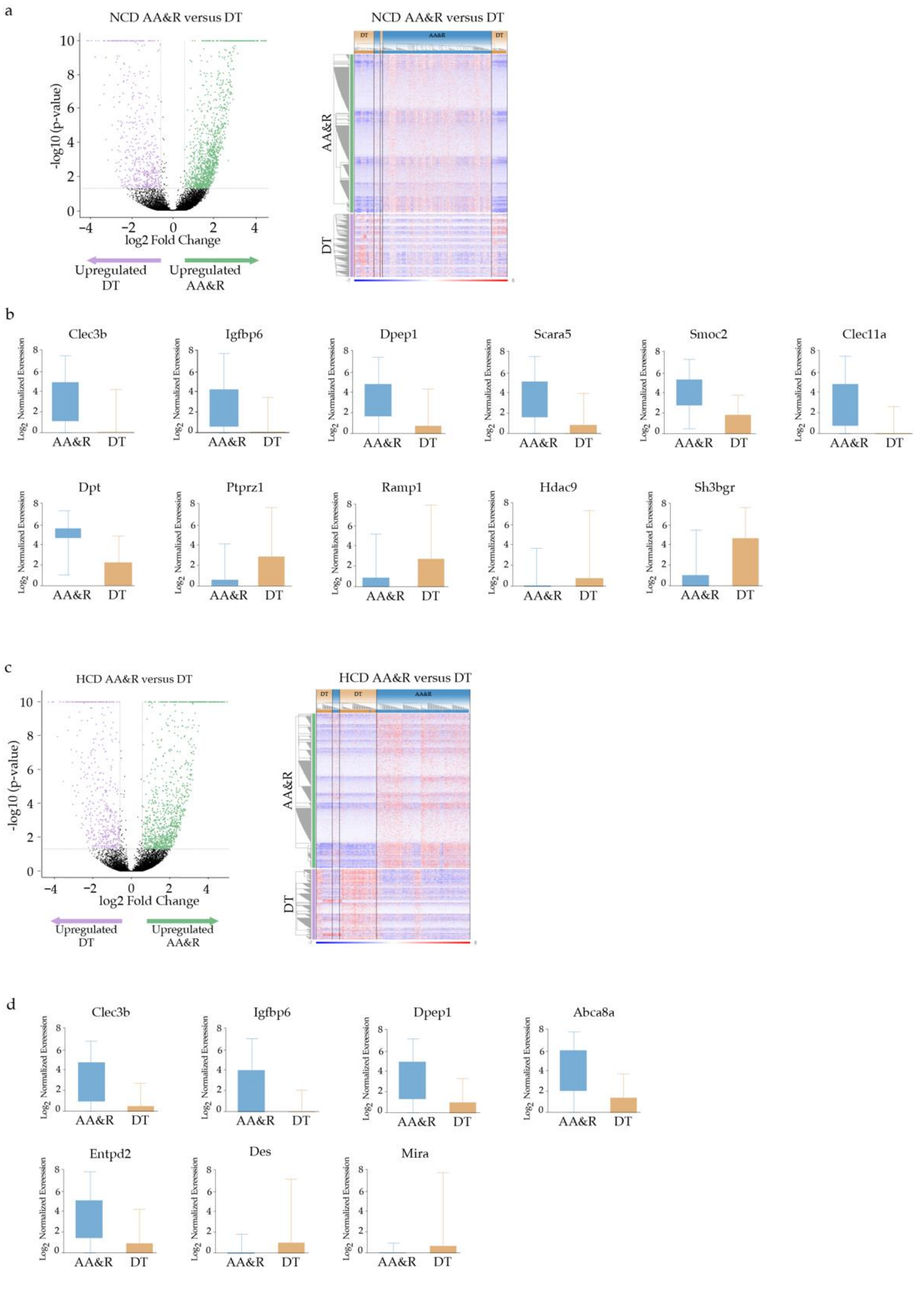

Figure S3. (**a**) Volcano plot showing significance (−log10 *p*-value) versus relative gene expression of CD45^{-} cells derived from AA&R of Apoe$^{-/-}$ versus DT derived from Apoe$^{-/-}$ on NCD. Each dot represents a gene within the performed comparison (*p*-Adj<0.05, Log2>±4), *n* = 6 mice. Rows represent genes and columns represent cells. Heatmap showing the expression of genes (HCD, red side strip or NCD, purple side strip) (p-Adj<0.05). (**b**) Genes of interest with significant differences in expression of CD45^{-} cells derived from AA&R of Apoe$^{-/-}$ versus DT derived from Apoe$^{-/-}$ on NCD are labelled and presented as graphs, (*p*-Adj<0.05), *n* = 6 mice. (**c**) Volcano plot showing significance (−log10 *p*-value) versus relative gene expression of CD45^{-} cells derived from AA&R of Apoe$^{-/-}$ versus DT derived from Apoe$^{-/-}$ on HCD. Each dot represents a gene within the performed comparison (*p*-Adj<0.05, Log2>±4), *n*=6 mice. Rows represent genes and columns represent cells. Heatmap showing the expression of genes (*p*-Adj<0.05). (**d**) Genes of interest with highly significant differences in expression of CD45^{-} cells derived from AA&R of Apoe$^{-/-}$ versus DT derived from Apoe$^{-/-}$ on HCD are labelled and presented as graphs, (*p*-Adj<0.05), *n* = 6 mice.

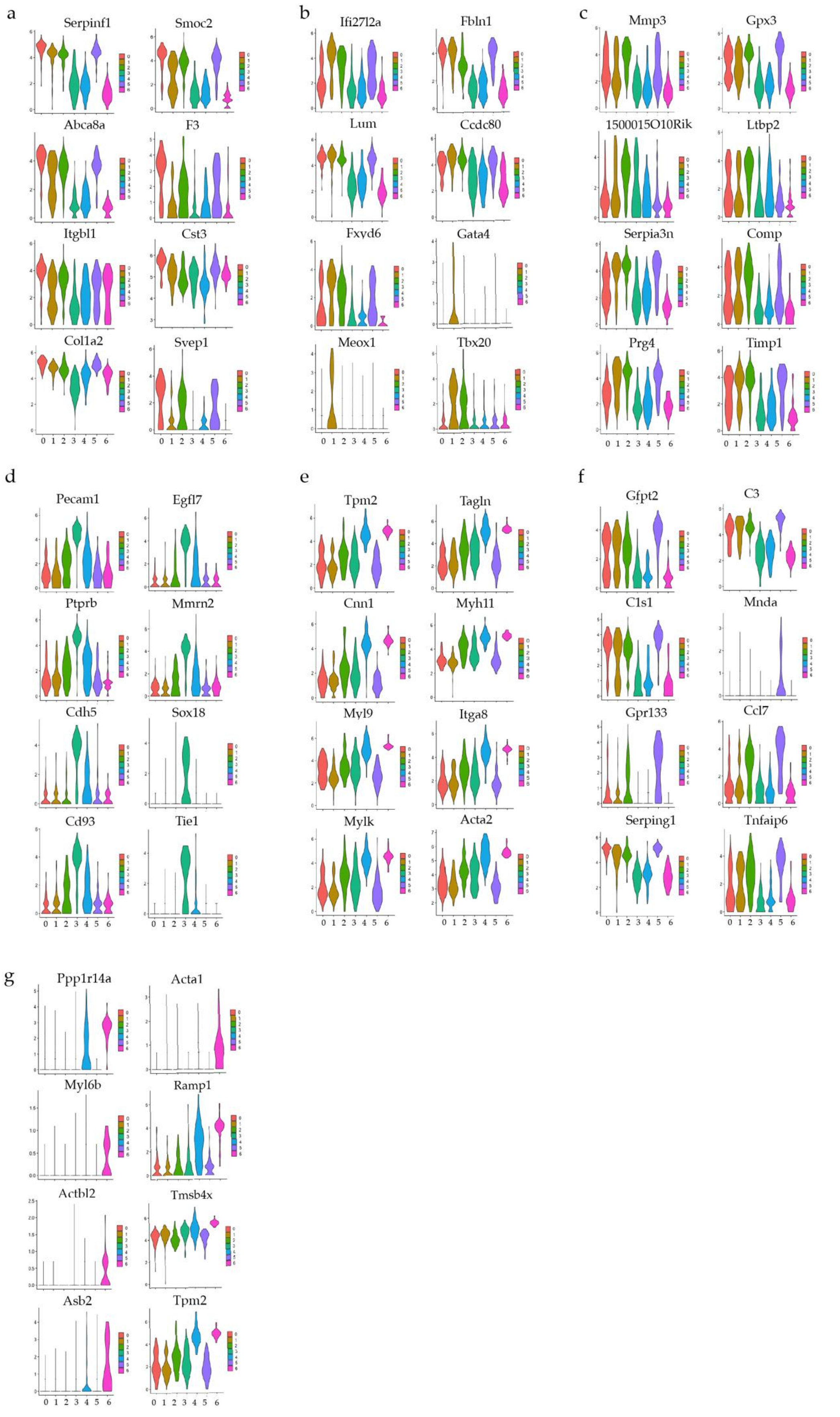

Figure S4. Top eight expressed genes per cluster: (**a**) cluster 0; (**b**) cluster 1; (**c**) cluster 2; (**d**) cluster 3; (**e**) cluster 4; (**f**) cluster 5; (**g**) cluster 6.

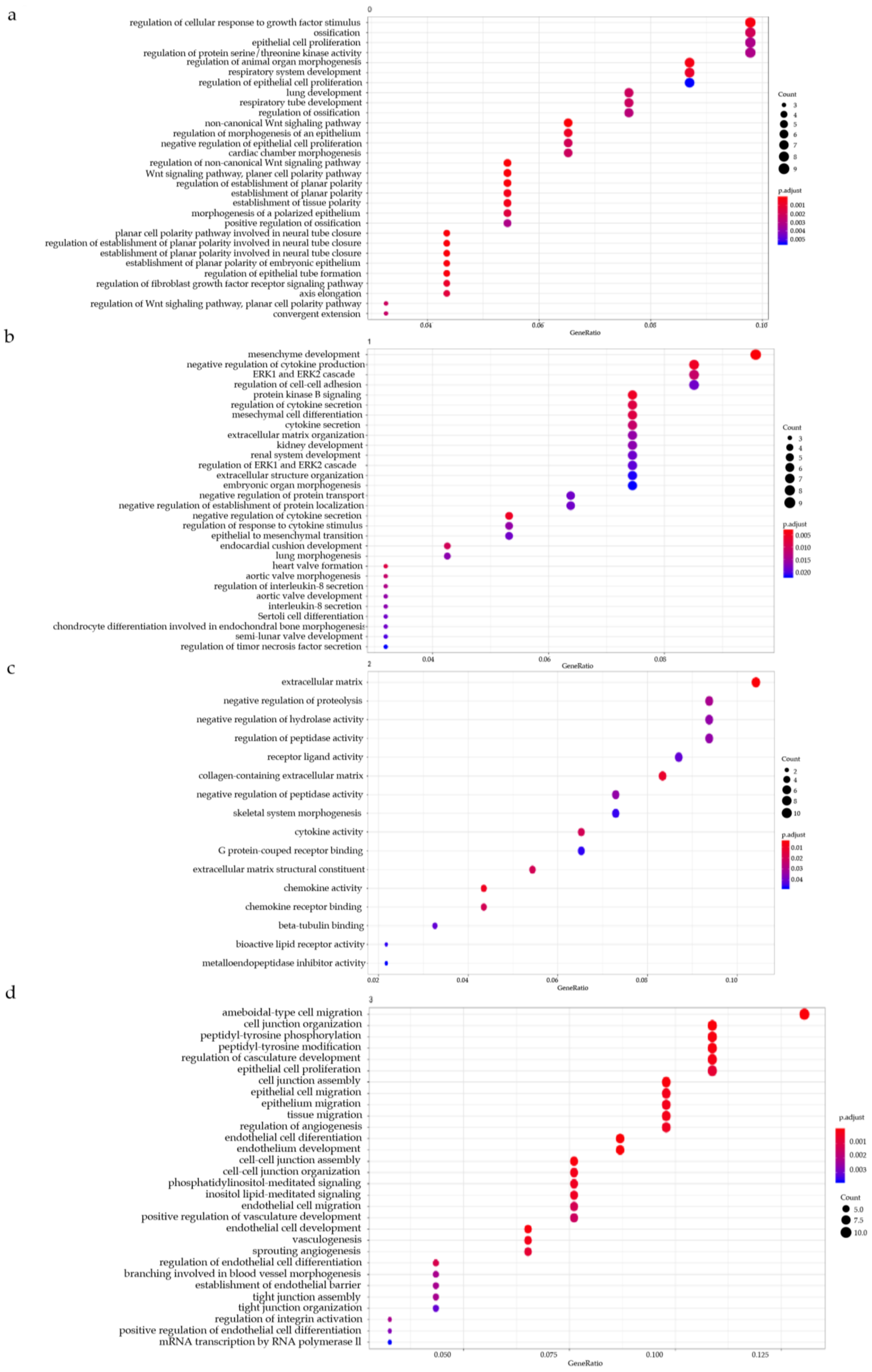
a
regulation of cellular response to growth factor stimulus
ossification
epithelial cell proliferation
regulation of protein serine/threonine kinase activity
regulation of animal organ morphogenesis
respiratory system development
regulation of epithelial cell proliferation
lung development
respiratory tube development
regulation of ossification
non-canonical Wnt sighaling pathway
regulation of morphogenesis of an epithelium
negative regulation of epithelial cell proliferation
cardiac chamber morphogenesis
regulation of non-canonical Wnt signaling pathway
Wnt signaling pathway, planer cell polarity pathway
regulation of establishment of planar polarity
establishment of planar polarity
establishment of tissue polarity
morphogenesis of a polarized epithelium
positive regulation of ossification
planar cell polarity pathway involved in neural tube closure
regulation of establishment of planar polarity involved in neural tube closure
establishment of planar polarity involved in neural tube closure
establishment of planar polarity of embryonic epithelium
regulation of epithelial tube formation
regulation of fibroblast growth factor receptor signaling pathway
axis elongation
regulation of Wnt sighaling pathway, planar cell polarity pathway
convergent extension
GeneRatio
Count
p.adjust
b
mesenchyme development
negative regulation of cytokine production
ERK1 and ERK2 cascade
regulation of cell-cell adhesion
protein kinase B signaling
regulation of cytokine secretion
mesechymal cell differentiation
cytokine secretion
extracellular matrix organization
kidney development
renal system development
regulation of ERK1 and ERK2 cascade
extracellular structure organization
embryonic organ morphogenesis
negative regulation of protein transport
negative regulation of establishment of protein localization
negative regulation of cytokine secretion
regulation of response to cytokine stimulus
epithelial to mesenchymal transition
endocardial cushion development
lung morphogenesis
heart valve formation
aortic valve morphogenesis
regulation of interleukin-8 secretion
aortic valve development
interleukin-8 secretion
Sertoli cell differentiation
chondrocyte differentiation involved in endochondral bone morphogenesis
semi-lunar valve development
regulation of timor necrosis factor secretion
GeneRatio
Count
p.adjust
c
extracellular matrix
negative regulation of proteolysis
negative regulation of hydrolase activity
regulation of peptidase activity
receptor ligand activity
collagen-containing extracellular matrix
negative regulation of peptidase activity
skeletal system morphogenesis
cytokine activity
G protein-couped receptor binding
extracellular matrix structural constituent
chemokine activity
chemokine receptor binding
beta-tubulin binding
bioactive lipid receptor activity
metalloendopeptidase inhibitor activity
GeneRatio
Count
p.adjust
d
ameboidal-type cell migration
cell junction organization
peptidyl-tyrosine phosphorylation
peptidyl-tyrosine modification
regulation of casculature development
epithelial cell proliferation
cell junction assembly
epithelial cell migration
epithelium migration
tissue migration
regulation of angiogenesis
endothelial cell diferentiation
endothelium development
cell-cell junction assembly
cell-cell junction organization
phosphatidylinositol-meditated signaling
inositol lipid-meditated signaling
endothelial cell migration
positive regulation of vasculature development
endothelial cell development
vasculogenesis
sprouting angiogenesis
regulation of endothelial cell differentiation
branching involved in blood vessel morphogenesis
establishment of endothelial barrier
tight junction assembly
tight junction organization
regulation of integrin activation
positive regulation of endothelial cell differentiation
mRNA transcription by RNA polymerase II
GeneRatio
p.adjust
Count

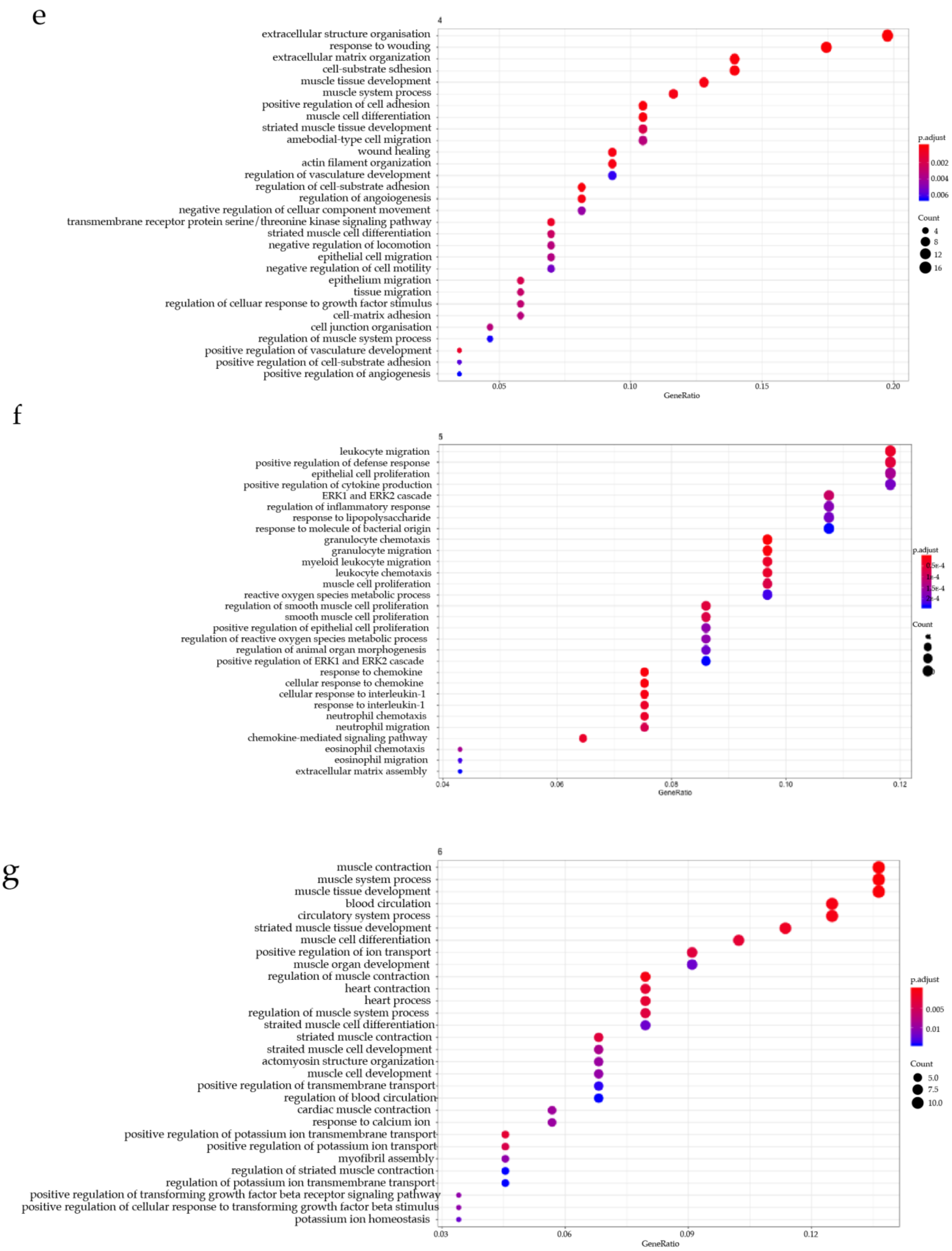
e
extracellular structure organisation
response to wouding
extracellular matrix organization
cell-substrate sdhesion
muscle tissue development
muscle system process
positive regulation of cell adhesion
muscle cell differentiation
striated muscle tissue development
amebodial-type cell migration
wound healing
actin filament organization
regulation of vasculature development
regulation of cell-substrate adhesion
regulation of angiogenesis
negative regulation of cellular component movement
transmembrane receptor protein serine/threonine kinase signaling pathway
striated muscle cell differentiation
negative regulation of locomotion
epithelial cell migration
negative regulation of cell motility
epithelium migration
tissue migration
regulation of celluar response to growth factor stimulus
cell-matrix adhesion
cell junction organisation
regulation of muscle system process
positive regulation of vasculature development
positive regulation of cell-substrate adhesion
positive regulation of angiogenesis
0.05
0.10
0.15
0.20
GeneRatio
p.adjust
0.002
0.004
0.006
Count
4
8
12
16
f
leukocyte migration
positive regulation of defense response
epithelial cell proliferation
positive regulation of cytokine production
ERK1 and ERK2 cascade
regulation of inflammatory response
response to lipopolysaccharide
response to molecule of bacterial origin
granulocyte chemotaxis
granulocyte migration
myeloid leukocyte migration
leukocyte chemotaxis
muscle cell proliferation
reactive oxygen species metabolic process
regulation of smooth muscle cell proliferation
smooth muscle cell proliferation
positive regulation of epithelial cell proliferation
regulation of reactive oxygen species metabolic process
regulation of animal organ morphogenesis
positive regulation of ERK1 and ERK2 cascade
response to chemokine
cellular response to chemokine
cellular response to interleukin-1
response to interleukin-1
neutrophil chemotaxis
neutrophil migration
chemokine-mediated signaling pathway
eosinophil chemotaxis
eosinophil migration
extracellular matrix assembly
0.04
0.06
0.08
0.10
0.12
GeneRatio
p.adjust
Count
g
muscle contraction
muscle system process
muscle tissue development
blood circulation
circulatory system process
striated muscle tissue development
muscle cell differentiation
positive regulation of ion transport
muscle organ development
regulation of muscle contraction
heart contraction
heart process
regulation of muscle system process
straited muscle cell differentiation
striated muscle contraction
straited muscle cell development
actomyosin structure organization
muscle cell development
positive regulation of transmembrane transport
regulation of blood circulation
cardiac muscle contraction
response to calcium ion
positive regulation of potassium ion transmembrane transport
positive regulation of potassium ion transport
myofibril assembly
regulation of striated muscle contraction
regulation of potassium ion transmembrane transport
positive regulation of transforming growth factor beta receptor signaling pathway
positive regulation of cellular response to transforming growth factor beta stimulus
potassium ion homeostasis
0.03
0.06
0.09
0.12
GeneRatio
p.adjust
0.005
0.01
Count
5.0
7.5
10.0

Figure S5. Bubble plot of GO term enriched analysis of cluster: (**a**) cluster 0; (**b**) cluster 1; (**c**) cluster 2; (**d**) cluster 3; (**e**) cluster 4; (**f**) cluster 5; (**g**) cluster 6. Dot size is proportional to the number of genes overlapping with each GO term and the adjusted *p*-value is colour-coded from red to blue, n = 6 mice/group.

Table S1. Differentially expressed gene HCD compared with NCD specifically of AA&R-derived cells of Apoe$^{-/-}$ mice.

DIFFERENTIAL EXPRESSION

Arch Root HCD vs Arch Root NCD

149 Genes ^ UP

2210407C18RIK	ACKR1	PERP
SREK1IP1	FOSL1	LAYN
GLIPR1	ANGPTL4	TMPPE
MMD	EMCN	RAB13
TAPT1	GBE1	EPB41
SLC35B4	FAM20A	ABCA3
PTGES3L	ZFP598	SH3BGR
FAM63A	MIF4GD	1700021K19RIK
RRAS2	LY96	MED25
TESK1	EXOC2	OTUB1
POLE3	TMEM214	TMED8
TMEM173	PELP1	NOL10
SLC30A9	CACNB3	NEK6
B3GALT1	LDLRAD4	FOXJ3
STX3	GPATCH11	TMEM53
GNE	TNFRSF1B	HSPB6
1190005I06RIK	RGS2	DNAJC30
ADSL	CEP164	SRD5A3
SLC1A4	ANO10	EIF1AD
EIF4E	TSPAN11	FABP4
PGM5	PLOD1	TLN2
SF3A1	YTHDF2	MRPL3
SDF2L1	LSMEM1	MAP2K1
COX19	RPAIN	INAFM1
RNF220	CISD1	TUG1
SPP1	IFT46	BCL10
FIGF	2210016L21RIK	RELB
GIGYF2	MVB12A	MYDGF
AURKAIP1	RGS7BP	ARL6IP5
PDLIM1	HTRA4	LOXL3
FBXO33	CCDC25	SYAP1
TLK1	RAMP1	AARS
RICTOR	ESYT1	GRPEL1
ENAH	RAB8A	GM6402
RIOK3	NDUFB3	HIGD1A
0610037L13RIK	OXR1	FUNDC2
KCNMB1	MRPS21	ZC3H15
SNRPE	XPO1	CDK11B
PA2G4	MICAL2	TPM2
CFLAR	LRP10	PRDX6
CRELD2	CNN1	ECH1
RPS7	TBCA	NPNT
RPL7	NDUFB9	TOMM7
MCFD2	NARS	ITGAV
RPSA	BC048507	AKAP2
GSTT1	MUSTN1	UQCRB
FAT1	CSNK1A1	COX7A2
SRSF2	PSMC3	2410006H16R...
PDLIM7	RPS16	ATP5O
TBRG1	GPC6	

28 Genes v DOWN

ZFP623	CHAD	SQLE
CIPC	CXCL14	LPAR6
PACS1	RARB	GM20199
DCLRE1A	PF4	LMTK2
ACY1	COL2A1	DCAF17
TMEM88	MAPKBP1	AI507597
VLDLR	HHIP	SELE
LAMC3	PJA1	TSPAN5
PLTP	RBP4	SMOC1
NR4A1		

Table S2. Differentially expressed gene HCD compared with NCD specifically of DT aorta of Apoe$^{-/-}$ mice.

DIFFERENTIAL EXPRESSION

Thoracic HCD vs Thoracic NCD

337 Genes ^ UP

TPPP3	DDX54	TMEM261
SLC25A39	MFAP3	CREB3
CD44	DARD1	PIGX
ADAMTSL5	TARS	ARRB1
SEPT6	FABP4	TMC6
FZD1	ELOF1	NUS1
SYNRG	ITIH4	OPTN
KCNH2	TCAP	KCNMA1
PRPS2	LAS1L	PHYH
SSSCA1	AARSD1	AGAP1
NUDT9	MARF1	OXSR1
CACNA1C	MORF4L2	1190005I06RIK
PAWR	SREBF1	RPRD1B
FAM210A	PRORSD1	IFT46
GM15417	MROH1	RAD23A
NCBP1	TESK1	EDEM1
LZTR1	WBSCR22	GLO1
ACP2	MRPS6	RPS6
NEK9	COL8A1	SLAIN2
MLEC	CLMP	FAT4
ABCC5	NFATC3	WFS1
MRC2	KLHL24	TBPL1
SEC61A1	DAPK2	ITSN1
HBS1L	SLC20A2	COL4A6
MAN2A2	SAMHD1	PPP1R15B
BAG5	CSNK1G2	CTCF
ARL6IP4	MMP14	ZMYM4
FTO	LYPLA1	NOMO1
COA5	UBXN7	DYNC1LI1
AHSA1	STAU1	GXYLT1
SEPT8	DPP3	1110059E24RIK
ARHGAP44	MARK3	SURF1
CAV3	SLC2A1	KDELR3
DHX29	RCAN3	DECR1
0610009L18RIK	EBP	MED14
WDFY1	TGFB3	SEH1L
ATG5	IFI35	SNAP23
MAPK6	KIFAP3	A430105I19RIK

ATXN10	RAD23B	ANO10
ABCE1	SLC25A24	SPP1
LRPPRC	GALNT16	GPAA1
FAM98A	HNRNPLL	SDHC
FBXW7	1810026J23RIK	PIK3R3
FUNDC1	SLC25A46	CERS2
MAP2K7	GNAI1	ZCCHC24
ABHD14A	PRDM5	GNAO1
TUBB6	TMEM38B	ZFP771
MRPL41	RNF181	DESI2
TMEM263	UBE2D2A	VPS4A
RHOG	SPSB1	PRKAB2
ALDH7A1	SEMA5A	ATP8B1
FAM89B	CAND1	JAK1
INF2	SEC24C	TRAPPC2L
ZFP68	FNIP1	CSNK2A1
YIPF6	GLIPR1	KANSL3
SMARCD3	MYO1E	ADCY7
PAIP1	SDHAF2	MALSU1
WDR6	ESD	EXOC6B
SOX4	CPT1A	SCFD1
HACD1	ARHGAP23	IGSF3
EFHD1	TMEM167	CRAT
POP5	GATAD2A	CUEDC1
RBPMS2	HSPB2	OSGEP
SEC61G	DYNLT3	OLFML2B
CACYBP	AGL	MAN1A2
UPF3B	B3GNT1	DDX1
CRYAB	INTS3	CTDSP2
MBTPS1	TMEM87B	DNAJC13
SNCG	ZFP651	PPP1R9B
KMT2D	DLC1	BLOC1S1
EXOC5	OSR1	NUP153
EMC8	MPND	OSBPL1A
PTK2	DTNA	PPFIA1
PTPN2	NEO1	PPIC

SLC6A6	OLFR1033	TMEM110
LSM14A	FIBIN	ANAPC4
NAT6	RAPH1	LYST
NF1	THBS2	ANAPC7
AMMECR1L	QRICH1	PRKAR2A
ARFGEF1	SCARB2	TNK2
MUSTN1	NBL1	E030013I19RIK
HSPB6	2310022B05RIK	ARL8B
FKBP10	EP300	MMP23
IRAK1	THOC7	JKAMP
ZBTB38	RERE	BICD2
DHX15	1810058I24RIK	MTPN
TMEM65	EFEMP2	PPP2R5A
AGRN	MTUS2	UNC5C
DAG1	RAN	FKBP8
FKBP3	MRPL33	FIGF
SORBS2	PDHA1	2310036O22R...
MSRB1	HSPE1	IMMT
GM15421	COLGALT1	PCM1
DNAJC5	MYOCD	ARF1
BC031181	CHCHD1	ZC3H13
S100A4	PCP4L1	NES
ALCAM	GLG1	EEF1B2
HMGN3	SEMA3C	ANP32E
BRD4	MGA	MSRB3
GUCY1B3	PPP2CA	ATP1B3
ST5	ITGAV	NDRG2
SAMD4	LRP1	HMGN1
PRNP	HNRNPC	CENPB
MYEOV2	FBXO30	ATP6V1A
PITPNA	SRP14	SYNPO
DEGS1	PPP1CB	MPRIP
GSTP1	PLEC	PI15
EIF3F	RARRES2	PDLIM7
SOST	HSBP1	ENDOD1
COL3A1	VKORC1	RPS21
ACTN1	HSPB1	UQCR10
LGALS1	COX6A1	OGN
RPL14-PS1		

111 Genes ˅ DOWN

KLK8	AHSG	BACE2
ADH1	G0S2	KANK4
1500015O10R...	IFIT3	ABCG2
FZD4	PAGR1A	FAM76B
ARL5B	RNF31	HS6ST1
RBBP5	S100B	GOLM1
RRS1	SUPT20	NDUFAF5
TMEM183A	COCH	ALOX5AP
MFNG	ZFP9	ILVBL
ACKR3	LDLRAP1	ALDH1A1
SLCO2A1	MIDN	NRIP1
MBD1	MIB1	FBLN7
ZFP869	CDK12	SIPA1L1
XIRP1	INTS6	APOD
ZFP280D	CNNM3	KLF4
GJA4	2310035C23RIK	WSB1
CERK	ATG9A	SOS1
EGFL7	TMEM9	PLCE1
ELP2	PER1	FAM160B2
ENG	NDUFB4	SRGN
SFRP1	CYTL1	ADD3
KDM6B	EPHX2	NR4A2
MAP3K3	2900060B14RIK	IFNGR1
EFEMP1	METTL7A1	BMX
NR4A1	PNRC1	EIF5
CHD2	BMP4	MAP2K3
ENTPD2	NP-904339.1	ZFP36L1
FOSB	SMAD7	KCNQ1OT1
POLR2A	ABLIM1	NUAK1
CDC42EP3	ADAMTS1	NFKBIZ
ABCA8A	ICAM1	SRSF7
KLF9	PECAM1	FAM3C
GATA6	PTPRB	MALAT1
FBLIM1	IER3	CFH
RBM39	FOS	ID3
HES1	DDX5	RPS24
UBC	RPS4X	DCN
