# Supplementary material for: Single-Cell Analysis Uncovers Osteoblast Factor Growth Differentiation Factor 10 as Mediator of Vascular Smooth Muscle Cell Phenotypic Modulation Associated with Plaque Rupture in Human Carotid Artery Disease
Source: Int J Mol Sci. 2022 Feb 4;23(3):1796. doi: 10.3390/ijms23031796 (PMC8836240; doi:10.3390/ijms23031796)
Supplement: Supplementary file 1 [file ijms-23-01796-s001.zip › Table S3. All differentially expressed genes among all 7 detected clusters.pdf]

p_val	avg_logFC	p_val_adj	cluster	gene
7.53904165908684e-38	0.948686432558543	1.04121704353648e-33	0	Serpinf1
3.02703087813591e-33	1.20	4.18063234579351e-29	0	Smoc2
1.49033217102682e-32	1.12	2.05829776140514e-28	0	Abca8a
1.70962257550768e-28	1.44	2.36115973903366e-24	0	F3
8.65323454389225e-28	0.978543581488903	1.19509822285696e-23	0	Itgbl1
1.5018306053642e-27	0.568905434097087	2.0741782490685e-23	0	Cst3
6.18559756968191e-27	0.573160587944816	8.54292880348769e-23	0	Col1a2
7.16040580555969e-25	1.33	9.88923645805849e-21	0	Svep1
1.01526513774416e-24	-1.26	1.40218268173845e-20	0	Fn1
2.52813546351657e-24	0.659759485269025	3.49160788866274e-20	0	Serping1
3.93286792466142e-24	0.647125287342419	5.43168389074988e-20	0	Ctsl
5.12768170756052e-24	1.43	7.08184120631184e-20	0	Gdf10
6.6453474771548e-23	0.904448910943556	9.17788940069849e-19	0	Mfap4
2.78538237379201e-22	0.739483551418517	3.84689159644415e-18	0	Pcolce
7.97423531906271e-22	-1.18	1.10132163991575e-17	0	Clu
1.25241601459942e-21	0.735049015271517	1.72971175776326e-17	0	Fbln1
3.18010716608493e-21	1.02	4.39204600707989e-17	0	Pdlim2
3.97256236111789e-21	1.31	5.48650587693992e-17	0	Cpxm2
4.03974302917851e-21	1.50	5.57928909759844e-17	0	Hsd11b1
9.55479525478003e-21	0.741015205601852	1.31961277263767e-16	0	Pdgfra
0.00	1.25	1.57266635179138e-16	0	Fmod
1.96150883885884e-20	0.794623590107641	2.70903985734795e-16	0	Abcc9
2.49144445674566e-20	0.667815335043508	3.44093393921143e-16	0	Igfbp4
2.79041984795978e-20	0.54	3.85384885201726e-16	0	Col1a1
3.42203142664774e-20	0.639679957411341	4.72616760334319e-16	0	Ppap2b
3.50467468124023e-20	0.91	4.84030620226088e-16	0	Cpxm1
4.07748665919897e-20	0.952739502278437	5.6314168250197e-16	0	Dpep1
1.29303905247809e-19	0.59	1.78581623537749e-15	0	Ogn
2.81496945955959e-19	0.859432861568306	3.88775432059774e-15	0	Colec12
3.33951770492811e-19	0.650673170487534	4.61220790227622e-15	0	Fmo2
4.8585323692514e-19	0.999038606431661	6.71011905517311e-15	0	Clec11a
1.38440531079684e-18	0.90	1.91200217474151e-14	0	Lama2
1.6773203809779e-18	0.492756977745771	2.31654717816858e-14	0	Sepp1
1.73453858116848e-18	0.870137658705037	2.39557123445179e-14	0	Nr2f2
7.63489775657294e-18	0.906969982153074	1.05445572916029e-13	0	Tgfbi
8.32868769401732e-18	0.501186202789802	1.15027505742073e-13	0	Dcn
2.36898162871974e-17	0.668232933724475	3.27180052742484e-13	0	Adamts2
2.8325890472428e-17	1.10	3.91208873314703e-13	0	Ndufa4l2
3.32277292753953e-17	-1.16	4.58908169022485e-13	0	Serpine2
4.74483293106772e-17	0.597104914356998	6.55308876109763e-13	0	Dpt
8.10134123352963e-17	-1.71	1.11887623776278e-12	0	Ncam1
8.77127250637106e-17	0.364887927390828	1.21140044585491e-12	0	Timp2
1.11154647859283e-16	0.784840235680463	1.53515684158455e-12	0	Entpd2
2.11519606910416e-16	0.630971378983956	2.92129729103976e-12	0	Cygb
2.51076202065463e-16	-1.67	3.46761342672611e-12	0	Tagln
3.6558717626155e-16	-1.53	5.04912449134827e-12	0	Pdlim1
5.81696647020935e-16	0.455479670062435	8.03381239200614e-12	0	Gsn
7.17789652954249e-16	-1.58	9.91339289695114e-12	0	Synpo2
7.21689148801741e-16	0.870732052874177	9.96724883410084e-12	0	Pltp
7.39655501947491e-16	0.997796614898677	1.02153821373968e-11	0	Rbp4
8.95490119358801e-16	-1.18	1.23676140384644e-11	0	Myh11
9.13319234562263e-16	0.655428069023715	1.26138519485394e-11	0	Col14a1
1.65225956849323e-15	0.403221565165966	2.28193569004599e-11	0	Cd63
1.12446960258085e-14	1.04	1.55300496812441e-10	0	Sfrp2
1.61299729645707e-14	0.719357208761395	2.22771056613687e-10	0	Nucb2

**2.30239005525905e-14**	0.727824618811614	3.17983090531827e-10	0	Akap12
**2.36626440352715e-14**	1.46	3.26804776771135e-10	0	Pex5l
**3.13362515764771e-14**	1.03	4.32784970522726e-10	0	Podn
**5.68285840782025e-14**	-0.852087851538703	7.84859574704055e-10	0	Flna
**5.95392431293756e-14**	0.42	8.22296486859807e-10	0	C1ra
**1.1546599061472e-13**	0.769870388106378	1.59470079637989e-09	0	Clec3b
**1.42881865195874e-13**	0.971104128774017	1.97334144022022e-09	0	Ntf3
**1.44804202296925e-13**	0.783573189223584	1.99989083792283e-09	0	Bicc1
**1.50462534874758e-13**	-0.947702126688808	2.07803806915529e-09	0	Tpm1
**1.72965302933743e-13**	1.19	2.38882379881793e-09	0	Pdgfrl
**1.78550131059321e-13**	0.649031786220351	2.46595586006028e-09	0	Cd302
**2.42350984149252e-13**	0.549647800804438	3.34710944208532e-09	0	Crispld2
**2.55494461209002e-13**	0.753186464070216	3.52863400375753e-09	0	Igf1
**4.56307238939547e-13**	-1.83	6.30205927699408e-09	0	Mcam
**8.83856035361262e-13**	0.777263078877313	1.22069357043744e-08	0	Olfml3
**9.45688303117415e-13**	0.401366497538366	1.30609011543546e-08	0	Aebp1
**1.07111162995823e-12**	0.483047399392857	1.47931227213532e-08	0	Tcf4
**1.49628571555288e-12**	-1.17	2.06652020175009e-08	0	Epas1
**1.59649619953133e-12**	0.417500296596018	2.20492090117272e-08	0	Prelp
**1.70896152771787e-12**	0.425730585161959	2.36024676593115e-08	0	Lum
**2.3464537641286e-12**	0.934944902327652	3.24068729363801e-08	0	Dio2
**2.85043482968281e-12**	-0.983459269304994	3.93673554327493e-08	0	Postn
**3.6451888855834e-12**	-1.18	5.03437036987923e-08	0	Sparcl1
**4.57410535827366e-12**	1.07	6.31729691031175e-08	0	Mdk
**4.8556680854653e-12**	0.662329800691227	6.70616319283613e-08	0	Hexa
**5.33240111332206e-12**	1.53	7.3645791776091e-08	0	Hhip
**6.09560893855269e-12**	-2.02	8.41864550503512e-08	0	Cnn1
**7.22327896839952e-12**	0.40	9.97607058325657e-08	0	Rcn3
**0.00**	1.04	1.02422940516822e-07	0	Tmem204
**8.02413234941918e-12**	-1.20	1.10821291877828e-07	0	Wwp2
**8.34197715684696e-12**	1.23	1.15211046513213e-07	0	Wnt5a
**9.20805310502715e-12**	0.654732151459167	1.2717242143353e-07	0	Cebpd
**9.35649828421837e-12**	0.309301146207243	1.2922259780334e-07	0	Timp3
**1.1361620292118e-11**	0.973030560525908	1.56915337854441e-07	0	Sorcs2
**1.31375102993119e-11**	0.291455493654014	1.81442154743796e-07	0	Nfib
**1.42001701135066e-11**	0.500799600548679	1.9611854943764e-07	0	C1s1
**1.55733630081702e-11**	0.375659370632103	2.15083716505839e-07	0	Serpinh1
**1.61247206674755e-11**	0.518158730538254	2.22698517138504e-07	0	Islr
**1.66139881080935e-11**	0.35	2.29455789760879e-07	0	Hsp90b1
**1.68435171723736e-11**	0.782548087309278	2.32625815667651e-07	0	Scara5
**1.78858518414653e-11**	0.560315861669211	2.47021499782477e-07	0	Abi3bp
**1.88143773021227e-11**	0.34	2.59845364919617e-07	0	Lrp1
**2.35950007275663e-11**	1.15	3.25870555048418e-07	0	Gpnmb
**2.7547869831102e-11**	0.438547767193759	3.80463630237349e-07	0	Rnase4
**2.78153412797857e-11**	-1.42	3.8415767841512e-07	0	Cbr2
**4.20169139681972e-11**	0.349255560949269	5.80295598814771e-07	0	Cfh
**4.36924359314708e-11**	-1.19	6.03436232649543e-07	0	Col18a1
**4.56126553219126e-11**	-1.59	6.29956382650935e-07	0	Pgm5
**4.83155655981267e-11**	0.599699720436588	6.67286276475727e-07	0	Tmed3
**5.15080851225914e-11**	-1.34	7.1137816362811e-07	0	Itga8
**5.24723423964606e-11**	0.655699794151996	7.24695520837517e-07	0	Mmp23
**6.13022188813328e-11**	0.57	8.46644944970087e-07	0	Loxl1
**6.56350517501366e-11**	0.545273070056423	9.06485699721137e-07	0	Plxdc2
**6.60862571707465e-11**	0.758738421096505	9.12717297785181e-07	0	Aspn
**7.9570385626347e-11**	0.392087582025282	1.09894659588548e-06	0	Tuba1a
**8.48274195139484e-11**	0.686183561191548	1.17155149090714e-06	0	Boc

**1.00744159816642e-10**	-1.63	1.39137759122764e-06	0	Tspan2
**1.07806341242473e-10**	0.536365026177494	1.48891337889979e-06	0	Cyp1b1
**1.1124076771587e-10**	0.386315667747261	1.53634624292388e-06	0	Mettl7a1
**1.11974213929813e-10**	0.606700547844863	1.54647586858465e-06	0	Gm2a
**1.19902715273018e-10**	0.285043406785377	1.65597640063565e-06	0	Pmp22
**1.2842917258286e-10**	0.40	1.77373530254187e-06	0	Mfap5
**1.52594856306247e-10**	-1.90	2.10748756044558e-06	0	Sh3bgr
**1.69508271259758e-10**	0.641460658613215	2.34107873436852e-06	0	Pcolce2
**2.13084502325643e-10**	0.395539123653319	2.94291006161945e-06	0	Pi16
**2.49046327908457e-10**	0.644467585127004	3.4395788347437e-06	0	Pdgfrb
**2.57705674912224e-10**	0.870818237259123	3.55917307621272e-06	0	Ms4a4d
**2.60963449881259e-10**	-1.93	3.60416620631007e-06	0	Ecscr
**2.81667838352808e-10**	0.838020510874898	3.89011451549063e-06	0	Lbh
**3.53210028214562e-10**	1.35	4.87818369967132e-06	0	D630003M21
**3.59841406299643e-10**	-0.930842355086839	4.96976966240437e-06	0	Pdlim3
**3.64995282536674e-10**	1.25	0.00	0	Glt8d2
**3.70578012236677e-10**	-2.04	5.11805292700075e-06	0	Tinagl1
**3.85976665456042e-10**	-1.33	5.3307237266134e-06	0	Enah
**4.90834679449474e-10**	-1.76	6.77891775787669e-06	0	Npnt
**5.28365658536959e-10**	0.798342221108697	7.29725811005394e-06	0	Col4a4
**6.26746399834948e-10**	0.755061271250469	8.65599452812046e-06	0	Fmo1
**6.50235855118001e-10**	0.468594734858688	8.98040739503471e-06	0	Ntn1
**7.88749819152594e-10**	0.396892342943088	1.08934237523165e-05	0	Il11ra1
**8.63752619376676e-10**	0.33	1.19292874262113e-05	0	Mt1
**8.67114629678505e-10**	0.278830775836894	1.19757201504898e-05	0	Sptbn1
**8.72127076944399e-10**	-1.78	1.20449470596791e-05	0	Tek
**1.09664778356359e-09**	1.56	1.51458025387968e-05	0	Gfra2
**1.12679428761827e-09**	-1.32	1.5562155906296e-05	0	Cd97
**1.20829430978955e-09**	0.514336152643474	1.66877527125035e-05	0	Vkorc1
**1.25407379713042e-09**	0.528051502557503	1.73200132121682e-05	0	Meg3
**1.46848707483231e-09**	-1.95	2.02812749905091e-05	0	Myom1
**1.51537074738256e-09**	0.647217250510793	2.09287853921006e-05	0	Gas1
**1.5486129620642e-09**	1.16	2.13878936190687e-05	0	Tmem119
**1.75389050456484e-09**	-0.651830750601069	2.4222981758545e-05	0	Utrn
**1.93910658207647e-09**	-0.991948702439684	2.67810010050581e-05	0	Acta2
**2.16412824233157e-09**	0.628705862873742	2.98887751548414e-05	0	Slit3
**2.20456944664823e-09**	-1.05	3.04473086276587e-05	0	Aoc3
**2.28355241945514e-09**	0.737465511122363	3.1538142465095e-05	0	Aldh1a1
**2.45463108342625e-09**	0.350948487382684	3.39009098931999e-05	0	Ppib
**3.6008174041998e-09**	0.619876348917895	4.97308891694034e-05	0	Atf5
**3.86259593719781e-09**	0.487829114534937	5.33463124886389e-05	0	Dnajc3
**3.9916514482811e-09**	-1.22	5.51286981522102e-05	0	Cdh13
**4.06275904237635e-09**	0.563542993297322	5.61107651342598e-05	0	Add3
**4.12143198508835e-09**	0.984916167759888	5.69210971460552e-05	0	Kcnj8
**4.19587270933259e-09**	0.539110807004194	5.79491979885925e-05	0	Fkbp7
**4.24206516859173e-09**	-1.50	5.85871620434203e-05	0	Efnb2
**4.42477449411229e-09**	1.01	6.11105605381848e-05	0	Arhgap20
**4.43290942486884e-09**	1.13	6.12229120668636e-05	0	Shisa3
**4.63378134618662e-09**	0.486072151338765	6.39971541721835e-05	0	Ssr4
**4.9812506870009e-09**	0.677147095277005	6.87960532381694e-05	0	Pkd2
**5.24239229122264e-09**	0.53	7.24026799340759e-05	0	Gpx8
**5.34647653163678e-09**	0.601223638870754	7.38401873784355e-05	0	Lpar1
**5.95974539749778e-09**	-0.668300715684147	8.23100436848419e-05	0	Pdlim7
**6.11366707132889e-09**	1.68	8.44358559221233e-05	0	AI507597
**6.45044833838285e-09**	0.272346751972493	8.90871420014055e-05	0	Anxa5
**7.19480690936187e-09**	-0.814132160114915	9.93674782251968e-05	0	Ktn1

**7.76269864637238e-09**	0.51	0.000107210631005049	0	Atp6ap2
**8.31383806097696e-09**	1.07	0.000114822417460153	0	Adamtsl2
**8.45789517764565e-09**	0.305335415875986	0.000116811990298464	0	Col3a1
**8.53757295246088e-09**	0.892784992521013	0.000117912420046437	0	Bace2
**8.78533343630196e-09**	0.368469825678334	0.000121334240088766	0	Gas6
**8.96682516659092e-09**	-1.27	0.000123840822375787	0	Bcam
**9.1952336821633e-09**	0.979407176281109	0.000126995372384357	0	Inmt
**9.9096340267241e-09**	0.733268960929139	0.000136861955543087	0	Ppm1d
**1.00796195470833e-08**	-1.34	0.000139209625564767	0	Limch1
**1.0741268193876e-08**	-0.706650378535855	0.000148347655025621	0	Lpp
**1.19324350554548e-08**	-1.02	0.000164798860550886	0	Itih4
**1.20220229479431e-08**	-0.963580156203032	0.000166036158934043	0	Cav1
**1.25182463645601e-08**	-0.973184259694637	0.00017288950054094	0	Csrp1
**1.28423059235256e-08**	0.580192911447656	0.000177365087109813	0	Runx1t1
**1.28880020441299e-08**	-1.69	0.000177996196231478	0	Hspb6
**1.3044162704278e-08**	0.993481170997198	0.000180152931108783	0	C1qtnf2
**1.34917926021558e-08**	0.309513923185901	0.000186335147628373	0	Selm
**1.48586672887756e-08**	-1.84	0.00020521305392528	0	Cd93
**1.76819866625235e-08**	-0.934433013299971	0.000244205917796112	0	Xpr1
**1.83991539489155e-08**	0.357655605558587	0.000254110715188472	0	Col6a1
**1.86489000198474e-08**	1.06	0.000257559958174113	0	Aspa
**2.00741575052513e-08**	0.375809825581602	0.000277244189305026	0	Gstm2
**2.10150377432841e-08**	1.29	0.000290238686272497	0	Angptl7
**3.0278900664264e-08**	-1.35	0.00041818189707415	0	Pcp4l1
**3.07255921141286e-08**	0.535702320367972	0.00042435115268823	0	Fcgrt
**3.16756507524734e-08**	1.03	0.00043747241254241	0	Fzd2
**3.19340663625437e-08**	-1.35	0.000441041390533091	0	Ehd4
**3.24938273860423e-08**	-1.38	0.000448772250028631	0	Lims2
**3.30452520861961e-08**	-0.579850113110261	0.000456387976562455	0	Dpysl3
**3.31750871131583e-08**	0.887011089122679	0.00045818112811983	0	Trib2
**3.55128740217385e-08**	-1.06	0.00049046830311423	0	Pkp4
**3.68675657945296e-08**	0.822802382600096	0.000509177951188248	0	Lrp6
**3.88555175644627e-08**	0.881504851997374	0.000536633553082795	0	Fbln7
**4.29324735045317e-08**	0.814577007795649	0.000592940391571087	0	Epha3
**4.34862298580609e-08**	-1.71	0.000600588320569679	0	Vwf
**4.6132743220561e-08**	0.562558452705735	0.000637139316619168	0	Leprel2
**4.7003323228044e-08**	-0.854468887202705	0.000649162897102515	0	Ppp1r12a
**4.90955275319577e-08**	0.561594897982186	0.000678058330743867	0	Prrx1
**5.35697785363855e-08**	-1.83	0.00073985221136602	0	Emb
**5.64781532703948e-08**	-1.39	0.000780019774817423	0	Srgn
**5.69951377411723e-08**	0.622885417828221	0.00078715984734333	0	Hexb
**5.80149543400901e-08**	0.502040032780116	0.000801244534390984	0	Chpf
**5.8584780673317e-08**	-0.999865307184346	0.000809114405879181	0	Mrvi1
**6.13346072385668e-08**	-0.994478820498319	0.000847092260571845	0	Mustn1
**6.27742812748056e-08**	0.89	0.00086697559868634	0	Kcne4
**6.56552169685571e-08**	-1.22	0.000906764201552742	0	Dsp
**6.68863938712972e-08**	1.09	0.000923767985756485	0	Deptor
**6.95705254104321e-08**	0.970092685778236	0.000960838526443478	0	Slc38a4
**7.40499094974638e-08**	0.612495849814001	0.00102270330006947	0	Epb4.1l2
**7.41338965949802e-08**	-1.64	0.00102386324587327	0	Nes
**7.57651833541618e-08**	0.319123261407228	0.00104639294730433	0	Sec62
**8.13215200730959e-08**	0.340410585787533	0.00112313151372953	0	Bmp4
**9.07896953261712e-08**	-1.09	0.00125389648214975	0	Inpp5a
**1.05885346671105e-07**	1.08	0.00146238252287463	0	Slc1a3
**1.10505786400443e-07**	-1.22	0.00152619541597652	0	Entpd1
**1.18396340719794e-07**	1.08	0.00163517186168108	0	5031426D15[illegible]

**1.18528519549048e-07**	0.630456788614335	0.00163699738349191	0	Igfbp5
**1.19489116794128e-07**	-1.02	0.0016502641920437	0	Cav2
**1.28776338664079e-07**	-1.12	0.00177853001328959	0	Mylk
**1.30701725286191e-07**	0.427919624403393	0.00180512152792759	0	Fkbp9
**1.33331899027852e-07**	0.758289598951928	0.00184144685747367	0	Gli1
**1.39705533611381e-07**	0.337381713544265	0.00192947312470678	0	Rarres2
**1.44926541043856e-07**	-1.31	0.0020015804583567	0	Tpm2
**1.47274726120643e-07**	-0.591481274151776	0.0020340112424522	0	Actn4
**1.6170439639735e-07**	-0.660708889103685	0.0022332994186438	0	Pdgfa
**1.71264526217686e-07**	-0.695582647728284	0.00236533437159246	0	Zyx
**1.76296153698731e-07**	0.301125202933143	0.00243482617873318	0	Fosb
**1.77475644148306e-07**	0.358795796644146	0.00245111612133225	0	Grn
**1.78096634568384e-07**	1.39	0.00245969262002396	0	Omd
**1.81892871819181e-07**	1.09	0.00251212245269471	0	Cntn6
**1.84948907864309e-07**	0.275075468261048	0.00255432936651397	0	Wfdc1
**1.92286116595471e-07**	0.366822264295442	0.00265566355630004	0	Fam198b
**1.94919603292478e-07**	0.718975898590896	0.00269203464107241	0	Dab2
**2.24882037217964e-07**	0.349341554648635	0.0031058458160173	0	Lamb2
**2.34554134463355e-07**	0.313127438545116	0.00323942715107339	0	Celf2
**2.5100544372077e-07**	0.492807640039877	0.00346663618322755	0	Scn7a
**2.67619869873316e-07**	-0.796276789197797	0.00369609802282037	0	Rab11a
**2.88673683475249e-07**	0.851386828617457	0.00398687224247667	0	G0s2
**2.99112570296978e-07**	0.465431567732884	0.00413104370837156	0	Fuca1
**3.25143019109735e-07**	0.597364562197213	0.00449055023692455	0	Ctsk
**3.44830253417477e-07**	0.280719432598625	0.00476245062994877	0	Rbms3
**3.52556230902655e-07**	-0.691689129314132	0.00486915410499657	0	Actn1
**3.67100576317582e-07**	0.944756993210765	0.00507002605952213	0	Dtx4
**3.76824259143971e-07**	0.326161611139119	0.00520431984303739	0	Ackr3
**4.01111285848517e-07**	0.283616416462959	0.00553974796885386	0	Fbln5
**4.33788975552029e-07**	0.659113379419781	0.00599105954134907	0	C7
**4.79021765880427e-07**	-0.494227819945403	0.00661576960857458	0	Tmsb4x
**5.58171036278441e-07**	0.898365113330684	0.00770890018204155	0	Vstm4
**6.18914055520324e-07**	-0.715055553707996	0.00854782202079119	0	Kif1c
**6.73111366812763e-07**	-1.43	0.00929634108705107	0	Efhd1
**6.86985038077045e-07**	0.312968555840807	0.00948795036088206	0	Slc43a3
**6.87408825048661e-07**	0.982620790546952	0.00949380328274705	0	Colec11
**7.20272183018077e-07**	1.16	0.00994767911966266	0	Lamc3
**7.26584424060271e-07**	-1.09	0.0100348574806964	0	Asap2
**7.81265575166492e-07**	-1.08	0.0107900588586244	0	Rcan2
**7.91022268044068e-07**	0.42	0.0109248085439566	0	Cfb
**8.19360618554305e-07**	-0.546885391203143	0.0113161895028535	0	Map1b
**8.56270081001328e-07**	0.436334707974773	0.0118259460887093	0	Atp1a2
**8.70061895596715e-07**	-0.840034879802548	0.0120164248400862	0	Stk38l
**8.7544135281677e-07**	0.336757466339993	0.0120907205237524	0	Mmp2
**1.03421906027933e-06**	0.708078545573693	0.0142835994415179	0	Pid1
**1.04500020302176e-06**	-1.06	0.0144324978039335	0	Speg
**1.17260222656835e-06**	0.420236925079748	0.0161948093511355	0	Twsg1
**1.2020792855104e-06**	-2.17	0.0166019170121842	0	Mmrn2
**1.23469912113093e-06**	-1.01	0.0170524295619393	0	Hbegf
**1.37067090188918e-06**	0.309403670985782	0.0189303358259914	0	Nid1
**1.45013592623565e-06**	0.700130685957676	0.0200278272772406	0	Qpct
**1.48022072981531e-06**	-2.15	0.0204433284994792	0	Egfl7
**1.51376974320943e-06**	-1.70	0.0209066739234654	0	Tie1
**1.51413673414904e-06**	0.45	0.0209117424353324	0	Ddost
**1.51542627069042e-06**	-0.764850559216914	0.0209295522245054	0	Kcnmb1
**1.59646500610047e-06**	-1.95	0.0220487781992536	0	Stab1

**1.62339983136438e-06**	-0.787126319376538	0.0224207750709735	0	Phldb2
**1.62800604209839e-06**	-0.320698063450097	0.0224843914474208	0	Vim
**1.69593512337269e-06**	-1.36	0.0234225599889003	0	Ptprb
**1.7317794017353e-06**	0.671114465560063	0.0239176053173662	0	Igfbp6
**1.75361060278634e-06**	0.336058478165391	0.0242191160350822	0	Pdia3
**1.78784418977847e-06**	-0.748080741794056	0.0246919161050305	0	Flnb
**1.95278291699851e-06**	1.03	0.0269698848666664	0	Wisp2
**1.98811320023297e-06**	-0.808008825047034	0.0274578314084176	0	Dstn
**2.0240154473705e-06**	-0.466633130285766	0.0279536773436339	0	Aplp2
**2.11595454937376e-06**	-0.822701804790141	0.029223448281401	0	Sfxn3
**2.14141382268092e-06**	0.394677510494168	0.0295750663050462	0	Pbx1
**2.14780843967342e-06**	0.363331102421946	0.0296633823603296	0	Cebpb
**2.18717740460402e-06**	0.38	0.0302071071349861	0	Ctsz
**2.21250646288582e-06**	1.03	0.0305569267589161	0	Mpg
**2.26123116339036e-06**	0.785892051696547	0.0312298635975842	0	Flt3l
**2.43771862501665e-06**	0.845644287672594	0.0336673319301049	0	Fst
**2.4571420308807e-06**	0.772609132840955	0.0339355885884934	0	Ntrk2
**2.49118156561202e-06**	-0.615476355216175	0.0344057086026676	0	Tgfbr1
**2.50294707197644e-06**	-0.812002500603307	0.0345682020110666	0	Hspb2
**2.59186192294107e-06**	-0.926686681762644	0.0357962050177392	0	Crim1
**2.6033049485424e-06**	-1.31	0.0359542446443191	0	Rgs4
**2.61950983607057e-06**	-0.521890006114694	0.0361780503459707	0	Sypl
**2.66055565112729e-06**	-1.18	0.036744934097719	0	2900026A02F
**2.69589258372064e-06**	0.750344361151981	0.0372329724737658	0	Col8a2
**2.72318203487765e-06**	-0.866390579513155	0.0376098670836952	0	Cmip
**2.79145697701475e-06**	-0.533442329158931	0.0385528123095507	0	Hspb1
**2.91205992122902e-06**	-0.436519471396339	0.040218459572094	0	Nek9
**0.00**	-1.11	0.0412334493289828	0	Susd5
**3.00811593704102e-06**	-0.844126965135679	0.0415450892064735	0	Cdc42ep3
**3.20015828438046e-06**	0.91	0.0441973860655786	0	Prss12
**3.21582153043887e-06**	0.821069724073539	0.0444137111568912	0	Sfrp4
**3.25203520211807e-06**	-0.651743187252763	0.0449138581764527	0	Btg1
**3.43179027684794e-06**	-0.678614068551395	0.0473964555135469	0	Kank1
**3.45622507017313e-06**	-0.651391578037937	0.047733924444161	0	Tgm2
**3.57013679581789e-06**	-0.76	0.0493071592870409	0	Pbxip1
**3.6299953044243e-06**	-0.749590593247228	0.050133865149404	0	Rhog
**4.07740693668704e-06**	0.339394108403063	0.0563130672025847	0	Rpn2
**4.10468710888288e-06**	-0.686804503323013	0.0566898336607814	0	Id1
**4.13591171961501e-06**	-1.19	0.057121076759603	0	Uchl1
**4.1880418245579e-06**	-0.919305606589236	0.0578410456389692	0	Ptpla
**4.54624266500626e-06**	-1.03	0.0627881574464015	0	Rcan1
**4.79247439914027e-06**	-0.410591144119959	0.0661888639265263	0	Crip2
**4.81883513535936e-06**	-1.20	0.0665529320544481	0	Cspg4
**4.82339933705725e-06**	0.319842645590037	0.0666159682440977	0	Il33
**4.90338244334548e-06**	-0.817148562133745	0.0677206149250445	0	Itga5
**4.93644661958442e-06**	-0.697965244907626	0.0681772642630805	0	Gstk1
**5.03215265249228e-06**	-0.764572691028077	0.0694990602835709	0	Gucy1a3
**5.13649760855528e-06**	-0.403785056639439	0.070940168471757	0	Eif6
**5.19354445228041e-06**	0.67	0.0717280424304447	0	Mrc2
**5.4792685010863e-06**	-0.590336797791403	0.0756741772685028	0	Coro1c
**5.6554502937629e-06**	0.291637553646146	0.0781074240071594	0	Atf4
**5.77854639849217e-06**	0.93	0.0798075043095753	0	Tspan11
**5.80457412577149e-06**	-1.99	0.08	0	Icam2
**6.51705043784689e-06**	-0.736215019719654	0.0900069835971034	0	Marveld1
**6.79410139785809e-06**	-1.20	0.0938333344058181	0	Sost
**7.12859316121104e-06**	0.910516550413663	0.0984530001494857	0	Slc22a17

**7.23462212149688e-06**	-1.56	0.0999173661199934	0	Selp
**7.25999169174386e-06**	-0.644389977751488	0.100267745254674	0	Sdhd
**7.34952891172821e-06**	0.925299157704718	0.101504343799878	0	Gria4
**7.38495255255495e-06**	-0.387377103323817	0.101993579703336	0	Trim47
**7.50617637661849e-06**	-0.389427146641548	0.103667801937478	0	Tagln2
**0.00**	0.338194448641404	0.105432105695575	0	Ctsh
**7.68923892739167e-06**	-0.623226084390234	0.106196078826206	0	Dhrs3
**7.80295024004705e-06**	-1.05	0.11	0	Cytl1
**8.64594712480789e-06**	0.859875133604798	0.119409175740722	0	Enpp3
**8.71920430911983e-06**	-0.478203327335583	0.120420930713254	0	Hes1
**1.02144773800665e-05**	-0.516186281724379	0.141072147096099	0	Hip1
**1.03027810618164e-05**	0.591742515660465	0.142291709244747	0	Kazald1
**1.07081900471266e-05**	-0.514212206757569	0.147890812740866	0	Tm4sf1
**1.17044381251207e-05**	0.495586475576504	0.161649994946042	0	Daam1
**1.17169127340382e-05**	0.620895721299361	0.161822281769801	0	Ptprd
**1.29416437699226e-05**	-1.53	0.18	0	Pecam1
**1.31845685742133e-05**	0.40	0.18	0	Adam22
**1.35635474018922e-05**	-1.92	0.187326153167534	0	Bmx
**1.41784822594923e-05**	-0.808755740850134	0.195819018485848	0	Nxn
**1.46638444392398e-05**	0.600475788961782	0.202522355550341	0	Gns
**1.50362969635323e-05**	-0.501529882892509	0.207666297363345	0	Kcnma1
**1.52524042328944e-05**	0.581985517083789	0.210650954860505	0	Herpud1
**1.54028970126331e-05**	0.594537263684968	0.212729410641476	0	Nbl1
**1.5942393231846e-05**	0.936970088834794	0.220180392925025	0	Chst2
**1.59855598421489e-05**	-1.26	0.220776566979918	0	Ldb3
**1.60501671871438e-05**	-1.31	0.221668859021643	0	Parm1
**1.63019988345459e-05**	-1.32	0.225146905903914	0	E030013I19R
**1.65703342546699e-05**	-1.03	0.228852886391246	0	Atp8b1
**1.68734381987193e-05**	-1.50	0.233039054962512	0	Gm13889
**1.69244025160208e-05**	-0.541617637560429	0.233742923148764	0	Il1r1
**1.70135269562058e-05**	-0.77	0.234973820792158	0	Smarcd3
**1.7505184224854e-05**	-0.606898615811739	0.241764099329459	0	Pdcl3
**1.97023286391581e-05**	-0.945351742011897	0.272108860835412	0	Notch1
**1.97864169412098e-05**	0.296334738581938	0.273270204375048	0	Mmp3
**2.20564540410267e-05**	-1.47	0.30	0	Fbxo32
**2.24769764939259e-05**	0.568242291311789	0.31	0	Medag
**2.32248803981978e-05**	-0.564327992970032	0.32	0	Fam71a
**2.37960310429556e-05**	0.668707291751086	0.328646984734259	0	Cacna1g
**2.53237825528611e-05**	0.733473979570368	0.349746760837565	0	Mfap2
**2.53354645673957e-05**	-0.868659619420643	0.349908101140302	0	Mob2
**2.63645920418875e-05**	-1.38	0.364121380690508	0	Rgs7bp
**2.65082112045405e-05**	-0.366222447892031	0.366104904945908	0	Cyb5r3
**2.65204433688568e-05**	0.37	0.366273843367282	0	S100a10
**2.66365131466917e-05**	-0.920696139800994	0.367876883068959	0	Ptprj
**2.71176812507574e-05**	-0.503504396067904	0.37	0	Rassf3
**2.72066510952416e-05**	0.707940281696932	0.375751058276382	0	Ank2
**2.91955999389133e-05**	-0.57	0.403220430756332	0	Vcl
**2.98773102359757e-05**	0.801868534872112	0.41	0	Abca8b
**3.01689885701904e-05**	-0.624162199771044	0.42	0	Sfrp1
**3.07705789380506e-05**	0.785532393620295	0.424972465713416	0	Igsf10
**3.12843416570333e-05**	0.515831075606791	0.432068042625287	0	Cbx5
**3.15359808563496e-05**	0.389620142966921	0.435543431607044	0	Sdf4
**3.27866654755613e-05**	-0.392411568281605	0.452816636882976	0	Pfdn1
**3.31383465318368e-05**	0.851074240289992	0.457673703951197	0	Rnf144a
**3.38789764813469e-05**	-0.534170421842087	0.467902544183882	0	Slc25a39
**3.59843729358246e-05**	-0.681399742702175	0.496980174616674	0	Uhrf1bp1l

**3.60250256122508e-05**	0.549066803395676	0.497541628730795	0	Pik3r1
**3.67441845990067e-05**	-1.35	0.507473933496882	0	Ctla2a
**3.68995286139514e-05**	-0.592872785381884	0.509619389687282	0	Agrn
**3.88192990645759e-05**	-0.452666348460013	0.536133339380858	0	Bnip2
**4.08964614923107e-05**	-0.643606092192681	0.564821029670303	0	Abl2
**4.14388342563639e-05**	-0.501706619531278	0.572311739914642	0	Map2k3
**4.17234314630768e-05**	0.792679538017593	0.576242311936553	0	Hlf
**4.18830824092573e-05**	0.764303547817119	0.578447251154253	0	Col4a3
**4.28178548879319e-05**	-0.735787963907802	0.591357393857227	0	Rnf150
**4.28865362503219e-05**	-0.709742992044346	0.592305952153196	0	Arl4a
**4.35265086449244e-05**	-0.965307380081205	0.601144610895051	0	Arl6ip5
**4.45849075795308e-05**	0.574059162962882	0.62	0	Mlec
**4.50819438489146e-05**	0.663851392976331	0.62	0	Dact1
**4.53678987607666e-05**	-0.36	0.626576049784948	0	Psmd8
**4.58833600029523e-05**	0.325338354829256	0.633695085000774	0	Tmed10
**4.64930201816446e-05**	0.855788716981328	0.642115101728694	0	Heph
**4.65580568076488e-05**	0.404097305952636	0.643013322570437	0	Itm2c
**4.78456441385169e-05**	-1.91	0.660796191197057	0	Cdh5
**4.80178551072426e-05**	0.616814858845533	0.663174596886128	0	Cacna2d3
**4.86911409706774e-05**	0.637492466896491	0.672473347946026	0	Fmnl2
**5.05994034386219e-05**	-1.91	0.698828360890807	0	Ptprz1
**5.16139860306844e-05**	-0.527837965352239	0.712840761069782	0	Atp2b4
**5.20296872721653e-05**	0.353732167254629	0.718582010915874	0	Nr3c1
**5.22427380587309e-05**	-1.36	0.721524455329132	0	Actg2
**5.30187455238497e-05**	0.491368293863049	0.732241894429888	0	Gaa
**5.3026278176475e-05**	-0.832564640554798	0.732345927895297	0	Ier5l
**5.30535903210503e-05**	0.341287145779734	0.732723135924026	0	Stat3
**5.43221890779248e-05**	-1.24	0.750243753355219	0	Tnfaip2
**5.52824437276162e-05**	0.634331291868843	0.763505830322107	0	Mxra7
**5.5971955245059e-05**	0.675838298073134	0.77	0	Thy1
**5.67220081629629e-05**	0.797951553230519	0.783387654738681	0	Rftn1
**5.80884174377025e-05**	-0.438396163810284	0.802259133232109	0	Zfp704
**5.90287331611711e-05**	-0.500052254133833	0.815245833688934	0	Fam3c
**6.30615911385776e-05**	0.668882518204708	0.870943635214896	0	Reck
**6.36542821560485e-05**	-0.491550203939648	0.879129290857186	0	0610010K14R
**6.51033840443017e-05**	-0.757402581713422	0.899142837035851	0	Perp
**6.53699044539041e-05**	-0.345022942757876	0.902823750412869	0	Adarb1
**6.58460442080776e-05**	0.593119936972311	0.91	0	Tnfsf12
**6.78335273389042e-05**	0.369029968546746	0.936848846077605	0	Thbd
**6.86812540062324e-05**	-0.455934034679284	0.948556799080076	0	Capzb
**6.95799030314519e-05**	0.374891891542713	0.960968040767382	0	Adamts5
**7.04707140766304e-05**	0.568978686907931	0.973271032112343	0	Slc29a3
**7.09675164729136e-05**	0.378230007934966	0.98	0	Frmd6
**7.21056603898155e-05**	-0.575817891217621	0.995851275643741	0	Cops6
**7.27167448067703e-05**	0.72	1.00	0	Gnptg
**7.32342845244491e-05**	0.460383005968742	1.00	0	Prdx4
**7.38628933363363e-05**	-1.27	1.00	0	Gucy1b3
**7.53644905135428e-05**	-0.470056349616899	1.00	0	Gbp2
**7.65166379356428e-05**	-0.921795127806601	1.00	0	Isyna1
**7.76430688723798e-05**	-0.951821635952526	1.00	0	Nov
**7.93768962478289e-05**	-1.03	1.00	0	Osbpl3
**8.11891156169152e-05**	-0.487539751083435	1.00	0	Uba1
**8.18783344972479e-05**	-0.659361978297578	1.00	0	Ece1
**8.25770815869159e-05**	-0.783063617442301	1.00	0	Dmpk
**8.85573946076735e-05**	-0.505683471217254	1.00	0	Filip1l
**8.89912174705257e-05**	-1.08	1.00	0	Sorbs2

**9.14800505183577e-05**	-1.50	1.00	0	Edn1
**9.24394397381538e-05**	-1.01	1.00	0	Rgs2
**9.30450912267451e-05**	-0.525981908886757	1.00	0	Fermt2
**9.34996440747248e-05**	-0.625865592291001	1.00	0	Fads3
**0.00010026016991919**	0.920167433320436	1.00	0	Ptgfr
**0.000100676578953695**	0.924414294278833	1.00	0	Tmeff2
**0.000101323050773963**	0.43	1.00	0	Pdpn
**0.000101658803554413**	-0.717360102924501	1.00	0	Sgk1
**0.000104074520732245**	0.630229188853047	1.00	0	N4bp2
**0.000108250546671912**	-1.62	1.00	0	Ramp1
**0.000110036766227316**	0.730689447888844	1.00	0	Kif1a
**0.00011015957574905**	0.469876995489511	1.00	0	Ergic3
**0.000110581236693602**	-0.743763212424915	1.00	0	Tppp3
**0.000111175854586388**	-0.440774450475999	1.00	0	Gnl3
**0.000112787574371114**	-0.558279801518893	1.00	0	Adam10
**0.000115089085831675**	-0.750338628375341	1.00	0	Sept9
**0.000116890296898588**	0.566010483981273	1.00	0	Dpp7
**0.000118668525289715**	0.779139552049135	1.00	0	Rasa3
**0.000119014723111133**	0.251756304926956	1.00	0	Fkbp2
**0.000122923662572143**	-0.320924131955002	1.00	0	Rock2
**0.000123589681973368**	-1.77	1.00	0	Nos3
**0.000125335301171158**	0.310887403761695	1.00	0	Npc2
**0.000127650208225536**	-0.659025440495966	1.00	0	Zak
**0.00012784915599489**	0.364672181028754	1.00	0	Glul
**0.000129555351275653**	-0.410867546315902	1.00	0	Cox5a
**0.000137127217903824**	-0.508820160620055	1.00	0	Csrp2
**0.000137195882111117**	-0.402915177534791	1.00	0	Gpx1
**0.00013846685378327**	-0.61	1.00	0	Ivns1abp
**0.000139433063924311**	-0.442580014544969	1.00	0	Sar1b
**0.000141163294790488**	-0.896987494582183	1.00	0	Mgat4a
**0.000142140801461062**	-0.490798517793897	1.00	0	Slk
**0.000148132782916758**	-0.479372706637716	1.00	0	Lrrfip1
**0.00014860619571083**	-0.412392340843842	1.00	0	Frzb
**0.000149394267504339**	0.439343612159043	1.00	0	Ifit2
**0.000153029034637855**	0.491586678822072	1.00	0	Dnm1
**0.000153333318577334**	0.272112852345085	1.00	0	Ski
**0.000153881080335343**	0.710631693383626	1.00	0	Atp1b1
**0.000155623886957254**	0.285103650697605	1.00	0	Ddr2
**0.000156742468303082**	0.520424125796725	1.00	0	H2-T23
**0.000157863685747637**	-0.432731643627496	1.00	0	Rnh1
**0.000158814759006373**	-0.649622571606554	1.00	0	Ly6c1
**0.000165252649690926**	-0.772498850197054	1.00	0	Ano1
**0.000165664872598072**	-0.628661379793538	1.00	0	Camk2g
**0.000165763408662365**	-0.45	1.00	0	Map1a
**0.000170258490092068**	-1.08	1.00	0	Tmem158
**0.000170997805960964**	0.740607945768999	1.00	0	Ephb3
**0.000172430891358394**	0.656285726466946	1.00	0	Lgi2
**0.000173854424683953**	-0.956295016889737	1.00	0	Adss
**0.000177831947072359**	-0.290182782350525	1.00	0	Paics
**0.000177864706589539**	0.273478448063619	1.00	0	Col6a2
**0.000178217312733243**	-1.60	1.00	0	Tes
**0.00018634972112875**	0.51	1.00	0	Tceal3
**0.000186798506834617**	-0.325733401430456	1.00	0	Dazap2
**0.000192351516808648**	-1.05	1.00	0	Stom
**0.000199853102674281**	-0.345019785508121	1.00	0	Zfand3
**0.000202866859660166**	-0.538191082570095	1.00	0	Endod1

**0.000203736966021769**	0.48	1.00	0	Ifnar2
**0.000203907814478994**	0.839193544427123	1.00	0	Shc2
**0.000206067649756685**	-0.466655793023908	1.00	0	Rock1
**0.000206351726435778**	0.372571846839787	1.00	0	Cxcl12
**0.000206571282074285**	-0.292556670705687	1.00	0	Clip3
**0.000209468023317346**	0.998362056332044	1.00	0	Sult5a1
**0.000209724227468231**	0.752752692590915	1.00	0	Aldh3a1
**0.000219548942710317**	-0.516710440069525	1.00	0	Wdr1
**0.000220166390943701**	-0.839730959758983	1.00	0	Dram2
**0.00022061742789177**	-0.74	1.00	0	Dpcd
**0.000225845883332802**	0.268568168603667	1.00	0	Phlda1
**0.000226009453173328**	-0.418442903308841	1.00	0	Azin1
**0.000227754573776579**	-0.59	1.00	0	Gja1
**0.000228668224839179**	-0.428711450899204	1.00	0	Arid5b
**0.000231650852190134**	-0.485780741386975	1.00	0	Sulf1
**0.000232098404581524**	-0.586717481266478	1.00	0	Tns1
**0.000234747777205553**	0.652407295006108	1.00	0	Abca9
**0.000235968238648872**	-1.64	1.00	0	Itgb3
**0.000242392910829125**	-0.359387537162645	1.00	0	Dnajc15
**0.000243703793318897**	-0.985167303974537	1.00	0	Smtn
**0.000244586790974881**	-0.269452098561904	1.00	0	Acly
**0.000255859819742647**	0.254023288956905	1.00	0	Tgfbr2
**0.000256250179887816**	-0.60	1.00	0	Pdlim5
**0.000262034840224271**	0.509857342191086	1.00	0	Gprasp1
**0.00026683107100846**	0.324350587625644	1.00	0	Mgst1
**0.000273213362955657**	0.258221883184783	1.00	0	Tapbp
**0.000277742167543976**	0.841027174954083	1.00	0	Zfp9
**0.00027864425605195**	-1.42	1.00	0	Ralgapa2
**0.000285070780636247**	-0.719825020598255	1.00	0	Ethe1
**0.000285447633748423**	0.255635027255607	1.00	0	Zeb2
**0.000285700263807351**	0.567606928123354	1.00	0	Erlec1
**0.000288550145737554**	0.291256085867756	1.00	0	Zfp36
**0.000292290078218309**	-1.17	1.00	0	Sorl1
**0.000303010476975291**	0.925511198226763	1.00	0	Figf
**0.000303342957325081**	-0.284882327570928	1.00	0	Cdk2ap2
**0.000310397941210039**	-0.808392899371485	1.00	0	Ap1s1
**0.000321708278498161**	-0.617629840027259	1.00	0	Rnf11
**0.00032415355282384**	-0.478931793700769	1.00	0	Ap1m1
**0.00032955953704715**	-0.67	1.00	0	Myo1e
**0.00033214042974224**	-1.25	1.00	0	Thsd4
**0.00033534641039833**	-1.12	1.00	0	Thbs1
**0.000335874290537976**	-0.323115850046797	1.00	0	S100a1
**0.000347656618560922**	0.413072248182272	1.00	0	Rab3il1
**0.000357092255582446**	-0.519729899559559	1.00	0	Rras
**0.000361351558035079**	0.315298834148924	1.00	0	Bmp1
**0.000368208436391253**	0.536755093311734	1.00	0	Btbd1
**0.000372821991997902**	-0.529537016822878	1.00	0	Atp1a1
**0.000373721508872909**	0.863355466690289	1.00	0	Smim1
**0.000378020765072459**	-0.646510821312888	1.00	0	Sbno1
**0.000382067651071967**	-0.545906485748915	1.00	0	Akap2
**0.000397786406996385**	-0.333920995827664	1.00	0	Dnaja2
**0.000402320376685721**	0.659446738467404	1.00	0	Gpr153
**0.000413761283799341**	0.411824937079774	1.00	0	Gstt1
**0.000421390509834741**	-1.16	1.00	0	Trf
**0.000421766195899214**	0.747340164102094	1.00	0	1700025G04I
**0.000423139326493928**	0.749545886916048	1.00	0	Prrg3

**0.000426145153113482**	-0.95	1.00	0	Myl9
**0.000428438857195285**	-0.51	1.00	0	Psmc6
**0.000440675137155193**	-0.880812519962264	1.00	0	Cxcl2
**0.000446570917604927**	0.834179205542495	1.00	0	Tnfsf13b
**0.000455050868712637**	-0.950249752642407	1.00	0	F11r
**0.000456534792378308**	-1.51	1.00	0	Calcrl
**0.000462832308494369**	0.474854755316107	1.00	0	Adamts10
**0.000464858685670869**	-0.675586529060139	1.00	0	Cd151
**0.000466402830588903**	-0.37	1.00	0	Tmem176b
**0.000476718925660859**	-0.498236141393813	1.00	0	Vcam1
**0.000478947292201543**	-0.861279282300544	1.00	0	Rmnd5a
**0.00051050944715288**	-1.20	1.00	0	Procr
**0.000511500590706967**	0.546166087876881	1.00	0	Lipa
**0.000515505926167288**	-0.750817703085937	1.00	0	Trnt1
**0.000526868844520274**	1.04	1.00	0	Npr3
**0.000530423460184009**	-0.341544023190555	1.00	0	Cand1
**0.000531168074256279**	-0.555136595449595	1.00	0	Pxdn
**0.000536380316366782**	-0.509289153787521	1.00	0	Rab8b
**0.000541665699930547**	-0.565383344215361	1.00	0	Eapp
**0.000542466807286163**	-0.937201837452469	1.00	0	Arhgef5
**0.000545169625461702**	-0.383476711427396	1.00	0	F2r
**0.000548698107804948**	0.258698321885334	1.00	0	Lpl
**0.000551026629568274**	0.53	1.00	0	Elmsan1
**0.000551809751169637**	-1.12	1.00	0	Trabd2b
**0.000567938262612424**	0.644824600576499	1.00	0	5430435G22I
**0.000572471531552652**	-1.62	1.00	0	Sgcg
**0.000574709772519531**	-0.858856217539724	1.00	0	Lama5
**0.000578768697270227**	-1.09	1.00	0	Ppp1r12b
**0.000581857050318817**	-0.539326163413762	1.00	0	Sh3bgrl
**0.000584053957294214**	-1.84	1.00	0	Esam
**0.000589869099512079**	-0.360353317451531	1.00	0	Hk2
**0.000609770650695211**	0.378859733489232	1.00	0	Lrpap1
**0.000615364804778144**	1.10	1.00	0	Plekha4
**0.000615625526496805**	-0.343174475478115	1.00	0	Cct7
**0.00061758677390453**	-0.366849926037012	1.00	0	Flnc
**0.000628908537543618**	-0.630193739683493	1.00	0	Npr1
**0.000632708528080607**	-0.502392271024046	1.00	0	Klf2
**0.00063442834263066**	0.405032672216698	1.00	0	Sec63
**0.000637870604457109**	1.13	1.00	0	Vipr2
**0.000639005876566138**	0.497999738837272	1.00	0	Prss23
**0.000639633131290186**	0.499268354136804	1.00	0	Camk2n1
**0.000641133532246057**	0.683577008425744	1.00	0	Hoxa2
**0.000646766627577113**	0.653038454153227	1.00	0	Cdk14
**0.000663808598441215**	-0.776010890988232	1.00	0	Itga7
**0.000665394074943863**	-0.866020954337089	1.00	0	Plin2
**0.000700007796349324**	0.412789378386267	1.00	0	Foxp2
**0.000702067654708244**	-0.423894399239909	1.00	0	Atl3
**0.000705495519322198**	-0.902123745066943	1.00	0	Unc5c
**0.000713387506916209**	0.309409270690118	1.00	0	Ier3ip1
**0.000714682228210265**	-0.569766013328173	1.00	0	Skap2
**0.000721582720118176**	-0.926280005739792	1.00	0	Phtf2
**0.00072447703482447**	0.653832650135629	1.00	0	H6pd
**0.000726666358598031**	-0.545260273500422	1.00	0	Nap1l4
**0.000732758500956241**	-0.53	1.00	0	Ict1
**0.000744488139628265**	-0.358155267895479	1.00	0	Gm12338
**0.000751630696692859**	0.668684269770849	1.00	0	Uap1l1

**0.000767890314618891**	-0.470237296615151	1.00	0	4833439L19R
**0.000773948622537096**	-0.362542898260834	1.00	0	Iqgap1
**0.000781525954999574**	0.694439005697314	1.00	0	Lrig3
**0.000786803136696772**	-0.318566178236071	1.00	0	Cox17
**0.000806265320973801**	-0.817153530381672	1.00	0	Lmod1
**0.00080664238062771**	-0.252407210018054	1.00	0	Calm1
**0.000814604201793993**	0.307395041375271	1.00	0	Olfml2b
**0.000816546797364251**	-0.274190921171932	1.00	0	Stra13
**0.000820087988011748**	-0.582545443098538	1.00	0	Hk1
**0.000831454302500769**	-0.81623038678957 8	1.00	0	Gnptab
**0.000841404186267296**	-0.54	1.00	0	Sugt1
**0.000843837577123699**	-0.322511929347933	1.00	0	Upf2
**0.000847109321351505**	0.398065007270485	1.00	0	Stk17b
**0.000858179464569046**	-0.33	1.00	0	Eif3i
**0.000863874176844364**	-0.451236680646999	1.00	0	Emc8
**0.000874086041617453**	0.516790757822408	1.00	0	Ripk1
**0.000880744020537145**	-0.507489721442693	1.00	0	Rnf20
**0.000895884813514558**	-0.455231077686287	1.00	0	Cct6a
**0.000911136686054844**	0.48	1.00	0	Cdh11
**0.000912319309166584**	-0.35	1.00	0	Stk24
**0.000912790541460882**	-0.569521962760806	1.00	0	Lrrc8a
**0.000917027605564495**	-0.903610893016433	1.00	0	Polr1c
**0.000930084741985556**	0.63	1.00	0	Adamtsl5
**0.000944780410776298**	-0.74	1.00	0	Zfp664
**0.000985269422265203**	-0.39	1.00	0	Ebna1bp2
**0.000987206310621143**	-0.293279516675244	1.00	0	Dhx9
**0.0009909199097887 1**	-0.637534747743501	1.00	0	Me1
**0.00102445821402015**	-0.381626982621565	1.00	0	Efna5
**0.00103440633509486**	-0.396673955499532	1.00	0	Nus1
**0.00105449337984745**	0.497267639764534	1.00	0	Rbms1
**0.00105867592935796**	0.33	1.00	0	Manf
**0.00106931051029447**	0.598692819744137	1.00	0	Pcnp
**0.00106953840343448**	0.457803133719793	1.00	0	Creb3l1
**0.00107904727687684**	0.460089093600824	1.00	0	Mmp14
**0.00108485944980676**	-0.294486575719798	1.00	0	Ssu72
**0.00109504876127133**	0.527908823371411	1.00	0	Hyou1
**0.00111703458580108**	-0.586679248010785	1.00	0	1110008P14R
**0.00113240173285839**	-0.424048458262675	1.00	0	Ndufa4
**0.00113266663749459**	-0.51	1.00	0	Mob4
**0.00113924663150554**	-0.426007079799144	1.00	0	Sh3glb2
**0.0011478662033613**	-1.35	1.00	0	Ccl2
**0.00115430552096513**	0.947815723246543	1.00	0	Mn1
**0.0011571351336638**	0.801047404403092	1.00	0	Hic1
**0.00116487644488918**	-0.66	1.00	0	Fam63b
**0.00116760079626576**	0.300173854371094	1.00	0	Lima1
**0.00117640358526878**	-0.45	1.00	0	Emp2
**0.0011828231810272**	-0.59	1.00	0	Spon1
**0.00120728986913086**	-0.433662604938368	1.00	0	Cacna2d1
**0.00121314590447028**	-0.416224457217074	1.00	0	Nmt2
**0.00121429224844806**	-0.317062238301357	1.00	0	Ube2j1
**0.00122214325414263**	-0.42	1.00	0	Tcp1
**0.00122932328370996**	0.461742722746604	1.00	0	Cnpy2
**0.00123408280523787**	0.569101935555433	1.00	0	Igsf3
**0.0012395405318632**	0.300484198072113	1.00	0	Alg11
**0.00125940876429687**	-0.698812918095623	1.00	0	Ppp2r2a
**0.00128762495586688**	-0.605722858201181	1.00	0	Fam219b

**0.00130360067001917**	-0.318485394266694	1.00	0	Ypel5
**0.00131428100455186**	0.454551838182199	1.00	0	Atraid
**0.00131899224292989**	0.831102899449662	1.00	0	Spry1
**0.00132118570983022**	-0.513599180049137	1.00	0	Hoxa5
**0.00132626919024556**	-0.377281989773163	1.00	0	Pkm
**0.00139094949564893**	-0.361162186067664	1.00	0	Lama4
**0.00141220365770486**	-0.594384362133345	1.00	0	Gid4
**0.00142275232630871**	-0.702220570477765	1.00	0	Cited2
**0.00143878799119659**	-0.464773709300587	1.00	0	Actr3
**0.00144156303387568**	0.724512513491784	1.00	0	Plekhf1
**0.00146245756135373**	-1.11	1.00	0	5930403L14F
**0.00147359053753429**	-0.394120971705485	1.00	0	Tmem30a
**0.00148395562685043**	0.720733102364514	1.00	0	Cpeb1
**0.00149595717865991**	0.794047274082442	1.00	0	Fancc
**0.00150309155698069**	-0.677812790109025	1.00	0	Mndal
**0.00151872766206241**	-1.66	1.00	0	Car8
**0.00153225039290945**	-1.62	1.00	0	Rbpms2
**0.00153575899615099**	0.709783370657992	1.00	0	Gpc3
**0.00154119337783608**	-1.61	1.00	0	Net1
**0.00154187034024101**	0.586280679851439	1.00	0	C2
**0.00155285015496275**	-0.317071397529967	1.00	0	Zc3h14
**0.0015548536764976**	-1.49	1.00	0	Adh7
**0.00156115525908339**	-0.699056749472969	1.00	0	Ppa1
**0.00160522618998708**	0.645720048499622	1.00	0	Tssc1
**0.0016129990386399**	0.664171078628135	1.00	0	Ednra
**0.00161798799072534**	-0.370440174626395	1.00	0	Cyr61
**0.00163224569935277**	-0.99	1.00	0	Arhgef7
**0.00164344610544401**	-0.468177458741308	1.00	0	Dcaf13
**0.001644988591989**	-0.461703763394051	1.00	0	Pa2g4
**0.00166448114527949**	-0.483053509292487	1.00	0	Fxyd5
**0.00168815843051495**	0.254142551101807	1.00	0	Nenf
**0.00170431268535809**	-0.368651887234681	1.00	0	Tmf1
**0.00172248255416048**	-0.584738188318698	1.00	0	Arhgdib
**0.00173580683306268**	0.580603925623332	1.00	0	Uchl4
**0.00174922599222322**	-0.433523440247437	1.00	0	Ugdh
**0.00174941914312854**	0.621828453359904	1.00	0	Foxs1
**0.00176525364946358**	-0.366608108953184	1.00	0	Nfkbib
**0.00177554673411812**	0.593283514897545	1.00	0	Usp37
**0.00178180043942569**	0.971669634886566	1.00	0	Lsamp
**0.00180767983438159**	-0.282167149760828	1.00	0	Pacsin2
**0.00181185915923734**	0.33	1.00	0	Tspan31
**0.00181988831062224**	0.693534072660566	1.00	0	Plekhh2
**0.00182117034943138**	-0.904230615710371	1.00	0	Acsl1
**0.0018516850622816**	0.536860944220828	1.00	0	Sardh
**0.0018571467305743**	-0.391835403110141	1.00	0	Polr2a
**0.00188038972587536**	0.469784319325516	1.00	0	Tgfb3
**0.00189048385014748**	0.422843185641208	1.00	0	Srpx
**0.00191301833277083**	-0.256065983795224	1.00	0	Srsf1
**0.00191467132451033**	-0.646355815000685	1.00	0	Ehbp1l1
**0.00193806319690849**	-0.581851155362864	1.00	0	Mtus1
**0.00194075375184831**	-0.44	1.00	0	Parp14
**0.00195320006442578**	0.54	1.00	0	Mmp16
**0.0019566595712455**	-0.697341427541116	1.00	0	Ivd
**0.00196728686171582**	-1.93	1.00	0	1190005I06R
**0.00197701525517529**	-0.31	1.00	0	Lxn
**0.00197813657066743**	0.750383903631859	1.00	0	Ptges

**0.00197921453015716**	-0.753406227555761	1.00	0	Drg1
**0.00198424755474478**	-0.807209160273435	1.00	0	Pla2g16
**0.00199197188655663**	0.63	1.00	0	Fnbp1
**0.00199437595364629**	-0.675664512194911	1.00	0	Ptgs1
**0.00200495425686149**	-0.382587395418979	1.00	0	Col4a2
**0.00200908362428703**	-0.358714357690978	1.00	0	Kpna1
**0.00204677874736782**	0.324529631631719	1.00	0	Maged2
**0.00206269500149053**	-0.547990211056174	1.00	0	Arpc1a
**0.00207456505013921**	-0.471852163130384	1.00	0	Cald1
**0.00211821769058625**	-0.61	1.00	0	Bcl7b
**0.00212712098186744**	0.344421901103533	1.00	0	Rnf145
**0.00214403049521784**	-0.422992196218273	1.00	0	Dnajb1
**0.00214908041492701**	0.315847733867905	1.00	0	Cd44
**0.00215121618412493**	-0.414728059817842	1.00	0	Cpe
**0.00218080115746257**	-0.299723601907971	1.00	0	Itgb1
**0.00219924586724256**	0.269015363798262	1.00	0	Sec61a1
**0.00220562496772326**	-0.50	1.00	0	Ext2
**0.00222039737363305**	-0.443570937129518	1.00	0	Prkacb
**0.00222116674796881**	-0.557932780042906	1.00	0	1110008F13F
**0.00222398465605719**	-0.430434307032277	1.00	0	Syngr2
**0.00223421478662655**	-0.561784711703996	1.00	0	Gng11
**0.00226476634482829**	-0.515149919038437	1.00	0	Mrpl54
**0.00227090175694675**	-0.515848289232471	1.00	0	Elovl5
**0.00228170836883689**	-0.680973739858849	1.00	0	Aqp1
**0.00232493430800797**	-0.645624007013117	1.00	0	4931406P16F
**0.0024028380858613**	-0.265212208328984	1.00	0	Pop5
**0.00241518198939328**	0.495859035141144	1.00	0	Tmem218
**0.00241575302247905**	-0.297030574391682	1.00	0	Cox6a1
**0.00242724934729681**	0.841456769469007	1.00	0	Tshz2
**0.00244968518158657**	-0.75	1.00	0	Tmem38b
**0.00246624508775044**	-0.403603610173034	1.00	0	Scoc
**0.00250226607245567**	-0.295568296120639	1.00	0	Prps2
**0.00254538235146231**	-0.638752018867447	1.00	0	1110058L19F
**0.00260493609202612**	0.414398540500617	1.00	0	Mettl9
**0.00260948590208135**	-0.370322725174949	1.00	0	Plin4
**0.00262436179176565**	0.578568525762074	1.00	0	Athl1
**0.00265874023879261**	-0.609602762880221	1.00	0	Mknk2
**0.00266667382641542**	0.675523053439529	1.00	0	Adcy7
**0.00267904432896771**	0.538273309463069	1.00	0	Ptprk
**0.00269778262223266**	-0.836237499283535	1.00	0	Tspan9
**0.00270351752092262**	-1.11	1.00	0	Serpini1
**0.00271393930865013**	-0.410735354240625	1.00	0	Cab39
**0.00272208865406455**	0.486255809535813	1.00	0	Ikbip
**0.00277370404188688**	-0.406762743442046	1.00	0	Ddx42
**0.00278104487659413**	-0.278554855293625	1.00	0	Macf1
**0.00278277266449068**	-0.365550642452189	1.00	0	Gltscr2
**0.00279841416273639**	-1.20	1.00	0	Sncg
**0.00280001856848882**	-1.60	1.00	0	Gja4
**0.00284369998536659**	-0.471513550502574	1.00	0	Lamtor3
**0.00284428969079831**	-0.620908753697099	1.00	0	Alcam
**0.00285431595162971**	0.849166573303521	1.00	0	Ift88
**0.00286238070404754**	0.293393109825436	1.00	0	Nav1
**0.00286518850876461**	-0.308345079379292	1.00	0	Ptgis
**0.00286924223025285**	0.372914170961829	1.00	0	Sec61b
**0.00287821804807479**	-0.357150591970693	1.00	0	Hif1a
**0.00289512332247093**	0.265138199808253	1.00	0	Arhgef10

**0.00299115151014835**	-0.30	1.00	0	Capza2
**0.00299566570164931**	-0.333888180976146	1.00	0	Zfhx3
**0.00300922558606042**	-0.429568948326153	1.00	0	Peli1
**0.00301145859090986**	-0.408142813540366	1.00	0	Git2
**0.00310708358188888**	0.441823535230449	1.00	0	Ccbe1
**0.00312038979681718**	-0.272812719258766	1.00	0	Chsy1
**0.00313845013596221**	-0.989929181855517	1.00	0	Lmo2
**0.00314171565629435**	-1.36	1.00	0	Cd24a
**0.00315325244027778**	-0.546219688500142	1.00	0	Slc11a2
**0.00316958267445567**	-0.524371458507378	1.00	0	2510039O18I
**0.00317237039936143**	-0.488332876800228	1.00	0	Msrb1
**0.00317257567676242**	0.650171535939456	1.00	0	Lrrk1
**0.00319371919770359**	-0.359886936010512	1.00	0	2010111I01R
**0.00322373880852589**	-0.730571399781895	1.00	0	Myo9b
**0.0032242596726511**	-0.499086419695469	1.00	0	Ankrd46
**0.00323716651503931**	-0.453772757077561	1.00	0	Atxn7
**0.00328999148588309**	-0.641640548398051	1.00	0	Tbl1x
**0.00336792352595907**	-0.351904983695439	1.00	0	Dusp1
**0.0034004623857945**	-0.453933228175198	1.00	0	Eftud2
**0.00341978781773448**	-0.308890709870451	1.00	0	2210013O21I
**0.00345135876269951**	-0.968257768907573	1.00	0	Tmem2
**0.00345424563182996**	-0.447710375057322	1.00	0	Lbr
**0.00352839918338302**	-1.47	1.00	0	Palmd
**0.00353450848432007**	0.590906886797672	1.00	0	Vwc2
**0.00356499900498571**	-0.592298768651917	1.00	0	Atp6v0a2
**0.00359502788452483**	-0.339489166827263	1.00	0	Insig2
**0.00360623211276448**	1.07	1.00	0	Cpz
**0.00361711077218855**	-0.585694811355422	1.00	0	Slmap
**0.00363180936204467**	-0.674080763402792	1.00	0	Dtna
**0.00365487148076417**	-0.677480887116481	1.00	0	Creb5
**0.00365841513805763**	-0.525629898085835	1.00	0	Bag6
**0.00371188078881718**	-0.331405130744456	1.00	0	4930503E24F
**0.00373085463783491**	0.56	1.00	0	Ptgir
**0.00374331690801868**	-0.354182397287315	1.00	0	Slc39a14
**0.0037477699317479**	-0.803272011239891	1.00	0	Rtfdc1
**0.00376455207395338**	-0.460576313958902	1.00	0	Pgls
**0.00378024051113002**	0.886312395677361	1.00	0	Plcxd3
**0.00379540437343775**	-0.567757004829454	1.00	0	Phf5a
**0.00380519655750272**	-0.449521981681174	1.00	0	Mgll
**0.00382212418495167**	-0.362509450825345	1.00	0	Usp22
**0.00383617944062044**	-0.929844835930594	1.00	0	Prdm16
**0.00388241254598628**	0.960422133317933	1.00	0	Fam174b
**0.00389468638264689**	-0.555186379053267	1.00	0	Pqlc1
**0.0039423448126321**	-1.42	1.00	0	S1pr1
**0.00396557754350934**	-0.294815650180789	1.00	0	Usf2
**0.00401204888069172**	-0.372718442444102	1.00	0	Myo1c
**0.00401236077645877**	-0.268584585402904	1.00	0	Tmx4
**0.00412791970394413**	-1.53	1.00	0	Amigo2
**0.0041449379610231**	0.590624275171521	1.00	0	Mmp19
**0.00416825688356269**	0.462204936759917	1.00	0	Adamts15
**0.00417543353278526**	-0.456463917519206	1.00	0	Tmbim1
**0.00419500697580248**	0.400511367754577	1.00	0	Capn1
**0.00427672616343054**	-1.28	1.00	0	Ttll7
**0.00428512862535202**	-1.18	1.00	0	Crispld1
**0.00432721973971135**	-0.397135216669406	1.00	0	Ddx24
**0.00435585784720823**	0.315101105159461	1.00	0	Osr1

**0.00436265390187983**	-0.787177746826628	1.00	0	Daxx
**0.00437719840895952**	-0.455639228971655	1.00	0	Fbxo30
**0.0043989828805284**	-0.441084042805058	1.00	0	Sema3f
**0.00441944287058951**	0.51	1.00	0	Mpp6
**0.00443072446675103**	-0.375065280415964	1.00	0	Dmd
**0.00444182573963547**	0.518649974773433	1.00	0	Dapk2
**0.00444328826561844**	-0.346589978247143	1.00	0	Gtf2i
**0.00446819042129361**	-0.792411600384774	1.00	0	Trappc11
**0.00448333572065981**	-0.486909608997584	1.00	0	Atp6v1a
**0.00448644633046711**	0.302816456974516	1.00	0	Cryab
**0.0045241359754354**	-0.514571430264105	1.00	0	Idnk
**0.00460703402872952**	-0.551983589570475	1.00	0	Usp15
**0.00462986866955595**	-0.264288885346515	1.00	0	Fkbp1a
**0.00468657626792938**	0.435302408480678	1.00	0	Dtx3
**0.00473446331156688**	0.419139932216308	1.00	0	Steap4
**0.00476856179263023**	-0.473112670168933	1.00	0	Uqcrc2
**0.00478457021381012**	-0.503277265357899	1.00	0	Vcan
**0.00480318407055803**	-1.01	1.00	0	Fli1
**0.00480925737994613**	-0.618147875849097	1.00	0	Ccdc85b
**0.00482438209098681**	0.530495190968709	1.00	0	Fgf7
**0.00484456430598364**	-0.386904700670448	1.00	0	Sorbs1
**0.0048550774634244**	-0.475503534590453	1.00	0	1110007C09I
**0.00486516593765868**	-0.59	1.00	0	Msi2
**0.00489263327987305**	0.445774125660328	1.00	0	Derl1
**0.00492154841067382**	0.750233209665216	1.00	0	Letm1
**0.00506204132553092**	-0.742130120784978	1.00	0	Pth1r
**0.00510176753078864**	-0.486650011619296	1.00	0	Snhg12
**0.00513113429499001**	0.406255830836172	1.00	0	Ssh2
**0.00514530419772998**	-1.14	1.00	0	Spint2
**0.00515227002914346**	0.61	1.00	0	Mapk8
**0.00516233810888971**	0.503877633160485	1.00	0	Csgalnact2
**0.00524219167361911**	-0.333452401233498	1.00	0	Srsf7
**0.00529782477550618**	0.270296114275864	1.00	0	Pla2g15
**0.00530020100647295**	0.432734703269197	1.00	0	Creg1
**0.00533088604253829**	-0.544457342365635	1.00	0	Itga9
**0.00533498077845244**	-0.542443735811052	1.00	0	Ulk2
**0.00536416512387998**	0.387178864468797	1.00	0	Fam102b
**0.00541109627994418**	0.414866255504188	1.00	0	Gabra3
**0.00543025900260273**	0.484014311824095	1.00	0	Vgll3
**0.0054380866453358**	-0.388433540893045	1.00	0	Rnpep
**0.00546302882520667**	-0.598282334557263	1.00	0	Zfp467
**0.00548245063469227**	0.599458208152165	1.00	0	Gba
**0.0054900023407109**	-0.999200492358861	1.00	0	Tnc
**0.00553522285421577**	-0.348129651348982	1.00	0	Psmd4
**0.0055373734614849**	-0.259773290602968	1.00	0	Arpc2
**0.00554793606960343**	0.419663430574029	1.00	0	Khsrp
**0.00556847292108516**	-0.52	1.00	0	Fcf1
**0.00558476560822741**	0.293727364311896	1.00	0	Sccpdh
**0.00559113118112749**	-0.504665964001103	1.00	0	Stk16
**0.00561135923894088**	-0.620729539188963	1.00	0	Inhba
**0.00589255400745559**	-0.458766183015344	1.00	0	Ppp3ca
**0.00591192821299834**	-0.489334558049505	1.00	0	Myc
**0.0059727223539595**	0.28	1.00	0	Arhgef25
**0.00602716478340072**	-0.72	1.00	0	Fam214b
**0.0060432923730897**	-0.382607025033795	1.00	0	Aco2
**0.00614329927347724**	0.562717160717336	1.00	0	Naalad2

**0.00621706593442193**	-0.313074691613704	1.00	0	Ugp2
**0.00623062475872726**	0.307270926170373	1.00	0	Smdt1
**0.00626407159338865**	-0.467836083974677	1.00	0	Rbpms
**0.00628153374204771**	0.518229326783208	1.00	0	Kirrel
**0.00630185149920956**	-0.260269858894548	1.00	0	Tmem33
**0.00639035948513432**	0.543931601175614	1.00	0	Farp1
**0.0064263610068074**	-0.362290632753307	1.00	0	Itfg3
**0.00648366283289899**	0.486617657429802	1.00	0	Uchl3
**0.00648751136526742**	-0.511355472832484	1.00	0	Gltp
**0.00648949346965368**	-0.477748487379842	1.00	0	Zbtb41
**0.00650638732757328**	0.278234574975778	1.00	0	Col6a3
**0.0065405074287733**	-0.259352731476941	1.00	0	Aip
**0.00657149091524453**	-0.367142342909256	1.00	0	Cs
**0.00657345183350094**	-0.668030540338705	1.00	0	Mef2c
**0.00658029978180525**	0.373170145291811	1.00	0	Ubtd2
**0.00662085854312097**	-0.402621047661544	1.00	0	Zranb2
**0.01**	-0.584316801834461	1.00	0	Setd8
**0.00670433818906913**	-0.302635467150957	1.00	0	Ext1
**0.006731634298361**	-0.59	1.00	0	Cyld
**0.00674786989573319**	-0.411419165873788	1.00	0	Mfge8
**0.00675616028222152**	0.314726137569797	1.00	0	Tmtc1
**0.00682220264429084**	-0.404467262464647	1.00	0	Sun2
**0.00683411179985017**	-0.833157065118716	1.00	0	Tnk2
**0.0069015191185003**	-0.338487870363192	1.00	0	Bzw1
**0.00695008898969766**	-0.773169283362784	1.00	0	Pik3r3
**0.00695896963587885**	-0.781414656567246	1.00	0	Nhsl2
**0.0069839654454605**	-0.68	1.00	0	Ugcg
**0.00705556291409141**	0.338038093347474	1.00	0	Unc119b
**0.0070564610542483**	-0.490825117416134	1.00	0	Gnl3l
**0.00711433744026477**	-0.357107558209216	1.00	0	Sgms2
**0.00712528460031178**	0.411536111412461	1.00	0	Xbp1
**0.00713528738879867**	-0.491383671910333	1.00	0	Adipor2
**0.00718655954606429**	-0.41	1.00	0	Kars
**0.00723394081635886**	-0.268730921837279	1.00	0	Clpp
**0.00731538706236375**	-0.321454897822966	1.00	0	Zcrb1
**0.00739211083731214**	-0.35	1.00	0	Ube2h
**0.00740936708875724**	-0.262287275704612	1.00	0	Tnfrsf12a
**0.0074124709467038**	-0.33	1.00	0	Plekha3
**0.0074220677599028**	-0.867039136162127	1.00	0	Ggta1
**0.00744508515161956**	0.363939502615906	1.00	0	Ppp1r9b
**0.0074949352352405**	-0.364666519056068	1.00	0	Ssfa2
**0.00759277785082914**	-0.681701600478864	1.00	0	Smap2
**0.00764242097685648**	-0.598413955468978	1.00	0	Max
**0.00764465781451282**	-0.510378735865058	1.00	0	Pdlim4
**0.00770757066246054**	-0.332110055252513	1.00	0	Ndufa8
**0.00772833381433073**	-0.768806416448376	1.00	0	Sort1
**0.00773601216339102**	-0.391478641367476	1.00	0	Snrpd2
**0.00788156271597631**	-0.377766883535214	1.00	0	Med10
**0.00790619043331038**	-0.65	1.00	0	Cdc37l1
**0.00802685365979195**	0.839247506361947	1.00	0	Galnt10
**0.00803873184168365**	-0.85	1.00	0	Cdk17
**0.00817122665174227**	-0.477704046462447	1.00	0	Serpine1
**0.00824536580551958**	-0.33	1.00	0	Fbxo3
**0.00826879605514093**	-0.519604228138158	1.00	0	Nek7
**0.00827900883659762**	-0.262009704197266	1.00	0	Ndufb7
**0.00839284039167505**	-0.357636653484376	1.00	0	Pcdh7

**0.0083952068930555**	0.420733678367483	1.00	0	Adprh
**0.00839576141440629**	0.757680521625255	1.00	0	Aff2
**0.00839758542726563**	-0.269816396561694	1.00	0	Ccdc59
**0.00843433661118836**	-0.354043294145679	1.00	0	Ash1l
**0.00843727122959191**	-0.66	1.00	0	Ppp1r14a
**0.00846452351537942**	0.551357642190889	1.00	0	Man1c1
**0.00846715683634218**	0.410039579889458	1.00	0	Slc4a1ap
**0.00859532758592162**	0.515306549904251	1.00	0	Col16a1
**0.00864002801535919**	-0.291429738430784	1.00	0	Mien1
**0.00868720217049983**	0.541325985382036	1.00	0	Gprasp2
**0.00869760972363078**	-0.279963381524016	1.00	0	Mcmbp
**0.0087346422284031**	-0.347274378984251	1.00	0	Wbp1
**0.00892164789701131**	-0.602465183673135	1.00	0	Plcb4
**0.00896690229755899**	0.544269889814221	1.00	0	Hmg20a
**0.00896784279413368**	-0.335650372786897	1.00	0	Smurf2
**0.00898354675924389**	0.699665570473618	1.00	0	Angptl1
**0.00917526679244811**	0.436274356838914	1.00	0	Tiam1
**0.00919403839566801**	0.660429541527037	1.00	0	Nsg1
**0.00924399176128277**	-0.479523117868704	1.00	0	Adcy5
**0.00929323775459945**	-0.559900752952119	1.00	0	0610012G03I
**0.00929493410435409**	-0.793493167391214	1.00	0	Arhgap31
**0.00929960933886155**	0.724448435573013	1.00	0	Snai1
**0.00930532076303289**	-0.424051224282053	1.00	0	Sirt1
**0.00936571851506413**	0.333982751958828	1.00	0	Tmem167
**0.00948459584722348**	-1.57	1.00	0	Gja5
**0.00948611130677851**	-0.340803485543459	1.00	0	Timm17a
**0.00951696358791903**	0.391867677886972	1.00	0	Itm2a
**0.00954268000105605**	-0.521270987404184	1.00	0	Tcof1
**0.00955073575172653**	-0.293312574526487	1.00	0	Bmpr2
**0.00957930213527533**	-0.417827824850847	1.00	0	Lats2
**0.00958985340971612**	0.406465855536424	1.00	0	Pacs2
**0.00969321500239496**	-0.313836708155318	1.00	0	Cd2ap
**0.00971442567894786**	-0.596480582690406	1.00	0	Nuak1
**0.00973594471054006**	-0.723624821307218	1.00	0	Sdpr
**0.00980088328248488**	-0.355538707479331	1.00	0	Mrpl34
**0.00980858303386556**	-0.775147089639854	1.00	0	Fam126b
**0.00982141018293688**	-0.268045130797017	1.00	0	Rnf187
**0.00988296839524271**	-0.552412568457989	1.00	0	Rnf123
**0.00997902055244648**	0.495048611329063	1.00	0	Ptgr1
**0.00998601849118786**	0.33	1.00	0	Ostm1
**0.00999871217456201**	0.644824600576499	1.00	0	Twist2

p_val	avg_logFC	p_val_adj	cluster	gene
1.0587593609494e-23	1.306757141	1.46225255340722e-19	1	Ifi27l2a
1.85415221372658e-23	-1.373113137	2.56076962237777e-19	1	Myh11
1.7437901834397e-18	-1.563770961	2.40834862234857e-14	1	Myl9
2.7789339326123e-17	0.678532308082521	3.83798565433085e-13	1	Fbln1
3.28230013383596e-17	0.560967605854793	4.53318471484084e-13	1	Lum
1.1140429590991e-16	-2.08943997	1.53860473081177e-12	1	Pgm5
1.19554769966375e-16	0.600265904646242	1.65117092800561e-12	1	Ccdc80
2.28415739610791e-16	0.982212205924457	3.15464977976464e-12	1	Fxyd6
4.24275260592743e-16	-1.535992495	5.85966562404637e-12	1	Col18a1
3.90840809911852e-15	-1.280648159	5.3979024256926e-11	1	Acta2
6.40058027362119e-15	-1.627310037	8.83984141589822e-11	1	Smtn
8.37642618700733e-15	1.194744551	1.15686822068758e-10	1	Gata4
1.14815960835269e-14	-1.427634914	1.5857232350959e-10	1	Cdh13
1.57204105010884e-14	1.784034201	2.17114589430532e-10	1	Meox1
1.73529066571184e-14	-0.964376283528586	2.39660993841463e-10	1	Smoc1
5.0609226611792e-14	1.060157189	6.98964028735459e-10	1	Tbx20
1.06293722502104e-13	0.847831987472733	1.46802260147656e-09	1	Gpm6b
1.11714487685275e-13	-0.746424380772212	1.54288878942133e-09	1	Cpe
2.10998106898652e-13	-0.839937611698111	2.91409485437729e-09	1	Eln
2.90802166895017e-13	-1.195401983	4.01626872698708e-09	1	Plcb4
2.90873801019021e-13	-1.516417461	4.0172580658737e-09	1	Mylk
7.26268488828329e-13	0.446219633480976	1.0030494099208e-08	1	Anxa2
1.19459099541229e-12	-1.012487773	1.64984962376392e-08	1	Fblim1
1.83510677631257e-12	-1.055220028	2.53446596876529e-08	1	Itga9
3.07089013270508e-12	-1.07072297	4.24120636227899e-08	1	Pbxip1
4.2444624326052e-12	-1.587180636	5.86202706567104e-08	1	Tpm2
4.44936506279641e-12	0.685015392243335	6.14501808822812e-08	1	Prg4
5.27994273779696e-12	0.37401046	7.29212891517138e-08	1	Pmp22
6.18613267595749e-12	-1.581150049	8.54366783876488e-08	1	Tagln
7.8582032297904e-12	-1.702550379	1.08529644806635e-07	1	Pecam1
9.60529846568109e-12	-1.146992296	1.32658777109522e-07	1	Dmd
1.03753398082289e-11	-1.02758315	1.4329381809145e-07	1	Anxa3
1.1033945160661e-11	0.461470059776602	1.52389816613889e-07	1	Cd34
1.15127024888573e-11	-0.754800892958174	1.59001934073609e-07	1	Cald1
1.2291065964942e-11	-1.45715373	1.69751912041814e-07	1	Itga8
1.26740402534505e-11	-2.217669101	1.75041169940404e-07	1	Vwf
1.3862101958933e-11	-0.808327557808041	1.91449490154823e-07	1	Flna
2.03618653955148e-11	0.409602334615812	2.81217722977455e-07	1	Lrp1
2.37914570755396e-11	0.561168602294283	3.28583813670277e-07	1	Olfml3
2.5096096548432e-11	-0.998177156887674	3.46602189430394e-07	1	Cav1
2.56647987111515e-11	-0.886530952086577	3.54456534999713e-07	1	Ppp1r12a
3.06820318913443e-11	-1.221910737	4.23749542451356e-07	1	Nox4
3.10819574179781e-11	0.769390401009204	4.29272913899695e-07	1	Sox9
3.61351120994491e-11	0.389377274964023	4.99062033205492e-07	1	Mxra8
3.61364791705778e-11	0.454617521207316	4.99080913824851e-07	1	Mmp2
3.62061197932387e-11	-2.260734569	5.0004272046442e-07	1	Mmrn2
3.78441889029086e-11	-2.040853712	5.22666092938071e-07	1	Tinagl1
5.68090502564036e-11	-0.763579981711009	7.8458979309119e-07	1	Map1b
6.06344205024411e-11	0.382462200327137	8.37421981559214e-07	1	Emp1
6.4926010833837e-11	-0.923918066613149	8.96693135626122e-07	1	Ccdc3
7.67369106379975e-11	-1.612056572	1.05981347282138e-06	1	Adh1
1.31857367326264e-10	0.333654303629015	1.82108210014303e-06	1	Rps18
1.50776290562901e-10	1.045131078	2.08237134896423e-06	1	Epha7

**1.87590265413668e-10**	-1.089730618	2.59080915562817e-06	1	Limch1
**3.00955064914818e-10**	-1.965612407	4.15649040153855e-06	1	Cbr2
**3.27994733632057e-10**	0.871442151282576	4.52993526619235e-06	1	Tcf21
**3.80983182188881e-10**	0.432347915180121	5.26175872921063e-06	1	Shisa5
**4.33707656090892e-10**	-1.733733551	5.98993643827132e-06	1	Myom1
**4.7678440586622e-10**	-1.653751845	6.58486942941837e-06	1	Sost
**5.14997943005692e-10**	-1.839405261	7.11263659085162e-06	1	Cnn1
**7.00526303118913e-10**	0.418246128172167	9.6749687723753e-06	1	BC048507
**1.12196000250746e-09**	-1.167402943	1.54953895946306e-05	1	Dmpk
**1.21483184102773e-09**	0.354572505202371	1.6778042556434e-05	1	Dcn
**1.23028145189296e-09**	-1.42005803	1.69914171320936e-05	1	Mcam
**1.35075287143641e-09**	-1.631207267	1.86552479074083e-05	1	Rcan2
**1.38894871229314e-09**	0.282147145012102	1.91827706654806e-05	1	Tmsb10
**1.41118616391894e-09**	-1.426465361	1.94898921098845e-05	1	Npnt
**1.44737615866048e-09**	-1.212799985	1.99897121272599e-05	1	Pkp4
**1.6533486195921e-09**	-2.188129937	2.28343977851864e-05	1	Ramp1
**1.96070697218969e-09**	0.801256256542982	2.70793239929118e-05	1	Bok
**2.14517217925646e-09**	0.483282512699479	2.9626972967711e-05	1	Serpina3n
**2.52053918576557e-09**	0.694024444476399	3.48111666946083e-05	1	Enc1
**2.59677203568056e-09**	-1.769473356	3.58640185847842e-05	1	Actg2
**3.15602051650027e-09**	-0.858692262197121	4.35877993533853e-05	1	Tns1
**3.34345620259238e-09**	-1.197123637	4.61764736140034e-05	1	Sdpr
**4.03046979628249e-09**	-0.871767245017265	5.56648183564574e-05	1	Tgm2
**4.36656360901318e-09**	-1.977187887	6.0306610004081e-05	1	Sh3bgr
**4.43802142736356e-09**	-1.744938403	6.12935139333181e-05	1	Esam
**4.76206488741811e-09**	0.268159522788766	6.57688781601316e-05	1	Ifitm7
**4.76311099778612e-09**	-2.154297863	6.57833259904241e-05	1	Egfl7
**4.9712097701851e-09**	0.588398462358146	6.86573781360264e-05	1	Vcan
**5.46286171623649e-09**	0.653840082278024	7.54475831629422e-05	1	Olfml2b
**6.29277211871448e-09**	-0.609512893872926	8.69094757315657e-05	1	Fn1
**6.36703208818556e-09**	0.341657802678561	8.79350801699307e-05	1	Gm12060
**6.48761351230296e-09**	0.862290380262258	8.96004302184162e-05	1	Matn2
**7.10505165871285e-09**	0.355899319890273	9.81278684584831e-05	1	Gsn
**8.02548525123903e-09**	0.831060313418166	0.000110839976804862	1	Ppp1r14b
**8.21059879202271e-09**	0.381434732582834	0.000113396579916626	1	Dpt
**9.73046904031518e-09**	-0.799511213858873	0.000134387507915793	1	Utrn
**1.03370840768509e-08**	0.830938384998126	0.000142765468185388	1	Dram1
**1.0617493737817e-08**	0.836659598069964	0.000146638206012991	1	Srd5a3
**1.07732199751467e-08**	-1.270893881	0.000148788941076751	1	Cgnl1
**1.38764338570083e-08**	-0.696809933128598	0.000191647427999142	1	Fmo2
**1.51717903086449e-08**	0.278020408076071	0.000209537595952695	1	Ltbp3
**1.62959718491119e-08**	-0.760803531638202	0.000225063667208085	1	Tln1
**1.84129999834373e-08**	0.312151683459183	0.000254301942771252	1	Col3a1
**2.01506467912745e-08**	0.334032005236342	0.000278300582834292	1	Rpl7
**2.48342600141897e-08**	-1.543205278	0.000342985965055974	1	Susd5
**2.57250891107123e-08**	0.437011361098681	0.000355289205708047	1	Mrc2
**2.90104118846937e-08**	-1.019162759	0.000400662798539504	1	Rbp4
**2.92385065779207e-08**	-0.928982523	0.000403813014347662	1	Lrrfip1
**3.06260182249073e-08**	-1.345814831	0.000422975937704194	1	Aoc3
**3.34685608300364e-08**	0.501203492261856	0.000462234293623632	1	Frzb
**3.74723034985892e-08**	0.475563609612666	0.000517529983619015	1	Mgst1
**3.78356306505497e-08**	0.291788609988812	0.000522547894914741	1	Cox6c
**3.98916028492051e-08**	-0.308330095896448	0.000550942926950371	1	NP-904338.1
**4.10693193815717e-08**	-2.043985448	0.000567208369978886	1	Edn1
**4.37369689811867e-08**	-1.132712384	0.000604051278599917	1	Lims2
**4.4661093467013e-08**	-0.790149060360382	0.000616814361872917	1	Pcdh7

**4.55148584778956e-08**	0.688653815519475	0.000628605710438217	1	St3gal4
**4.95814099436005e-08**	0.482240192487474	0.000684768852731066	1	Igf1
**5.57102085500469e-08**	0.256826334998229	0.000769413690284697	1	Lhfp
**5.76356054684721e-08**	0.524929525065863	0.000796005347125068	1	Sdf2l1
**5.96351998762334e-08**	-1.302121794	0.00082362174549066	1	Nov
**8.1168041588758e-08**	0.831579778679405	0.00112101182238234	1	Palm2
**8.12343415015959e-08**	0.418032440525568	0.00112192749047854	1	Anxa1
**8.52618960977541e-08**	-0.786619092117191	0.00117755204700608	1	Actn1
**9.42399951956568e-08**	0.352839144497498	0.00130154857364722	1	Cyb5
**9.81217622694529e-08**	-1.305904799	0.00135515965870341	1	Mrvi1
**1.00372010198952e-07**	0.629446849	0.00138623783285772	1	Psd3
**1.21169636631766e-07**	0.270991198840433	0.00167347385152132	1	Dynll1
**1.2612057880205e-07**	-0.482333317409096	0.00174185131383511	1	Htra1
**1.30563099918528e-07**	-1.130890526	0.00180320697297479	1	Lmod1
**1.36310815305829e-07**	0.426058840819551	0.00188258867018881	1	Arpc5
**1.4954387347103e-07**	1.161314551	0.00206535043650839	1	Htra4
**1.53754217556479e-07**	-1.749058478	0.00212349949867253	1	Cd93
**1.53918427552043e-07**	-0.428048123789636	0.00212576740292127	1	Aebp1
**1.57304759774105e-07**	-0.647768749190985	0.00217253603724016	1	Ptprd
**1.60209588739774e-07**	0.255341947808917	0.00221265463008502	1	Cd9
**1.82051319296788e-07**	0.751702214447683	0.00251431077080794	1	Ssc5d
**2.02706495593189e-07**	0.969504519234766	0.00279957941063753	1	Lhfpl2
**2.14665074328073e-07**	0.444350312989799	0.00296473934154502	1	Dkk3
**2.19277441021748e-07**	0.464799502788134	0.00302844073795136	1	Pkd2
**2.24602884098092e-07**	0.380069923981516	0.00310199043227875	1	Gem
**2.40014574033711e-07**	-0.750558121355011	0.00331484128197958	1	Zyx
**2.60657688524796e-07**	0.356803622318927	0.00359994333621596	1	Bhlhe40
**2.61888568178559e-07**	-1.447146998	0.00361694301511408	1	Entpd1
**2.81691906982684e-07**	0.431762459470837	0.00389044692733785	1	Gpx8
**2.96885587654826e-07**	-0.892134543917474	0.00410028685110081	1	Pdgfa
**3.06487674341707e-07**	0.348789475344705	0.00423290127033332	1	Cd302
**3.0852374464232e-07**	0.509817377022542	0.00426102143725509	1	Htra3
**3.08962185211886e-07**	0.299251809889992	0.00426707673996135	1	Wwtr1
**3.20618118590627e-07**	-1.131276882	0.00442805683585514	1	Tmem47
**3.29533449432478e-07**	-0.646834400104763	0.00455118647011195	1	Prss23
**3.76663938399703e-07**	-1.185960171	0.0052021056532383	1	Synpo2
**3.81703206761948e-07**	0.366659724	0.00527170298858927	1	Snx18
**4.1966983147057e-07**	0.305982263069763	0.00579606004244004	1	Col6a1
**4.20466445575801e-07**	0.451765116	0.00580706207984739	1	Emilin1
**4.6623088214172e-07**	0.300131335976279	0.00643911471325929	1	Gm6251
**4.71315655512546e-07**	-1.090959617	0.00650934051828378	1	Bmp4
**4.71437525711791e-07**	0.733738897496706	0.00651102366760555	1	Chic2
**4.72014648866608e-07**	0.361694312536747	0.00651899431549673	1	Ndufa4l2
**5.29238978428882e-07**	0.683789282791045	0.00730931953108128	1	Tmem45a
**5.30734687745384e-07**	-1.868700286	0.0073299767724515	1	Icam2
**5.33638129877988e-07**	-1.045503792	0.00737007621174489	1	Itih4
**5.42064219698162e-07**	0.514845492030922	0.00748644893825132	1	Gnpnat1
**5.59715095838611e-07**	-1.032326659	0.00773022518862706	1	Rassf3
**5.67661273817409e-07**	0.721628693304318	0.00783996985269223	1	Sdc3
**5.76519396198884e-07**	-0.923869111664439	0.00796230938090279	1	Crim1
**5.85607356432789e-07**	0.454882436048297	0.00808782319969324	1	Abi3bp
**5.92098548401932e-07**	0.326176196583091	0.00817747305197908	1	Sdc2
**5.93826641050052e-07**	0.352845135	0.00820133973954227	1	Fuca1
**6.82824820580911e-07**	0.263434875936351	0.00943049359704297	1	Ifitm3
**6.86344464425051e-07**	-1.825833081	0.00947910339817438	1	Nos3
**6.97642756643511e-07**	0.295281157250229	0.00963514411200352	1	Tgfbr2

**7.46354431458193e-07**	0.332184947401663	0.0103079010528691	1	S100a10
**7.54458270894053e-07**	0.502649064118681	0.0104198231793178	1	Ado
**7.55607357242738e-07**	-0.453649575340755	0.0104356932108795	1	Ogn
**7.79049345319992e-07**	-0.535517623166213	0.0107594505082144	1	Cyr61
**8.05148238565677e-07**	0.623700933775977	0.0111199023228306	1	A4galt
**8.25867956111266e-07**	-0.868772013109698	0.0114060623418527	1	Vldlr
**8.48436061808417e-07**	-1.770401673	0.011717750449636	1	S1pr1
**8.8214194821845e-07**	0.732365911	0.012183262446845	1	Tmem176a
**8.91977345525993e-07**	0.586072515	0.0123190991190595	1	Snai1
**8.97614519511367e-07**	0.916477383194477	0.0123969541289715	1	2610002M06
**9.36572211404941e-07**	-0.895368760697268	0.0129349988117136	1	Lmcd1
**9.79836457949743e-07**	0.294230533552961	0.0135325213207439	1	Crip1
**1.23137066004181e-06**	0.507464469796308	0.0170064601858375	1	Gas1
**1.23668782198446e-06**	0.387038362877952	0.0170798955094274	1	Atp5l
**1.24116355482112e-06**	0.356935258921329	0.0171417098556345	1	Rpl35
**1.27292392781192e-06**	0.659737698334199	0.0175803523670104	1	Gprc5b
**1.2980741468432e-06**	0.631396778	0.0179277020420515	1	Rpl12
**1.32389704913814e-06**	1.080179485	0.0182843421456468	1	Fam49a
**1.35094681483421e-06**	-0.850889866058659	0.0186579264596753	1	Nexn
**1.35420560633619e-06**	-0.577672111	0.0187029336291091	1	Ctsh
**1.39109587191678e-06**	0.283857668374566	0.0192124250870426	1	C3
**1.42982009340118e-06**	0.411845449076284	0.0197472453099637	1	Mtch1
**1.484982897581E-06**	0.696777868045495	0.0205090987984912	1	Etv1
**1.64589620508509e-06**	-1.23438157	0.0227314724884302	1	Srgn
**1.65813181312891e-06**	0.269130351044087	0.0229004584711233	1	Tram1
**1.6716019692452e-06**	0.754109113738201	0.0230864947972454	1	Itih5
**1.68703071809739e-06**	-0.947901106148435	0.023299581247643	1	Ncam1
**1.72341970759961e-06**	0.323027885597142	0.0238021495816582	1	Col5a2
**1.74301616741847e-06**	0.399510749306049	0.0240727962882165	1	Gda
**1.8607169297205e-06**	0.443859861943978	0.0256983615163698	1	Timp1
**1.90378505313301e-06**	-0.714128200900522	0.026293175	1	Cspg4
**1.91122522328191e-06**	0.678932507725484	0.0263959315587465	1	Pdpn
**1.9539397301244e-06**	-1.936768419	0.0269858616127481	1	Cdh5
**2.04657418262871e-06**	0.379567939439995	0.0282652360362851	1	Rhoq
**2.11089456918853e-06**	0.630743410557813	0.0291535648950628	1	Sema6d
**2.19615655973599e-06**	0.762543504026824	0.0303311182465138	1	Eps8
**2.20423505925417e-06**	-1.007286428	0.0304426904033594	1	Dsp
**2.22160118561546e-06**	0.338693857692104	0.0306825339745351	1	Uba52
**2.22694493911569e-06**	0.402926439075125	0.0307563365541269	1	1810009A15F
**2.23573652961817e-06**	0.406847685977456	0.0308777572105566	1	Dbi
**2.47281317890168e-06**	-0.493295404991391	0.0341520228138112	1	Clu
**2.62814149235781e-06**	1.146402628	0.0362972621509537	1	Gm17455
**2.69258481630642e-06**	-1.09713221	0.037187288898008	1	Sfrp2
**2.83483330387277e-06**	0.694666276	0.0391518827597868	1	Anxa4
**2.90577307581457e-06**	0.257245422114905	0.0401316319500751	1	Selk
**3.03268426076189e-06**	0.559148739102706	0.0418844023253825	1	Tomm20
**3.12400915767245e-06**	0.432135240332834	0.0431456904766142	1	Psmb3
**3.14317257075471e-06**	-0.934145070980346	0.0434103563746933	1	Tspan2
**3.26097588791833e-06**	0.376945331065558	0.0450373379880401	1	Kdelr2
**3.38372138285214e-06**	0.407534676017085	0.0467325760185709	1	Smdt1
**3.39972764097976e-06**	0.292750059495379	0.0469536384495715	1	Tgif1
**3.55635985798302e-06**	0.729368354441976	0.0491168859986034	1	Sept8
**3.56477840290632e-06**	0.306069772026935	0.0492331545225392	1	Rap1a
**3.91538287915315e-06**	0.585432801462738	0.0540753529439841	1	Ube2l6
**3.91890350071881e-06**	-1.95699501	0.0541239762484275	1	Bmx
**3.92243623234661e-06**	0.587761471	0.054172766804939	1	Robo1

**4.29626215548033e-06**	0.32751349	0.0593356766293389	1	Rhoj
**4.52674824215581e-06**	0.435806934523191	0.0625189199724139	1	4930503E24R
**4.54587935557506e-06**	-1.489321656	0.0627831397798472	1	Rasl12
**4.72964268293742e-06**	0.502979264049876	0.0653210950940487	1	Sox6
**4.78546523375461e-06**	0.737253150808073	0.066092060343385	1	Supt4a
**5.19462734645439e-06**	0.254477748957486	0.0717429982818816	1	Eno1b
**5.37134768566264e-06**	-0.598414882854656	0.0741836828866867	1	Fam71a
**5.75342755493444e-06**	0.271121362	0.0794605879611996	1	Slc43a3
**5.7727976359726e-06**	-0.663694677329065	0.0797281081504176	1	Lpp
**5.93592016021343e-06**	-1.540408636	0.0819809933327077	1	Tes
**5.93745773813979e-06**	-0.706392719485645	0.0820022288214486	1	Cnn2
**6.20136551274978e-06**	0.651756325005342	0.0856470590965873	1	Lsm2
**6.30883753452909e-06**	0.569679134	0.0871313551893813	1	Rtn4rl1
**6.4492413048806e-06**	0.457251215987698	0.0890704716617059	1	Gm15421
**6.5926036728381e-06**	-1.214504632	0.091050449325567	1	Bcam
**6.64177703040149e-06**	-1.614848605	0.0917295825668749	1	Jph2
**6.68098336736904e-06**	0.783485455999572	0.0922710612867338	1	Smim3
**6.8940555427682e-06**	-1.2807546	0.0952138011011717	1	Ptprb
**6.94841299760031e-06**	0.391454003837076	0.0959645319098579	1	Atp6v1f
**6.98103864496707e-06**	0.294054163747639	0.0964151247256403	1	Prelp
**7.06305607943156e-06**	0.611849834062892	0.0975478675130293	1	Man2a2
**7.43627399187242e-06**	0.392574921518724	0.10270238	1	Anxa7
**7.49351610781656e-06**	-0.551731283330074	0.103492950965055	1	Actn4
**8.20038337631743e-06**	-1.489035425	0.113255495	1	Thsd4
**8.33467226986239e-06**	0.482608854224309	0.115110159	1	Pdgfrl
**8.43184996690085e-06**	-1.132264025	0.116452279892868	1	Hspb6
**8.60211783277435e-06**	0.554974883120421	0.118803849388447	1	Rnft1
**8.64870334386088e-06**	0.771624414783907	0.119447241882063	1	Gm3258
**8.6545801062289e-06**	0.316678802518852	0.119528405847127	1	Psenen
**8.71377156775348e-06**	0.335238890476209	0.120345899122243	1	Snrpg
**8.78578081833555e-06**	0.504184479723521	0.121340418882032	1	Taf10
**8.97015693980611e-06**	-1.908135089	0.123886837495662	1	Stab1
**9.2624663570809e-06**	0.404947556436274	0.127923922857644	1	Wls
**9.46087901153578e-06**	0.310124684203705	0.130664200028321	1	Btf3
**9.58742714240077e-06**	0.339938062672424	0.132411956263697	1	Snx7
**9.65439343631338e-06**	0.455540986132234	0.133336827748924	1	Cmtm3
**9.88881921500979e-06**	-0.476234495923497	0.136574482	1	Ptrf
**1.02768986547652e-05**	0.353519371739923	0.141934247320962	1	Aldoart1
**1.03071933004702e-05**	-0.959081577481814	0.142352646672794	1	Fbxo30
**1.04514556971472e-05**	0.39825438	0.144345054633299	1	Pigs
**1.04874415742921e-05**	-0.378618642343371	0.144842055582548	1	Dst
**1.0733025239064e-05**	-1.047683072	0.148233811576713	1	Serpinb6b
**1.081044458577E-05**	-0.375754381938386	0.149303050174069	1	Pam
**1.1068141323435e-05**	0.678395105240006	0.152862099817961	1	Tlr2
**1.10728675917298e-05**	0.678533991717895	0.152927374309381	1	Steap3
**1.14479182657294e-05**	-1.712004868	0.158107199167988	1	Myct1
**1.19294185504633e-05**	0.513518092402434	0.164757199600448	1	Ankrd50
**1.21760117223369e-05**	0.313418271159334	0.168162897897195	1	Gm4788
**1.22811137559352e-05**	0.431679653	0.169614462	1	Shisa4
**1.23040254046252e-05**	0.460827569265343	0.169930894863279	1	Zfp385a
**1.25264449421091e-05**	-0.781029766	0.173002731095468	1	Ehd4
**1.3265857644621e-05**	0.381391939574791	0.18321476	1	Immp1l
**1.33225028274765e-05**	-0.788025818894713	0.183997086550277	1	Wfdc1
**1.39392656217711e-05**	0.722369222300809	0.192515198	1	Myo7a
**1.43355335154017e-05**	0.569710581774174	0.197988053381212	1	Icosl
**1.45295070172324e-05**	0.612800246053617	0.200667021414997	1	Layn

**1.46091192705779e-05**	0.349367270917514	0.201766546245951	1	Adam19
**1.52302614052925e-05**	0.281327825282474	0.210345140268495	1	Rnase4
**1.52382022189636e-05**	0.556592546605767	0.210454810846106	1	Sgce
**1.5432871820361e-05**	-0.482560659220511	0.213143392711006	1	Ltbp1
**1.6125393472199e-05**	0.88971216	0.222707809244541	1	Srpx2
**1.62070765216135e-05**	-1.546411797	0.223835933840005	1	Tmem158
**1.62782587872737e-05**	0.339945740976072	0.224819032111037	1	Fbln2
**1.65464652123334e-05**	0.432355078663424	0.228523231047536	1	Hras
**1.68111870021715e-05**	0.289049572691435	0.232179303686991	1	Fbn1
**1.70443936985692e-05**	-0.838957674807809	0.235400121370939	1	Tpm1
**1.73521107317849e-05**	0.293939654306032	0.239650001316682	1	Tspo
**1.73890458873147e-05**	0.257850602304398	0.240160112749704	1	Add3
**1.73950933100805e-05**	-1.362283577	0.240243633705522	1	Sgcg
**1.75109631620113e-05**	-0.819077726785218	0.241843912230538	1	Slmap
**1.77989700258511e-05**	-0.777854192798107	0.245821575027029	1	Msrb3
**1.81740479739756e-05**	0.571318564408693	0.251001776568577	1	Ppdpf
**1.86621034461295e-05**	0.623773637688135	0.257742310694494	1	Apobec1
**1.88668828419333e-05**	-1.210095349	0.260570518929941	1	Ldb3
**1.89020804577553e-05**	-0.577689812571316	0.261056633202058	1	Zbed6
**1.90192339265378e-05**	0.393635099720693	0.262674639759414	1	Dact3
**2.06066959865677e-05**	-0.644354176348765	0.284599078270487	1	St5
**2.10779794352229e-05**	0.627887397869369	0.291107973979864	1	Lgi4
**2.15802006215462e-05**	-0.571248450160253	0.298044150784175	1	Cytl1
**2.21153908100482e-05**	0.406810668790146	0.305435662477576	1	Ccl19
**2.30710125664287e-05**	0.261859989374748	0.318633754554947	1	Rpl10
**2.3483301001853e-05**	-0.67384273	0.324327870136592	1	Kctd10
**2.41671617798518e-05**	0.541561404	0.333772671341533	1	Phf11d
**2.4205336784506e-05**	0.556136701922385	0.334299906330813	1	Pole4
**2.58483373602169e-05**	0.377914106819905	0.356991387281956	1	Igf2r
**2.5965789588196e-05**	0.484701779	0.358613520002576	1	Dnm1
**2.63162790515652e-05**	0.461150035932288	0.363454129981167	1	Dbn1
**2.65602626764687e-05**	0.332124164	0.366823787824709	1	Mfap5
**2.72383355178412e-05**	0.311525368195934	0.376188651836905	1	Romo1
**2.73383805624844e-05**	0.483831361914581	0.377570373948472	1	Atp6v0a2
**2.73842084921149e-05**	-0.601400805485412	0.378203303484599	1	Lox
**2.79213636983885e-05**	0.366939273293917	0.385621954038443	1	Psmb2
**2.85284181873217e-05**	0.375053932750431	0.394005983585099	1	Sec61b
**2.98214160632836e-05**	0.602158084387694	0.411863577250009	1	Aldh1a1
**3.06094581626783e-05**	-1.226630777	0.422747227	1	Kcnmb1
**3.1579186164743e-05**	0.748481757648938	0.436140140121266	1	Cdh11
**3.17625064852929e-05**	0.629440829145873	0.438671977068381	1	Serf1
**3.28104697236789e-05**	-1.260326389	0.453145397	1	Myocd
**3.40047623518832e-05**	0.505046155694668	0.469639772841859	1	Gpsm1
**3.43328310037775e-05**	0.250879946196457	0.474170728993171	1	Rpl37a
**3.44481567901911e-05**	0.420942666762201	0.475763493429329	1	Rpl22l1
**3.46335841641578e-05**	0.460561467849972	0.478324430891184	1	Smyd2
**3.56675541208186e-05**	0.631219580384516	0.492604589962626	1	Cisd2
**3.60570417286563e-05**	0.285755180384911	0.497983803314472	1	Npc2
**3.64550075825422e-05**	0.431495143186279	0.503480109722491	1	Ube2q2
**3.76475949294229e-05**	0.271323650873441	0.519950934	1	Rps16
**3.79440222424025e-05**	0.618731742096984	0.524044891189822	1	Ahi1
**3.84844881543814e-05**	-0.808326982073288	0.531509265900162	1	Epas1
**3.87525989063923e-05**	-1.408997754	0.535212143496184	1	Gpr116
**3.99841414953493e-05**	-0.622450051959869	0.552220978192269	1	Cyp1b1
**4.0571690159566e-05**	-1.11709955	0.560335612793766	1	Ephx2
**4.07448651436164e-05**	-0.624127841010213	0.562727332498486	1	Pdlim3

**4.09790043012088e-05**	0.269272336524443	0.565961028403995	1	Golga7
**4.12037642783611e-05**	0.463130243942772	0.569065188448446	1	Il1r1
**4.15029697262794e-05**	0.317735977074449	0.573197514889645	1	Numbl
**4.15991458799016e-05**	-0.613715861816621	0.574525803747321	1	Wwp2
**4.2101843379641e-05**	0.405533560067139	0.581468558916221	1	Ugdh
**4.22021509283964e-05**	-0.972965252845622	0.582853906472083	1	Pde5a
**4.23757375708577e-05**	0.579348136391999	0.585251311591115	1	Ptgfrn
**4.24458357068277e-05**	-1.845943125	0.586219436946997	1	Mylk4
**4.31726721100608e-05**	0.846149366451558	0.596257775	1	BC017643
**4.32965620493522e-05**	-1.079599003	0.597968818463603	1	Hey2
**4.4338221977433e-05**	0.291160992885666	0.612355183730327	1	Pabpc4
**4.4653804198192e-05**	0.622155036465623	0.616713689781229	1	C1qtnf7
**4.51379013858147e-05**	0.492509560266242	0.623399556039487	1	Yif1a
**4.77175339339648e-05**	-1.488247275	0.659026861161988	1	Fmo3
**4.84294130637825e-05**	0.394841241301829	0.668858624	1	Mrps33
**4.8991477234109e-05**	0.531572819	0.676621292	1	Sdccag3
**4.9479767106803e-05**	0.439375058639921	0.683365063512056	1	Prelid1
**4.96793355658521e-05**	0.262492719748619	0.686121303499983	1	Col15a1
**4.9708770835964e-05**	-1.81365328	0.686527834015499	1	Cd24a
**5.03588149390549e-05**	0.463780649718383	0.695505593123288	1	Tmem41b
**5.07761205358229e-05**	0.473763965957269	0.701269001	1	Atxn10
**5.25245229492398e-05**	0.329344949936333	0.725416186451951	1	Pomp
**5.30658873091739e-05**	0.261404082832117	0.732892969627001	1	Il33
**5.35806674529717e-05**	0.304604765	0.740002598192993	1	Rpsa
**5.44368339770286e-05**	0.452099024148666	0.751827114056742	1	Alyref
**5.47199809629924e-05**	-0.624048596	0.755737657079889	1	Ttc28
**5.47864454173947e-05**	-1.310614226	0.756655597659639	1	Mtus2
**5.91890155158356e-05**	-1.591573392	0.817459493289206	1	E030013I19R
**6.02734502702134e-05**	-0.386380818628917	0.832436621681918	1	Amotl2
**6.189932016979E-05**	-1.097370121	0.854891510864969	1	Sema3f
**6.28479014260113e-05**	0.279489530690233	0.867992366594642	1	Fau
**6.28971215323072e-05**	-0.775484635506359	0.868672145482695	1	Nuak1
**6.32968553546204e-05**	0.503969878621436	0.874192869302662	1	Pmm1
**6.41142501552328e-05**	0.279393575787583	0.885481909	1	Prdx2
**6.43799098139753e-05**	0.418203038282342	0.889150934440812	1	Anpep
**6.47913779193497e-05**	0.390521580157312	0.894833720444139	1	Ccdc53
**6.61051207969764e-05**	0.325477285662548	0.912977823327041	1	Herpud1
**6.72300205368835e-05**	0.384729548883191	0.928513813634899	1	Fbxw8
**6.73002815198953e-05**	0.684973059196621	0.929484188071275	1	Col12a1
**6.73978650818257e-05**	0.261184388132392	0.930831914645095	1	Col6a2
**6.78546367323066e-05**	0.437722003	0.937140387909886	1	Manf
**7.04626955863841e-05**	0.525482238668456	0.973160288743551	1	Ints4
**7.1839548775351e-05**	0.408841238	0.992176008136373	1	Sh3d19
**7.20194331758604e-05**	0.728486660088531	0.994660391591808	1	Tril
**7.27589765504738e-05**	0.784175481912452	1	1	Crlf1
**7.34148697076709e-05**	0.343593267205296	1	1	Hand2
**7.48555124364193e-05**	0.285210956486132	1	1	C4b
**7.68158556391998e-05**	0.460249969	1	1	Vdac3
**7.88268045816485e-05**	0.472954592936313	1	1	Aspn
**7.8972535950664e-05**	0.407639487670495	1	1	Ptplb
**8.02044643604759e-05**	0.393131857670007	1	1	Trps1
**8.13397691830214e-05**	0.633724830428073	1	1	Tenm3
**8.19054789959736e-05**	-0.289138874	1	1	Actb
**8.24973153247225e-05**	0.531249251066704	1	1	Commd6
**8.25009120918416e-05**	0.397536257156697	1	1	Arntl
**8.25508122681349e-05**	0.281222595225364	1	1	Snrpd2

**8.47907840518215e-05**	0.417507288378516	1	1	Sra1
**8.70135703715974e-05**	0.407634007722993	1	1	Usp22
**8.70666624341197e-05**	0.326999631390785	1	1	Lipa
**8.76445781805579e-05**	0.555079582371917	1	1	Papss2
**9.11095274361052e-05**	0.649778861952155	1	1	Apbb1ip
**9.20813251398107e-05**	0.323915956	1	1	Nsa2
**9.2273679448351e-05**	-0.685449765461661	1	1	Cav2
**9.26102462997324e-05**	0.388831809328163	1	1	Uqcc2
**9.66787696809851e-05**	-1.263381262	1	1	Npy1r
**0.000100094122746365**	0.792951218447294	1	1	Cbr3
**0.000101516476902479**	0.278142574342728	1	1	Rps15a
**0.000101607368165138**	-0.688873654173171	1	1	Adcy5
**0.000101612258082556**	-0.505423290723608	1	1	Mical2
**0.000102671827931422**	0.631144584989506	1	1	Gpr153
**0.000104331878812366**	-1.423376292	1	1	Podxl
**0.000104673019212935**	0.751934189	1	1	Kctd15
**0.000106596111381375**	0.377939430499225	1	1	Srprb
**0.000112454976567899**	-0.847615373020041	1	1	Neat1
**0.000112546213305647**	0.353775607	1	1	Medag
**0.000117879198008548**	0.366275503084359	1	1	Atp6v1g1
**0.000120593125021391**	0.630556898564674	1	1	Gfra1
**0.000120700444461358**	0.284070857104795	1	1	Fkbp7
**0.000122492576388878**	0.414486786341477	1	1	Bcap31
**0.000122944155568708**	0.324466148867617	1	1	Hypk
**0.000123523657376224**	-0.563480679945735	1	1	Vcl
**0.000125316631132277**	0.334802794154311	1	1	Tmem176b
**0.000125404612328321**	0.506333322817935	1	1	Hr
**0.000125453430763785**	-0.864338932	1	1	Enah
**0.000126048657558646**	0.465424359612115	1	1	Cdk4
**0.00012780224061792**	-0.514320480925009	1	1	Arhgap29
**0.000129131784390342**	-0.474472786876211	1	1	Cd200
**0.000130865680735008**	0.540096685286345	1	1	Cand1
**0.000134609749439527**	-1.177866263	1	1	Mmp17
**0.000135790183597581**	0.318531965769327	1	1	G0s2
**0.000136955838438665**	0.390317854651677	1	1	Lpar1
**0.000138126573768633**	0.323095127281817	1	1	Socs2
**0.000138151849959128**	0.474993190120933	1	1	Stx18
**0.000142978505988106**	0.658239220452134	1	1	Spry4
**0.000144141213661291**	0.415071787523206	1	1	Ppp2r3a
**0.000146606106728443**	0.352324668562067	1	1	Oaf
**0.000147448257192066**	0.271117034953554	1	1	Rbm8a
**0.000149178309394347**	-0.716880228600409	1	1	Rbpms
**0.000149906053209545**	-0.650349280435462	1	1	Chd3
**0.00015274507871868**	0.502807257550029	1	1	Sdhd
**0.00015606817166896**	0.489931227229799	1	1	Cd55
**0.000156374213762076**	-0.585581679	1	1	Pygm
**0.000158501131500057**	0.371963524	1	1	Gtf2h5
**0.00016012336237888**	-0.643349517568603	1	1	Slit3
**0.00016093486607931**	0.498037565649731	1	1	C1d
**0.000167362647970288**	0.446761298480188	1	1	Pqlc3
**0.000171142507988194**	-0.983984825210536	1	1	Phtf2
**0.000173707575592306**	-1.509426433	1	1	Tie1
**0.000173866516615352**	0.502445988764645	1	1	Slc44a1
**0.000174157358990055**	-1.230566949	1	1	Speg
**0.00017566047960629**	0.268942993094394	1	1	Hint1
**0.000179004817545052**	0.279846849	1	1	Pdgfra

**0.000179112737923903**	0.459763486634967	1	1	Kbtbd2
**0.000179204785202004**	0.358892543037378	1	1	Fam102b
**0.000179418938972929**	-1.117444014	1	1	Lmo2
**0.000181782117370273**	0.343220619539348	1	1	Ak1
**0.0001826362679409**	0.531888438210162	1	1	B4galt5
**0.000187309247696611**	0.370397409527274	1	1	Tgfbr3
**0.000187606006976641**	0.303190345922293	1	1	Sri
**0.000188878591776483**	-0.487927921422021	1	1	Fbln5
**0.000202952018319819**	0.326932385937724	1	1	Il13ra1
**0.000203395115340374**	-0.498253493539112	1	1	Mfge8
**0.000209580617668512**	0.288083952992388	1	1	Cct5
**0.000210056446967433**	-0.666778303120927	1	1	Npr1
**0.00021078001738307**	0.504589996829436	1	1	Chkb
**0.000214120732311708**	0.709052785392245	1	1	Twist2
**0.00021790225472141**	0.268058736224117	1	1	Cstb
**0.000218416253923125**	0.284020965157807	1	1	Prnp
**0.000221013965510968**	-0.478775952823808	1	1	Setd5
**0.000221503767688046**	0.461546807545163	1	1	Gm3219
**0.000222340744324917**	-1.51058377	1	1	5930403L14R
**0.000224767966136598**	0.494461951796496	1	1	Enpp1
**0.000227211163529421**	0.302733241050323	1	1	Akt2
**0.000228060613544131**	-0.518177729598584	1	1	Oxct1
**0.000233867742906386**	-0.403459058246483	1	1	Sema3d
**0.000233963841101758**	0.435203009250998	1	1	Carhsp1
**0.000235275229529408**	0.332938664039902	1	1	Nhp2l1
**0.000236109945164259**	0.478424302245621	1	1	Col16a1
**0.000245270815826742**	-0.546752804017434	1	1	Tm4sf1
**0.000247460668089886**	0.768838054	1	1	Tmem184c
**0.000255598721109224**	0.727691520464578	1	1	Ccdc157
**0.000259463891893164**	0.492001068133023	1	1	Fndc1
**0.000259584595420068**	0.428474300560808	1	1	Mif
**0.000264689799914428**	0.282006690131791	1	1	Dnajc19
**0.000267717481179868**	0.461865117881702	1	1	Palm
**0.000270075198125297**	-0.643584486593203	1	1	Pdlim4
**0.000272090397646686**	0.446367930201424	1	1	Gpx7
**0.000274911168538653**	-0.306708334316326	1	1	Col4a1
**0.000282716119770494**	-0.567015106985949	1	1	Igf1r
**0.000287268740011112**	-0.687510109103574	1	1	Pltp
**0.000287303765411732**	-1.177778684	1	1	Cbfa2t3
**0.000288948577849182**	0.260617368666185	1	1	Ier3ip1
**0.000290197811596561**	0.543781870712298	1	1	B3gnt9
**0.000306490492022606**	0.279516775915072	1	1	Entpd2
**0.000307625038776674**	-0.381745979801725	1	1	Adamts1
**0.000311902761319176**	0.373775894880934	1	1	Rnf2
**0.000313901219596241**	0.541488746551231	1	1	1700025G04R
**0.000313957536396315**	0.412720158317947	1	1	Mbd6
**0.000313984953463154**	0.387123509198357	1	1	Bmper
**0.000316544852541691**	-0.874422131641052	1	1	Itga1
**0.000320130600984647**	0.410769009963198	1	1	Ugp2
**0.000320507404532941**	0.286854131580466	1	1	Ly6a
**0.000321739602405386**	0.289769412348167	1	1	Abhd14a
**0.000322118791657597**	0.575989388138875	1	1	Tmem50b
**0.000325412757972703**	0.502084437839382	1	1	Bccip
**0.000326159015121809**	0.393383848435108	1	1	Actr2
**0.000328682951895579**	0.310893464106213	1	1	Hiatl1
**0.00032932050932578**	0.472298588783686	1	1	Fam26e

**0.000329378627390987**	0.598335785394084	1	1	Fam63a
**0.000331976639939407**	0.322940318414667	1	1	Dnajc3
**0.000332088120651997**	0.256252843081652	1	1	Crispld2
**0.000335421052255382**	0.268963174278051	1	1	Atp6v0d1
**0.000338824546974107**	-1.18358446	1	1	Perp
**0.000339797583374352**	0.256021531727864	1	1	Vimp
**0.000349647516586729**	-1.316827294	1	1	Fnbp1l
**0.000352307424071369**	0.350148311480338	1	1	Banf1
**0.000353228043840279**	0.327592157370268	1	1	Lgals3bp
**0.00035585490619733**	0.501349022155053	1	1	Clmp
**0.00036141066279743**	0.835030607837138	1	1	Col5a3
**0.000374931548340755**	0.312510035977047	1	1	Comt
**0.000380406512769674**	0.485544331612928	1	1	Spin1
**0.00038098418964213**	0.275461644053622	1	1	Ngf
**0.000381145689932455**	0.665773586380444	1	1	Tmem100
**0.000382992098826052**	0.412270270934486	1	1	Tmem29
**0.000389047092845211**	0.295932814355659	1	1	Ociad1
**0.000397190860233951**	0.666236665639784	1	1	Dpm2
**0.000398131651404889**	0.526787726004924	1	1	Angptl1
**0.00040609451481304**	0.416894002214559	1	1	2410006H16l
**0.000410758346299001**	0.577524019026443	1	1	Tmem9b
**0.000411088196239505**	0.346471015	1	1	Mme
**0.000411371294310806**	0.362960197120807	1	1	H2afv
**0.000419389403963412**	0.634141771804339	1	1	Arsb
**0.000421344831438009**	0.290441577241474	1	1	Cd44
**0.000423252543166233**	0.458848050429832	1	1	Gstm5
**0.000425551653695597**	0.789615686800288	1	1	Angpt1
**0.000434175947362139**	-0.648383981052318	1	1	Rrad
**0.000450070107867683**	0.301859239490591	1	1	Hnrnpa1
**0.000451649323147008**	0.594761069919223	1	1	Rcor3
**0.000453664184963406**	0.525933608326992	1	1	Sdc1
**0.000462400165652597**	0.262076158129844	1	1	S1pr2
**0.000469222001173613**	0.448160878834612	1	1	Twf1
**0.000477208911727991**	0.421906457959219	1	1	Cdk8
**0.000481409546232593**	0.629346394608914	1	1	Sgpp1
**0.000481774929392997**	0.285918944860514	1	1	Clptm1l
**0.000488585716932413**	-0.946359027710102	1	1	Rnf144a
**0.00050385249391613**	-1.13279446	1	1	Procr
**0.00051211378565081**	-1.25044528	1	1	Itgb3
**0.000522468909546703**	0.389727904956203	1	1	Dap
**0.000525643479200026**	0.693866080468694	1	1	Piezo1
**0.000525708531783986**	-0.738049668054316	1	1	Ntf3
**0.000528466154072356**	-0.634886330307319	1	1	Uaca
**0.000559965219839247**	-0.535478366497077	1	1	Foxp1
**0.000561209282670686**	0.378870962937337	1	1	Pgk1
**0.000593749657015367**	-1.333471229	1	1	Rasip1
**0.000605034711419257**	0.384364951814173	1	1	Capg
**0.000621602578391993**	0.809359260722043	1	1	Cx3cl1
**0.000624996250623598**	0.508548786	1	1	Cdk9
**0.000637747651831135**	0.497044253644624	1	1	Per2
**0.000642802984555127**	-0.422943863332488	1	1	Gadd45g
**0.000647449910837362**	0.459467949749303	1	1	Enpp2
**0.000647555967256231**	0.285069156958408	1	1	Fxyd5
**0.000652534885076033**	0.625453087223564	1	1	Commd9
**0.000666030151885**	0.420009113008756	1	1	Tmx2
**0.000672082783082451**	-0.579968459390587	1	1	Ppp1r12c

**0.000675459296382359**	0.836950703	1	1	Cinp
**0.000688411301895736**	0.676639677637476	1	1	2610034B18I
**0.000696670621511235**	0.349795506	1	1	Ufm1
**0.000699665201688714**	-0.754044585360973	1	1	Cdc42ep3
**0.000702137433287239**	0.419354321058768	1	1	Tm9sf1
**0.00070660265844927**	0.401287040932744	1	1	Cmpk1
**0.000723085737672368**	0.272923413706493	1	1	Farp1
**0.000725408688714816**	0.441010173429107	1	1	Tbrg1
**0.000735527291867129**	-0.932727161616705	1	1	Csrp1
**0.000751945584169795**	0.511028008796417	1	1	Med8
**0.000754165727569872**	-0.689043240656037	1	1	Ehbp1l1
**0.000764698305023565**	0.414971494608816	1	1	Ivns1abp
**0.000775972825343216**	0.285579762215769	1	1	Ctsk
**0.00078443279220039**	0.626508672198938	1	1	Micu1
**0.000794088130101683**	0.535620251053118	1	1	Cnpy4
**0.000796649527855089**	0.430261725677053	1	1	Snx2
**0.000821003249205439**	0.264805212361281	1	1	Morf4l2
**0.000837493646273459**	0.431135572279604	1	1	Nop16
**0.000862598179364022**	-1.042119114	1	1	Tek
**0.000863584759446651**	0.540198233902833	1	1	Adamtsl4
**0.000863607089891667**	-0.252624449179938	1	1	Ubc
**0.000865993676444816**	0.611447858884507	1	1	Ahsg
**0.000873075347091**	0.306340811464832	1	1	Smc2
**0.000873835315144943**	-0.621123221007448	1	1	Lifr
**0.000886097629918346**	-0.461371329074757	1	1	Zc2hc1a
**0.000890175718504862**	-0.461098758503641	1	1	Lrrc32
**0.000890744918607489**	-1.57793261	1	1	Ptprz1
**0.000902013155504853**	0.322167559911745	1	1	Fzd1
**0.000936668491238684**	0.422112163205534	1	1	Mark2
**0.000952972311289515**	0.311535960160462	1	1	Mxra7
**0.000969155274476418**	0.497178435181445	1	1	Apoa1bp
**0.000982279187639832**	0.448395730878904	1	1	Tmem55a
**0.00101491247617401**	-0.411633907295307	1	1	Gstm2
**0.00103345795869672**	0.468480881325569	1	1	Sidt2
**0.00106085813737505**	0.561046438	1	1	Cmklr1
**0.00106229567089577**	0.433976476045713	1	1	Lsp1
**0.00106406862041219**	-0.987168158820475	1	1	F3
**0.00108288927371293**	-1.090496852	1	1	Omd
**0.00108631787809799**	-0.497961509265112	1	1	Hip1
**0.00109594168216544**	-0.848119068326518	1	1	Gdf10
**0.00110433760567857**	0.394290246742735	1	1	Kremen1
**0.00110892768693192**	0.752303153094192	1	1	Itm2a
**0.00112087553329492**	0.674267460071809	1	1	Olfml1
**0.00113084023626943**	-0.471868096196629	1	1	Ddb1
**0.00113127305820658**	-1.514480161	1	1	Fbxo32
**0.0011335325278609**	-0.961376789814199	1	1	Efnb2
**0.00114361199342647**	0.770714394950035	1	1	Nol3
**0.00119276805019809**	0.337878285971357	1	1	Mgat2
**0.00122264272348106**	0.376319229	1	1	Tmem237
**0.00123569860994066**	-0.951059581126566	1	1	Ttll7
**0.00126344955732024**	0.626812706739604	1	1	Lbp
**0.00126893952136334**	0.252787483183399	1	1	Lrif1
**0.00127985173705255**	0.378447737628658	1	1	Cdv3
**0.001289974258809**	0.533542927561552	1	1	Otud7b
**0.00129056001035451**	-0.572343320714961	1	1	Map1a
**0.00129677588334903**	0.493581659514546	1	1	Evc2

**0.00130351168255118**	0.604149620739175	1	1	D19Bwg1357
**0.00132302949595284**	-1.002785584	1	1	Clec14a
**0.00132733928251525**	-0.359517110195934	1	1	Abcc9
**0.00132993514263359**	0.594861106933001	1	1	Tns3
**0.00133254014173669**	0.497257462	1	1	Slc16a1
**0.00135929173861061**	0.527767490186251	1	1	Kdelc1
**0.00138407277914518**	-0.628940368982929	1	1	Tacc2
**0.00139074336571324**	-0.433895225404325	1	1	Secisbp2l
**0.00139132129056275**	-0.412048565377495	1	1	Cux1
**0.00140496735270471**	-0.779893763855414	1	1	Mustn1
**0.00143752213389219**	0.277531305797228	1	1	Cyba
**0.00145356548687394**	0.48717256	1	1	Praf2
**0.0014546456634665**	-0.545957311514754	1	1	Dusp3
**0.0014563740191278**	0.260471514232965	1	1	Atp6v0e
**0.00145984419002316**	-1.050728793	1	1	Ppp1r14a
**0.00146486716059618**	-1.30461011	1	1	Net1
**0.00146701928409391**	-0.769039672186392	1	1	Atp1b2
**0.00147335246057844**	0.492911833231513	1	1	Dnajb11
**0.00148100055256058**	0.591761813019339	1	1	1810030O07
**0.00148589929415113**	0.334424743	1	1	Ddx19a
**0.00148674848052374**	-0.535067259975605	1	1	Pdlim7
**0.00149393810695244**	-0.408617747140636	1	1	Arid5b
**0.00149498321011633**	-1.910765301	1	1	Rgs7bp
**0.00151451673692656**	0.457079642293594	1	1	Lamtor1
**0.00152645404335285**	0.301848687394364	1	1	Acap2
**0.00157904381650473**	0.272965891694504	1	1	Txndc12
**0.00158187974542361**	0.730638302679346	1	1	Coq10a
**0.0015841595498827**	0.252181854127684	1	1	Nav1
**0.00159192912463676**	-0.702494723006676	1	1	Ripk1
**0.0016241621534621**	0.460307176481874	1	1	Angptl2
**0.00162503843875852**	0.385015375509678	1	1	Anapc13
**0.00165007254130987**	0.486248037040465	1	1	Unc5b
**0.00166016124887284**	0.584773654539747	1	1	N6amt2
**0.00166099122189853**	0.739292120813183	1	1	Sh3kbp1
**0.00168590674037402**	-0.906445813878147	1	1	Plin4
**0.00168811512093806**	0.652505347716003	1	1	Ptprf
**0.00169495584080872**	0.504922889130567	1	1	Dclk1
**0.00169822373544409**	0.432759356433021	1	1	1500009L16
**0.00170401451799607**	0.440278750412305	1	1	1810026J23
**0.00172765652777662**	0.407503823699506	1	1	Atp7a
**0.00176029371964962**	0.585722777810985	1	1	Fut11
**0.00176768928467329**	0.402818927833773	1	1	Clpx
**0.00177160049528805**	-0.465749456174751	1	1	Rock1
**0.00179964219892342**	0.316907497503113	1	1	Plin3
**0.00182423193407828**	0.290834644572026	1	1	Rnps1
**0.00183406103561566**	0.394131414072618	1	1	Egfr
**0.00183993040881861**	-0.494729666696668	1	1	Hdac5
**0.00184841723128904**	0.308122875985927	1	1	Otub1
**0.00185443150068525**	-1.10946761	1	1	Dock9
**0.00185633421975706**	-0.30162906	1	1	Itgb1
**0.00188797855987147**	0.528454246592683	1	1	Ap1s1
**0.00190081394781096**	-0.305715818444951	1	1	Mbnl1
**0.00192363191541662**	0.371984690875022	1	1	Tmbim1
**0.0019395918064571**	-0.821003581978432	1	1	Skap2
**0.00195413139055678**	0.261138826932302	1	1	Atp6ap2
**0.00196989539207877**	0.333559589238368	1	1	Tars

**0.00197343515504094**	0.427012189718931	1	1	Nans
**0.00198128470327962**	0.551523128813753	1	1	Comp
**0.00198582677783556**	0.322541721594739	1	1	Mrpl52
**0.00199892920686473**	-0.431183264085241	1	1	Ptplad2
**0.00203562883232775**	0.642238795731697	1	1	Plekha6
**0.00204894236504718**	0.267021375285303	1	1	Ywhaq
**0.00205180636449734**	-0.350554338543652	1	1	Gm15417
**0.0020573766624674**	0.262103693122016	1	1	Glis2
**0.00206443890106217**	0.399342563097135	1	1	Cab39l
**0.00209125918731674**	-0.450168532909349	1	1	Flnc
**0.00209266675443814**	0.828086188949425	1	1	Mphosph10
**0.00209577733661404**	-0.564347358	1	1	5031426D15R
**0.0021217988108841**	0.388020803457588	1	1	Pnkd
**0.00213091762605045**	0.526455088	1	1	Rnf38
**0.0021372015328351**	0.457123529129464	1	1	Abhd4
**0.00213758407729911**	0.295928130132372	1	1	Dram2
**0.0021776806290297**	0.357443115392643	1	1	H2afz
**0.00218491639640556**	0.298941597404884	1	1	Ostc
**0.00221733522105972**	0.485931717412265	1	1	Gli3
**0.00222050933987975**	-0.588869390955057	1	1	Efna5
**0.00222087345875389**	0.354946449956992	1	1	Arhgef40
**0.00222631839763959**	0.253217596643937	1	1	Gnpda2
**0.00222685891861559**	0.299211543406806	1	1	2010107E04R
**0.00222770817407833**	-0.658281880518212	1	1	Osr1
**0.00222814891637777**	0.355775647962689	1	1	Fkbp3
**0.00224076289920293**	0.500824961392504	1	1	Arhgdib
**0.00226418097660207**	0.608544591	1	1	Dip2a
**0.00226435614720415**	0.592862689014381	1	1	Fam111a
**0.00226942620589511**	0.525164992520303	1	1	Fcf1
**0.00229241111461026**	-0.499269038490183	1	1	Pls3
**0.00229408291230083**	0.466042275952897	1	1	Gpn3
**0.00230021464648397**	-0.388175465083441	1	1	Prpsap1
**0.00231093974860088**	0.401254329	1	1	Cd320
**0.00234337336863007**	-1.054800001	1	1	Filip1
**0.00235766593485351**	0.588591493919406	1	1	Pex5
**0.00236325803649388**	0.328970328047665	1	1	Srp19
**0.00236922114623133**	-0.414631152113076	1	1	Phactr2
**0.00242638774890295**	-0.700756368367504	1	1	Trabd2b
**0.0024451471232643**	0.274841024165849	1	1	Jade1
**0.00246063971488041**	0.269497626216546	1	1	Elovl5
**0.00246221576123977**	0.420379088110806	1	1	Rhobtb3
**0.00246861418240718**	-1.152253936	1	1	Etl4
**0.00247406185011045**	0.580116866928434	1	1	Dguok
**0.00248798355398919**	0.381431830309125	1	1	Aimp1
**0.00250575996958341**	0.584253092320808	1	1	Polr2d
**0.00252855943465477**	0.364262205191772	1	1	Scp2
**0.00253442752210674**	-1.841281749	1	1	1190005I06R
**0.00253756695007684**	0.461970052428875	1	1	Polr2h
**0.00257203598200897**	0.321692419935264	1	1	F2r
**0.00257793185300155**	-0.953478242754745	1	1	Efhd1
**0.00257878906316051**	-0.802525823623827	1	1	Lama5
**0.00258322448640397**	0.308950017137832	1	1	Rbm15b
**0.00262828056473377**	0.401732557337718	1	1	Tspan12
**0.00268254199094124**	0.299261475	1	1	Rps23
**0.00272023964405051**	-0.253004205210035	1	1	Ctdspl
**0.00274357870770134**	0.296157330225055	1	1	Epb4.1l2

**0.00275456845256406**	0.258912043844328	1	1	Rora
**0.00277973932613857**	0.279320254346198	1	1	Nqo2
**0.00279505451905569**	0.318586105056191	1	1	Prmt2
**0.00282509293302528**	0.419731790146955	1	1	Acot10
**0.00287085894876965**	0.590131140104999	1	1	Sp3os
**0.00287594129772382**	0.324773198189509	1	1	Slc16a9
**0.00294186850472693**	0.632903445235206	1	1	Cblb
**0.00294621892714448**	-0.519511919154515	1	1	Mob2
**0.00298560000165976**	0.584709153668963	1	1	Epb4.1l3
**0.00298642794005637**	0.392718182	1	1	Rel
**0.00302558610437291**	0.396451288340987	1	1	Nid2
**0.0030401210240365**	0.312479514452543	1	1	Il1rap
**0.00305687033259939**	0.45061642	1	1	Thbs3
**0.00324106506464083**	0.397070334790324	1	1	Adamtsl2
**0.00328015322783021**	0.326725237430335	1	1	Abce1
**0.00336651309903236**	-0.705728700868726	1	1	Nacc2
**0.00339351215140328**	-0.849670741205978	1	1	Ptgs1
**0.00341991568998439**	0.628501812412201	1	1	Derl2
**0.00344919444847441**	-0.375023001744357	1	1	Ablim1
**0.00344973822522646**	-0.375280637025564	1	1	Atp13a1
**0.00345274592430094**	0.339451713115433	1	1	Eif2s1
**0.00348002623829926**	-1.059765821	1	1	Fam13c
**0.00355327158548477**	-1.087098273	1	1	Adh7
**0.003570961422857**	-0.398478343009293	1	1	Gnaq
**0.00371814265488264**	0.292920936731448	1	1	Arcn1
**0.0037365038575874**	0.315399675816518	1	1	Psmb5
**0.00375491273742417**	0.327856557714605	1	1	Rpl23
**0.00378106760757571**	0.366324696168373	1	1	Lemd3
**0.00378601623289155**	0.262866191890179	1	1	Ganab
**0.00379729874937103**	0.305095596589335	1	1	Nbl1
**0.00381009669495474**	0.250179529809209	1	1	Erp44
**0.00382806571826389**	-0.250199998918764	1	1	Hp
**0.00385880499308857**	-0.559916500078714	1	1	Pdlim1
**0.00388683500727535**	0.306918906521572	1	1	Cdon
**0.00389181291339044**	-1.178377621	1	1	Sorbs2
**0.00391004177060588**	0.383133170317754	1	1	Sugt1
**0.00392503602256291**	0.508065110299318	1	1	Cxcl16
**0.00395741287691855**	0.382130230762449	1	1	Rwdd1
**0.00400749397500704**	-0.784282341974816	1	1	Figf
**0.00403614515631929**	0.250002059403106	1	1	Igsf8
**0.0041028497764413**	0.814652665865964	1	1	Stk32b
**0.00410926276016299**	0.644697589130001	1	1	Lgals3
**0.00414159351374101**	0.343670078436318	1	1	Sept11
**0.00415822372243432**	-0.537579364628073	1	1	Phldb2
**0.00417301233632674**	0.290636284349213	1	1	Serpinb1a
**0.00418384383358611**	0.500201756959655	1	1	Cln5
**0.00419157573541728**	-0.470925607350057	1	1	Mef2c
**0.0042234779859408**	0.473180134793994	1	1	Fkbp5
**0.00423008607722109**	0.887850694471513	1	1	Cilp
**0.00423108383876883**	0.269291176307785	1	1	Brox
**0.00423538916098184**	0.656334452043159	1	1	Tln2
**0.0042457280655495**	0.281192797181604	1	1	Zfp451
**0.00425660816720467**	0.450485119695655	1	1	Loxl2
**0.004257619422786**	0.506704334447222	1	1	Rps12
**0.00429481010632614**	-1.113474433	1	1	Hoxa5
**0.00429555580867144**	0.523454193752451	1	1	Ebf2

**0.0043165996061272**	0.344591685	1	1	Mxd4
**0.00434324531370742**	0.883783549546533	1	1	Piezo2
**0.00434344931922143**	0.251806806116148	1	1	Ppic
**0.00440172112957196**	0.310434857368126	1	1	Rnh1
**0.00448626260813622**	0.337628004403288	1	1	Cdc14b
**0.00448732994033416**	-1.023765651	1	1	Selp
**0.00451362254161147**	0.322837538817494	1	1	Man2a1
**0.004517855249875**	-1.450649616	1	1	Pcp4l1
**0.00452967216165553**	-0.896740917118824	1	1	Ntrk3
**0.00455376552837369**	-1.022237037	1	1	Prdm16
**0.00459860128757851**	0.462979268160653	1	1	H2-T23
**0.00460921999707342**	0.480036087365061	1	1	Xpnpep1
**0.00461385997346926**	0.321135290068744	1	1	Snrpe
**0.00462943105835209**	0.259983582742231	1	1	Rer1
**0.00463506258926945**	-1.119137226	1	1	Rbpms2
**0.00465994751646473**	0.299645161	1	1	Seh1l
**0.00466162397262805**	0.271800619139137	1	1	Pdzrn3
**0.00467962962422884**	0.263956972958687	1	1	Runx1
**0.00469323069665905**	-0.383709395389029	1	1	Sh3pxd2a
**0.00469477455287971**	0.474155048021554	1	1	Traf3
**0.00471197932734125**	-0.632642478808876	1	1	Fyco1
**0.00472437379830344**	0.520407029913829	1	1	Gnptab
**0.00473135175441992**	-0.316777851089692	1	1	Ccdc107
**0.00474760248801855**	-0.491160559193605	1	1	Emb
**0.00475577766414015**	0.458879887397749	1	1	Mios
**0.00477706936746129**	0.508616761820006	1	1	Ccng2
**0.00478138949750131**	0.555801671096415	1	1	Rab31
**0.00478677352351435**	0.603282209	1	1	Thyn1
**0.00479646692337685**	0.398217530763894	1	1	Knop1
**0.00481979190449725**	-0.889511949336631	1	1	Plekhg3
**0.00483919610931401**	0.504710009938704	1	1	Bach1
**0.00490666619745646**	0.411924545805676	1	1	Dennd2a
**0.00493178231472797**	0.564325178223121	1	1	Dirc2
**0.00495407611910052**	0.306493705	1	1	Rnf44
**0.00495861950489732**	0.307095764878302	1	1	Vamp5
**0.00496083652602693**	0.309705052012231	1	1	Sf3b6
**0.00497977351309497**	0.357862132362245	1	1	Cnpy2
**0.0050062720872521**	-0.492955665181601	1	1	Thrb
**0.00502524162660401**	0.791790828182336	1	1	Prrx2
**0.00502653686281314**	0.535460724486461	1	1	0610009B22I
**0.00502721080673328**	0.563187010859392	1	1	Negr1
**0.0050914816891749**	0.431052842716672	1	1	Acvr1
**0.0050953304894354**	0.276792798360799	1	1	Lamtor4
**0.00510771226500115**	0.456971933118983	1	1	Prrc1
**0.0051583303384621**	-0.718319976834659	1	1	Plxna2
**0.00518424421039189**	-0.772385059216195	1	1	Osbpl3
**0.00520545309746299**	0.444593185556166	1	1	Ubtd2
**0.00523866003428097**	0.252449541014906	1	1	Lman2
**0.00524498712961469**	0.558990110400843	1	1	LOC1026365
**0.00532136793337548**	0.264182969	1	1	Ston2
**0.00532712833019247**	0.264452962991044	1	1	Vit
**0.00533859453412827**	0.414554396105065	1	1	Adpgk
**0.00536045052946542**	0.463428351	1	1	Hsdl2
**0.00538295997168994**	-1.123299486	1	1	Mtss1
**0.00547259120982052**	-1.032715591	1	1	Tnk2
**0.00548874879660109**	-0.460879691814484	1	1	Unc45a

**0.00549029279597775**	-1.346539352	1	1	She
**0.00550880340576714**	0.432896258343016	1	1	B9d2
**0.00551017147938819**	-0.504433556547332	1	1	Tle4
**0.00551889695489965**	-1.410595453	1	1	Gja5
**0.00558398124916818**	0.265792109965031	1	1	Rab32
**0.00561202213750437**	0.31048146	1	1	Zc3h14
**0.00562355484003757**	0.275912294448529	1	1	Zrsr2
**0.00564819819179895**	0.651088305	1	1	Pole3
**0.00565814430777654**	0.267420369824372	1	1	Zfp91
**0.00566179469221114**	-1.065730115	1	1	Sult5a1
**0.00584469402409612**	0.390910337305621	1	1	Gas7
**0.005846864039163**	0.738643955	1	1	Dlg5
**0.00585325090703229**	-0.881215331775095	1	1	Slc25a23
**0.00586620310760543**	0.629518153097272	1	1	Naa60
**0.00591632202474623**	0.339710736788863	1	1	P2rx4
**0.00592723205369633**	-0.796718776584736	1	1	Ctnnal1
**0.00599033063780622**	0.444428412720496	1	1	Zfp827
**0.0060028716477709**	0.616786081896311	1	1	Sac3d1
**0.00603701377826489**	0.291345596346343	1	1	Srp14
**0.00608378497079773**	0.363635743148878	1	1	Mcc
**0.0061019604166507**	0.311563804455168	1	1	Slc41a1
**0.00611629557353514**	0.380687493863735	1	1	Actr6
**0.00612053089660369**	-0.271100161812269	1	1	Ankle2
**0.00617468026322648**	0.566689523382107	1	1	2310033P09R
**0.00619214468988115**	0.335095659075644	1	1	Ppp2r3c
**0.00622578173910181**	0.939040736815685	1	1	Myoc
**0.00634966472126914**	0.483525139169717	1	1	Tiam1
**0.00636109513076951**	0.375269074613618	1	1	Eif4e2
**0.00639513432956973**	-0.512854506	1	1	Polr2b
**0.00644379554235094**	0.758440299166676	1	1	Ifit3
**0.00647654524694614**	0.364159214070672	1	1	Psmd14
**0.00652353493126031**	-0.553166328893567	1	1	Calcrl
**0.00654239569477873**	0.256294628708674	1	1	Prkra
**0.0065962439977866**	0.340626520879406	1	1	Nadk
**0.00660223731882774**	0.620872720869985	1	1	Naalad2
**0.00667567104456052**	-0.767747962	1	1	Ppp1r12b
**0.00674656715293822**	0.752697425450622	1	1	Myo5a
**0.00678251942435582**	0.488340592602104	1	1	Tstd2
**0.00678512298781249**	-0.670125311	1	1	Dstn
**0.00678696059990258**	0.539801329749974	1	1	Tm2d3
**0.006798222807854**	0.259685333884578	1	1	Abhd12
**0.00681906396109179**	0.375346124722269	1	1	Cox16
**0.00683870831072945**	0.414846478929949	1	1	Pld1
**0.00683909354844712**	0.386589965280372	1	1	Sav1
**0.0069757204102362**	0.671945151940268	1	1	Osbpl8
**0.00699373079580032**	0.324524273195571	1	1	Maob
**0.00714819981744799**	0.722002947	1	1	Ifih1
**0.00715008583065774**	0.830813607100086	1	1	Dut
**0.00717568302450261**	0.462623521948113	1	1	Cmtr1
**0.00725639317013035**	0.567487532572365	1	1	Rtkn
**0.00737147371542448**	-0.280547114619002	1	1	Hsph1
**0.00740027139220972**	-0.453646387220964	1	1	Mkl2
**0.00743192900467899**	-0.326748829	1	1	Mapkap1
**0.00745992776386725**	-0.401964368681794	1	1	Samd4
**0.00756049386484447**	0.284664795122792	1	1	Copb2
**0.00756623012330358**	0.345065369807638	1	1	Nabp2

**0.00757023152647665**	-0.455799240582065	1	1	Insr
**0.00760109194692837**	0.778704519557134	1	1	Ifit1
**0.00764713994911929**	0.370289724674583	1	1	A830080D01I
**0.00771994003771141**	0.488391328820889	1	1	Tcp11l2
**0.00784715435165332**	0.281886655289954	1	1	Mapre3
**0.00787852004697869**	-0.678075781582342	1	1	Plcd1
**0.00793828782800322**	-0.479601603070244	1	1	Lbh
**0.00795339274168248**	-0.593499017	1	1	F11r
**0.00796744342671959**	0.43661591	1	1	Wdr77
**0.0080012426000469**	0.379934152886822	1	1	Tgfb3
**0.00802333348113006**	0.558020779692191	1	1	9130401M01
**0.00805428258935535**	-0.730815506	1	1	Tbx18
**0.00809760448443237**	0.545106381979667	1	1	Xaf1
**0.00817164581798037**	0.254389405832732	1	1	Tug1
**0.00831526515424338**	-0.562600842613144	1	1	Hspb2
**0.00849412807364314**	0.272575187838113	1	1	Nedd9
**0.0086437767364157**	0.491448133	1	1	Rap2b
**0.00876893986710614**	-0.505708623371293	1	1	Tmpo
**0.0087722963379842**	-0.853007712	1	1	Itga7
**0.00879201727373921**	0.594075524184995	1	1	Gng8
**0.00880970125449205**	-0.484008708776015	1	1	Pdcd4
**0.00884061179336609**	0.648976395793713	1	1	Ttc7
**0.00885619629231613**	0.408141345	1	1	Gab2
**0.00890868887699721**	0.382723904890063	1	1	Fam220a
**0.00894006941703659**	-0.567664608331984	1	1	Heg1
**0.00895457014760371**	0.415894650499661	1	1	Tubgcp3
**0.00896154722010853**	0.387720217	1	1	Yipf1
**0.00901561985195815**	-0.872234136847954	1	1	2900026A02I
**0.00916396092172892**	0.387230429082234	1	1	2510039O18I
**0.0092184360513801**	-0.547778751390462	1	1	Mtus1
**0.00929813846940229**	0.407947588807129	1	1	Mpp1
**0.00942454538455703**	0.350441400769128	1	1	Pgrmc1
**0.00944467151994978**	-0.771073788955601	1	1	Plekha1
**0.00946808581900639**	-0.511583560482079	1	1	Flnb
**0.00949543713934722**	-0.475301975805669	1	1	Ktn1
**0.00953980225295212**	0.324182015629785	1	1	Smim7
**0.00955090078973134**	0.290455777123419	1	1	Tmem64
**0.00959605581188025**	0.565849208960552	1	1	Vps4a
**0.00962128808268694**	0.367043213	1	1	Pfn2
**0.00964379675819161**	0.386350085876728	1	1	Alg14
**0.00965665897625371**	-0.402776205	1	1	Pde4dip
**0.00973450247346472**	0.556551413407628	1	1	Fam104a
**0.00976868036461321**	0.361539200063032	1	1	Llph
**0.00989373876734698**	0.338076893410551	1	1	Arl5a
**0.00993556804402686**	-0.983984825210536	1	1	Slc9a3r2

p_val	avg_logFC	p_val_adj	cluster	gene
**5.6273920069279e-25**	0.905809712346525	7.77199110076812e-21	2	Mmp3
**3.67705218517664e-24**	0.861055956029762	5.07837677294745e-20	2	Gpx3
**3.93889337797183e-24**	0.911509595415816	5.44000564431689e-20	2	1500015O10[illegible]
**1.98707012252282e-22**	0.948396330867066	2.74434254621627e-18	2	Ltbp2
**3.16059725008836e-21**	0.866007196605403	4.36510086209703e-17	2	Serpina3n
**1.4463209240468e-20**	0.888148433228801	1.99751382820103e-16	2	Comp
**4.54288775470074e-20**	0.909291335399861	6.27418227801719e-16	2	Prg4
**2.31134121315462e-19**	0.846940327682896	3.19219334948785e-15	2	Timp1
**4.60115259815401e-19**	1.242965289	6.35465185331051e-15	2	Hdac6
**8.27835597117368e-19**	0.717557164	1.1433237431788e-14	2	Frzb
**1.22851197349226e-18**	0.674514534142804	1.69669788659015e-14	2	Cxcl1
**1.2789416847639e-18**	1.093667411	1.76634636082742e-14	2	Nr4a2
**3.14033245218305e-18**	0.748811600242197	4.33711314971001e-14	2	Crispld2
**1.41260026240158e-17**	-0.644085104815417	1.95094222240282e-13	2	Gm1821
**2.23726096121415e-17**	0.666235120905436	3.08988111353286e-13	2	Lamb1
**2.31114743906261e-17**	0.506252060410611	3.19192572808938e-13	2	Col1a1
**4.73266328854218e-17**	1.371693012	6.53628126780561e-13	2	Lcn2
**4.95851394263347e-17**	0.713161976927744	6.84820360617108e-13	2	Sfrp2
**5.55797333998376e-17**	0.656559191606581	7.67611697985158e-13	2	Ptgs2
**1.89509866597847e-16**	0.690656360466699	2.61732076758287e-12	2	Il6
**2.00245590071007e-16**	-0.759481654380287	2.76559184447068e-12	2	Rps7
**3.15430850921265e-16**	-0.692582822867611	4.3564154820736e-12	2	Rps26
**7.72133252394172e-16**	-0.566654262115971	1.06639323488159e-11	2	Mir682
**8.96924676487196e-16**	0.646283014699409	1.23874267069647e-11	2	Cfb
**1.05066297261716e-15**	1.000585807	1.45107063148155e-11	2	Col8a1
**1.17800067183052e-15**	0.749982693003266	1.62693672786512e-11	2	C4b
**1.60313799260118e-15**	0.544423867042792	2.21409388158149e-11	2	Ccdc80
**1.75259224696592e-15**	0.452937521564567	2.42050515228464e-11	2	Matn2
**2.34841623631182e-15**	0.561964683897303	3.24339766397025e-11	2	C3
**2.56242897100877e-15**	0.596735679	3.53897065186022e-11	2	Ccl7
**3.15606094960639e-15**	0.869990447235942	4.35883577750139e-11	2	Anxa8
**4.03550064774137e-15**	-0.572648598249996	5.57342994459561e-11	2	Prdx1
**4.40979993309134e-15**	0.977687875579306	6.09037468759245e-11	2	Il33
**4.52020914314964e-15**	-0.583630472251218	6.24286084760397e-11	2	Rps21
**5.34777995132161e-15**	0.631877538387573	7.38581889077028e-11	2	Cygb
**8.90693410005813e-15**	-0.600207137553159	1.23013666855903e-10	2	Lars2
**1.11364671095017e-14**	0.670736681257863	1.53805747249328e-10	2	Fam46a
**1.5249628532768e-14**	1.037672741	2.10612619666059e-10	2	Gpm6b
**1.97305784480224e-14**	-0.577006840061711	2.72499018945638e-10	2	Tmsb4x
**3.26867064716039e-14**	0.541726277358457	4.51436103079322e-10	2	Penk
**6.01990612277582e-14**	0.759904821892754	8.31409234616568e-10	2	Tnfaip6
**6.32768433398063e-14**	0.775451995559073	8.73916483366065e-10	2	Lpl
**6.72976140789708e-14**	1.014514444	9.29447348044666e-10	2	Rhou
**7.04927502721508e-14**	0.614724310602952	9.73575374008675e-10	2	Lgals3bp
**7.13523197880648e-14**	0.539593888536588	9.85446888592962e-10	2	Efemp1
**1.15968225183621e-13**	0.543644589	1.60163715801100e-09	2	Dpep1
**1.21492211213387e-13**	0.410573736709666	1.67792892906808e-09	2	Inmt
**1.23609300278442e-13**	-0.688961774	1.70716804614556e-09	2	Tpt1
**1.27988694377533e-13**	0.722135401483638	1.76765185804811e-09	2	Cnot6
**1.51880434347138e-13**	0.605229604498587	2.09762067876833e-09	2	Fmod
**1.78951187330171e-13**	0.716813894805915	2.47149484821699e-09	2	Slc16a2
**1.8842131550718e-13**	0.576443359983759	2.60228678846967e-09	2	Uhrf1bp1
**2.22599933645349e-13**	-0.403778055831186	3.07432768357591e-09	2	Rps3a1
**2.44843077857491e-13**	0.646120518344529	3.3815277482898e-09	2	Adamts5
**3.28029133080022e-13**	0.489509890443364	4.53041035696819e-09	2	Gja1

**3.65935566125334e-13**	0.559786088569672	5.05393610375699e-09	2	Cxcl12
**4.37302053438819e-13**	-0.410220261642752	6.03957866004353e-09	2	Pfn1
**1.02315917124979e-12**	0.509422318816299	1.41308513141309e-08	2	Cp
**1.25099144464799e-12**	0.321021329066561	1.72774428420333e-08	2	Osmr
**1.39907128568228e-12**	0.408432503850979	1.9322573526558e-08	2	Mt2
**1.66243408825703e-12**	0.624346777864458	2.29598771929178e-08	2	Nfkbiz
**1.82880274503523e-12**	0.276846974946861	2.52575947116815e-08	2	Cpxm2
**1.96412296043235e-12**	0.589350891500515	2.71265022065312e-08	2	Trf
**3.03399951435563e-12**	0.533713135003123	4.19025672927656e-08	2	Xdh
**3.20377928341808e-12**	-0.598930952707561	4.42473956832872e-08	2	Rpl35a
**3.42643138347662e-12**	0.507470523473659	4.73224438371956e-08	2	Spon1
**3.91815728830226e-12**	0.492740188560751	5.41136703087425e-08	2	Wisp2
**4.95284804454562e-12**	1.112729604	6.84037843432196e-08	2	Slfn2
**4.96646353244664e-12**	0.800222153570421	6.85918278466205e-08	2	Cxcl2
**5.09840235677503e-12**	0.872206866988633	7.04140349494199e-08	2	Itih5
**5.34079279995794e-12**	0.542733039718391	7.37616893602191e-08	2	Vcan
**7.04448202285006e-12**	-0.848960417	9.72913412175822e-08	2	Rpl37a
**7.06886660012223e-12**	0.626849309290806	9.76281166142881e-08	2	Ctsk
**7.25518734546405e-12**	0.503938051858348	1.00201392428204e-07	2	Lum
**7.68373929398646e-12**	-0.514014711835499	1.06120123389247e-07	2	Cd9
**8.55218368671021e-12**	0.965898081799931	1.18114208897155e-07	2	Ppl
**8.63501194549781e-12**	0.748433045646465	1.1925814997927e-07	2	Suv39h1
**1.09412667194811e-11**	0.534052351923052	1.51109834662754e-07	2	Tmem176b
**1.12752712360139e-11**	0.512871413389654	1.55722771040588e-07	2	Cpxm1
**1.16064065172236e-11**	0.818050930402238	1.60296080409375e-07	2	Fbln7
**1.24706619664501e-11**	0.261036448554526	1.72232312418642e-07	2	Steap4
**1.50474361219416e-11**	0.498063931926953	2.07820140280135e-07	2	Scarf2
**1.59080119045267e-11**	0.451325382177643	2.19705552413419e-07	2	C7
**1.98655654275354e-11**	0.671775941974538	2.74363324119691e-07	2	Col16a1
**2.02598990942768e-11**	-0.417661811432479	2.79809466391056e-07	2	Gm6654
**2.02807940875757e-11**	-0.522899396582455	2.80098047143508e-07	2	Cox6c
**2.20484994667121e-11**	0.484936575472729	3.04511826134761e-07	2	Mmp2
**2.44425455324923e-11**	-0.351115365633416	3.37575996349251e-07	2	Rplp0
**2.75340943442681e-11**	-0.356720765840464	3.80273376988686e-07	2	Rps11
**2.89570083214282e-11**	0.480277921659531	3.99925241927244e-07	2	Dpt
**2.92289982765113e-11**	-0.346469341	4.03681695196898e-07	2	Sepw1
**2.99063044370884e-11**	-0.541488329042393	4.13035970580628e-07	2	Rpl14-ps1
**3.94553585292906e-11**	-0.265114362763128	5.44917956648032e-07	2	NP-904340.1
**3.95338932530232e-11**	-0.337332365513269	5.46002599717503e-07	2	Rps20
**4.23073577838289e-11**	-0.796306093382097	5.84306918352461e-07	2	BC048507
**4.71290501888037e-11**	0.481350341205187	6.50899312157567e-07	2	Tgfbi
**5.48427836380849e-11**	0.371728640486917	7.5743368482559e-07	2	Vcam1
**5.49550718435015e-11**	0.887823983540512	7.58984497230599e-07	2	S1pr3
**7.89898642006242e-11**	0.511200470355346	1.09092901447482e-06	2	Slc7a2
**8.3772742355905e-11**	0.682831752566685	1.1569853446774e-06	2	Gda
**1.35375365024752e-10**	0.507129675317076	1.86966916635685e-06	2	Cxcl16
**1.3819754466314e-10**	-0.413037766465425	1.90864628934262e-06	2	Mir6236
**1.48289759740291e-10**	0.387650981353068	2.04802987177316e-06	2	Ccl2
**1.76681018330316e-10**	-0.509878334459268	2.44014154415999e-06	2	Rps18
**1.84359037402378e-10**	0.332628531566546	2.54618266556424e-06	2	Ifi27l2a
**1.91790163488962e-10**	0.431752357891688	2.64881394794605e-06	2	Igfbp4
**1.98334131260494e-10**	0.413199815929699	2.73919268683868e-06	2	Tgfb3
**2.07893614950069e-10**	-0.330142733367225	2.8712187160754e-06	2	Tmsb10
**2.40571660277496e-10**	0.424486875012946	3.3225352000925e-06	2	Smoc2
**2.47282911873132e-10**	0.422618156620968	3.41522429587983e-06	2	Baiap2
**2.90414185048597e-10**	0.307937202125321	4.01091030970617e-06	2	Aldh1a1

**3.06559864169798e-10**	0.442856859198209	4.23389828404907e-06	2	Pid1
**3.25000532837536e-10**	1.023482306	4.4885823590192e-06	2	Cxcl14
**3.37010750665642e-10**	0.362053185464652	4.65445547744318e-06	2	Mmp14
**4.07023603618645e-10**	1.489211088	5.6214029895771e-06	2	Pf4
**4.10630790497424e-10**	0.604374152139058	5.67122184755993e-06	2	Bmp1
**4.24843710652114e-10**	0.465713082683763	5.86751648781635e-06	2	Adamts2
**5.04578099075979e-10**	0.275307139024658	6.96872812633835e-06	2	Lrrc59
**5.2034958470537e-10**	0.760029790118961	7.18654811436587e-06	2	Pgf
**5.27778030373977e-10**	0.380999607207999	7.28914237749499e-06	2	Meg3
**5.38360267298899e-10**	0.424158917338135	7.43529365166509e-06	2	Scn7a
**6.3434312390904e-10**	0.459886733299434	8.76091288430776e-06	2	Sod2
**6.71490522348336e-10**	-0.618782114517929	9.27395560415287e-06	2	Gm6251
**6.75481173552703e-10**	0.928536971192404	9.32907048793638e-06	2	Gfra1
**7.15581367216168e-10**	0.417454093777727	9.88289426262249e-06	2	Srrm2
**7.74751227966309e-10**	0.431824787021312	1.07000892094427e-05	2	Cpz
**7.79454819248789e-10**	0.431478772796385	1.0765050508645e-05	2	Smc5
**7.91999764168108e-10**	-0.636370559530001	1.09383087429257e-05	2	Rps8
**7.9759748486303e-10**	-0.865053787857123	1.10156188634433e-05	2	Gm6402
**8.20396138399446e-10**	0.306515825320542	1.13304910674347e-05	2	Bud31
**8.35985256463324e-10**	0.430236788543565	1.1545792377015e-05	2	Raph1
**8.43930822847777e-10**	0.664912838531427	1.16555285943506e-05	2	Dclk1
**9.06991803876058e-10**	-0.383628400318767	1.25264638033322e-05	2	Rps15a-ps6
**9.89352772743899e-10**	1.202608939	1.3663951144366e-05	2	Chil1
**1.01440253674503e-09**	0.506229702194739	1.40099134349856e-05	2	Zfp704
**1.08837101554621e-09**	-0.796359372522945	1.50314920957088e-05	2	Mir684-1
**1.10949473782045e-09**	0.368425658479646	1.53232318240383e-05	2	Htra3
**1.13136088585747e-09**	0.665885320983208	1.56252251945775e-05	2	Nfkb1
**1.1914779006797e-09**	1.765470569	1.64555012862873e-05	2	Chad
**1.41188196714252e-09**	0.488296697431587	1.94995018482053e-05	2	Tcea3
**1.47413050964891e-09**	-0.497321450723625	2.03592164687611e-05	2	Gm12060
**1.49575321709239e-09**	0.514198219183452	2.0657847681263e-05	2	Scara5
**1.52756903457788e-09**	0.545500814454462	2.10972559365551e-05	2	Dio2
**1.55024475334629e-09**	-0.481304154330503	2.14104302884656e-05	2	Dynll1
**1.68316493150135e-09**	0.68142248	2.32461908689652e-05	2	Tmem100
**1.76995974545191e-09**	0.713097075336616	2.44449140444364e-05	2	C2
**1.9149245235507e-09**	0.406766801918891	2.64470225947587e-05	2	Papss2
**1.95695486944753e-09**	0.413038479693937	2.70275037019399e-05	2	Gsta3
**2.05005594540404e-09**	-0.556067885333401	2.83133226619752e-05	2	Rpl37
**2.08652265285991e-09**	0.330736275297583	2.88169643586482e-05	2	Rcn1
**2.08961782803063e-09**	0.567273270258463	2.88597118229311e-05	2	Ankrd17
**2.29725733244647e-09**	0.514141902667653	3.17274210184182e-05	2	Usf2
**2.40794153602712e-09**	0.470839548440083	3.32560805540706e-05	2	Col6a1
**2.52208827679674e-09**	0.414059537179815	3.48325611908398e-05	2	Apod
**2.52885404181837e-09**	0.453730260354037	3.49260031715535e-05	2	Snw1
**2.66767312570947e-09**	0.356875079586895	3.68432335391735e-05	2	Hsd11b1
**2.78803159890232e-09**	-0.398195325836117	3.850550441244E-05	2	Mir703
**2.83742293688486e-09**	0.475654535763574	3.91876481813169e-05	2	Zbed6
**2.8516791878782e-09**	0.731380956582941	3.93845412637858e-05	2	Coq2
**2.87853729778865e-09**	0.564308438715781	3.9755478619759e-05	2	Zeb2
**2.8873839865446e-09**	0.465882339926967	3.98776602381675e-05	2	Nmb
**2.9994684773875e-09**	0.663002956212027	4.14256591411988e-05	2	Unc5b
**3.00838739692947e-09**	0.252590844196817	4.15488383389929e-05	2	Lrg1
**3.22800438175417e-09**	0.295111245112281	4.45819685164069e-05	2	Igf1
**3.35498590392825e-09**	1.386247373	4.6335710319153e-05	2	Slc10a6
**3.41580745125458e-09**	0.455294778809944	4.7175716709277e-05	2	Cd248
**3.71969626657712e-09**	-0.289211732641402	5.13727251376967e-05	2	Jund

**4.08605740835587e-09**	-1.101269704	5.64325388668029e-05	2	Btf3
**4.25588605651658e-09**	0.923930628861607	5.87780423265505e-05	2	Trps1
**4.3375839599285e-09**	0.340929195923738	5.99063720705725e-05	2	Birc3
**4.37349130901252e-09**	0.432377539632536	6.04022884687719e-05	2	Arid4a
**4.51911284330697e-09**	0.432630362518145	6.24134674789126e-05	2	Prelp
**4.71856596072339e-09**	0.420817706133195	6.51681144835508e-05	2	Nop58
**4.8142359371946e-09**	-0.373603679547427	6.64894125285946e-05	2	Rps19-ps3
**4.82582737161647e-09**	0.747061834891181	6.66495018293951e-05	2	LOC1026365
**4.8981603121521e-09**	0.322176765462004	6.76484920711327e-05	2	Nid1
**4.93435244506947e-09**	0.465625671119984	6.81483416188544e-05	2	Plce1
**5.23464935965892e-09**	0.635027602858496	7.22957423062493e-05	2	Lamc3
**5.61437493699924e-09**	0.308696148987568	7.75401322548964e-05	2	Bicc1
**5.87955004433378e-09**	-0.300586002546188	8.12024656622938e-05	2	Gm4832
**6.07530021647554e-09**	0.450902091	8.39059712897437e-05	2	Aff3
**6.83514024510926e-09**	0.376765393846708	9.44001219252039e-05	2	Akap12
**7.04822660985849e-09**	0.514966901	9.73430577087556e-05	2	C1qtnf7
**7.40400632027083e-09**	0.403188270588043	0.00010225673128926	2	Pdk3
**8.6960893032504e-09**	-0.592648003973496	0.000120101689367191	2	Rps16
**8.98204599553854e-09**	0.442452413255801	0.000124051037244383	2	Gbp2
**9.0712517686943e-09**	-0.356483653	0.000125283058177437	2	Rps9
**9.3877982465005e-09**	0.533623222949287	0.000129654881582418	2	Mfap4
**9.40225245493674e-09**	0.302636024310978	0.000129854508655131	2	Zfp655
**1.02162086264801e-08**	0.507644218115443	0.000141096057340317	2	Agpat2
**1.02281540045054e-08**	-0.676789938535173	0.000141261034956224	2	Uba52
**1.03081571022547e-08**	0.441444032286269	0.00014236595773924	2	Sox9
**1.06537995071822e-08**	0.417131778108252	0.000147139624993693	2	Dcn
**1.07391736278729e-08**	0.638197126969652	0.000148318726974553	2	Marcks
**1.1026630390574e-08**	0.347739008824423	0.000152288792324218	2	Tbk1
**1.11435107606705e-08**	0.285154633086025	0.00015390302711562	2	Zfp622
**1.15216978157336e-08**	0.942367044699136	0.000159126168533096	2	Zfp518b
**1.23113949714494e-08**	0.391516161048989	0.000170032675950688	2	Larp4b
**1.250633258948E-08**	0.621549704	0.000172724959393308	2	Dnm1
**1.33461197879854e-08**	0.437721967106782	0.000184323260391867	2	Fbn1
**1.36045258239101e-08**	0.337444767072011	0.000187892106154022	2	Fut10
**1.37492967904546e-08**	0.566516909	0.000189891537972969	2	Ptpn1
**1.40006100403851e-08**	0.709212238506671	0.000193362425267759	2	Mvk
**1.71027705434318e-08**	0.379063344623194	0.000236206363975337	2	Meox2
**1.72312621772133e-08**	0.46064123	0.000237980961929493	2	Stag1
**1.74651839379827e-08**	0.313938069655408	0.000241211655367479	2	Col3a1
**1.76416245630159e-08**	0.440237352409079	0.000243648476839813	2	Med13
**1.76657612064698e-08**	0.338059109942063	0.000243981828022555	2	Tmem176a
**1.85352683850753e-08**	0.354614721690395	0.000255990591666274	2	Crebbp
**1.91256651142954e-08**	0.358102437982364	0.000264144560893533	2	Prkab2
**1.92373165721986e-08**	-0.648938955774184	0.000265686579178635	2	Shfm1
**1.99843640092617e-08**	0.390728474611453	0.000276004051331913	2	Col14a1
**2.08167157665252e-08**	0.357906245261169	0.00028749966145148	2	Aspn
**2.09618058138662e-08**	0.340932977821683	0.000289503500095307	2	Amotl1
**2.10062166552517e-08**	0.454221878278495	0.000290116858225682	2	Sh3d19
**2.2580320347898e-08**	0.514928414017504	0.000311856804324819	2	Medag
**2.40183895147765e-08**	-1.421065323	0.000331717977588578	2	Ppp1r14a
**2.59890373294888e-08**	0.501987836775123	0.00035893459455757	2	Thy1
**2.78105457114048e-08**	0.467285316363916	0.000384091446820212	2	Col5a2
**2.95545257570369e-08**	-0.518510765600686	0.000408177555230436	2	Myl12b
**2.98568447655625e-08**	0.423398946763267	0.000412352883057184	2	Tcn2
**3.02832254744056e-08**	0.448818351642806	0.000418241627027016	2	Chpf
**3.03551635333892e-08**	0.340699122793932	0.000419235163559638	2	Abca8a

**3.12396706448617e-08**	0.447275041106438	0.000431451091276185	2	Rhbdf1
**3.17705658797437e-08**	0.784018156561819	0.00043878328536514	2	Lpin3
**3.30930203390021e-08**	0.398324378865632	0.000457047703901957	2	Abi3bp
**3.3916527703667e-08**	0.280386813497201	0.000468421164115345	2	Mycbp2
**3.55111420531037e-08**	0.416499428262273	0.000490444382895415	2	AI507597
**3.65969273409913e-08**	0.287660492446816	0.000505440163506431	2	Igfbp6
**3.70383823317981e-08**	-0.355971581107926	0.000511537098384463	2	Rpl18a
**3.74100025093247e-08**	0.498434942964715	0.000516669544656283	2	B4galt1
**4.07420924545628e-08**	0.699961630196051	0.000562689038889967	2	Cd55
**4.09784533116565e-08**	0.605049395672021	0.000565953418687288	2	Jade1
**4.17673717807325e-08**	-0.412685401075206	0.000576849171663696	2	S100a11
**4.26443847685045e-08**	0.78029184	0.000588961598037816	2	Trib1
**4.68692444638419e-08**	0.588555034826504	0.00064731113529012	2	Rbbp6
**4.74636095628032e-08**	-0.343583803594784	0.000655519911671875	2	Rps15a
**4.86390179783711e-08**	0.635741337483905	0.000671753477299283	2	Gm20199
**4.96469003865231e-08**	0.770282086105315	0.00068567334123827	2	Cul7
**5.45951720826393e-08**	-0.932430546912166	0.000754013921633331	2	Rpl36al
**5.47919670750536e-08**	1.109541828	0.000756731857273566	2	Bmper
**5.50900051630788e-08**	0.417698632949075	0.000760848061307282	2	Pcolce
**5.63535253422301e-08**	0.387578976543142	0.00077829853850154	2	Specc1l
**5.97154380877016e-08**	1.166653189	0.000824729915429247	2	Tbx20
**6.28521635969488e-08**	0.449519920919268	0.000868051231437459	2	Lztr1
**7.27417171172743e-08**	0.402391946926106	0.00100463585510668	2	Hk2
**7.46780821049495e-08**	0.442031696432567	0.00103137899195146	2	Socs2
**8.14107275149132e-08**	0.372240042661539	0.00112436355770847	2	Lrp1
**8.14271529242893e-08**	1.122845438	0.00112459040903736	2	Map3k4
**8.28351648079229e-08**	0.828504790152377	0.00114403646116222	2	Ccl19
**8.65551549863795e-08**	0.301235611	0.00119541324551689	2	Tspan4
**8.84224410361598e-08**	0.595874184	0.0012212023331504	2	Tcf21
**8.89504198027251e-08**	0.540244690431145	0.00122849424789544	2	Fech
**9.12960630895415e-08**	0.273988478459543	0.00126088992732966	2	Gorasp2
**9.4864828856899e-08**	0.398598056398099	0.00131017815134263	2	Fnip1
**9.58325935750605e-08**	-0.537105857422408	0.00132354394986516	2	Psme2
**1.00824463302123e-07**	1.707885545	0.00139248666266562	2	Saa3
**1.04842701727194e-07**	-0.505247515996046	0.00144798255355427	2	Gng5
**1.05319423266489e-07**	0.724553047879833	0.00145456655473348	2	Asap3
**1.1198960528197e-07**	-0.657002985097349	0.00154668843854928	2	Psenen
**1.13501961783652e-07**	0.674705070459683	0.00156757559419401	2	Cdk14
**1.16591912086288e-07**	-0.728512386780966	0.00161025089782372	2	Atp5j2
**1.26472994224231e-07**	0.476058068131966	0.00174671852323086	2	Nfkbia
**1.2778326536593e-07**	0.395349042	0.00176481467796886	2	Cdc42ep1
**1.29839006422408e-07**	-0.899849993001341	0.00179320651769987	2	Atp5l
**1.31065701783472e-07**	0.323694258830219	0.00181014840733154	2	Ccl11
**1.37158719331926e-07**	0.382603657179634	0.00189429907269323	2	Entpd2
**1.47834449629854e-07**	-0.463069217120479	0.00204174158383792	2	Ndufb4
**1.52039953259E-07**	0.310651543418239	0.00209982379446005	2	Lsp1
**1.87113001526503e-07**	-0.865510772892046	0.00258421766408253	2	Ndufa4
**1.95680196998984e-07**	0.457064693126552	0.00270253920075296	2	Pak1ip1
**1.96604343361999e-07**	-0.547062066429053	0.00271530258617257	2	Rpl35
**2.12243240684533e-07**	0.370207832595042	0.00293129139709409	2	Ecm1
**2.30583842770511e-07**	0.445653265125827	0.00318459345250353	2	Slfn8
**2.43316689659473e-07**	-0.442752805103813	0.00336044680088698	2	Rpl18
**2.64878406490191e-07**	0.504040259604956	0.00365823567203603	2	Mpp5
**2.70362416582765e-07**	0.873617390445324	0.00373397533542456	2	Nrip1
**2.77568482160073e-07**	0.368895876968258	0.00383349830711277	2	Zdhhc5
**2.7909907108936e-07**	0.395710945885978	0.00385463727081515	2	Adamts10

**2.87552200416643e-07**	0.486319575406282	0.00397138343995426	2	Sqle
**2.99039420062953e-07**	0.416241807758622	0.00413003343048944	2	Rin2
**3.07384079384932e-07**	0.654293084322285	0.0042452815203853	2	Epb4.1l5
**3.10621016573993e-07**	0.340902596858736	0.00428998685990341	2	Slc5a3
**3.12163344532633e-07**	-0.393305872413869	0.00431128795134019	2	Rplp2
**3.15972438275908e-07**	0.365615040942957	0.00436389534502856	2	Ankhd1
**3.40585174226011e-07**	0.362012753738039	0.00470382184123544	2	Fxyd6
**3.55748976888222e-07**	0.364509416022932	0.00491324911980323	2	Brwd1
**3.59188905864196e-07**	0.808049860752433	0.00496075797889042	2	Hhip
**3.60187502776882e-07**	0.401430170846787	0.00497454960085152	2	Mbd3
**3.66809342227321e-07**	0.259166987	0.00506600382550154	2	Ube2j1
**3.83946585374495e-07**	-0.355446479785553	0.00530268629060716	2	Rpl7
**3.91382275503797e-07**	0.491662535938844	0.00540538060698294	2	Lrig1
**4.04893688203025e-07**	0.491909258	0.00559198672777197	2	Sntb2
**4.08940760505384e-07**	-0.303151833524028	0.00564788084333986	2	Cox4i1
**4.1740993071777e-07**	0.264634218544101	0.00576484855314312	2	Sfrp1
**4.18743400518084e-07**	0.620507290571747	0.00578326510455526	2	Fdft1
**4.27524204279366e-07**	0.323131430711069	0.00590453678530233	2	Ptprd
**4.28249611718932e-07**	0.361393720389014	0.00591455538745017	2	Gas6
**4.33346638329579e-07**	0.391795699513809	0.00598495042196982	2	Haus7
**4.69137954805108e-07**	0.366056314760516	0.00647926429381335	2	Vps26b
**4.78211110838645e-07**	1.373730233	0.00660457365179252	2	Kifc3
**4.7964310647332e-07**	0.381440294370052	0.00662435094350302	2	Pdgfra
**4.84130801914518e-07**	0.261832112898756	0.0066863305052414	2	Lbp
**4.93875153295467e-07**	0.282291862451555	0.00682090974216369	2	Ppic
**5.00846119119935e-07**	0.360047372338999	0.00691718575116542	2	Csnk1d
**5.13494183395817e-07**	0.343513402442918	0.00709186816687963	2	Hivep2
**5.41010102705185e-07**	0.578542228730289	0.00747189052846131	2	Bdnf
**5.58820752819657e-07**	0.685879133466421	0.00771787341719228	2	Cdk5rap2
**5.7736542212678e-07**	0.487692477212515	0.00797399384499295	2	Smad5
**5.83988917116274e-07**	0.332593734500659	0.00806547093429285	2	Mfap2
**5.89987570146206e-07**	0.423380849836124	0.00814831833128926	2	Ubr4
**5.91792734701054e-07**	-0.830719195721062	0.00817324945895626	2	Bcam
**6.07700905219391e-07**	0.252780851764932	0.00839295720198501	2	NP-904339.1
**6.20710750192487e-07**	1.117303827	0.00857263617090844	2	Cyp26b1
**6.26958725693315e-07**	0.547916749503719	0.00865892696055037	2	Aox3
**6.30945096499121e-07**	-0.340188332669701	0.00871398272774936	2	Rpl14
**6.64979523694489e-07**	0.610342588643055	0.00918403220174459	2	Birc2
**6.65070943189143e-07**	0.656960080712271	0.00918529479638525	2	Tsc2
**7.11705838408751e-07**	0.458301595219055	0.00982936933426326	2	Enpp2
**7.19911789541126e-07**	-0.722226910625494	0.00994270172535249	2	Ywhah
**7.21684038852626e-07**	0.353519371739923	0.00996717826059362	2	Cars
**7.3139625452319e-07**	-0.567411739690735	0.0101013136712198	2	Gm6548
**7.57887805123059e-07**	0.345565453	0.0104671884765546	2	Serping1
**7.67393303910284e-07**	0.353025422606639	0.0105984689203049	2	Arid1b
**7.73558354746686e-07**	0.533513012571896	0.0106836144374065	2	Adamts15
**7.76183978908497e-07**	0.552700153639413	0.0107198769327053	2	Ehmt2
**7.77635583764921e-07**	0.531408264952155	0.0107399250473773	2	Trip12
**7.82329909794444e-07**	0.659266451589342	0.0108047583841711	2	Ptgfr
**7.94386865706566e-07**	0.421894643644625	0.0109712770022734	2	Fgl2
**7.99510830965288e-07**	0.254418459	0.0110420440864616	2	Abca1
**8.19135789579866e-07**	0.277585719957946	0.0113130843898875	2	Jmjd1c
**8.60206715467597e-07**	0.368045731014714	0.011880314947323	2	Nnmt
**8.64335195603004e-07**	0.342788617333346	0.0119373333864731	2	Fcgrt
**8.6995969579295e-07**	-0.292566970990324	0.0120150133585964	2	Rpl6
**8.81735465741657e-07**	0.365256008275676	0.012177648517358	2	Figf

**9.04594222277562e-07**	0.325777750839529	0.0124933508038754	2	Por
**9.20648252556574e-07**	0.482586024998333	0.0127150730160588	2	Angpt1
**9.27387342313348e-07**	0.876586604407865	0.0128081465846896	2	S100b
**9.66121036778799e-07**	-0.546532170296938	0.013343097638952	2	Rpl29
**1.00232604065865e-06**	-0.711305740844419	0.0138431249475366	2	Eif4a1
**1.06667922553626e-06**	-0.257177827135689	0.0147319067838813	2	Gm13826
**1.08735164009889e-06**	0.318211672638264	0.0150174135014057	2	Pde4b
**1.11852300479755e-06**	0.252613206431476	0.015447921219259	2	Tmem205
**1.12057848569095e-06**	-0.541427172573548	0.0154763094658777	2	Rps25
**1.20572774829713e-06**	0.855995255270582	0.0166523059317317	2	Senp1
**1.21309054335625e-06**	0.266027548	0.0167539934942931	2	C1s1
**1.26440154835872e-06**	0.337648786646939	0.0174626497843822	2	Phip
**1.31702208743702e-06**	0.406418228072561	0.0181893920495927	2	Vipr2
**1.3518088523155e-06**	0.636249081702594	0.0186698320593294	2	Negr1
**1.39258818738601e-06**	0.407731773033682	0.0192330354559882	2	Clmp
**1.48214934110546e-06**	0.408620379620449	0.0204699645500075	2	Sil1
**1.48871420622751e-06**	-0.411448595357998	0.0205606319022081	2	Pcnp
**1.54462403579109e-06**	0.450304478065315	0.0213328025583107	2	Ptp4a1
**1.5737796297285e-06**	0.321735332255428	0.0217354704661803	2	Pxdn
**1.74564517484555e-06**	0.984661555350068	0.0241091055097919	2	Pdxk
**1.78772423830281e-06**	0.370857898902195	0.0246902594552001	2	Erlec1
**1.79521257488363e-06**	-0.403329859812628	0.0247936808717178	2	Gstp1
**1.81639592440682e-06**	-0.296130017225424	0.0250862441119826	2	Rpl13
**1.82195697715493e-06**	0.423976408565633	0.0251630478114868	2	Ier3
**1.82471334793057e-06**	0.361538510770808	0.0252011160482692	2	Smc4
**1.85871380838848e-06**	0.275114666070625	0.0256706964076533	2	Sepn1
**1.86365154190299e-06**	0.437663158	0.0257388914452222	2	Ntrk2
**1.88362928314792e-06**	0.342093083086779	0.026014804029556	2	Cdk5rap3
**1.89283733793795e-06**	0.423307012243818	0.026141976474261	2	Hook3
**1.89708918806219e-06**	0.848475050599147	0.026200698776327	2	3632451O06I
**2.0095295011042e-06**	0.314272115434109	0.0277536119397501	2	Ift27
**2.02797259996676e-06**	0.496376385388383	0.028008329578141	2	Pi16
**2.06326204712922e-06**	-0.338705178382525	0.0284957121329016	2	Eif5a
**2.07247041415443e-06**	0.354378883843618	0.0286228888898869	2	Crem
**2.07373603049177e-06**	0.602405287412703	0.0286403683171218	2	Csrnp1
**2.13441217779557e-06**	1.188695011	0.0294783665875346	2	Dusp8
**2.19098823969221e-06**	0.444611104363114	0.0302597385783891	2	Sin3b
**2.22347130983406e-06**	0.466947504011389	0.0307083622601181	2	Srgap2
**2.23475229337589e-06**	-0.585142833983993	0.0308641639238145	2	Tma7
**2.25731541706391e-06**	-1.331785353	0.0311757832250697	2	Spint2
**2.27931376047347e-06**	0.702036127977191	0.0314796023458991	2	Sphk1
**2.28720000780947e-06**	0.582243572871441	0.0315885193078566	2	Jkamp
**2.30545294465303e-06**	0.491083511032602	0.0318406106186031	2	Zfc3h1
**2.31224015356961e-06**	0.462205367154558	0.0319343487609498	2	Gna13
**2.31893057805284e-06**	0.331451485368131	0.0320267502134878	2	Dnajc25
**2.34976760249373e-06**	0.341898323305894	0.0324526403580409	2	Adm
**2.36072490234351e-06**	0.26739186	0.0326039716262663	2	Gba
**2.37756392116482e-06**	0.677814678646269	0.0328365353152074	2	Sp1
**2.38550905956757e-06**	0.337049696019786	0.0329462656216876	2	Stau1
**2.68443902019431e-06**	0.255296809483363	0.0370747873079036	2	Pagr1a
**2.7086416817102e-06**	0.270502127898727	0.0374090502660996	2	Pofut1
**2.71420169178488e-06**	-1.095299021	0.037485839565241	2	Gpsm2
**2.76450910379131e-06**	-0.397637168748013	0.0381806352324618	2	Atp5h
**2.7736992234144e-06**	0.451828084411323	0.0383075599745762	2	Ddx46
**2.8097858235282e-06**	0.543733375	0.038805952008748	2	Prickle1
**2.8128658837721e-06**	0.397017740523605	0.0388484907207765	2	Clcn4-2

**2.82055536765923e-06**	0.291815231854187	0.0389546901827416	2	Herc1
**2.8416696026682e-06**	-0.318281814	0.0392462988824505	2	Tceb2
**2.84230059349294e-06**	0.913674442807482	0.039255013496731	2	Col27a1
**2.88477619390696e-06**	0.681620943234599	0.039841644014049	2	Ccbe1
**2.95176844659899e-06**	-0.413715548911996	0.0407668740159786	2	Ptms
**3.08380511093437e-06**	0.280024175596967	0.0425904323871146	2	Ythdc1
**3.20894950838703e-06**	0.587547059975725	0.0443188016603332	2	Papola
**3.25056881991403e-06**	0.605592705336387	0.0448936059718327	2	Gm16515
**3.35705183776403e-06**	0.488215312079287	0.046364242931359	2	Slc39a14
**3.44925251436569e-06**	0.269378483495638	0.0476376264759046	2	Tmem204
**3.66671807269495e-06**	-1.18097917	0.0506410433019899	2	Gja5
**3.70274955049037e-06**	-0.333397259069387	0.0511386740418225	2	Pebp1
**3.7115332985268e-06**	0.453826997036192	0.0512599863859537	2	Piezo2
**3.72337275397151e-06**	-0.331580584662721	0.0514235011051005	2	Naca
**3.80583200900993e-06**	-0.936317832553292	0.0525623458764362	2	Usmg5
**3.82428607278326e-06**	0.590460725134414	0.0528172149512096	2	Loxl2
**3.86598335023549e-06**	0.304678088053048	0.0533930960501024	2	Aprt
**3.96683197796653e-06**	0.492332097147749	0.0547859164476958	2	Mpzl1
**4.03004942339295e-06**	-0.491726541277919	0.055659013	2	Rpl22
**4.04661990907557e-06**	0.644162365847463	0.0558878675642427	2	Abhd11os
**4.07069001597115e-06**	0.267892176473948	0.0562202998105775	2	Kcnj8
**4.08828855717219e-06**	0.477674157	0.0564633532631051	2	Epn1
**4.11886507924203e-06**	0.715247702256276	0.0568856456094117	2	Pja1
**4.29790554109483e-06**	-0.760105290197194	0.0593583734280607	2	Pabpc4
**4.3368112001277e-06**	0.574734033100884	0.0598956994849637	2	Pgrmc2
**4.40692914252565e-06**	0.269680619935767	0.0608640983874217	2	Ccnl2
**4.44827031242053e-06**	0.271868366059451	0.0614350612848399	2	Cdyl
**4.47043405078556e-06**	0.310620716802537	0.0617411646753994	2	Sash1
**4.54445485596578e-06**	0.769247771	0.0627634660157435	2	Ptpn23
**4.57716569903586e-06**	0.473733079237167	0.0632152354693842	2	Zfp386
**4.59845519503472e-06**	-0.643471668833789	0.0635092646986246	2	Uqcrb
**4.81995875207311e-06**	-0.309253856953394	0.0665684503248817	2	S100a6
**5.39270709337151e-06**	0.713603989087649	0.0744786776665539	2	Maob
**5.63981772427046e-06**	-0.600584881163773	0.0778915225898993	2	Tomm6
**5.85861396403332e-06**	0.540840649491848	0.0809133174572642	2	Sdc2
**5.89139537229924e-06**	-0.302858852255421	0.0813660614868248	2	Eif1
**5.94125992244014e-06**	0.825444555713009	0.0820547407888208	2	S1pr2
**6.04211606852807e-06**	0.300919062856178	0.0834476650224412	2	Litaf
**6.10067953600942e-06**	0.328345429774304	0.0842564850718261	2	Ankrd11
**6.16032187264358e-06**	0.310514223787359	0.0850802053830805	2	Pdcd11
**6.4626709133836e-06**	0.645040220498869	0.0892559479847409	2	Cnst
**6.56535499838797e-06**	0.327249306	0.0906741178827362	2	Ptk2
**6.57293835397115e-06**	0.283462507863904	0.0907788516066955	2	Kdelr3
**6.99152333212367e-06**	-0.909949556535708	0.096559929	2	Psmb3
**7.28536124609878e-06**	0.344205356201887	0.100618124	2	Heph
**7.29755857396192e-06**	-1.388277038	0.100786581464988	2	Nos3
**7.44323868808415e-06**	-0.447721715582568	0.10279857	2	Snrpd2
**7.44785344912383e-06**	0.440086820172926	0.102862303985849	2	Tmem260
**7.51102046275999e-06**	0.307059127181823	0.103734703611178	2	Nrd1
**7.68018135262425e-06**	0.250137852761672	0.106070984661093	2	Gfpt2
**7.70930136081052e-06**	0.315914781150674	0.106473161094154	2	Grina
**7.71233387625161e-06**	0.640166092152125	0.106515043164911	2	Abhd5
**7.85636124295511e-06**	0.446315092085169	0.108504205126453	2	Rftn1
**8.17315115193469e-06**	0.395969131640083	0.112879391	2	Itgbl1
**8.23588111188327e-06**	0.353678524826319	0.113745754	2	Pltp
**8.48731839148203e-06**	0.420691877313459	0.117218354304758	2	Fundc1

**9.05968908236551e-06**	-0.479988054088705	0.125123366	2	Ost4
**9.07267287751788e-06**	0.473642812511006	0.125302685111399	2	Pnn
**9.08039326213997e-06**	0.298328238195774	0.125409311343415	2	Fmo1
**9.2877198934606e-06**	0.509948366600293	0.128272699448584	2	Ahsg
**9.91669385971244e-06**	0.346423225026859	0.136959458896489	2	Lpar1
**1.00884690964362e-05**	0.615171131724118	0.139331847	2	Nid2
**1.02726017075211e-05**	0.444211260033853	0.141874902182573	2	Thbs2
**1.05155245907062e-05**	0.338221129904982	0.145229910122243	2	Alcam
**1.08054255589378e-05**	0.714499937019706	0.149233732394491	2	Rgs10
**1.10407314509099e-05**	1.307479226	0.152483542068517	2	Dcaf17
**1.10835800400223e-05**	-0.297436492943333	0.153075323932748	2	Nme2
**1.12731788347464e-05**	-1.034262735	0.155693872886682	2	Txnip
**1.13174701411903e-05**	-0.484443952959054	0.15630558	2	Psma5
**1.18216489754793e-05**	0.264076171422276	0.163268794000345	2	Smpd1
**1.18587119502809e-05**	0.406450475129192	0.163780671	2	Kmt2e
**1.23836254514388e-05**	0.579561785968018	0.171030251109821	2	Grpel2
**1.2408933024639e-05**	0.549408268348367	0.171379774003289	2	Tgfbrap1
**1.25033780338064e-05**	0.717766247253493	0.172684154024901	2	G6pdx
**1.25723155066583e-05**	0.479900066	0.173636249462458	2	Wwp2
**1.27466868929994e-05**	1.165361845	0.176044492679214	2	Naa25
**1.2846516300933e-05**	0.286772531707123	0.177423236632186	2	Tubb6
**1.30840056210099e-05**	-0.359942643565192	0.180703201631768	2	Rpl13a
**1.31219942917408e-05**	-0.327864741499661	0.181227863163232	2	Gas5
**1.32813763192555e-05**	0.314743300775778	0.183429088345238	2	Fn1
**1.3500732245282e-05**	0.568725648889172	0.186458613039589	2	Fmnl2
**1.36325767941929e-05**	0.284783318231641	0.188279518104599	2	Kmt2c
**1.38689801229245e-05**	0.282065940062287	0.191544484	2	Man2b1
**1.38730665064203e-05**	-0.405887594	0.191600921520171	2	Dstn
**1.38941001089685e-05**	0.496297907654507	0.191891416604964	2	Trim2
**1.47480764856665e-05**	0.610673801045547	0.203685684	2	Sele
**1.48263031524199e-05**	0.508308919318718	0.204766072838071	2	Man2a2
**1.5143859701216e-05**	0.513005819	0.209151846333494	2	Ptcd2
**1.59216882138281e-05**	0.251466466594854	0.219894436	2	Chrdl1
**1.61244202392479e-05**	-0.560480408387677	0.222694367924253	2	Mpv17l2
**1.64474474357773e-05**	0.501511813251597	0.227155697	2	Idnk
**1.66734992012794e-05**	-0.393844819223635	0.230277697	2	Arf1
**1.68842337213979e-05**	0.306226562745246	0.233188151926227	2	Muc13
**1.78761671830417e-05**	0.331119910541744	0.246887744964989	2	Ccrn4l
**1.80308772898237e-05**	0.717308995355737	0.249024446249755	2	Rasa1
**1.80976401787807e-05**	-0.260142359450124	0.249946509	2	Aldoart1
**1.83450057211719e-05**	0.536502085582755	0.253362874015106	2	Polr2e
**1.8391936463783e-05**	0.315571390920027	0.254011034501307	2	Elmod2
**1.93461177320847e-05**	0.794787217432325	0.267189231997822	2	D10Jhu81e
**1.93688740652875e-05**	0.440083861741841	0.267503519715686	2	Smarcd2
**1.94831127506723e-05**	0.508082317560783	0.269081270199536	2	Dctn3
**1.96111924726171e-05**	0.330241686870577	0.270850179239315	2	Eif2ak4
**1.96700585086235e-05**	0.290373981621218	0.271663178062599	2	Egr1
**1.97386878249995e-05**	1.123757388	0.272611017551067	2	Pappa
**1.97648677547123e-05**	0.298135495	0.272972588560332	2	Secisbp2l
**2.00424932126144e-05**	0.295576219877423	0.276806873759417	2	Arl5b
**2.16596715057182e-05**	0.437037449038595	0.299141723165475	2	Rif1
**2.16805812540766e-05**	0.385096357750999	0.299430507700051	2	Jak1
**2.17052016859934e-05**	0.380393510363251	0.299770540485255	2	Bhlhe40
**2.17320457550509e-05**	0.286039611090994	0.300141283923009	2	Fxyd5
**2.19091660771855e-05**	0.465021976800678	0.302587492692009	2	Bclaf1
**2.23320133241255e-05**	0.878334979055095	0.308427436019498	2	Kctd17

**2.23340484833996e-05**	0.616990968441104	0.308455543604232	2	Cdon
**2.28050422343756e-05**	0.258133781	0.314960438298961	2	Prpf40a
**2.30688451025525e-05**	0.489844885	0.318603819711353	2	Sobp
**2.33213777029973e-05**	-0.552687241067607	0.322091547456096	2	1190005I06R
**2.37643045328335e-05**	0.854193942884663	0.328208809902964	2	Ctr9
**2.4410119411701e-05**	0.556329765743247	0.337128159195002	2	Tom1l2
**2.44898010054293e-05**	0.591402458525056	0.338228641685984	2	Lsamp
**2.48581401144287e-05**	-0.262251683719964	0.343315773120374	2	Scamp2
**2.63050166444128e-05**	0.311585050447644	0.363298584875985	2	Yars
**2.63736592044495e-05**	0.428585770130363	0.364246607272653	2	Slk
**2.66797781935733e-05**	0.829145054184347	0.368474417	2	Npy1r
**2.67889793660996e-05**	0.280290134	0.369982594025202	2	Smurf2
**2.67955328054222e-05**	0.406521089044703	0.370073103575685	2	Sqstm1
**2.73822214586376e-05**	0.453793542607963	0.378175860565243	2	Gm15417
**2.88033493833732e-05**	0.811614628591891	0.397803058333767	2	Ift22
**2.94106304676868e-05**	0.419999195571421	0.406190217389223	2	Traf3ip2
**3.05759437401988e-05**	-0.477352033713578	0.422284358995886	2	Atp5j
**3.0746081285365e-05**	-0.377951410853213	0.424634128632176	2	Rpl10
**3.21732207198654e-05**	-0.318541828032334	0.444344351362061	2	Serinc3
**3.22829437332914e-05**	-1.080091101	0.445859735900487	2	Snrpf
**3.31969393830367e-05**	0.377011138051328	0.45848293	2	Atp6v1b2
**3.34429954642466e-05**	-0.274589412640801	0.461881210356709	2	Sec61g
**3.42646701405376e-05**	0.511077813918702	0.473229359310965	2	Wbp4
**3.5292190824861e-05**	0.305907647	0.487420447482156	2	Cyp2j9
**3.55484037370544e-05**	0.413394649197576	0.490959004012459	2	Alpl
**3.5787150630852e-05**	0.276641294195187	0.494256337362696	2	Arfgef1
**3.5958825156565e-05**	0.687675452143305	0.496627334237319	2	Cipc
**3.6040424457005e-05**	0.470206375566138	0.497754302175695	2	Mpp6
**3.6629528807776e-05**	0.730509605	0.505890422364194	2	Stk32b
**3.68453072970453e-05**	0.370061963787353	0.508870539079493	2	Tex264
**3.70086522722124e-05**	0.338484430711217	0.511126496531525	2	Stard3
**3.72460739673416e-05**	0.380327102407305	0.514405527562954	2	Kidins220
**3.76454088838206e-05**	0.378360346356735	0.519920742094446	2	Iffo2
**3.78413943750569e-05**	0.383997301516328	0.522627498	2	Smc6
**3.78766719201872e-05**	-0.835467561358209	0.523114715889706	2	E030013I19R
**3.79494879010018e-05**	0.505135814	0.524120377400735	2	Bahd1
**3.80108456909922e-05**	0.479858294	0.524967789838294	2	Prss12
**3.82180967300216e-05**	0.272129211944937	0.527830133938328	2	Rbms1
**3.85244922622408e-05**	-0.658345009838646	0.532061762633808	2	Tcea1
**3.87927102362639e-05**	0.414374324905472	0.535766121	2	Zranb2
**3.88061836691856e-05**	0.570585761847991	0.535952202655123	2	Ivns1abp
**3.89367617235098e-05**	-0.364129643986317	0.537755616163394	2	Selk
**4.07766810498749e-05**	0.482754901128094	0.563166741979822	2	Bin3
**4.3093205200512e-05**	-0.781460561099062	0.595160257017907	2	Khsrp
**4.31859185183234e-05**	0.625823008516753	0.596440720656565	2	Slc25a38
**4.36946969799577e-05**	-0.489373756	0.603467459990196	2	Atp5g1
**4.40886398933535e-05**	0.601282381487486	0.608908205567105	2	Map2k7
**4.47284354557873e-05**	0.259582086852457	0.617744422079878	2	Sfrp4
**4.59432839971989e-05**	0.359471770267218	0.634522695285314	2	Aco2
**4.6060093733851e-05**	0.279992154693718	0.636135954558216	2	Ttc1
**4.63097599859481e-05**	0.936660210744255	0.639584095165929	2	Klhl17
**4.64051347311629e-05**	0.487346578595267	0.640901315772091	2	Sertad4
**4.76889675810893e-05**	0.828516998087077	0.658632331262425	2	Arhgef5
**4.80523676491087e-05**	0.411957376285496	0.66365125	2	Pcdh18
**4.87051315796162e-05**	0.288891759774193	0.672666572	2	Tnrc6a
**4.87227475032779e-05**	-0.471875526489013	0.672909865767772	2	Polr2l

**4.87885697334691e-05**	0.285394538848387	0.673818936588942	2	Zbtb20
**5.06793430433861e-05**	0.305834826180871	0.699932406772206	2	Cc2d2a
**5.1500645410461e-05**	-0.430795122304195	0.711275413763877	2	Ybx1
**5.16542909264549e-05**	0.404285231773065	0.713397411985269	2	Phlda1
**5.18163556512561e-05**	-0.325566066339813	0.715635687899498	2	Apoa1bp
**5.19114846441087e-05**	-0.720032836926867	0.716949514419785	2	Camta1
**5.27922884124846e-05**	-0.824959583759794	0.729114295264824	2	Pdcd5
**5.43974242611228e-05**	-0.357204255865882	0.751282826470367	2	Sat1
**5.58494991684465e-05**	0.547939726652945	0.771337433015414	2	Lcorl
**5.69487449060123e-05**	1.027212283	0.786519115896935	2	Ttbk2
**5.77254819641306e-05**	0.407961549340701	0.797246631406608	2	Tmem140
**5.7750126193593e-05**	-0.446909253386541	0.797586992859713	2	Dynlrb1
**5.79636162550606e-05**	1.227522561	0.800535504098642	2	Bag4
**5.85033873602621e-05**	-0.527508459535316	0.807990283	2	H2-K1
**5.91455525211292e-05**	0.470162383146744	0.816859225869316	2	Fam214b
**5.9509929379813e-05**	-0.561159756239891	0.821891634664598	2	Rpl38
**6.09086493694066e-05**	0.465838106818478	0.841209356440875	2	Rspo3
**6.2877557929564e-05**	0.472081246964777	0.868401952565208	2	Taf3
**6.33243107772438e-05**	0.376055381537173	0.874572056144514	2	Fam21
**6.51066704495927e-05**	-0.369820591608169	0.899188225579325	2	Uqcr11
**7.05395542885284e-05**	0.445731373344804	0.974221784278865	2	Rps4l
**7.08154256504962e-05**	0.426596206	0.978031843659004	2	Ets2
**7.31428598585082e-05**	0.388533230853656	1	2	Prdm2
**7.37102477504276e-05**	0.371521926727479	1	2	Prrx1
**7.4819244044514e-05**	0.253749874896082	1	2	Rela
**7.57030635162398e-05**	0.275179464385891	1	2	Rab3d
**7.72860323424123e-05**	0.537122504372839	1	2	Cxcl5
**7.81171497946002e-05**	0.285496243192567	1	2	Il6st
**7.820568881559E-05**	0.696453666389226	1	2	Gas7
**7.8494308063374e-05**	0.505488994	1	2	Myoc
**7.97276905684759e-05**	0.289561015428406	1	2	Gstm5
**8.21854174967423e-05**	-0.777311177552251	1	2	Perp
**8.28975639986744e-05**	0.576698796850122	1	2	Kdm7a
**8.42299705699892e-05**	0.316657778435764	1	2	Lrrc28
**8.78918493330226e-05**	-0.394376978763404	1	2	Npm1
**8.87156153330886e-05**	0.273100661924211	1	2	Rnase4
**9.00239033220301e-05**	0.324556124150094	1	2	Ppfibp1
**9.01363667688707e-05**	-0.466266214	1	2	Skp1a
**9.07509757546479e-05**	0.548147394201022	1	2	Slit2
**9.14021145687309e-05**	0.664116743193468	1	2	Nfasc
**9.19560629607086e-05**	-0.953637668821244	1	2	Rps29
**9.20328416696106e-05**	0.323182057076748	1	2	Rora
**9.24060412182084e-05**	0.480887662060118	1	2	Suv420h1
**9.2537042699805e-05**	0.258401349175713	1	2	B9d1
**9.28215743568962e-05**	0.281905900853726	1	2	Malsu1
**9.46281482858143e-05**	0.399868068170756	1	2	Fam129b
**9.66750512342727e-05**	0.409418683651975	1	2	Tardbp
**9.77132875599827e-05**	-0.986625284423978	1	2	4930503E24F
**0.000100067960200744**	0.610438293280723	1	2	Cdc42ep4
**0.000100571497372697**	0.785547458231799	1	2	Ppp4r1
**0.000100953820449452**	1.147274557	1	2	Rint1
**0.000101922884507369**	0.880969562384906	1	2	Map3k3
**0.000102094667019252**	0.469458333520585	1	2	Arhgef40
**0.000103135892995434**	0.361917741438754	1	2	Rbbp8
**0.0001031908984396**	-1.105304273	1	2	Tmem38b
**0.000106208054828566**	0.782979985404303	1	2	Cep162

**0.000106785035943019**	-0.994694929781827	1	2	Ppp4c
**0.000109651941352726**	0.665652540055868	1	2	Eps8
**0.000110278272221529**	0.392317198701659	1	2	Akap8
**0.000111197341231767**	0.290596924859306	1	2	Fto
**0.000112843850435899**	-0.866190127	1	2	Mylk4
**0.000113161800899708**	0.896205124794573	1	2	Gnl3l
**0.00011498306476465**	0.602913137540758	1	2	Cherp
**0.000123277758000704**	0.559951903562362	1	2	Gcdh
**0.0001248658986693**	0.281157158050482	1	2	Usp33
**0.000129534042082452**	1.309876233	1	2	Rnf8
**0.0001302881506501**	0.439214092844805	1	2	AI607873
**0.000130363587820113**	0.390261665646648	1	2	Tmem248
**0.000131053251581053**	0.391981606320357	1	2	Ppp1r18
**0.00013212919441347**	0.257976449082634	1	2	Myo10
**0.000133643351193168**	0.354898092540524	1	2	Lonrf3
**0.000134250309103**	0.486850928092715	1	2	Lmo4
**0.00013459513104181**	0.486596797246358	1	2	Il6ra
**0.000135384331076787**	0.330318039	1	2	Man1a
**0.000135509988797123**	0.255552502349489	1	2	Pkd2
**0.000135818237974451**	0.444359103623607	1	2	Add1
**0.000136744590706199**	0.390308655638249	1	2	Acsl4
**0.000137762766508039**	-0.288396823242529	1	2	Crip1
**0.000139499168400549**	0.518574955543467	1	2	Fam63b
**0.000140411622363693**	-0.320828956557463	1	2	Fkbp1a
**0.00014270298317612**	0.559483691048997	1	2	Chordc1
**0.000144229731604265**	-0.463854826639357	1	2	Atp5k
**0.000145091020125907**	0.330903746445993	1	2	Sdc3
**0.000146215881507068**	0.830591518333131	1	2	Pih1d1
**0.000149022372362214**	0.533301030906386	1	2	Trappc1
**0.000149918306244653**	0.531224855777811	1	2	Smim3
**0.000151381527879371**	0.456966153739847	1	2	Svep1
**0.00015197783895563**	0.275584669063003	1	2	Tnrc6c
**0.000152178663097509**	0.338799745849355	1	2	Tmem33
**0.000161676960475235**	0.408245474	1	2	Coro1b
**0.000162124603683906**	0.522458048	1	2	Cilp
**0.000162953213280713**	0.493051138	1	2	Zmym2
**0.000166117836629968**	0.292203037640563	1	2	Phlpp2
**0.000169183813123845**	0.425771022588353	1	2	Setd7
**0.000169420788344987**	-0.368368839911466	1	2	Eno1b
**0.000171085548356115**	0.260984380814384	1	2	Rabl6
**0.000171878087874303**	-0.703279473572226	1	2	Sart1
**0.00017749638183514**	0.348451710090543	1	2	Dlgap4
**0.000178854733111125**	0.388937734	1	2	Ndn
**0.000179810494854268**	-0.743025264684817	1	2	Ppp1r14b
**0.000180136739452012**	-0.628861471963223	1	2	Nhp2l1
**0.000180359554527324**	-0.705460440793302	1	2	U2af1
**0.000182584953642472**	0.351129178446652	1	2	Ikbkap
**0.000183216954301864**	0.267070523	1	2	Cpt1a
**0.000188187715773822**	-0.317828598088793	1	2	Pabpc1
**0.000189209233828345**	0.303607293041571	1	2	Chml
**0.000191542539256039**	0.505033193763858	1	2	Atxn7
**0.00019293574449223**	-0.725053273121786	1	2	1810022K09I
**0.000193825204701133**	0.778372273755705	1	2	Ulk1
**0.000194283684097397**	0.542197114317092	1	2	Itga10
**0.000195583299232964**	0.615358571449191	1	2	Plekhh2
**0.000198018957981901**	0.717882019753142	1	2	Gria4

**0.000202467386506959**	0.605713263172503	1	2	Slc11a2
**0.000204965424099281**	0.271083091372925	1	2	Exosc10
**0.000209726665390129**	-0.680602607381656	1	2	Hoxa5
**0.000210497845034432**	-0.270374808567866	1	2	Anxa2
**0.000221521370999599**	0.250178051279826	1	2	H6pd
**0.000222454325819181**	0.337837809285518	1	2	Mmp19
**0.000222848927642806**	0.260611493717407	1	2	Def8
**0.000224139496003002**	0.368009646553014	1	2	Itgb5
**0.000230207788934407**	0.507822218037593	1	2	Mfsd6
**0.000230647881930087**	0.291494422533023	1	2	Fkbp1b
**0.000231463427218016**	-0.605330974553029	1	2	Sumo1
**0.000233413910105849**	-0.696054982688112	1	2	Cdk17
**0.000235025339941079**	0.290217821087872	1	2	Cxcl10
**0.000240322956033899**	0.965199072191839	1	2	Chl1
**0.000241260122906276**	0.303060620822102	1	2	Ccnl1
**0.000241592037055752**	0.395672042483954	1	2	Il1r1
**0.000243383015053283**	-1.120947364	1	2	Ivd
**0.000247302197116297**	0.861792904480911	1	2	Art4
**0.000249984092490768**	-0.341913524100879	1	2	Hnrnpk
**0.000251902472974631**	0.254916901559061	1	2	Kdm5a
**0.000256198133445451**	0.897060081182809	1	2	Mafb
**0.000260129778161114**	0.461887247825572	1	2	Hsd3b7
**0.000265401365775332**	0.390562807920094	1	2	Cdk20
**0.000270236945039195**	0.478584990824884	1	2	Slc29a3
**0.0002705517160721**	0.344708064034436	1	2	Txnrd1
**0.000274690963906345**	0.927989102080833	1	2	Mthfr
**0.000278015705706761**	0.680231684585875	1	2	Zfml
**0.000278499988331053**	0.300304223470963	1	2	Syde1
**0.000280119381998675**	0.269326829	1	2	Ext2
**0.000287929792932461**	0.254034091955532	1	2	Abcb1b
**0.000290095694463855**	0.92812369	1	2	Gpatch4
**0.000292804886156199**	0.877001800818196	1	2	Emd
**0.000294903071022254**	0.503606651072915	1	2	Wdr18
**0.000296315387978286**	0.367256659154312	1	2	Ino80
**0.000297686487848747**	-0.781767751430794	1	2	Mrps21
**0.000301616134572827**	0.354975900810351	1	2	Cdkn1a
**0.00031373355982472**	0.377385501222972	1	2	Col4a5
**0.000315108995844893**	0.272413911953474	1	2	Chd2
**0.000315831427891194**	0.559767873107918	1	2	Angptl7
**0.000315953587321119**	-0.868856294	1	2	Brox
**0.0003170727765916**	0.303396984939427	1	2	Umps
**0.000321689275387637**	0.291789153957468	1	2	Ddr2
**0.00032671252090479**	0.494470072003225	1	2	Hdgfrp3
**0.000326970679281407**	0.295577221002521	1	2	Foxp2
**0.000327567654232168**	0.613138781084176	1	2	AW112010
**0.000327724392013036**	0.671704230493379	1	2	Gnao1
**0.00033217033892779**	0.519413454391929	1	2	Ubxn2b
**0.000332525860173567**	0.706917099019766	1	2	Las1l
**0.000332847935370902**	0.409060712966149	1	2	Laptm4a
**0.000339569683732282**	0.348652836372972	1	2	Gprasp2
**0.000343692732415357**	0.768515278843291	1	2	Lepre1
**0.000343923002807049**	-0.789727220887844	1	2	Rlf
**0.000344590490677062**	0.697272433214535	1	2	Mrpl39
**0.00034896392160631**	0.579023608033407	1	2	Runx1
**0.000352740762171781**	0.759665388887928	1	2	Ano1
**0.00035720398450491**	0.302882843895482	1	2	Lgals3

**0.000362023539082929**	0.354919815838304	1	2	Epyc
**0.000367055349884446**	0.394526196034631	1	2	Ptpdc1
**0.000368281579933976**	-0.310620777	1	2	Emc1
**0.000371150443178106**	0.565836059449089	1	2	Fosl2
**0.000372013160305574**	0.322512874048129	1	2	Gab1
**0.000373028430890658**	-0.460202834124183	1	2	Adh1
**0.000377048240125507**	0.342978063396982	1	2	Akap13
**0.000378493740394941**	0.649400498515012	1	2	Atad2b
**0.000379203141040786**	0.515037800904114	1	2	Mapkbp1
**0.000380923422117462**	-0.734099581108869	1	2	C1galt1c1
**0.00038550097042568**	0.578970753590264	1	2	Ankrd40
**0.000390650499447904**	0.276485238231615	1	2	Usp2
**0.000392045799064485**	-1.303632806	1	2	Amigo2
**0.000394994274149444**	0.664744499235089	1	2	Pth1r
**0.000395699264393616**	0.296084567556083	1	2	Fam53c
**0.000397014134757296**	0.626812706739604	1	2	Ampd2
**0.000400769065435317**	0.315670116700142	1	2	Add3
**0.000400874748096831**	0.317194270803616	1	2	Kpna4
**0.000406674631325005**	0.349820952224984	1	2	Tmem64
**0.000409779506007661**	0.493555587292884	1	2	Nr2f2
**0.000409805858419875**	-0.469882939549554	1	2	Cyb5r3
**0.000413030855283682**	0.327039106091268	1	2	Phf23
**0.000414948790663918**	-0.817035773778814	1	2	Adck3
**0.000417469650403324**	0.277081647082875	1	2	Tmem198b
**0.000417865534569847**	0.457258462	1	2	Nfs1
**0.000433588807735969**	-0.361214705838709	1	2	Eef1g
**0.000439552575772431**	-0.540233102860487	1	2	Cav1
**0.00044175021509915**	0.282837107	1	2	Cdadc1
**0.000446980651930502**	0.413121173524963	1	2	Ythdf1
**0.000447515998342006**	-0.598872999	1	2	Eif4a2
**0.000449904145207558**	0.666735420623715	1	2	Kctd12b
**0.000454945376429097**	0.623911454112852	1	2	Snx1
**0.000455406601498185**	0.614627601187317	1	2	Srxn1
**0.000456269916992127**	0.304258974005168	1	2	Dpy19l1
**0.000457746613942817**	0.435668561	1	2	Dedd2
**0.000463725768796437**	0.759460415367809	1	2	Mfsd5
**0.000475748559972919**	0.287412801579313	1	2	Alkbh7
**0.000480338454238487**	-0.810699870874487	1	2	Net1
**0.000489614537089099**	-1.224303925	1	2	Tes
**0.00049035322279774**	-0.574210242939911	1	2	Sema3f
**0.000492149078620002**	0.890426374	1	2	Mxd1
**0.000492956461104227**	0.335981025384926	1	2	Ccdc23
**0.00049342157252628**	-0.665452705	1	2	Hnrnpa1
**0.000493568549620875**	0.594338103486691	1	2	Eif2d
**0.000501582651068246**	0.456217896738194	1	2	Tmem29
**0.000504464402745234**	0.459021152283771	1	2	Ubn2
**0.000505128000046498**	0.374029633753538	1	2	Pabpn1
**0.000509159028812407**	0.381201848	1	2	Smim13
**0.000513365846437681**	0.426715628285741	1	2	Vwa1
**0.000513457707876218**	-1.238777367	1	2	Rasl11a
**0.000513918366368364**	-0.732069386120663	1	2	Ewsr1
**0.000516893809819362**	0.473028246088976	1	2	Mecom
**0.000524325485579511**	0.367893679073478	1	2	Uhrf2
**0.000525968202223173**	-0.425187299327263	1	2	Sep15
**0.000555687719388931**	0.260517164813889	1	2	Kif13b
**0.000557643581148344**	0.451702291238931	1	2	Msl1

**0.000558607375981856**	0.468640486822875	1	2	Prkcg
**0.000559204762524262**	0.470835008462814	1	2	R3hdm1
**0.000565861336809448**	0.335569432103497	1	2	Zswim6
**0.000565945019121065**	0.512259986	1	2	Slc15a4
**0.000566895307539741**	0.347222705417781	1	2	Nfia
**0.000575315452611004**	0.439246561958564	1	2	Camsap2
**0.00057594290414056**	0.333049643	1	2	Zfp593
**0.000576756731822271**	-0.252772321479177	1	2	Ccdc127
**0.000577383233380152**	0.336836529129354	1	2	Ywhag
**0.000577678007735148**	0.432996166638952	1	2	Fam19a5
**0.000590335445675932**	0.700036896246318	1	2	Creb3l1
**0.000594254188232456**	0.777357416424448	1	2	Ube3c
**0.000598261134009722**	-1.099433801	1	2	Cap2
**0.000612429902812637**	0.344954762573811	1	2	Gpr108
**0.000612868610652478**	-0.306318222802431	1	2	Cox7c
**0.000621273933408754**	0.543812122762297	1	2	Msto1
**0.00062617304801631**	0.451919486099583	1	2	Cops5
**0.000646510616341678**	0.571196177871059	1	2	Cyth2
**0.000646952485583331**	0.289034396692161	1	2	Mtmr12
**0.000661904801254999**	0.416386754930105	1	2	Nfe2l2
**0.000666985341898079**	0.700351041	1	2	Wif1
**0.000669348505650852**	-0.433480717626369	1	2	Pcbp2
**0.000670257622165844**	0.303263929388538	1	2	Sec31a
**0.000678276205422062**	-0.554477751841395	1	2	Cand2
**0.000679147228093155**	0.439214092844806	1	2	Cd109
**0.000679699451929264**	0.339814394998224	1	2	2610507B11R
**0.000687861076925825**	1.044805848	1	2	Vcpip1
**0.000694007404245472**	1.644128483	1	2	Ipmk
**0.000695144327371548**	0.428742793	1	2	Zfp462
**0.00069677342377243**	0.347505666355306	1	2	Sbno1
**0.000697888037806509**	0.684499007218006	1	2	Diap2
**0.000699347216050051**	0.283282192798295	1	2	Dnajc13
**0.000711290480740875**	0.318927926970303	1	2	Abhd8
**0.00071403973580145**	0.387359946	1	2	Cdk10
**0.000715067514887932**	0.380186807857519	1	2	Lrrk1
**0.000720468681366677**	0.341437440385738	1	2	Ndst1
**0.000722429224517734**	0.324816791982553	1	2	B4galt5
**0.000728127774570369**	-0.315046428283666	1	2	Naa20
**0.000748292950512726**	1.191790438	1	2	Thra
**0.000757363961951594**	0.444920242	1	2	Atp13a1
**0.000767811571872629**	1.115059568	1	2	Slc35a2
**0.000778850934588404**	0.303352642747312	1	2	Ncoa1
**0.000778867090390203**	0.312908345211917	1	2	Cox18
**0.000780808360359178**	0.413792163056843	1	2	Vps53
**0.000780974481951424**	0.319662141393888	1	2	Mob3c
**0.000781663965403907**	0.391962727163517	1	2	Gtf3c2
**0.000786707748554969**	0.290984932	1	2	Itfg1
**0.000798683518377358**	0.271486520592099	1	2	Bdh2
**0.000802768315647468**	0.318450429050285	1	2	Stx4a
**0.000805236226932358**	0.840753443830916	1	2	Ism1
**0.000807924692233732**	-0.697525022665857	1	2	Ssbp1
**0.000813390162033883**	0.418663787810206	1	2	Atp6v1a
**0.000816532230346121**	0.293161734676799	1	2	Zkscan17
**0.000822816551631434**	0.347434189513773	1	2	Arhgap32
**0.00082693860663624**	-1.000125077	1	2	Ppp2r5a
**0.000828768181652414**	0.745041275929755	1	2	Rabgap1

**0.000832260564954056**	0.289337293307961	1	2	Atg9a
**0.000841395990464869**	0.299331719326007	1	2	St6galnac2
**0.000844718076834004**	0.280181184913659	1	2	Guf1
**0.000861927394828758**	0.528931174585875	1	2	Tm9sf4
**0.000865754182734897**	0.866559735727344	1	2	Abcd4
**0.000865897585263596**	0.531467771929106	1	2	Tnpo3
**0.00087334035298481**	-0.407453916	1	2	Rbm17
**0.000887949437210477**	0.284759230505696	1	2	Ctsc
**0.000894391155285528**	1.070182171	1	2	Rpp25l
**0.000911420373971125**	0.397724798553876	1	2	Zhx3
**0.000923024960332219**	-0.383317478755092	1	2	Atp5b
**0.000924577429662755**	0.691773005956538	1	2	Homer3
**0.000925380693021486**	0.280571979351968	1	2	Lamp2
**0.000929023948057151**	0.474808497257375	1	2	Ccdc34
**0.000944207757494929**	0.378870962937337	1	2	Klf9
**0.00100282733140179**	-0.280993447475446	1	2	Eif2s2
**0.00101282147485268**	0.785518487508237	1	2	Tmcc1
**0.0010181258185111**	0.322720420124301	1	2	Flt1
**0.00101920625795062**	0.422441318725976	1	2	Fnbp1
**0.00103175533008442**	0.511446548964457	1	2	Mmgt1
**0.00107340464897919**	0.594280524	1	2	Ric8b
**0.00108520157301549**	0.403081129860617	1	2	Rnf11
**0.00108674263897213**	0.545720007719528	1	2	Peak1
**0.00111106977915404**	0.503184516687202	1	2	Agtrap
**0.00111422337141687**	0.386629794228888	1	2	Spen
**0.00112963478360102**	0.521985925537302	1	2	Ogfr
**0.00117263625555675**	0.342850214868014	1	2	Arid5a
**0.00118586404642368**	0.532961131	1	2	Lage3
**0.00118876192980307**	0.577683250653412	1	2	Cbx4
**0.00123496285182494**	0.679141132008634	1	2	Ipo7
**0.00123726378083462**	0.476106809225171	1	2	Rfx7
**0.00124442397994742**	0.436302075256269	1	2	Psen1
**0.00128688169607937**	0.358182884252344	1	2	Crtap
**0.00129780168152332**	0.261952178214432	1	2	Mdn1
**0.00130551449921897**	-0.304192461463404	1	2	Psmd4
**0.00133806174275941**	-0.706710065	1	2	Wdr83os
**0.00133861619055912**	0.364085004143453	1	2	Tcf12
**0.00133936167992909**	-1.099548862	1	2	Sgcg
**0.00134221734395106**	-0.690177950377752	1	2	Psmb5
**0.00138581710699194**	0.322486374292942	1	2	Gspt1
**0.00142953828425581**	0.297166435188791	1	2	Tro
**0.00143900315503645**	0.589019545577508	1	2	Tns3
**0.0014501497063905**	-0.891836279616764	1	2	Nop10
**0.00146291735174765**	0.274365393283975	1	2	Nfix
**0.00148252736011882**	0.582069065794661	1	2	Hand2
**0.00150458783676478**	0.337969868051861	1	2	Samd14
**0.00151292909023894**	0.397536257156697	1	2	Sf3b3
**0.00151762784692808**	0.300059443	1	2	Atf5
**0.00152310196444556**	0.36695567	1	2	Bcorl1
**0.00152385129405652**	0.530294602613321	1	2	Nup153
**0.00152650637977695**	0.334683778813821	1	2	1110008P14F
**0.00153231079505672**	0.339468415071308	1	2	E4f1
**0.00153875821538584**	-0.553958358295315	1	2	Rhoj
**0.00155836766347669**	0.454447109543691	1	2	Dlg5
**0.00156845306588884**	0.320760137156358	1	2	Limch1
**0.00156953232105087**	0.338103412831293	1	2	Dcaf4

**0.00157732246373164**	-0.812532305		1	2	Llph
**0.00157844140650717**	0.391246034927041		1	2	Map4
**0.00159030477071455**	0.306546789992299		1	2	1190002N15[illegible]
**0.00159287879354797**	0.475973576779606		1	2	Nbea
**0.00160794166448552**	0.500314681131194		1	2	Myo6
**0.00161457572652833**	-0.752128680075028		1	2	Ppme1
**0.00162422293310493**	0.767767510850756		1	2	Mtmr4
**0.0016256398258757**	-0.804776555		1	2	Srsf4
**0.00165394904386645**	-1.390811404		1	2	Cdh5
**0.00165697015614464**	0.408144676292712		1	2	Pde3b
**0.00166398166326254**	0.680635327563487		1	2	Ctif
**0.0016809416988269**	1.010168529		1	2	Snx21
**0.00174507342133909**	0.503738925759039		1	2	H2-Ab1
**0.00174962525755879**	0.667424850418857		1	2	Smad1
**0.00175183872531515**	-0.444672068940741		1	2	Cd200
**0.00175707877249128**	0.262699531175295		1	2	Fndc3a
**0.00176328513486641**	-0.297252436263546		1	2	Mien1
**0.00176746024725251**	0.316321700462512		1	2	Gpr133
**0.00178729095915142**	-0.954979770754009		1	2	Hey2
**0.00179743310454286**	0.571134472814997		1	2	Akap2
**0.00180892615557227**	-0.590696959276285		1	2	BC002163
**0.00181048741742054**	0.302579875483013		1	2	0610037L13[illegible]
**0.00181910503014427**	0.949923023688089		1	2	Rbmx
**0.00188315894780586**	0.364083467281433		1	2	Kcnab1
**0.00188604991977422**	-0.841792245277409		1	2	Mmp17
**0.00192489578656272**	-0.313799797029505		1	2	Tmbim4
**0.00193378800329846**	1.121614073		1	2	Pepd
**0.00194227046544977**	0.515229687881165		1	2	Camkk2
**0.00195104106645903**	0.841865824748468		1	2	Enc1
**0.00196931133756729**	-0.357312191052857		1	2	Hnrnph1
**0.00197623500255165**	-0.477974823474155		1	2	Scn1b
**0.00200407249224432**	-0.442245779049543		1	2	Mesdc2
**0.00205855104232072**	0.988848256920432		1	2	Irgq
**0.00206369498173575**	0.253281677512703		1	2	Nab1
**0.0020939305697488**	0.782887574191533		1	2	Slc2a10
**0.00212704050435918**	-0.331207362996351		1	2	Tomm7
**0.00214145109639254**	-0.716824768427929		1	2	Srgn
**0.00214870164744532**	-0.453408397583208		1	2	Ndufv1
**0.00218442598356649**	-0.313566506377692		1	2	Zfp266
**0.00219910279395597**	-0.681091020434564		1	2	Rras2
**0.00220058740840277**	0.346694149545067		1	2	Cdc27
**0.00221534191786651**	0.322154099254957		1	2	Srgap3
**0.00221838962644366**	0.302065689		1	2	1110058L19[illegible]
**0.00222306056829591**	0.457124085515735		1	2	Elp3
**0.00222981600966189**	0.252350846		1	2	Cstb
**0.00223683423950404**	0.855261310283507		1	2	Ldlrad3
**0.00224073408582378**	0.483145694051899		1	2	Tcerg1
**0.00227573574847079**	0.331579044523862		1	2	Sik3
**0.00228508239882562**	0.972361024030852		1	2	Rap2c
**0.00232898850464053**	-1.217586157		1	2	Mgst3
**0.00233024362471625**	0.447647528736486		1	2	Pcdh9
**0.00233204985239008**	-0.491014146873131		1	2	Smim14
**0.00236160792701741**	-0.590202092956729		1	2	Dock6
**0.00237764713366037**	0.398431151186154		1	2	Scoc
**0.00238514318100276**	0.882104842721813		1	2	Mdm2
**0.00239741624022058**	0.292221861504621		1	2	Ip6k1

**0.00242800544206038**	-1.142867375	1	2	Ahsa1
**0.00243859273217992**	-0.259625405966085	1	2	Myl6
**0.0024635699061819**	0.749661376069599	1	2	Sbf2
**0.00247327569074921**	0.31619465	1	2	Laptm4b
**0.00248028835069272**	-0.264921111563963	1	2	Rbm8a
**0.002529434**	-0.279519998244073	1	2	Marc2
**0.00264598591269314**	-0.291642473667878	1	2	Pcmt1
**0.00267667724929304**	0.426532847326707	1	2	Ubp1
**0.00270639290607143**	0.700729597880015	1	2	Neat1
**0.00272430678104417**	0.313470350197967	1	2	Ifi203
**0.0027530926951453**	-0.256790138	1	2	Rit1
**0.00278822691610561**	0.268086971330131	1	2	Ppp1r12a
**0.00285467406190716**	0.258991613227965	1	2	Kras
**0.00291750746889698**	-0.945998212263287	1	2	Pck2
**0.00292484712510985**	-1.005980539	1	2	B3galt1
**0.00295629816594927**	0.290094894055683	1	2	Alkbh5
**0.00296468574334768**	0.370282304359492	1	2	Ednra
**0.00297928835376677**	-0.828141374970599	1	2	Rasl12
**0.00300032563387574**	0.357750853722805	1	2	Myo18a
**0.00300472793656095**	0.387373752491753	1	2	Celf1
**0.00301749121156755**	0.372499775462441	1	2	Fam111a
**0.00304488553666776**	0.503320119201614	1	2	Tbc1d9b
**0.00304546032070452**	0.338566141	1	2	Pcsk5
**0.00304632201229857**	0.358885841250155	1	2	Ralgapb
**0.00307688102397438**	-0.254878826052565	1	2	Per1
**0.00313236201938038**	0.467305828	1	2	Rfwd2
**0.00315766873465285**	0.536204322638779	1	2	Chpt1
**0.00316650282087386**	0.456737752976561	1	2	Zfp62
**0.00319291040242203**	0.725858898	1	2	Csad
**0.00320241588887446**	0.381841752380873	1	2	Akap7
**0.00322392903193853**	0.737679968859352	1	2	Dcakd
**0.00329430746811284**	0.626325769750864	1	2	Ppp3ca
**0.00333825575761911**	0.260075924497348	1	2	Zdhhc20
**0.00336827466617828**	0.344107144420105	1	2	Micu2
**0.00337010738161355**	0.262080554511209	1	2	Foxc2
**0.00338760552699247**	0.252119257298193	1	2	Ap3s2
**0.00340430804333676**	0.911420179567043	1	2	Fyn
**0.00344022393454066**	0.363100496407475	1	2	Pink1
**0.00345011738406109**	0.296496337827783	1	2	Sh3pxd2b
**0.00347947014236847**	-0.798141367536386	1	2	Zfp414
**0.00351634151105966**	1.247674736	1	2	Idua
**0.00353804813544825**	-0.818257773266871	1	2	Med21
**0.00359763367997784**	0.325611084	1	2	Chd4
**0.00363718987170065**	-0.250355822838051	1	2	Ppib
**0.00366566316497047**	0.270811390310346	1	2	Siae
**0.00367552684288286**	0.375778598024837	1	2	Egr2
**0.00372323040004922**	0.420159049286119	1	2	Tgm2
**0.00374159035355081**	0.323742128242472	1	2	Itga7
**0.00374563793561864**	0.403669155425394	1	2	Ttc9c
**0.00375569274881392**	0.466588519023149	1	2	Gtf3c6
**0.00376552701448582**	0.498724985696258	1	2	Gm17455
**0.00381305916011856**	-0.457410844476406	1	2	Nudcd3
**0.00385812820847521**	-1.124356618	1	2	Cd24a
**0.00387212501695703**	0.918921019518135	1	2	Bcor
**0.00387955803949536**	0.265369974311453	1	2	Emp1
**0.0038929766418403**	0.952413918082174	1	2	Prpf19

**0.00393334373838167**	0.297810202167386	1	2	Abi1
**0.00396206609154075**	-0.702468115585865	1	2	Supt4a
**0.00397530623895103**	0.296697674292602	1	2	Rab10
**0.00403786090856681**	0.928766128463896	1	2	Ngf
**0.00404436267351553**	-0.743481313033846	1	2	H2afz
**0.00404785207166295**	-1.14847424	1	2	Gkn3
**0.00408626959123802**	-0.954391532407644	1	2	Plcd1
**0.00410625279123472**	0.454994175198014	1	2	Pml
**0.00415650092151363**	0.281675387651608	1	2	Rapgef1
**0.00415829194283503**	0.688073468986402	1	2	Abi2
**0.00416195969556075**	-0.603308729172261	1	2	Rcor1
**0.00418144361921863**	0.598581313569676	1	2	Ifi35
**0.00419927933382301**	0.683617643585865	1	2	Cpeb2
**0.00423952223703753**	0.477590615635729	1	2	Rsf1
**0.00425741162922166**	0.342594314717205	1	2	Ncln
**0.00429906242173225**	0.278951940354156	1	2	Jun
**0.00432688428905397**	0.304727241386882	1	2	Jag1
**0.00435535630926691**	0.382468353405325	1	2	Nr4a1
**0.00437295823900629**	-0.765008711482432	1	2	Glo1
**0.0043757695642083**	-0.491989100896613	1	2	Snrpa
**0.00438005738586994**	0.625651940503641	1	2	Ptn
**0.00439982025322748**	0.284532098087728	1	2	Slc39a7
**0.00454514862625023**	-0.577743018	1	2	Tomm20
**0.00458237846111291**	-0.626841544194364	1	2	Capzb
**0.00471629318813712**	-0.493328301812144	1	2	Nifk
**0.00485700834383702**	-0.807756826235828	1	2	Cdkal1
**0.00491130150014515**	0.483590593477179	1	2	Gigyf2
**0.00494469690789909**	0.275571528618875	1	2	Tmem175
**0.00496757709830651**	0.482536774832104	1	2	Fmnl3
**0.00497522839540329**	0.390561897770515	1	2	Pcnx
**0.00499605744837231**	0.306249615374399	1	2	Il13ra1
**0.00507165933184575**	0.486403707912978	1	2	Pdxdc1
**0.00510836037323019**	-0.895844340826983	1	2	Hnrnpl
**0.00511925538768332**	-0.254437941	1	2	Cox6b1
**0.00513241952603061**	0.362715609426854	1	2	Epc2
**0.00519442892565786**	0.458685559009924	1	2	Lrig2
**0.00524668843370664**	0.287407382620653	1	2	Ank
**0.00532693616595965**	-0.905916451840717	1	2	Cdo1
**0.00533812874720883**	0.296299808910442	1	2	Fxr2
**0.00535978269798332**	-0.420897087031896	1	2	Ptgis
**0.00541441190018532**	-0.256013467	1	2	Abhd14a
**0.00544688648894223**	0.339723578	1	2	Sin3a
**0.00548455518571163**	-0.351880301	1	2	Serf2
**0.00551892926497945**	0.297759960618134	1	2	Polk
**0.00551975648580144**	0.565231529497781	1	2	Fbxo33
**0.00552640167843938**	0.508266609517476	1	2	Tceal8
**0.00560425835831708**	-0.424857115262438	1	2	S100a10
**0.00560571811419485**	0.278816682755374	1	2	Pcf11
**0.00564661364624677**	-0.294499804364047	1	2	Ngfrap1
**0.00567967888811608**	0.394759047166708	1	2	Akap11
**0.0056861709776807**	0.953362555485348	1	2	Bcas3
**0.00574304552624193**	0.323572844256897	1	2	Pisd-ps1
**0.00577268110889666**	0.254110033096224	1	2	Mybbp1a
**0.00577511339601104**	0.848125902550275	1	2	Ube3a
**0.00589162521653199**	0.389230985346281	1	2	Fam174b
**0.00591356772564724**	0.320143855785633	1	2	Rgs12

**0.0059179886549576**	0.674760529149276	1	2	Plod1
**0.00596556154059338**	0.273094733284748	1	2	B3galnt1
**0.00598488096387713**	0.427346005361409	1	2	Nasp
**0.00604101136672037**	-0.586638841540876	1	2	Stard13
**0.00608521972748036**	-0.706477408964024	1	2	Sgk1
**0.00615977658331061**	-0.615857387540016	1	2	Pawr
**0.00619933169533779**	0.337533222573755	1	2	Bag3
**0.00620237421278133**	0.963503546487712	1	2	Pi4k2a
**0.00626745308577229**	-0.312898299	1	2	Oard1
**0.00627920573831144**	-0.637260212124972	1	2	Zfp598
**0.00627948976040084**	0.447710182	1	2	Rnpepl1
**0.00628084884930248**	0.597516113665551	1	2	Naprt1
**0.00633141416566778**	0.270447888404225	1	2	Chchd6
**0.00638193500633904**	-0.256574076077446	1	2	Aplp2
**0.0064222948718885**	-0.509473299525611	1	2	2610002J02F
**0.00651719096578394**	0.951422408050997	1	2	Arfgap1
**0.00655743889856516**	-0.290783430885703	1	2	Supt16
**0.00678541383555295**	0.346615404309912	1	2	Fam98a
**0.00678572334441156**	-0.708950845733071	1	2	Tbc1d1
**0.0068501396405128**	-0.379806727384387	1	2	2900060B14I
**0.00686074893817425**	0.440785662963707	1	2	Prnp
**0.00687691165217546**	0.296017776974544	1	2	BC005561
**0.00687781733474762**	0.259812015774713	1	2	Cog2
**0.00693714886256746**	-0.786665585862897	1	2	Zcchc14
**0.00694661205033546**	0.555183891377008	1	2	Rnf144a
**0.00697792132760493**	-0.539570234614826	1	2	Anapc11
**0.00699631204348165**	0.673466118	1	2	Mme
**0.00702854942699282**	0.373044289497131	1	2	Hjurp
**0.00706272645998187**	0.642852244558367	1	2	Frs2
**0.00709672759406134**	0.370320907347071	1	2	Adam10
**0.00715876868350556**	0.526597165234338	1	2	Cnnm3
**0.00717502789762012**	-0.344110351564257	1	2	Ctnnb1
**0.00721303380363698**	-0.614624721744488	1	2	Parp1
**0.00731103660430447**	0.512525586681981	1	2	Fam208a
**0.00740351607528638**	0.284965771070489	1	2	Wbp1l
**0.00745677548921956**	-0.305947542647633	1	2	Yif1a
**0.00745749858396815**	0.476215643367675	1	2	Tsc22d4
**0.0074721547695364**	-0.748718885800094	1	2	Ptpla
**0.00747680957855441**	-0.391618033574427	1	2	Gle1
**0.00749317277991002**	0.614817101722983	1	2	Slc35b2
**0.00751914161633529**	0.390986718	1	2	Dnase1l1
**0.00754104411024944**	-0.588138574012656	1	2	Eif3m
**0.00756599338547291**	-0.440541896520714	1	2	Anxa1
**0.00757890785982873**	0.512998230798806	1	2	Abhd11
**0.00758868816642698**	-1.521953326	1	2	Ramp1
**0.0077304810296294**	0.545322192240124	1	2	Champ1
**0.00773859378149293**	0.353659698701974	1	2	Uggt2
**0.00774406723141508**	0.314644874904016	1	2	Il17rc
**0.00777091553408682**	0.808289312067994	1	2	Abcb1a
**0.00779578909659066**	-0.284018370010221	1	2	Sec13
**0.00787699165275486**	-0.576462990552176	1	2	Tnc
**0.00790049298440671**	0.328632522253164	1	2	Morc4
**0.00801845525699453**	-0.636915028088347	1	2	A4galt
**0.00810937000536875**	-0.525456602878543	1	2	Osbpl3
**0.00814483193162466**	-0.790734197884391	1	2	Ptp4a3
**0.00818313119697622**	-0.294501737390193	1	2	Reep5

**0.00827252040899677**	-0.987893069464183	1	2	Sarnp
**0.00828284101704338**	0.418325605	1	2	Kdm5c
**0.00835364994034208**	-0.886199765770763	1	2	Cyb5r1
**0.00843304686454711**	-0.873624204334048	1	2	Npr1
**0.00843618292674687**	0.338099706361465	1	2	Rasa2
**0.0084395455073037**	0.648830328880672	1	2	Ltn1
**0.00847255322090814**	-0.348845903935199	1	2	Ensa
**0.00850705998646734**	0.255488041437952	1	2	Ddx21
**0.00857252406723221**	-0.410172611	1	2	Myct1
**0.00863281904377135**	0.294452579018992	1	2	Gpr64
**0.00864577731105682**	0.604533304200467	1	2	Psmd10
**0.00866722396967239**	0.499496374518516	1	2	Vhl
**0.00868818098048761**	0.293466599873609	1	2	Airn
**0.00880380841978177**	-0.861521205860708	1	2	Cuedc1
**0.00883763365046446**	-0.602418068327904	1	2	2310035C23I
**0.008853917**	0.255277516016762	1	2	Jarid2
**0.0088766340859356**	-1.067288102	1	2	Kctd12
**0.00888661351034775**	-0.762511606587642	1	2	Casp12
**0.00898866099193227**	0.258815243814871	1	2	Tia1
**0.00899481071248259**	-0.911252650649072	1	2	Zscan26
**0.00908597415548781**	-0.437535080032054	1	2	Atp6v0e
**0.00911490133059441**	0.583283661	1	2	Ankrd13a
**0.00914518600215146**	-0.618278344515805	1	2	Rnft1
**0.00916038865707216**	-0.389352128271881	1	2	Cox7a2
**0.00922583439417217**	0.498625023697601	1	2	Tnfrsf11b
**0.009291167706619**	-0.721804298689652	1	2	Dguok
**0.00936441591046651**	0.296430607	1	2	Cys1
**0.00942935682827861**	0.418398991901847	1	2	Carhsp1
**0.00965142584333311**	0.317006976382514	1	2	Timm17b
**0.00969250059633859**	0.598938962444831	1	2	Smim8
**0.00978453286371762**	0.319967428103408	1	2	Ralgds
**0.00981045918583066**	0.383565725607395	1	2	Gstt3
**0.00983983647538663**	-0.852348592915208	1	2	Rassf3
**0.00987492151321038**	0.276363562721116	1	2	Wnk1
**0.00990754179511919**	-0.625124486120474	1	2	Rtca
**0.00999630971850392**	0.458046814541375	1	2	Clcn3

p_val	avg_logFC	p_val_adj	cluster	gene
3.08360641213438e-54	2.32	4.25876881579879e-50	3	Pecam1
4.48214210370383e-53	2.48	6.19028645942535e-49	3	Egfl7
1.10725046001991e-46	2.33	1.5292236103335e-42	3	Ptprb
1.50157481877624e-45	-1.87	2.07382498221187e-41	3	Rarres2
6.91476987937604e-45	2.19	9.54998868040625e-41	3	Mmrn2
1.65056030647539e-44	2.61	2.27958883927315e-40	3	Cdh5
2.17688419446862e-44	2.07	3.00649476098061e-40	3	Sox18
9.3267191881618e-43	2.47	1.28811318707703e-38	3	Cd93
2.06344834842407e-41	-1.81	2.84982851400849e-37	3	Lrp1
2.64031108138754e-41	2.45	3.64653363450434e-37	3	Tie1
3.81235347483192e-41	-1.47	5.26524138409037e-37	3	Col1a1
4.06910086295195e-41	-1.24	5.61983520182294e-37	3	Col1a2
1.05317475628774e-40	-1.47	0.00	3	Col3a1
4.00091276138458e-40	2.59	5.52566061474825e-36	3	Vwf
1.67774605363413e-39	-2.07	2.3171350746741e-35	3	Col6a1
6.31155426524216e-39	-1.51	8.71688759572595e-35	3	Ogn
1.79388650713616e-38	-1.14	2.47753665500575e-34	3	Lgals1
1.17964844440998e-37	2.50	1.62921246657462e-33	3	Bmx
1.63190598304738e-37	2.03	2.25382535318673e-33	3	Cldn5
1.81692003424286e-37	-0.97	2.50934825929281e-33	3	Igfbp7
1.86857565992536e-36	1.61	2.58068984392292e-32	3	Ece1
3.998482221115E-36	2.65	5.52230379558193e-32	3	Nos3
8.78210710981125e-36	-2.27	1.21289681293603e-31	3	Htra3
3.82277527454188e-35	1.54	5.27963493166979e-31	3	Epas1
4.47633092852822e-35	-2.19	6.18226064539032e-31	3	Islr
7.89967750653228e-35	2.53	1.09102446042717e-30	3	Stab1
3.56375573814487e-34	1.86	4.92190304995187e-30	3	Bmp6
2.73793727363411e-33	2.46	3.78136516861607e-29	3	Icam2
4.36561042881657e-33	-1.59	6.02934456323857e-29	3	Axl
4.87324842863024e-33	-1.60	6.73044340478122e-29	3	Fxyd1
8.22486121754493e-33	-1.37	1.13593558275513e-28	3	Prelp
4.945084882327E-32	2.50	6.82965673098181e-28	3	Myct1
5.62865060675229e-32	-1.03	7.77372935298558e-28	3	Mt1
7.69327443883966e-32	1.57	1.06251813274815e-27	3	Cytl1
3.29247205846908e-31	-2.25	4.54723315995165e-27	3	Dpt
9.42933986484356e-31	-1.31	1.30228612873354e-26	3	Gsn
1.36921914957197e-30	-1.58	1.89102856747385e-26	3	Serping1
1.6350745352467e-30	-1.46	2.25820144062921e-26	3	Ddr2
2.26369827448188e-30	2.22	3.12639368688692e-26	3	Clec14a
3.36481968258985e-30	0.92133463016€	4.64715246362485e-26	3	Clu
6.29815793214922e-30	0.90793000559	8.69838592009129e-26	3	Tm4sf1
1.19189118881796e-29	-1.69	1.64612092087648e-25	3	Loxl1
1.23106855581153e-29	-1.10	1.7002287824313e-25	3	Aebp1
2.14287456299272e-29	2.25	2.95952405894925e-25	3	Tek
3.21172685569651e-29	-1.93	4.43571596040245e-25	3	Mmp23
3.60755756627621e-29	2.09	4.98239775478408e-25	3	Ecscr
3.64509164489372e-29	-2.10	5.03423607076271e-25	3	Fbln1
3.9388143280219e-29	1.76	5.43989646843105e-25	3	Srgn
4.35804990982325e-29	2.21	6.01890273045689e-25	3	Esam
5.27292220181274e-29	-1.76	7.28243285292358e-25	3	Adamts2
6.80187252811733e-29	-1.37	9.39406614858285e-25	3	Col6a2
1.68541384218061e-28	-1.15	2.32772505743564e-24	3	Ecm1
2.19026981156426e-28	2.18	3.0249816367514e-24	3	Podxl
3.23729443996698e-28	-1.49	4.47102735103839e-24	3	Mt2
8.47316421895671e-28	-1.92	1.17022871028011e-23	3	Abcc9

**1.16661511445548e-27**	-2.24	1.61121213457446e-23	3	Cygb
**1.44495876733605e-27**	1.75	1.99563255356782e-23	3	Lmo2
**3.30056517002334e-27**	2.35	4.55841055631924e-23	3	Gja5
**4.64911497307941e-27**	-0.77634062331	6.42089268931998e-23	3	Dkk3
**8.45517179437664e-27**	-1.93	1.16774377652136e-22	3	Pcolce
**9.67923711505691e-27**	-1.48	1.33679943796051e-22	3	Cd302
**1.07688930458714e-26**	-1.70	1.48729181856531e-22	3	Abi3bp
**1.15599695973277e-26**	0.992412826685	1.59654740108692e-22	3	Ly6e
**1.70370586689538e-26**	-1.85	2.35298817276921e-22	3	Gpx8
**1.82316763910197e-26**	-1.48	2.51797682636373e-22	3	Lum
**3.72855365870128e-26**	2.20	5.14950545803234e-22	3	Cd24a
**4.78416900271514e-26**	1.80	6.60741580964988e-22	3	Itgb4
**7.37933221597057e-26**	-2.09	1.0191595723477e-21	3	Lama2
**1.10913841605806e-25**	1.50	1.53183106641778e-21	3	Fam3c
**1.25537113804442e-25**	2.72	1.73379307875315e-21	3	AU021092
**1.45299942444724e-25**	-1.93	2.00673750510409e-21	3	Crispld2
**1.45412477318832e-25**	2.03	2.00829172425038e-21	3	Tmem158
**1.75573919119661e-25**	-2.24	2.42485139696164e-21	3	Abca8a
**2.10034099688849e-25**	1.63	2.90078095080269e-21	3	Sdpr
**2.49006581884423e-25**	-1.56	3.43902990240577e-21	3	C3
**3.25173160453568e-25**	1.98	4.49096651902423e-21	3	Mall
**4.99146305511375e-25**	-1.38	6.8937096254176e-21	3	Fbn1
**6.46083865725123e-25**	2.07	8.92306426952967e-21	3	S1pr1
**1.10933081474242e-24**	1.79	1.53209678824076e-20	3	Tinagl1
**6.520012324452E-24**	-0.81786121864	9.00478902130065e-20	3	Celf2
**1.21706449227417e-23**	1.82	1.68088777027985e-19	3	Cgnl1
**1.55929473087835e-23**	1.47	2.15354195281609e-19	3	Cmip
**1.64719410209989e-23**	1.58	2.27493977441016e-19	3	Crim1
**1.99457659158637e-23**	-1.68	2.75470973063994e-19	3	Serpinf1
**2.23206389094672e-23**	-1.15	3.08270343978651e-19	3	Dcn
**2.4629455201084e-23**	2.00	3.40157405782171e-19	3	Ehd4
**4.77091391146117e-23**	2.10	6.58910920311902e-19	3	Tal1
**4.82116279829511e-23**	-1.89	6.65850794072538e-19	3	C1s1
**8.48276104818996e-23**	1.29	1.17155412836552e-18	3	Gata2
**9.21003583438178e-23**	-1.63	1.27199804908647e-18	3	Mfap4
**9.44444070374096e-23**	-1.88	1.30437170559366e-18	3	Prrx1
**1.36520338880288e-22**	0.936007785214	1.88548240027565e-18	3	H2-K1
**2.1527329884636e-22**	1.21	2.97313953036707e-18	3	Klf2
**3.48264560909106e-22**	1.10	4.80988185071566e-18	3	Cav1
**4.22824276333257e-22**	2.22	5.83962608043861e-18	3	Edn1
**4.36410795830569e-22**	-0.91794328306	6.02726950121599e-18	3	Pmp22
**7.64210269442427e-22**	-2.03	1.05545080312694e-17	3	Gas1
**1.57113352846282e-21**	-1.73	2.16989251616001e-17	3	Olfml2b
**1.62180106207432e-21**	1.69	2.23986944683084e-17	3	Efnb2
**1.76470525273669e-21**	-1.03	2.43723442455465e-17	3	Il11ra1
**2.52379026057902e-21**	-2.14	3.48560672888568e-17	3	Smoc2
**3.40888301101348e-21**	-1.98	4.70800832651072e-17	3	Clec3b
**4.14493601878527e-21**	-1.67	5.72457113554434e-17	3	Col14a1
**4.93970048733744e-21**	2.01	6.82222034306174e-17	3	F11r
**5.92859935088154e-21**	-1.68	8.18798856350249e-17	3	Igf1
**9.65886112059121e-21**	-1.15	1.33398530936485e-16	3	Fgl2
**9.82955374956582e-21**	1.30	1.35755966835254e-16	3	Ramp2
**1.11093336538552e-20**	1.65	1.53431007093393e-16	3	Cbfa2t3
**1.11181265607667e-20**	-1.89	1.53552445930749e-16	3	Chpf
**1.20327830532818e-20**	-1.40	1.66184766748875e-16	3	Ltbp1
**1.43971683842067e-20**	-1.70	1.98839292554279e-16	3	Pdgfra

**2.09460625278497e-20**	1.66	2.89286069572133e-16	3	Nrarp
**2.4962249037269e-20**	1.38	3.44753621453722e-16	3	Cav2
**3.8713925833742e-20**	-2.22	5.34678029689811e-16	3	Clec11a
**4.85455585514658e-20**	-1.59	6.70462709154295e-16	3	Gpx3
**5.12823974715413e-20**	-0.96116332200	7.08261191479456e-16	3	Cryab
**8.24804365378112e-20**	2.02	1.13913730902371e-15	3	Gprc5a
**8.42944285566817e-20**	-2.18	1.16419035279633e-15	3	Dpep1
**1.21482951695128e-19**	2.23	1.67780104586141e-15	3	Rasip1
**1.48204524761628e-19**	-1.71	2.04685269148285e-15	3	Bicc1
**1.83504661749812e-19**	-2.07	2.53438288342666e-15	3	Scn7a
**1.93575329332622e-19**	-1.84	2.67346887341284e-15	3	Aspn
**2.06017934998712e-19**	2.45	2.84531370026721e-15	3	Pkhd1l1
**2.40397409988229e-19**	2.39	3.32012862934743e-15	3	Irf6
**2.53305512829061e-19**	1.74	3.49840243768216e-15	3	Pkn3
**3.53237364473713e-19**	-1.16	4.87856124074646e-15	3	Igfbp4
**3.84392486878882e-19**	-1.62	5.30884463628424e-15	3	Timp1
**4.89090530729445e-19**	-1.04	6.75482931990437e-15	3	Col5a1
**7.67657587152651e-19**	-1.93	1.06021189361653e-14	3	Entpd2
**7.76983360670973e-19**	-1.56	1.07309171942268e-14	3	Tgfbi
**8.6394608928212e-19**	1.86	1.19319594390754e-14	3	Selp
**8.67857115611944e-19**	0.73	1.19859746237166e-14	3	Fkbp1a
**9.17735789787531e-19**	-1.18	1.26748489927556e-14	3	Mrc2
**1.71075744167906e-18**	2.26	2.36272710270295e-14	3	Adh7
**1.71470228814765e-18**	1.69	2.36817533016071e-14	3	Palmd
**2.13033503701755e-18**	-1.73	2.94220571962494e-14	3	Rbp4
**2.90356275013354e-18**	2.05	4.01011051420944e-14	3	Gpr116
**5.76271991763903e-18**	-1.26	7.95889247825126e-14	3	Sdc2
**5.95007479996884e-18**	-2.03	8.21764830623696e-14	3	Ctsk
**7.35015930760727e-18**	-1.20	1.01513050197364e-13	3	Atp1a2
**8.43671858753696e-18**	-1.14	1.16519520412473e-13	3	Col6a3
**1.14650780467504e-17**	-1.21	1.58344192903669e-13	3	Bmp1
**1.26007873076769e-17**	-0.79911134978	1.74029473506326e-13	3	Lhfp
**1.35454576360565e-17**	-1.57	1.87076315411577e-13	3	Olfml3
**1.59708330802132e-17**	-1.98	2.20573175670825e-13	3	Tnfaip6
**2.19470518678882e-17**	2.01	3.03110733347404e-13	3	Sox7
**3.41810580824123e-17**	-1.45	4.72074593176197e-13	3	Pdlim2
**4.08181702319292e-17**	1.22	5.63739749073175e-13	3	Anxa3
**4.14351911356024e-17**	-1.80	5.72261424773805e-13	3	Cfb
**5.96527989728114e-17**	-2.01	8.23864806613498e-13	3	Egfr
**6.38684107576748e-17**	2.34	8.82086620974247e-13	3	Sox17
**6.44990182495634e-17**	-1.49	8.9079594104472e-13	3	Prg4
**7.12325948286286e-17**	-0.87141961931	9.8379336717819e-13	3	Anxa1
**7.23965513771224e-17**	1.29	9.99868771069437e-13	3	Bmp4
**1.13789456831726e-16**	-2.06	1.57154618830297e-12	3	Pdgfrl
**1.27570977605809e-16**	2.15	1.76188277171382e-12	3	Acer2
**1.34748608970819e-16**	1.78	1.86101303849598e-12	3	Gbp4
**1.44098043671262e-16**	1.89	1.9901380811438e-12	3	Stmn2
**1.52374901337825e-16**	1.09	2.10444976237671e-12	3	Dennd5b
**1.86546881266354e-16**	-1.33	2.57639897716962e-12	3	Efemp2
**1.88390793160991e-16**	-0.84211515154	2.60186524434645e-12	3	Plxdc2
**2.34490057605661e-16**	-1.43	3.23854218559178e-12	3	Nbl1
**2.85272814149792e-16**	-0.82525673308	3.93990283622277e-12	3	Twsg1
**2.88624281989303e-16**	-1.31	3.98618995855427e-12	3	Colec12
**3.08233455568548e-16**	1.35	4.25701225485722e-12	3	Nuak1
**3.57983143804717e-16**	-1.77	4.94410519908694e-12	3	Akap12
**5.08993656605521e-16**	-2.09	7.02971139137884e-12	3	Medag

**5.61204542463236e-16**	-1.51	7.75079593595975e-12	3	Lsp1
**8.48335758807434e-16**	1.50	1.17163651648895e-11	3	Notch1
**8.5500075839936e-16**	-2.10	1.18084154742536e-11	3	Cd248
**9.04489745977248e-16**	2.05	1.24919078816918e-11	3	She
**1.16250340793173e-15**	-1.46	1.60553345669452e-11	3	Fat1
**1.21244676289623e-15**	-0.66285342365	1.67451022423598e-11	3	Rbms3
**1.3117915520914e-15**	-1.61	1.81171531259343e-11	3	Cpxm1
**1.50711916149362e-15**	-1.56	2.08148227393884e-11	3	Foxp2
**1.58457996325521e-15**	2.20	2.18846338725177e-11	3	Eltd1
**1.72833226364294e-15**	-1.08	2.38699968931727e-11	3	Itgbl1
**1.83802713331651e-15**	-1.37	2.53849927382343e-11	3	Hk2
**2.02399683633082e-15**	1.84	2.79534203065649e-11	3	Slc9a3r2
**2.24588023954458e-15**	-1.61	3.10178519883501e-11	3	Gfpt2
**2.98445242746807e-15**	-2.08	4.12182724757615e-11	3	Scara5
**3.2196297063398e-15**	0.528058078253	4.4466305874259e-11	3	H2-D1
**3.23113967280604e-15**	-1.15	4.46252700211242e-11	3	Adamts5
**3.75289792622877e-15**	1.87	5.18312732591455e-11	3	Ccm2l
**4.5231898606733e-15**	-0.82123868469	6.2469775165759e-11	3	Fmo2
**4.82950856673396e-15**	-1.39	6.67003428151628e-11	3	Col15a1
**5.78762403012245e-15**	1.11	7.99328754800211e-11	3	Sfrp1
**6.43479518845621e-15**	-1.29	8.88709563477688e-11	3	C4b
**7.159223722918E-15**	1.97	9.88760388372205e-11	3	Gja4
**7.74290057838221e-15**	-1.44	1.06937199888037e-10	3	Igfbp5
**8.04772119076361e-15**	-1.82	1.11147077365636e-10	3	Wisp2
**8.93855146295268e-15**	0.699386264562	1.23450334254839e-10	3	Bcam
**1.02419613481172e-14**	2.06	1.41451728178846e-10	3	Amigo2
**1.04804499714476e-14**	-0.78832012584	1.44745494555663e-10	3	Gem
**1.06720621986751e-14**	-1.96	1.47391851025902e-10	3	Gdf10
**1.11733378111637e-14**	1.87	1.54314968509982e-10	3	Ephx2
**1.2181938897003e-14**	-1.16	1.68244758106508e-10	3	Tgfb3
**1.4019074859957e-14**	-1.19	1.93617442890866e-10	3	Epb4.1l2
**1.61364779802653e-14**	2.00	2.22860897385444e-10	3	Notch4
**1.74836821447666e-14**	-0.86	2.41467134101372e-10	3	Nid1
**1.79866186889614e-14**	1.63	2.48413190713246e-10	3	Clic5
**2.1253212962259e-14**	-1.65	2.93528124221759e-10	3	Thbs3
**2.3767279441808e-14**	2.18	3.28249896370811e-10	3	Gcnt1
**2.51153047144619e-14**	-1.46	3.46867473411433e-10	3	Wfdc1
**3.02395193917784e-14**	2.04	4.17638002319851e-10	3	Plcg2
**3.29471101138203e-14**	-1.26	4.55032537781972e-10	3	Vcan
**3.34563039796163e-14**	-0.79622329881	4.6206501426248e-10	3	Oat
**3.69626496396927e-14**	-1.78	5.10491154173796e-10	3	Ms4a4d
**3.75944711415037e-14**	-0.97982584427	5.19217240935308e-10	3	Gpc6
**3.76369140716645e-14**	-1.33	5.19803420243758e-10	3	Vasn
**3.79424059474202e-14**	-0.29389687401	5.2402256853982e-10	3	Dpysl3
**4.62231463991222e-14**	1.52	6.38387874918276e-10	3	Heg1
**5.36364057104407e-14**	1.63	7.40772399266896e-10	3	Rnf125
**5.37614900685117e-14**	-1.33	7.42499939336216e-10	3	Phlda1
**5.54144114211883e-14**	1.85	7.65328436138032e-10	3	Fam189a2
**5.59362397116906e-14**	-0.63662394870	7.72535406658159e-10	3	Rcn3
**6.30687665998222e-14**	-1.37	8.71042735510145e-10	3	Col4a5
**6.55400279363317e-14**	-0.89	9.05173325828677e-10	3	Pdgfrb
**7.55572439359113e-14**	-1.72	1.04352109599887e-09	3	Il33
**7.667279160709E-14**	-1.13	1.05892792488552e-09	3	Nr2f2
**8.08523531235479e-14**	1.43	1.11665184898932e-09	3	Shank3
**8.59412467711503e-14**	1.61	1.18693455915636e-09	3	Ptgs1
**9.20541454746521e-14**	-1.40	1.27135980315042e-09	3	Ifi27l2a

**9.74373952429177e-14**	-2.01	1.34570786569994e-09	3	Igfbp6
**1.05613127770462e-13**	-1.34	1.45862290763785e-09	3	Arhgef25
**1.08789379101418e-13**	1.74	1.50249011476968e-09	3	Kctd12
**1.12824788098277e-13**	1.54	1.5582231484253e-09	3	Plvap
**1.2097460493912e-13**	-0.57348966456	1.67078026881419e-09	3	S100a6
**1.41806094908541e-13**	-1.80	1.95848397678186e-09	3	Col16a1
**1.43494700931368e-13**	-1.06	1.98180531456312e-09	3	Tspan3
**1.4788344718731e-13**	0.95919920352	2.04241828910394e-09	3	Ctsh
**1.50855567195537e-13**	2.14	2.08346623853757e-09	3	Gkn3
**1.68504362540785e-13**	-0.93	2.32721375105078e-09	3	Pkd2
**1.6969988363267e-13**	1.74	2.3437250928508e-09	3	Car8
**1.76083953898246e-13**	-0.43931737909	2.43189548728867e-09	3	Sspn
**1.76900471241955e-13**	-1.37	2.44317240832264e-09	3	Lpar1
**2.07586021669674e-13**	-0.63611569291	2.86697054527987e-09	3	Scpep1
**2.20393635908691e-13**	-1.81	3.04385650553493e-09	3	Comp
**2.22086364681913e-13**	-0.72731027654	3.0672347826219e-09	3	Ndrg2
**2.29526462356141e-13**	-1.77	3.16998997160067e-09	3	Svep1
**2.37568044445467e-13**	-0.77374823854	3.28105226183635e-09	3	Oxct1
**2.75465940128779e-13**	-1.09	3.80446009911856e-09	3	Lpl
**2.77039790087478e-13**	-1.11	3.82619654089816e-09	3	Ebf1
**2.95026830394033e-13**	-0.86949065425	4.07461555457199e-09	3	Aldh1a1
**3.4716779434521e-13**	-0.83527491400	4.79473440770169e-09	3	Gstm2
**3.80376402588986e-13**	-0.83275677078	5.25337849615649e-09	3	Cebpb
**4.04968455857197e-13**	-2.20	5.59301934384374e-09	3	Cpxm2
**4.22012941029104e-13**	-0.79045214076	5.82842072855296e-09	3	Itgb5
**4.64967012529239e-13**	-0.76554572112	6.42165941004133e-09	3	Ppap2b
**5.42059163997447e-13**	1.51	7.48637911396875e-09	3	Cdk17
**5.55425826799567e-13**	-1.34	7.67098609392882e-09	3	Slit3
**5.77567815651565e-13**	1.13	7.97678910196377e-09	3	Adam10
**6.10262338379558e-13**	-0.89807781077	8.42833315536007e-09	3	Cd44
**6.98470004176676e-13**	-1.70	9.64656922768407e-09	3	Igsf10
**7.22900684816177e-13**	1.75	9.98398135799622e-09	3	Casp1
**7.26230312381259e-13**	-0.98116027954	1.00299668442976e-08	3	Zeb2
**7.78032973492336e-13**	-1.75	1.07454133969027e-08	3	Atf5
**7.7874734262436e-13**	-0.70726511028	1.0755279548985e-08	3	Tuba1a
**8.17832714078996e-13**	1.23	1.1295087614145e-08	3	Rab11a
**9.29003276934033e-13**	1.85	1.28304642577359e-08	3	Gpr56
**9.54201836517368e-13**	1.93	1.31784815641414e-08	3	Klk8
**1.11288291597737e-12**	-1.50	1.53700259525634e-08	3	F3
**1.22915758987145e-12**	-1.63	1.69758954737147e-08	3	Sfrp2
**1.27158976150332e-12**	0.98878620679	1.75619261961223e-08	3	Efna5
**1.33371921004774e-12**	-1.02	1.84199960099694e-08	3	Serpina3n
**1.34786091673455e-12**	1.61	1.86153071210209e-08	3	Dll1
**1.58375308455001e-12**	1.32	2.18732138507202e-08	3	Hhex
**1.59064137606441e-12**	-1.52	2.19683480448256e-08	3	Pi16
**1.66944180374057e-12**	-1.00	2.3056660751461e-08	3	Sh3d19
**1.97736068090283e-12**	-0.70896023540	2.7309328363949e-08	3	Fkbp7
**2.19955955551403e-12**	2.17	3.03781170212043e-08	3	Klk10
**2.21435513656697e-12**	-0.54450421651	3.05824587911265e-08	3	Osr1
**2.27832167393434e-12**	-1.81	3.14659006387072e-08	3	Srpx
**2.31076432684077e-12**	-0.85884834718	3.19139661179978e-08	3	Ecm2
**2.33982380366241e-12**	-0.66	3.23153065523815e-08	3	Lmo4
**2.56954363176547e-12**	0.98349021368	3.54879670983129e-08	3	Ptprj
**2.61917370340273e-12**	0.71401011701	3.61734080176951e-08	3	Myl12b
**2.77923815879107e-12**	-0.69895641331	3.83840582110634e-08	3	Ckap4
**2.93093898180411e-12**	-1.57	4.04791982776965e-08	3	C7

**4.0331686047234e-12**	0.917606489007	5.57020915998349e-08	3	Pgm5
**4.50568477570496e-12**	1.88	6.22280124372612e-08	3	Myzap
**4.58279187008913e-12**	-0.70584142888	6.3292938517801e-08	3	Ccdc80
**5.02032207758128e-12**	1.14	6.9335668213475e-08	3	Flnb
**5.10129406276913e-12**	1.34	7.04539723009045e-08	3	Exoc6
**5.25290687465165e-12**	-0.94711028191	7.25478968458139e-08	3	Lgals3bp
**5.4360043685962e-12**	2.10	7.50766563346821e-08	3	Gimap4
**5.68530858852978e-12**	2.10	7.85197969161847e-08	3	Prkch
**6.18364735751314e-12**	1.75	8.5402353654614e-08	3	Fzd6
**7.12524088429511e-12**	1.42	9.84067018529998e-08	3	Slco2a1
**7.72422167901861e-12**	2.20	1.06679225608926e-07	3	Lsr
**7.7575230326369e-12**	-1.46	1.07139150603748e-07	3	Xdh
**8.08163412904576e-12**	0.861150932471	1.11615448956251e-07	3	Myh9
**8.14288425776762e-12**	-0.99777927573	1.12461374484029e-07	3	Igsf3
**9.53636727238451e-12**	1.47	1.31706768398902e-07	3	Tspan14
**9.92473031692584e-12**	-1.34	1.37070450407063e-07	3	Hspb8
**1.10451248903288e-11**	1.67	1.52544219860332e-07	3	Adh1
**1.24811466170098e-11**	-1.19	1.72377115927523e-07	3	Cxcl1
**1.53237093764141e-11**	-0.94159225140	2.11635750197655e-07	3	Fcgrt
**1.55604278802618e-11**	-0.80588518213	2.14905069454296e-07	3	Mmp14
**1.93348930645989e-11**	-1.65	2.67034208115176e-07	3	Thbs2
**2.11874756475235e-11**	-0.63444064192	2.92620226167946e-07	3	Gas6
**2.20728852209941e-11**	1.53	3.04848617787149e-07	3	Sox13
**2.28024617216735e-11**	1.52	3.14924798838032e-07	3	Arhgap31
**2.33372863144609e-11**	-0.65504101183	3.2231126128902e-07	3	Ctsl
**2.3671554337898e-11**	-0.77	3.26927836960709e-07	3	Pde5a
**2.39099957131292e-11**	0.786889498501	3.30220950794028e-07	3	Ablim1
**2.44409276525992e-11**	-1.11	3.37553651810048e-07	3	Gja1
**2.52361633336314e-11**	-0.55207483176	3.48536651800784e-07	3	Selm
**2.776891561443E-11**	-1.74	3.83516493550893e-07	3	Matn2
**2.85185959021311e-11**	0.85	3.93870328004332e-07	3	Vcam1
**2.88331900199411e-11**	-1.13	3.98215187365407e-07	3	Ugdh
**3.26622414293985e-11**	2.07	4.51098216381422e-07	3	Arap3
**3.54518395316291e-11**	-1.19	4.89625355771329e-07	3	Fam102b
**3.68958993496711e-11**	-0.75558873564	5.09569265918307e-07	3	Serpine2
**3.85464863433122e-11**	-1.58	5.32365522887485e-07	3	Mmp3
**3.93780426701941e-11**	-0.36479234181	5.43850147318051e-07	3	Rhoq
**4.37202531188709e-11**	-0.71885381131	6.03820415824726e-07	3	C1ra
**4.63135511456096e-11**	-0.92110668541	6.39636454872015e-07	3	Fam46a
**4.89785254227561e-11**	-1.59	6.76442414613684e-07	3	Mlec
**4.90676907897248e-11**	-1.53	6.7767387749689e-07	3	Dnm1
**5.14395353218984e-11**	1.65	7.10431422330738e-07	3	Dhh
**5.46657304006886e-11**	-1.84	7.54988402563911e-07	3	Gpr133
**5.48754779367277e-11**	-1.01	7.57885225784147e-07	3	Fam114a1
**5.67435992462045e-11**	0.49	7.83685849189331e-07	3	Rhob
**6.92103627300865e-11**	-0.52460798763	9.55864319665224e-07	3	Nenf
**6.97014900731369e-11**	-1.26	9.62647279400094e-07	3	Slc6a6
**7.23469665261078e-11**	-0.71	9.99183954692074e-07	3	Herpud1
**7.75512886940166e-11**	-1.08	1.07106084815306e-06	3	Adamtsl3
**8.35019129418981e-11**	-1.62	1.15324491964055e-06	3	Il6
**8.48378231711927e-11**	-1.22	1.17169517581734e-06	3	1700025G04I
**9.20693511075453e-11**	-1.98	1.27156980814631e-06	3	Inmt
**9.39006437319387e-11**	-0.64779936320	1.29686179058181e-06	3	Mfap5
**1.05767471984879e-10**	-0.74876826035	1.46075455558317e-06	3	Tmed3
**1.23477619981459e-10**	1.31	1.70534940956392e-06	3	Plxnd1
**1.25327637078346e-10**	-0.82333193093	1.73089999568904e-06	3	Errfi1

**1.27714623019406e-10**	-2.05	1.76386665852101e-06	3	Fmod
**1.34512035770067e-10**	-0.54016960418	1.85774572602039e-06	3	Snhg18
**1.35274575644332e-10**	1.89	1.86827716422387e-06	3	C130074G19
**1.42838778108575e-10**	-0.74467989628	1.97274636445752e-06	3	Mmp2
**1.6926337300341e-10**	-1.72	2.33769644455009e-06	3	Naalad2
**1.78057505483544e-10**	-0.74030785687	2.45915220823322e-06	3	Lamc1
**1.79656911970828e-10**	-0.79	2.4812416112291e-06	3	Pi15
**1.92692217704255e-10**	-1.72	2.66127221871346e-06	3	Gabra3
**2.0813361735986e-10**	-0.91674819908	2.87453338935703e-06	3	Cxcl12
**2.21085015105895e-10**	-1.10	3.05340514362751e-06	3	Runx1t1
**2.35621507841156e-10**	1.35	3.25416864479421e-06	3	Adra2a
**3.03358744105545e-10**	-1.32	4.18968761484168e-06	3	Man2a2
**3.08388691757223e-10**	1.43	4.25915622185901e-06	3	Fgd5
**3.12493473880964e-10**	1.36	4.31584736776999e-06	3	Ptprm
**3.14544226327936e-10**	-1.58	4.34417030981513e-06	3	Plce1
**3.18461462011043e-10**	1.01	4.39827125183451e-06	3	Smad6
**3.22716549078174e-10**	0.58	4.45703825931866e-06	3	Ctnnb1
**3.58893565785394e-10**	-1.21	4.95667903706208e-06	3	Mpp6
**3.63985361645101e-10**	1.48	5.02700182968049e-06	3	Ctla2a
**3.82236448365243e-10**	-0.73183328692	5.27906758837237e-06	3	Mpc2
**4.04153177034746e-10**	-0.60498579846	5.58175952802687e-06	3	Rnase4
**4.0834640485468e-10**	1.10	5.63967219744799e-06	3	Spon1
**4.19342384538305e-10**	-0.80024495758	5.79153767285853e-06	3	Prnp
**4.45510050461925e-10**	-1.16	6.15293930692965e-06	3	Pid1
**4.79043423725474e-10**	-1.68	6.61606872507252e-06	3	Adamts15
**5.03526452550877e-10**	-0.58355182600	6.95420383618016e-06	3	Mxra8
**5.15379543179222e-10**	-0.85955400568	7.11790687084824e-06	3	Antxr1
**5.64199115155969e-10**	-1.55	7.79215397941909e-06	3	Steap4
**5.64417126656551e-10**	1.36	7.79516493625362e-06	3	Mkl2
**5.80242086521009e-10**	1.14	8.01372345694166e-06	3	Serpinb6b
**6.34364120523796e-10**	-0.83974507428	8.76120286855414e-06	3	Gstm5
**6.68187059427746e-10**	-1.84	9.22833147775659e-06	3	Dact1
**6.89191337253384e-10**	-0.69616135655	9.51842155880649e-06	3	Zcchc24
**7.2093045339196e-10**	-0.46838304317	9.95677049179636e-06	3	Timp2
**7.31769699350625e-10**	-0.68507509301	1.01064713177315e-05	3	Mpdz
**7.73889559614134e-10**	1.57	1.06881887078308e-05	3	Myrip
**8.03703095403004e-10**	1.17	1.10999434506109e-05	3	Cd2ap
**8.19161705103629e-10**	0.88132855942[illegible]	1.13134423091862e-05	3	Cdh13
**8.38055912119714e-10**	-1.27	1.15743902022854e-05	3	Mmp19
**8.45122840138891e-10**	-1.37	1.16719915451582e-05	3	Itih5
**8.48820445381671e-10**	-0.77589899148	1.17230591711663e-05	3	Mdh1
**8.89467726274861e-10**	1.44	1.22844387675821e-05	3	Tspan6
**9.08657683223451e-10**	0.93826929131[illegible]	1.25494712629991e-05	3	Efcab4a
**1.01202236318096e-09**	-1.63	1.39770408578923e-05	3	Gpm6b
**1.04686303317291e-09**	-0.68038425006	1.4458225351151e-05	3	4930523C07[illegible]
**1.05602023293769e-09**	-1.30	1.45846954371024e-05	3	Fstl3
**1.09094359000387e-09**	-0.76179337150	1.50670219215435e-05	3	Lrrc61
**1.09982994636193e-09**	-0.34451730822	1.51897513892046e-05	3	Atp6v0b
**1.10108077636093e-09**	1.24	1.52070266023207e-05	3	Nt5e
**1.13901343174251e-09**	-0.57686393501	1.57309145057958e-05	3	Nptn
**1.1454724522377e-09**	-0.33238851859	1.58201200378549e-05	3	Ctsb
**1.20188312334157e-09**	-0.63865143515	1.65992078164705e-05	3	Cnpy2
**1.20819562798874e-09**	-1.61	1.66863898181525e-05	3	Abca9
**1.22399319888888e-09**	-1.57	1.69045700698543e-05	3	Qpct
**1.41476975437829e-09**	-0.42745095050	1.95393850777185e-05	3	Map1lc3a
**1.54385454590377e-09**	-0.95166213393	2.13221751334769e-05	3	Papss2

**1.60804018181232e-09**	1.63	2.22086429510099e-05	3	Rasgrf2
**1.62509888863042e-09**	-1.30	2.24442407508747e-05	3	C1qtnf7
**1.65703314803997e-09**	-2.00	0.00	3	C2
**1.67457036674519e-09**	0.540761938975	2.31274913351179e-05	3	Serinc3
**1.75813585978035e-09**	1.35	2.42816143594265e-05	3	Il15
**1.9133840068177e-09**	-0.60190523872	2.64257465181593e-05	3	Bloc1s1
**1.92912474156719e-09**	-1.47	2.66431418057844e-05	3	Gpnmb
**1.93416365627876e-09**	-0.79720027317	2.6712734256866e-05	3	Adamtsl5
**2.10342138005242e-09**	2.11	2.9050352679904e-05	3	Klhl6
**2.16348809716719e-09**	1.71	2.9879934109976e-05	3	Calcrl
**2.41782432555823e-09**	-0.85861127870	3.33925717602847e-05	3	Add3
**2.49187233017785e-09**	1.51	3.44152487520863e-05	3	Tmem2
**2.49408889295765e-09**	-1.32	3.44458617006381e-05	3	Slc7a2
**2.52331024182156e-09**	-1.40	3.48494377497975e-05	3	Clmp
**2.56242567847724e-09**	1.23	3.53896610454492e-05	3	Tspan7
**2.62729820037578e-09**	-0.52202299575	0.00	3	Lamb2
**2.63074850099891e-09**	0.934569259046	3.6333267547296e-05	3	Lpar6
**2.76340887533586e-09**	-1.46	3.81654399772636e-05	3	Tmem119
**2.98480125731938e-09**	-0.77290702779	4.1223090164838e-05	3	Cped1
**3.06855791968202e-09**	-0.75	4.23798534287284e-05	3	Rora
**3.11935569768653e-09**	-0.64292514855	4.30814215407487e-05	3	Glis2
**3.12421417738204e-09**	1.52	4.31485220038234e-05	3	Krt18
**3.43363119447711e-09**	-1.65	4.74218804269234e-05	3	Dio2
**3.49622925145394e-09**	0.77	4.82864221918304e-05	3	Cdc42ep3
**3.56275210951915e-09**	-1.02	4.9205169384569e-05	3	Tcea3
**3.62158413385333e-09**	-0.50054455612	5.00176984726483e-05	3	Meg3
**3.74757633920539e-09**	1.91	5.17577768207657e-05	3	Rgs3
**3.75106929798625e-09**	-1.95	5.1806018074488e-05	3	Tmem100
**3.92816053234046e-09**	-0.63374232417	5.4251825112154e-05	3	Vkorc1
**4.00498609772854e-09**	-1.27	5.53128629957288e-05	3	Lbp
**4.04382163556475e-09**	0.835055097876	5.58492206087848e-05	3	Fxyd5
**4.33080517704585e-09**	-1.54	5.98127503001802e-05	3	Lrrc17
**4.52543767810985e-09**	1.95	6.25008197723751e-05	3	Gimap6
**4.55004502506126e-09**	-0.95636813668	6.28406718411211e-05	3	Sema3c
**4.55615709620826e-09**	-0.43253355645	6.29250856557323e-05	3	Gm2a
**4.63782822249888e-09**	-1.22	6.40530455809321e-05	3	Rab34
**4.65307161855069e-09**	0.428896065649	6.42635721238036e-05	3	Rps3
**4.87512280231363e-09**	-1.03	6.73303210227536e-05	3	H6pd
**4.90206110959841e-09**	1.68	6.77023659846636e-05	3	Adcy4
**4.91646865786623e-09**	-1.62	6.79013486337905e-05	3	Hsd11b1
**4.94548023102449e-09**	-0.61944187222	6.83020274706792e-05	3	Dlc1
**5.08148809847723e-09**	-0.43	7.0180432128069e-05	3	Fuca1
**5.254191739646E-09**	-0.47953358780	7.25656421162509e-05	3	Csrp2
**5.61076274291713e-09**	-0.40876755442	7.74902442424284e-05	3	Cebpd
**5.63094105874563e-09**	-0.79496321201	7.77689269623359e-05	3	Ctdsp2
**6.10532881363949e-09**	-1.00	8.43206962451751e-05	3	Slc12a4
**6.4865543106961e-09**	-0.59410255357	8.95858015850239e-05	3	Sod2
**6.55449528533465e-09**	-0.75897884671	9.05241343857569e-05	3	Fnip1
**6.68496868740438e-09**	-1.50	9.23261025417419e-05	3	Thy1
**6.84450267708572e-09**	-0.62579710211	9.45294264732309e-05	3	Plod2
**7.0373285634008e-09**	1.96	9.71925447891285e-05	3	Arhgef15
**7.4180541405259e-09**	-1.21	0.000102450745734803	3	Sox9
**7.56765928246305e-09**	-0.85082009682	0.000104516942350097	3	Creb3l1
**7.62470952737671e-09**	-1.34	0.0001053048632826	3	Pamr1
**8.42025667199106e-09**	-1.75	0.000116292164896868	3	Lamc3
**8.4763241157822e-09**	1.34	0.000117066512363068	3	Dennd3

**8.52083540876338e-09**	-0.91337225394	0.000117681257830431	3	Slc16a2
**8.58059666050473e-09**	0.365516248467	0.000118506620478231	3	Cpe
**9.17276047854630e-09**	-1.54	0.000126684994969203	3	Adcy7
**9.24058852784127e-09**	-1.69	0.000127621768158016	3	Fbln7
**1.01763090958138e-08**	1.12	0.000140545004922285	3	Sema3f
**1.11231557254994e-08**	1.65	0.000153621903724872	3	Enpp6
**1.1376093002268e-08**	-0.68047502500	0.000157115220454323	3	Ckb
**1.1417053703243e-08**	-0.60351823501	0.000157680928695489	3	Hexa
**1.15456093353301e-08**	-0.73289718342	0.000159456410530244	3	Zak
**1.17141891331694e-08**	-0.69	0.000161784666118203	3	Tnxb
**1.33856094201238e-08**	1.63	0.00018486865170133	3	Lmo7
**1.34690063116316e-08**	-0.66630634140	0.000186020446169944	3	Nox4
**1.37306033017165e-08**	1.20	0.000189633362200006	3	Fli1
**1.46215673096357e-08**	-0.68	0.000201938466113379	3	Tnfrsf12a
**1.47188553668271e-08**	-0.95137672633	0.000203282111471249	3	Prickle1
**1.47754650571196e-08**	-0.86449954484	0.000204063947903879	3	Atp6v0d1
**1.49291214888795e-08**	-1.06	0.000206186096882915	3	Kdelr3
**1.58771011235091e-08**	-2.13	0.000219278643616784	3	Heyl
**1.65454382853986e-08**	-0.99	0.00022850904815964	3	Prss23
**1.65828325754415e-08**	0.734506354527	0.000229025500699423	3	Ace
**1.66508942170318e-08**	-0.51986786348	0.000229965500031426	3	Kank2
**1.75336495468353e-08**	-0.48	0.000242157233891342	3	Mrpl52
**1.76466330175947e-08**	-0.67510204573	0.000243717648606	3	Gprc5b
**1.80277781293366e-08**	-1.08	0.000248981643744268	3	Adarb1
**1.85664860045107e-08**	1.57	0.000256421738208298	3	Kank3
**1.94120166896913e-08**	-1.50	0.000268099362501326	3	Dtx4
**1.98561257900111e-08**	1.87	0.000274232953285844	3	Golm1
**2.11213610355615e-08**	1.24	0.000291707117262141	3	4931406P16R
**2.14170953050818e-08**	-1.03	0.000295791503258484	3	Samd4
**2.17748654113235e-08**	-1.43	0.000300732666195789	3	C1qtnf2
**2.20168257218997e-08**	1.13	0.000304074380045157	3	Elk3
**2.23573958469129e-08**	-1.67	0.000308777994041714	3	Apod
**2.29560192689514e-08**	-0.61161558777	0.000317045582123488	3	Tmem106b
**2.30263438419581e-08**	-0.47576261731	0.000318016834801283	3	Sec61b
**2.31726981343746e-08**	-1.02	0.000320038133933848	3	Igsf8
**2.63133798354895e-08**	2.04	0.000363414088907946	3	Tspan13
**2.65482061591742e-08**	-1.26	0.000366657275264354	3	Pdpn
**2.70957821845427e-08**	2.35	0.00037421984775072	3	Coch
**2.72420327138587e-08**	-0.87222018744	0.000376239713811103	3	Sept8
**2.85997728338005e-08**	-1.48	0.000394991462607618	3	Ssc5d
**2.87913734952404e-08**	0.519000231332	0.000397637659342765	3	Hid1
**2.91306159218232e-08**	0.959403657079	0.0004023229364963	3	Eng
**2.93153042480962e-08**	1.27	0.000404873666970457	3	Hoxa5
**2.94404844667782e-08**	1.08	0.000406602530970674	3	Atp2a3
**3.01892504171806e-08**	-1.36	0.000416943737511682	3	Kcnj8
**3.07186976420405e-08**	-0.78253199757	0.000424255933134221	3	Mfap2
**3.08035366647931e-08**	-0.98620354743	0.000425427644877457	3	Rnd3
**3.31384294871101e-08**	-0.76108086702	0.000457674849646478	3	Sec24d
**3.60574787535124e-08**	-0.56649039297	0.00049798983906476	3	Hivep2
**3.75931074061067e-08**	-0.54565861982	0.000519198406385739	3	Ergic3
**3.80081543924394e-08**	0.557919160784	0.00052493062031398	3	Crip2
**3.810753301274E-08**	-0.84290388494	0.000526303138438952	3	Ptprd
**3.86158710635399e-08**	0.40	0.00053332379525855	3	Aplp2
**3.94877499667478e-08**	-1.25	0.000545365314790754	3	Stk17b
**3.95612624179052e-08**	-0.61478026621	0.000546380595253689	3	Uqcc2
**4.01415419254514e-08**	-1.72	0.000554394835532409	3	Ccl7

**4.13794212911411e-08**	-1.51	0.000571491187451949	3	Gas7
**4.17967849476534e-08**	-0.38	0.000577255396912041	3	Cox7b
**4.30617219305939e-08**	-0.42351361841	0.000594725441583432	3	Cd63
**4.313882406389E-08**	-0.54	0.000595790299146384	3	Ugp2
**4.37168835751541e-08**	-0.60864428776	0.000603773879056454	3	Dnajc3
**4.65456664043361e-08**	-0.28534728375	0.000642842198710285	3	Fth1
**4.73233638743419e-08**	-0.59587323452	0.000653582978468535	3	Pfkp
**4.75602332556109e-08**	-1.66	0.000656854381493242	3	Aff3
**4.85518409813417e-08**	-0.38272411911	0.00067054947579331	3	Gucy1a3
**4.86636962321899e-08**	-0.31073810196	0.000672094308662775	3	Arhgap21
**5.01272347077704e-08**	-0.46402845895	0.000692307238549017	3	Lamp2
**5.01377116560317e-08**	1.40	0.000692451935681454	3	Fgfr3
**5.10018257701881e-08**	0.95012903788	0.000704386215712069	3	Sgk1
**5.17138500137105e-08**	-0.75027041060	0.000714219982539356	3	Crebbp
**5.4737291325756e-08**	-0.40591106856	0.000755976730500016	3	Gns
**5.55522761256663e-08**	-0.62362599966	0.000767232485571577	3	Chchd1
**5.56936556721021e-08**	-0.67740687057	0.000769185078487402	3	Polr2f
**6.21532731985999e-08**	-0.53678240602	0.000858398856145863	3	Anapc13
**6.22291020351362e-08**	-0.69601576263	0.000859446128207266	3	Ltbp2
**6.30921533833752e-08**	1.49	0.000871365730377795	3	Procr
**6.98100490606589e-08**	-0.30274539117	0.000964146587576761	3	Sparc
**7.02171061720788e-08**	-1.08	0.00096976845334258	3	Ank2
**7.19014918630726e-08**	-1.12	0.000993031504120896	3	Pcsk6
**8.10572520555042e-08**	1.99	0.00111948170813857	3	Sp100
**8.30346968331779e-08**	-0.28669237165	0.00114679219796302	3	Cstb
**8.39276255099266e-08**	-0.34159467009	0.0011591244359176	3	Egr1
**8.48684115614589e-08**	-0.41728768796	0.00117211763207531	3	Reep5
**8.533702648752E-08**	-0.55100853619	0.00117858967281914	3	Tmem45a
**9.95545744757898e-08**	-0.58273712373	0.00137494822808513	3	Ilk
**1.01750949826698e-07**	-1.30	0.00140528236805652	3	Tppp3
**1.02691882682566e-07**	-1.40	0.00141827759172892	3	Ccl19
**1.03287550230629e-07**	-0.99	0.00142650435623522	3	Arl3
**1.04825595993759e-07**	-1.54	0.00144774630626981	3	Ntf3
**1.06483635487588e-07**	-0.64764243043	0.00147064548971908	3	Apoa1bp
**1.13357756210105e-07**	-0.53485990135	0.00156558397101776	3	Nfkb1
**1.1382695999036e-07**	-1.63	0.00157206414442687	3	Sfrp4
**1.2087753981514e-07**	-1.08	0.0016694397023869	3	Ndufa4l2
**1.2184024809963e-07**	-0.48115474061	0.00168273566650399	3	Mrpl33
**1.33549021417587e-07**	-0.41390841765	0.0018444455347983	3	Mia3
**1.34013143129861e-07**	-0.58232101276	0.00185085551976651	3	C1qtnf1
**1.34248925030807e-07**	-0.33846288553	0.00185411190360047	3	Fkbp10
**1.34514033438776e-07**	-0.73581788510	0.00185777331582294	3	Fzd1
**1.35074393476133e-07**	0.77221509205	0.00186551244829887	3	Ly6c1
**1.36840997252641e-07**	-1.30	0.00188991101305623	3	Zfp703
**1.50421910556771e-07**	-1.46	0.00207747700669957	3	Col4a6
**1.57322137451671e-07**	2.04	0.00217277604034503	3	Ptprr
**1.57349281346479e-07**	-0.36577566479	0.00217315092467622	3	Sod3
**1.60692715720368e-07**	-1.77	0.002219327096814	3	Has1
**1.62087751722176e-07**	-1.38	0.00223859393903498	3	Fgf7
**1.64516503380073e-07**	1.18	0.00227213742818218	3	Dock9
**1.66205933819028e-07**	1.35	0.00229547015197459	3	Etl4
**1.68388062068562e-07**	1.15	0.00232560752522891	3	Cenpt
**1.71319029543141e-07**	1.09	0.00236608711702032	3	Mgst3
**1.7633714167896e-07**	-1.18	0.00243539226372811	3	Slc44a1
**1.87835622481756e-07**	-1.91	0.00259419778209553	3	Gpc3
**1.88020405427552e-07**	1.76	0.00259674981935992	3	Isl1

**1.9824345820685e-07**	-0.43590238946	0.00273794040129481	3	Ywhaq
**1.98861379676883e-07**	-1.45	0.00274647451471743	3	Meox2
**1.99117275338981e-07**	-1.45	0.00275000868970666	3	Prrx2
**2.17324279748321e-07**	-1.45	0.00300146562760406	3	Dse
**2.1993234094907e-07**	-0.82771092709	0.00303748556084761	3	Loxl2
**2.35188299328028e-07**	0.532143783878	0.00324818560201939	3	Tpm3
**2.35692740553592e-07**	0.73	0.00325515243978566	3	Ifngr1
**2.37138724833895e-07**	-1.45	0.00327512292868093	3	Fam26e
**2.50900551847476e-07**	-0.74638474615	0.00346518752156549	3	Anxa4
**2.51160583205127e-07**	-0.55019360551	0.00346877881464601	3	Tmem43
**2.64303447488342e-07**	-0.77689891343	0.00365029491326149	3	Boc
**2.68917964068718e-07**	-0.61467452636	0.00371402600175306	3	Camk2n1
**2.69248541263759e-07**	1.34	0.00371859160339377	3	Arhgdib
**2.7224185653329e-07**	-1.65	0.00375993228058127	3	Bmper
**2.85708890130361e-07**	-0.92574805143	0.00394592548159042	3	Penk
**2.89019687685994e-07**	-0.82674879944	0.00399165090663126	3	Daam1
**2.97335825422316e-07**	-0.61731478048	0.00410650508490761	3	Nudt9
**3.07480712357511e-07**	1.23	0.00424661611836958	3	Plxna2
**3.12001255214288e-07**	-0.99320672710	0.00430904933576453	3	Alcam
**3.20332225193215e-07**	-0.70	0.00442410836214349	3	Ccdc107
**3.35455017871336e-07**	-0.51282666831	0.00463296925182102	3	Copz2
**3.37907099180342e-07**	-0.41792918295	0.0046668349467797	3	Bmpr1a
**3.45378577381418e-07**	1.02	0.00477002353221477	3	Dock6
**3.4560888603352e-07**	0.685265238501	0.00477320432500894	3	Nrp1
**3.46923003250044e-07**	-0.33830975789	0.00479135359788635	3	Tenm3
**3.51092338614259e-07**	-1.61	0.00484893628860153	3	Clip4
**3.58844001431229e-07**	-0.90293462444	0.0049559945037667	3	Por
**3.63587859994039e-07**	-1.25	0.00502151193437768	3	Lpgat1
**3.64823149017441e-07**	-1.49	0.00503857251107988	3	Dpp7
**3.76761791162711e-07**	0.310294451115	0.0052034570977482	3	Lars2
**3.85029597218654e-07**	0.56	0.00531764376718683	3	Zyx
**3.91227682074572e-07**	-1.74	0.00540324551713192	3	Tcf21
**3.98457794299738e-07**	-0.42103318044	0.00550310059707369	3	Cyba
**4.03164969884384e-07**	1.17	0.00556811139907323	3	Ssfa2
**4.19785918515319e-07**	-0.80286073469	0.00579766332061507	3	Jam3
**4.26256940559451e-07**	-0.71076585785	0.00588703460606658	3	Lbh
**4.36802488994641e-07**	-0.64	0.00603267917550498	3	Dip2c
**4.41718535362893e-07**	-0.40	0.00610057469189692	3	Pmepa1
**4.57878549668356e-07**	-0.40891946964	0.00632376064946967	3	Map1b
**4.59524658519607e-07**	0.604748834226	0.0063464950588143	3	2900060B14I
**4.67397202539338e-07**	-0.73092979685	0.0064552227642708	3	Steap3
**4.698380850201E-07**	-1.63	0.0064889337922126	3	Wnt5a
**4.71323061708549e-07**	-0.41542073531	0.00650944280525677	3	Fkbp2
**4.89680695288025e-07**	-1.62	0.00676298008262291	3	Myoc
**4.94737180267942e-07**	1.25	0.0068328151966805	3	Cpd
**4.96848100823831e-07**	-0.78224508996	0.00686196912047792	3	Fxyd6
**5.16487075528328e-07**	-0.91311882793	0.00713320300012173	3	Pofut2
**5.18868069404623e-07**	-0.38	0.00716608690654724	3	Ndufa11
**5.47731689003951e-07**	-0.59098955721	0.00756472235683357	3	Wipf1
**5.49427104698477e-07**	1.98	0.00758813774299066	3	Flt4
**5.63145403016539e-07**	0.525339794736	0.00777760116106142	3	Atrx
**5.71393059274206e-07**	-0.34207710591	0.00789150954163606	3	Sqstm1
**5.73463061951753e-07**	0.636233140233	0.00792009834861567	3	Rnd1
**5.73995009404895e-07**	-0.58613161657	0.007927445074891	3	Tmed1
**6.00347533183124e-07**	-0.52942101375	0.00829139978079213	3	Etfa
**6.07773928457052e-07**	-1.23	0.00839396572592034	3	Cdon

**6.14272519633976e-07**	-0.50	0.00848371776866484	3	Ier5
**6.14344092748066e-07**	0.902114511438	0.00848470626494354	3	9430020K01I
**6.17886342281522e-07**	-0.35247743367	0.0085336282732501	3	Atf4
**6.21889825558996e-07**	-0.63381430174	0.0085889203807953	3	Cnih4
**6.26702621332772e-07**	-0.42967246652	0.00865538990322691	3	Atp2a2
**6.35276289745791e-07**	-1.06	0.00877380083767912	3	Podn
**6.79333484888877e-07**	-0.40896349518	0.00938227475980028	3	Dag1
**6.79992151718855e-07**	-0.85686678086	0.0093913716073891	3	Dact3
**6.89875007334175e-07**	-0.88054471382	0.00952786372629229	3	Cacna2d1
**6.99450364662258e-07**	-0.77402397134	0.00966010898635045	3	Grb14
**7.19667265746924e-07**	-0.41690842199	0.00993932460723077	3	Atraid
**7.23496956695552e-07**	-0.41651298638	0.00999221646892227	3	Atp6ap2
**7.25217389227124e-07**	-1.03	0.0100159773626158	3	Clip3
**7.37446341876104e-07**	-0.71906842684	0.0101848714276509	3	Nfil3
**7.46312573216376e-07**	-0.67612363343	0.0103073229486914	3	Higd1a
**7.66058114124162e-07**	-0.56118976987	0.0105800286141688	3	Slc1a5
**7.77060534989774e-07**	0.33	0.0107319830487438	3	Rps20
**8.08263003680702e-07**	-0.37	0.0111629203438342	3	Serpinh1
**8.16638078833195e-07**	1.28	0.0112785885067653	3	Plk2
**8.39244202875698e-07**	-1.73	0.0115908016859163	3	Gsta3
**8.40641027803395e-07**	-0.44049136386	0.0116100932349927	3	Gstm1
**8.54718343293896e-07**	-0.94970823094	0.011804515039232	3	Rtn4rl1
**8.63544991254892e-07**	-0.73504118603	0.0119264198742213	3	Scarf2
**8.66888376780582e-07**	-0.31433869317	0.0119725953717166	3	Plbd2
**8.91506629017637e-07**	-0.36934423871	0.0123125980533626	3	Dbi
**8.9569722785277e-07**	-1.28	0.0123704744138746	3	Ldhb
**9.1125857152999e-07**	-1.08	0.0125853921314007	3	Plekhh2
**9.11477489327051e-07**	-1.06	0.0125884156050959	3	Sh3kbp1
**9.24852349056286e-07**	-0.46581417567	0.0127731357928164	3	Smdt1
**9.44299194257161e-07**	-0.36681348092	0.0130417161718857	3	Acap2
**9.60083513793596e-07**	-0.77453239728	0.0132597134090034	3	Tbc1d2b
**9.62447813161653e-07**	-0.57338963525	0.0132923667475756	3	Ppt1
**9.67018565245279e-07**	-0.64252995068	0.0133554934046026	3	Cxxc1
**9.88615535575422e-07**	1.39	0.0136537691618321	3	Glce
**9.94553441369038e-07**	-0.60402261350	0.0137357775787478	3	Sgcb
**1.00930265993655e-06**	2.03	0.0139394790363837	3	Emcn
**1.02042229868035e-06**	-0.47511860208	0.0140930523670744	3	Tiparp
**1.03572846968398e-06**	-0.34166231736	0.0143044458948054	3	Sdc4
**1.04312964334531e-06**	-0.98515986341	0.0144066635042421	3	3632451O06I
**1.06305590851302e-06**	-0.94225818278	0.0146818651524734	3	Smo
**1.16545594068211e-06**	-0.79618029086	0.0160961119967606	3	Pde4dip
**1.18419572964721e-06**	-0.40333797102	0.0163549272221576	3	Vezf1
**1.1867642519675e-06**	-0.66390049433	0.0163904010839232	3	Lipa
**1.19762455033753e-06**	-0.54205551396	0.0165403926647116	3	Phpt1
**1.19781897815323e-06**	-0.32547780119	0.0165430779072743	3	Creg1
**1.20272472098488e-06**	1.11	0.0166108311215222	3	Fnbp1l
**1.21499526196961e-06**	-0.42265554832	0.0167802995630623	3	Rasl11b
**1.21682301017245e-06**	-1.77	0.0168055425934917	3	Ebf2
**1.27175279013599e-06**	-0.48178931769	0.0175641777845681	3	Nav1
**1.27805333500297e-06**	-0.84514236413	0.0176511946097261	3	Angptl2
**1.45908763312081e-06**	-1.57	0.0201514593010315	3	Ism1
**1.46727806704045e-06**	-0.48271562606	0.0202645773838956	3	Ssr4
**1.4933213375798e-06**	-0.28704984450	0.0206242609933146	3	Zc3h11a
**1.49597693349228e-06**	0.345234114083	0.0206609374284619	3	Ly6c2
**1.51185059276104e-06**	-0.91712725005	0.0208801685366228	3	Dapk1
**1.51320481510527e-06**	-0.47523547132	0.0208988717014189	3	Nfix

**1.53390949209868e-06**	0.746284306482	0.0211848239953748	3	Azin1
**1.54658274745121e-06**	-0.84222548728	0.0213598543250486	3	Tubb6
**1.55284079077794e-06**	-0.58668710112	0.0214462841614342	3	Ift81
**1.61258985035484e-06**	-0.65923631957	0.0222714784232507	3	Syne1
**1.63073888064644e-06**	-0.33642773974	0.0225221346806079	3	Grina
**1.65764073436812e-06**	-0.47821201832	0.0228936761823581	3	Lsm4
**1.72500583635036e-06**	-1.12	0.0238240556058348	3	Ndrg4
**1.76524329981993e-06**	-0.98912760470	0.0243797752138131	3	Atp9a
**1.86405305902116e-06**	-0.82067755777	0.0257444367981413	3	Tnfsf12
**1.87619899097772e-06**	1.57	0.0259121842643933	3	Gngt2
**1.87875085776713e-06**	-0.52157783629	0.0259474280966218	3	Sidt2
**1.95136930712063e-06**	-1.00	0.026950361500643	3	Runx1
**1.95140258501222e-06**	-0.39	0.0269508211016037	3	Nudt4
**1.95819397206824e-06**	-0.44163934019	0.0270446169482344	3	Sec31a
**1.97289822076115e-06**	1.33	0.0272476973269322	3	Unc13b
**2.0045006425103e-06**	-1.24	0.0276841583737098	3	Smim1
**2.10048087131728e-06**	-0.53580881677	0.029009741313763	3	Myh10
**2.1683262724947e-06**	1.05	0.0299467541494243	3	Hey2
**2.25261944405728e-06**	-1.30	0.031110927141875	3	Tspan11
**2.26876876408109e-06**	-0.99510219460	0.0313339654007239	3	Fto
**2.27401585723772e-06**	-0.39524174415	0.0314064330043101	3	Serp1
**2.3417098112894e-06**	-0.77863587490	0.032341354203718	3	Prkab2
**2.36387505359972e-06**	1.62	0.0326474783652658	3	Ica1
**2.36526810151596e-06**	-1.62	0.0326667177500369	3	Sult5a1
**2.41081745234991e-06**	-0.45401656561	0.0332957998344046	3	Nr3c1
**2.44466905602826e-06**	-1.35	0.0337633243328063	3	Pdzrn3
**2.47083005645806e-06**	-1.55	0.0341246339097423	3	Ntrk3
**2.48629887060217e-06**	-1.18	0.0343382737018866	3	Deptor
**2.49141142500383e-06**	-0.80971340292	0.0344088831907279	3	B4galt5
**2.52108456856489e-06**	-0.44596990713	0.0348186989764497	3	Scarb2
**2.59975695268286e-06**	-1.27	0.035905243273503	3	Fzd2
**2.63321688134604e-06**	-1.48	0.0363673583482701	3	5031426D15R
**2.7120971298471e-06**	-0.47983695792	0.0374567734603183	3	Lpp
**2.84076286590818e-06**	-0.81474735726	0.0392337759410578	3	Adm
**2.843515566225E-06**	-0.70127742490	0.0392717934851335	3	Stx4a
**2.86609865601716e-06**	-0.43175578709	0.039583688538253	3	Tpi1
**2.89484190536443e-06**	-1.34	0.0399806615549881	3	Mn1
**2.92299503977791e-06**	-0.63373970823	0.0403694844943727	3	S100a4
**2.9292968463578e-06**	-0.27583278899	0.0404565187450475	3	Zbtb20
**2.98771268455647e-06**	-0.89918261702	0.0412632998864094	3	Slc25a24
**2.99158357241543e-06**	1.42	0.0413167607186295	3	Afap1l2
**3.02040012760239e-06**	-1.39	0.0417147461623167	3	Cdk14
**3.02553310793532e-06**	0.759226745997	0.0417856377536947	3	Sgms1
**3.21078659740357e-06**	-0.40691181724	0.0443441736967407	3	Ndufa5
**3.2595797600832e-06**	-1.62	0.0450180560665091	3	Gpr153
**3.29433402523808e-06**	-0.93695724093	0.0454980472225631	3	Map3k8
**3.46148733417548e-06**	0.294527685670	0.0478066015722976	3	B2m
**3.49097001559186e-06**	-1.55	0.0482137868853392	3	Abhd11os
**3.58975932413355e-06**	-1.26	0.0495781660256084	3	Abca8b
**3.62718016522078e-06**	-0.54341435183	0.0500949852618643	3	Marcks
**3.64079301955455e-06**	-1.43	0.0502829923930679	3	Figf
**3.66025874123926e-06**	-0.29459379615	0.0505518334752554	3	Ctgf
**3.98108424620504e-06**	0.993740347836	0.0549827545243377	3	Epha4
**3.9857810412331e-06**	-0.90526145716	0.0550476219604703	3	Gnao1
**4.21684492323169e-06**	-1.52	0.0582388452347529	3	Glt8d2
**4.24380678239717e-06**	-0.55058166304	0.0586112154716874	3	Tars

**4.28790670488916e-06**	1.04	0.0592202795012242	3	Ptpn14
**4.40800923090032e-06**	-0.58702518328	0.0608790154879644	3	Kifap3
**4.40900079610145e-06**	-1.07	0.0608927099949571	3	Cdr2
**4.45267603871077e-06**	-1.44	0.0614959087706344	3	Kcne4
**4.50045235882319e-06**	-1.52	0.062155747527707	3	Ccbe1
**4.58213573947908e-06**	-0.34692131601	0.0632838766979456	3	Nab1
**4.58665639828435e-06**	-0.50942174892	0.063346311516705	3	Bag3
**4.58927773977228e-06**	-0.33840615737	0.0633825148639949	3	Ak3
**4.63528777289946e-06**	-1.12	0.0640179594315144	3	Ccng1
**4.76875101650953e-06**	-0.50521446620	0.0658612202890131	3	Derl1
**4.81021389743328e-06**	-0.65051602661	0.066433864137451	3	Chpt1
**4.94731179351759e-06**	-1.49	0.0683273231802714	3	Htra4
**4.97142946824011e-06**	-0.42165209063	0.0686604123858641	3	Stt3b
**4.99587830211917e-06**	-0.79	0.0689980752305678	3	Ptgs2
**5.02347521499651e-06**	-0.88311493809	0.0693792161943168	3	Cdh11
**5.07473790693712e-06**	-0.35833385862	0.0700872052327086	3	Rpn2
**5.21599943919265e-06**	-0.49606971628	0.0720381682546897	3	9530068E07R
**5.26171345931803e-06**	-0.47738162704	0.0726695245866413	3	Morf4l2
**5.33962084845744e-06**	-0.37066235262	0.0737455035380458	3	Sepp1
**5.36465119271025e-06**	1.71	0.0740911976225213	3	Clec1a
**5.51378584262364e-06**	-0.79729028856	0.0761508962724751	3	Eml1
**5.62880803075655e-06**	-0.75282470152	0.0777394677127787	3	Ptgfrn
**5.8497847347089e-06**	-1.41	0.0807913769710646	3	Fap
**5.86317101953167e-06**	-0.42049020761	0.0809762549507519	3	Hspe1
**5.95431253155641e-06**	-0.54	0.0822350103733256	3	Asph
**5.95509273641281e-06**	0.765678351156	0.0822457857825974	3	Ppp3ca
**6.04461428897128e-06**	-0.55152157157	0.0834821679449823	3	Mbd3
**6.09043947050489e-06**	-0.39206887639	0.084115059527143	3	Gpx7
**6.20448887608374e-06**	-1.03	0.0856901958675925	3	Srpx2
**6.64876478908564e-06**	-0.29235021307	0.0918260905020618	3	Nupr1
**6.66367461703389e-06**	-1.48	0.0920320101358551	3	Rhou
**6.82512943025852e-06**	-1.06	0.0942618625613004	3	Serf1
**6.82545618159824e-06**	0.440864774185	0.0942663753240533	3	Foxp1
**7.13510197952281e-06**	-0.65313612774	0.0985428934391895	3	Rnf181
**7.30036468945477e-06**	-1.10	0.10	3	Sgpp1
**7.38492139734136e-06**	-0.71172215955	0.101993149418682	3	Wbp4
**7.60588956526771e-06**	-0.50549366211	0.105044940785912	3	Socs3
**7.6975652572598e-06**	-1.26	0.106311073768015	3	Emilin2
**7.79544054075909e-06**	1.26	0.107662829308424	3	Hyal2
**7.85798658133605e-06**	-1.06	0.108526652674832	3	Cdk5rap3
**7.98057003692316e-06**	-1.29	0.110219652779946	3	Galnt16
**7.98738837060662e-06**	-0.36794606701	0.110313820786448	3	Ndufb3
**8.16390165328951e-06**	-0.42225586451	0.112751645733581	3	Ube2b
**8.27351738297514e-06**	-0.39443130295	0.11	3	Fkbp9
**8.44408037344167e-06**	-0.93	0.116621194037603	3	Tgfbrap1
**8.53888962518581e-06**	0.702812014076	0.117930604613441	3	Bmpr2
**8.80123075592766e-06**	-0.45310891156	0.121553797970117	3	Dnajc1
**8.85985980115231e-06**	1.14	0.122363523713715	3	Zfpm1
**8.97758803926138e-06**	-1.15	0.123989468410239	3	Gnai1
**9.25375995563533e-06**	-1.51	0.13	3	Chil1
**9.26257310285997e-06**	1.49	0.127925397123599	3	Sncaip
**9.32482220874023e-06**	-0.30965200509	0.128785119524911	3	Siae
**9.53489793357167e-06**	-0.46	0.131686475360558	3	Vimp
**9.56904080961555e-06**	-0.79488604109	0.13	3	Dclk1
**9.60457829905697e-06**	-0.42558670387	0.132648830888276	3	Oaf
**1.00432292320422e-05**	-0.51512466282	0.138707038923735	3	Dph3

**1.01407618655772e-05**	-0.96	0.140054062125486	3	Sema3d
**1.01423352835448e-05**	-1.00	0.140075792601037	3	Ccl2
**1.05474116801636e-05**	-0.33122933040	0.145670302714739	3	Calm2
**1.0573854030958e-05**	-0.33	0.146035498021561	3	Slc25a5
**1.06575173928519e-05**	-1.07	0.147190972712678	3	Pmm1
**1.06880841504723e-05**	-0.38	0.147613130202174	3	Evc2
**1.07692065646373e-05**	-0.54977832848	0.148733511864206	3	Golph3
**1.08273413449204e-05**	-1.56	0.149536411314696	3	Fst
**1.08386794961904e-05**	-0.83060180920	0.149693002521886	3	Arsb
**1.09885003721229e-05**	-1.27	0.151762178639389	3	Tbx20
**1.10928680527972e-05**	0.33482398056	0.153203600677182	3	Ifitm2
**1.11343597164169e-05**	-0.81668519907	0.153776642043434	3	Gmppa
**1.11627570135616e-05**	-1.14	0.154168837114299	3	Trak1
**1.11929634405077e-05**	-1.82	0.154586018076852	3	Ptx3
**1.13168873304989e-05**	-0.33700188900	0.156297530921521	3	Rtn4
**1.16448818285775e-05**	-0.56856085777	0.160827462934484	3	Ptov1
**1.17432202932289e-05**	-0.38100462653	0.162185615469785	3	Ifrd1
**1.18834185808856e-05**	1.43	0.164121894020611	3	Sema6a
**1.20910457564151e-05**	-0.46777535069	0.166989432941849	3	Ndufa1
**1.23797368434055e-05**	-0.34953278049	0.170976545544273	3	Rpn1
**1.24614763331679e-05**	-0.85054973211	0.172105449637381	3	Slc5a3
**1.27274767101414e-05**	-0.90631315827	0.175779180843763	3	Enpp2
**1.27481234979577e-05**	-0.59249102154	0.176064333630293	3	Sntb2
**1.28421240091704e-05**	-0.90090617397	0.177362574690652	3	Ptgir
**1.31243203087152e-05**	1.12	0.181259987783666	3	Adipor2
**1.33074184096365e-05**	-0.57640043619	0.18	3	Fkbp3
**1.33872493737835e-05**	1.75	0.184891301101324	3	Nhsl2
**1.3397347131153e-05**	-0.77049198774	0.185030761228354	3	Angpt1
**1.37746988671281e-05**	-0.53232833870	0.190242366053906	3	Tram1
**1.38082633217687e-05**	-0.29654452801	0.190705924736948	3	Man2a1
**1.39184805889523e-05**	-0.65408267850	0.19	3	Mdk
**1.39481368352614e-05**	-0.76153849703	0.192637717831796	3	Idh1
**1.42693186554804e-05**	-0.37493097896	0.20	3	Tgfbr1
**1.43817752922836e-05**	-1.67	0.198626698561729	3	Maob
**1.44651475785675e-05**	-0.37526822350	0.199778153207596	3	Stx2
**1.4628447078661e-05**	1.23	0.202033482603387	3	Pdia5
**1.47046320791515e-05**	-0.34330724609	0.203085673645162	3	Bscl2
**1.51788946441958e-05**	-0.54391326183	0.209635713930988	3	Vdac1
**1.54036226392468e-05**	-0.50051369474	0.212739432270637	3	Commd3
**1.56357952744509e-05**	-0.63	0.215945968535442	3	Pdcd6
**1.60389768525784e-05**	-1.31	0.221514309310961	3	Kcnma1
**1.61931937082321e-05**	-1.33	0.223644198304393	3	Mme
**1.62485166571532e-05**	-0.52370363320	0.224408263551943	3	Gpsm1
**1.63416136493536e-05**	-0.47	0.225694026111223	3	Ipo4
**1.70018860564711e-05**	-0.47411129905	0.234813048325922	3	Myo9a
**1.72670466532003e-05**	-1.06	0.24	3	Rftn1
**1.72818563611404e-05**	1.67	0.238679718203711	3	Atp10d
**1.73818073245997e-05**	-1.33	0.240060140960046	3	Tmeff2
**1.76312556213236e-05**	-1.27	0.243505271386101	3	Chrdl1
**1.76903282128399e-05**	-0.34725648070	0.244321122947532	3	Cct5
**1.77066918042845e-05**	0.61616395141	0.244547120508973	3	Asap2
**1.80082865788222e-05**	-1.29	0.248712445940114	3	LOC1026365
**1.80812769282419e-05**	-0.80758413331	0.249720515655948	3	Ptprf
**1.82489968141439e-05**	-1.34	0.252036895000142	3	Fam132a
**1.83136502191708e-05**	-0.80017761451	0.252929823176968	3	Naaa
**1.87858926266955e-05**	0.93812814362	0.259451963067291	3	Mllt3

**1.91880630674101e-05**	-0.84161244058	0.265006339024001	3	Pja1
**1.93096187523316e-05**	-0.77047996200	0.266685144588451	3	Dpy19l1
**1.93549056233967e-05**	-0.99067339882	0.267310601564732	3	Pcbp4
**1.96520238622258e-05**	0.99670200407	0.271414101561201	3	Nxn
**1.96904272205945e-05**	-1.16	0.271944490343631	3	Sobp
**1.97318838042183e-05**	-0.65756245583	0.272517047220059	3	Tnfaip3
**2.00368174525277e-05**	-0.97929038043	0.28	3	Col27a1
**2.03300173381321e-05**	-1.44	0.280777869456943	3	S1pr3
**2.04323213884072e-05**	0.35623700301	0.282190790695292	3	Epha7
**2.06021952353719e-05**	-1.44	0.284536918395721	3	Cilp
**2.14004027871189e-05**	-1.28	0.295560962892899	3	Ifi205
**2.16210709746167e-05**	-0.81117125241	0.298608611230431	3	Plekhm2
**2.17147048239447e-05**	-1.18	0.299901788323501	3	Bdnf
**2.17628996582804e-05**	-1.36	0.300567407180511	3	Ildr2
**2.25701634718568e-05**	-0.59658532761	0.311716527709814	3	Creb3
**2.27913443024007e-05**	-0.44053567184	0.314771256160455	3	Vps35
**2.28758788401997e-05**	-0.90739809747	0.315938762661998	3	Flrt2
**2.30500298163869e-05**	-0.74266030194	0.32	3	Cd109
**2.39067602231676e-05**	-0.48650582392	0.330176265442168	3	Trappc2l
**2.39610352382803e-05**	-0.43613498287	0.330925857675889	3	Kmt2c
**2.44438367105116e-05**	-0.85956055500	0.337593828808876	3	Pqlc3
**2.44812036494014e-05**	-0.26368716120	0.338109903601883	3	Cacybp
**2.45162011419385e-05**	0.87081067323	0.338593253971312	3	Apbb2
**2.45266333796132e-05**	-0.35706579812	0.338737333605838	3	Slc25a4
**2.48699380346942e-05**	-0.36278602563	0.343478714197161	3	Marc2
**2.48842364435644e-05**	0.71	0.343676189522067	3	Dmap1
**2.49147072568335e-05**	-1.55	0.344097021924128	3	Gng8
**2.50437471980512e-05**	-0.86	0.345879192552285	3	Baiap2
**2.51746068373855e-05**	-1.32	0.347686495031132	3	Foxs1
**2.5406188226001e-05**	-0.27458837990	0.350884865589299	3	Mrpl20
**2.55302942953232e-05**	-1.02	0.352598894512708	3	Isg20
**2.59224203695739e-05**	-1.27	0.358014547724186	3	Cys1
**2.60554661126933e-05**	-0.39488781351	0.359852042482407	3	Acaa2
**2.63445451119289e-05**	0.42865830594	0.36	3	Rpl18a
**2.65148579619363e-05**	-1.22	0.366196703312303	3	Akr1b8
**2.66097351766521e-05**	-1.20	0.367507052524743	3	Pcdh18
**2.67368812193143e-05**	-0.26202842116	0.37	3	Atf6
**2.67595137633678e-05**	-0.51065442800	0.369575644585873	3	Sec61a1
**2.68145067756482e-05**	-0.38577944122	0.370335153078477	3	Jak1
**2.69000692818971e-05**	-0.79034900512	0.371516856852281	3	Enc1
**2.69571367393383e-05**	-0.70	0.372305015507001	3	Gnptg
**2.71622844253543e-05**	-1.19	0.375138310198569	3	Lsamp
**2.71664431343345e-05**	-0.27305746392	0.375195746128294	3	Hcfc1r1
**2.73235247346532e-05**	-1.71	0.377365200110295	3	Slc10a6
**2.75826684701341e-05**	-0.36616525704	0.380944234241022	3	Lrp6
**2.84790671938985e-05**	1.01	0.393324397014932	3	Prpf40b
**2.97659535552462e-05**	0.78622278873	0.411097584551505	3	Id1
**2.97739482567226e-05**	0.58684679798	0.411207999373596	3	Tulp4
**3.07902335177803e-05**	-1.22	0.425243915114063	3	1500009L16
**3.10405020102736e-05**	-0.31965574117	0.428700373263889	3	Tmem167
**3.13262941738982e-05**	0.58088128558	0.432647448835708	3	Sulf1
**3.13450943110395e-05**	-0.36421227597	0.432907097529767	3	Map3k2
**3.13568919535464e-05**	-0.56415942299	0.433070034770429	3	Cnn2
**3.14169286842288e-05**	-0.62262395151	0.433899202057884	3	Ptprs
**3.17779081943832e-05**	-0.40066738164	0.438884690072627	3	Romo1
**3.20726194826883e-05**	-0.78	0.442954947675408	3	Uchl4

**3.2350003276364e-05**	-0.38251304239	0.446785895249863	3	Surf4
**3.26083409170586e-05**	-0.36003759154	0.450353796405496	3	Ifnar2
**3.39093836783208e-05**	-0.80571734602	0.468322497981288	3	Habp4
**3.39297598349919e-05**	-0.28540161024	0.468603913081073	3	Rbbp7
**3.41051972153553e-05**	-0.55161293197	0.471026878741273	3	Anxa6
**3.41998434601678e-05**	-0.59332245164	0.472334038028378	3	Psmc5
**3.45331224437974e-05**	-0.62484962577	0.476936954071285	3	Cct3
**3.48724656490288e-05**	-1.35	0.481623623078737	3	Gfra1
**3.51739778288785e-05**	-0.51317248500	0.485787807794641	3	Csrnp1
**3.62826824239239e-05**	-0.89831563751	0.501100126956813	3	Elmo2
**3.76786556776706e-05**	-0.32523767633	0.520379913564308	3	Ccdc23
**3.7695303963315e-05**	-1.37	0.520609843037344	3	Aldh7a1
**3.78645269889147e-05**	0.551149978038	0.522946982243901	3	Gata6
**3.82069703209984e-05**	-1.16	0.53	3	Cpeb1
**3.85289544429443e-05**	-0.59186259724	0.532123389811504	3	Man1a2
**3.8750170402405e-05**	-0.46	0.535178603427616	3	Csnk1a1
**4.01013159825748e-05**	-1.03	0.55	3	Acot13
**4.04809983662505e-05**	-0.42408325562	0.559083068436286	3	Prdx4
**4.20937151098432e-05**	-1.04	0.581356299382044	3	Pde1a
**4.25497122270028e-05**	-1.14	0.587654075567135	3	Omd
**4.27182880325182e-05**	-1.11	0.589982276017109	3	Fam19a5
**4.34638808612375e-05**	-0.30182117434	0.60	3	Nucb1
**4.37073631611204e-05**	-1.01	0.603642392618233	3	Prkca
**4.37262058872033e-05**	-0.75521312713	0.603902629508165	3	Fam3a
**4.47789791528256e-05**	0.504623993257	0.618442481079674	3	Msn
**4.52561694822145e-05**	-0.75753804593	0.625032956718865	3	Fam89b
**4.53121524795591e-05**	-0.52597361542	0.63	3	Fkbp4
**4.57259876129103e-05**	-1.00	0.631521614921904	3	Hspb2
**4.58523839931645e-05**	-1.01	0.633267275329595	3	Cspg4
**4.61654148309476e-05**	-0.48375614730	0.637590544230218	3	Ikbip
**4.63609680576735e-05**	-0.54249281147	0.640291329844528	3	1110001J03R
**4.72101588437741e-05**	0.437169620552	0.652019503791364	3	Tgm2
**4.78903268120497e-05**	-0.29033307776	0.661413303601218	3	Egln1
**4.86901379754327e-05**	-0.46106166332	0.672459495578701	3	Vapa
**4.99507447690403e-05**	-1.26	0.689869736005216	3	Pi4k2a
**5.18689020349684e-05**	-0.37101360646	0.716361406004948	3	Tgfbr3
**5.1889398323175e-05**	-1.16	0.72	3	Sil1
**5.29503229195877e-05**	-0.34338980777	0.731296909842425	3	Ttc3
**5.35528089003282e-05**	-0.80	0.739617843722433	3	Ang
**5.39079013430409e-05**	-0.32214286273	0.744522025448738	3	Flii
**5.43105515879612e-05**	-0.66126827535	0.750083027981332	3	C1qtnf6
**5.71288090247514e-05**	-0.30989805333	0.789005981440841	3	Tspan4
**5.73879121014789e-05**	0.609219290805	0.792584454033525	3	Bnip2
**5.83652132338185e-05**	-0.46020839388	0.806081959972268	3	Slc39a14
**5.99365126837778e-05**	-0.97294114102	0.827783176675656	3	Abcb1b
**6.01191129999736e-05**	-0.57712071645	0.830305069642635	3	Dbndd2
**6.06758864912958e-05**	-0.74863019267	0.837994668331286	3	Ssbp2
**6.07299394578641e-05**	-0.47430121454	0.838741193852561	3	2010012O05R
**6.11749337339909e-05**	-1.28	0.844887009800148	3	Gria4
**6.1213574324123e-05**	1.15	0.845420674990463	3	AW112010
**6.16922837124249e-05**	-1.01	0.85	3	Efs
**6.17655478442443e-05**	-0.74046597719	0.853043981276858	3	Papss1
**6.24563372202355e-05**	0.749617486003	0.862584473348673	3	Hspa1a
**6.35531242875687e-05**	-0.43698357266	0.877732199535611	3	Etfb
**6.43289573989481e-05**	0.662427139390	0.888447230636872	3	Entpd1
**6.55315310404762e-05**	-0.53242769813	0.905055975200017	3	Fam98a

**6.57612106160071e-05**	0.57108260740‹	0.908228079817674	3	Fbln2
**6.60054584172896e-05**	-1.70	0.911601386201186	3	Ccl11
**6.73808877725455e-05**	-0.40607754525	0.930597441026625	3	Cndp2
**6.83982491492744e-05**	-0.41622568650	0.944648219000628	3	Atp5d
**6.88136924599158e-05**	0.29376913807‹	0.950385906563897	3	Cd200
**6.89327734069976e-05**	-1.58	0.952030533524043	3	Cxcl14
**7.05230512241967e-05**	-0.28100842794	0.973993860457381	3	H2afj
**7.0540220109473e-05**	-0.25337250097	0.974230979931932	3	Clptm1l
**7.10747348452525e-05**	-1.31	0.981613162947783	3	Epyc
**7.12354617236176e-05**	-0.26404817032	0.983832961864883	3	Kcnb1
**7.16801768254705e-05**	-0.86873993032	0.989974922136574	3	Wdr35
**7.22251628043E-05**	-0.43297153437	0.997501723490188	3	Mrpl32
**7.2235723029686e-05**	-0.93079055945	0.997647570762993	3	Abcc5
**7.34539983674304e-05**	0.48834342909‹	1.00	3	Ywhae
**7.75013481981504e-05**	-0.99265738613	1.00	3	Mustn1
**7.84125706763952e-05**	-0.39280964086	1.00	3	Rabgap1l
**7.88869655948284e-05**	-0.63011118509	1.00	3	Plin3
**7.93967197353435e-05**	-0.80095193343	1.00	3	Glo1
**8.12898280545716e-05**	-0.76909267399	1.00	3	Nnmt
**8.27173095773605e-05**	-1.16	1.00	3	Efhd1
**8.31832258658585e-05**	-0.74745318481	1.00	3	Hlf
**8.39814891887279e-05**	-0.96458738340	1.00	3	Adcy5
**8.49056539280197e-05**	-0.91522591845	1.00	3	Nsg1
**8.49321643197069e-05**	-0.78196890454	1.00	3	Kazald1
**8.54155305653044e-05**	-1.19	1.00	3	Fibin
**8.64722926364867e-05**	-0.39802899260	1.00	3	Acadsb
**8.68018360114678e-05**	-1.28	1.00	3	Irs1
**8.69450866148709e-05**	-1.34	1.00	3	Loxl3
**8.81602527373812e-05**	-0.89929681790	1.00	3	Pfn2
**8.8335252528821e-05**	-0.42605488980	1.00	3	Gm15421
**8.90818169385804e-05**	0.92560608239‹	1.00	3	Slc7a1
**9.00807904271419e-05**	-0.54472322953	1.00	3	Lztr1
**9.09069906704832e-05**	-0.38038921390	1.00	3	Nop58
**9.15740560584366e-05**	-0.65224836871	1.00	3	Csad
**9.31097534377137e-05**	-0.48060826230	1.00	3	Npepps
**9.33170652706694e-05**	-0.29215454410	1.00	3	Comt
**9.39799817760772e-05**	0.38911476575‹	1.00	3	Cyr61
**9.39882658430945e-05**	-0.48923645549	1.00	3	Flot1
**9.4328413316334e-05**	-1.13	1.00	3	Pgls
**9.43474549547231e-05**	-0.29664565806	1.00	3	Trib1
**9.44450644275513e-05**	-0.41374931677	1.00	3	N6amt1
**9.47666657820172e-05**	-0.27999831665	1.00	3	Gstp1
**9.49169708287225e-05**	-1.34	1.00	3	Hp
**9.51790077618521e-05**	-1.10	1.00	3	Ldlr
**9.52258824839984e-05**	-0.56794838789	1.00	3	Chd3
**9.67147524696143e-05**	-0.31363017359	1.00	3	Ndufb2
**9.73991489667701e-05**	-0.27780738566	1.00	3	Tead1
**9.78334419116052e-05**	-0.25203947715	1.00	3	Ufc1
**9.91978865092351e-05**	-0.77981187701	1.00	3	Npy1r
**9.97927215066926e-05**	-1.45	1.00	3	Olfml1
**9.98397038798397e-05**	-0.45010275900	1.00	3	Ptp4a1
**0.000101264414853515**	-0.63150347280	1.00	3	Bcl3
**0.000102087726398434**	0.81026933999‹	1.00	3	Adipor1
**0.000102361226695667**	-1.21	1.00	3	Ptpdc1
**0.000105983597122001**	-0.63925650686	1.00	3	Gda
**0.000107129934326185**	-0.28	1.00	3	Cbx6

**0.000107916750554147**	-0.89325942828	1.00	3	Uchl3
**0.000108144019423403**	-0.52243198966	1.00	3	P4ha1
**0.000110754686184377**	-1.03	1.00	3	Twist2
**0.000110771926885659**	-0.30848954096	1.00	3	2010107E04F
**0.000110852266326798**	-0.68	1.00	3	Phf2
**0.00011351460344542**	0.250258440366	1.00	3	Rps11
**0.000113946580282679**	-0.61949960080	1.00	3	Nol12
**0.000114396687360623**	-0.66	1.00	3	Crtc3
**0.000115001068933699**	-0.67670076709	1.00	3	Fam20c
**0.000115353447193341**	-1.20	1.00	3	AI607873
**0.000117129224808573**	0.815241116869	1.00	3	Med11
**0.000117154138459462**	-0.98183133026	1.00	3	Tdrd7
**0.000119990152622797**	-0.87471865993	1.00	3	Agtrap
**0.000122336053076994**	-0.73559099911	1.00	3	Gm3258
**0.000123293834324758**	-0.82167934993	1.00	3	Wdsub1
**0.00012453105926624**	-0.54826902864	1.00	3	Smim4
**0.000125510009868981**	-1.44	1.00	3	Enpp3
**0.000128934616083601**	-0.98050001093	1.00	3	Antxr2
**0.000129269086191874**	-0.79890241124	1.00	3	Khdrbs3
**0.000131364036494241**	-0.37762117811	1.00	3	Tgfb2
**0.000131962025938082**	-1.09	1.00	3	Lrig1
**0.000132035322196926**	-0.36735034917	1.00	3	Ppp1cb
**0.000133134602242985**	1.01	1.00	3	Gata3
**0.000133224633168051**	1.46	1.00	3	Ralgapa2
**0.000134771678718692**	-0.54	1.00	3	Rcor3
**0.000135646985171906**	-0.54408603271	1.00	3	Olfr1033
**0.000137691036665992**	-1.05	1.00	3	Gcsh
**0.000139345900585385**	-0.85753112362	1.00	3	Ift74
**0.000139833166744801**	-0.91780106208	1.00	3	Lepre1
**0.000145004334170132**	-0.53	1.00	3	Cercam
**0.00014684915078421**	-0.27399984044	1.00	3	Sgce
**0.000150221981581898**	-0.73072057724	1.00	3	Trps1
**0.000150925827880243**	0.819476850949	1.00	3	Arhgap29
**0.00015196638935406**	-0.64077931527	1.00	3	Tmem150a
**0.000152493366200778**	-0.92309904326	1.00	3	Fbxw8
**0.000152642677247619**	-0.29	1.00	3	Sash1
**0.000155246618518708**	-0.68215432912	1.00	3	Nolc1
**0.000159797019875609**	-0.34170413364	1.00	3	Gnb4
**0.000161333677340402**	1.20	1.00	3	Ephb4
**0.000161450989863318**	-0.39	1.00	3	Ces2g
**0.000163460636593621**	1.07	1.00	3	Pqlc1
**0.00016370483492899**	-0.28560714733	1.00	3	Zcrb1
**0.000164091423857712**	0.592707623911	1.00	3	Tmod3
**0.000167456652099999**	-1.37	1.00	3	Bdh2
**0.000167992281157448**	-1.06	1.00	3	Htatip2
**0.000170030961801612**	-1.41	1.00	3	1110065P20F
**0.000172380947698086**	-1.07	1.00	3	Fam13c
**0.000173388457554073**	-0.39230791941	1.00	3	Creld2
**0.000173953347602968**	-0.69292669094	1.00	3	Aldh18a1
**0.000174753856264719**	-0.79327457909	1.00	3	Syvn1
**0.000176808116133712**	-0.37633464659	1.00	3	Tmem147
**0.000177154229869093**	-0.55874886113	1.00	3	2310022B05F
**0.000178611937077481**	-0.25481357517	1.00	3	Cops5
**0.00018055193269885**	-1.79	1.00	3	Angptl7
**0.000183308067435314**	-0.94497923259	1.00	3	Thrb
**0.00018442118639755**	-0.55287113291	1.00	3	Usp2

**0.000184786969656817**	-0.77026082260	1.00	3	Cmtm3
**0.000185038878717033**	-0.33887493051	1.00	3	Sh3pxd2a
**0.000185862183588577**	-0.34065733278	1.00	3	Osbpl8
**0.000186577302293482**	-0.76995668214	1.00	3	Pdxk
**0.000188079442371972**	-0.45865334286	1.00	3	Mtdh
**0.000192465435716332**	1.16	1.00	3	Enpp4
**0.000192979262472619**	0.26	1.00	3	Pam
**0.000197872150480262**	-0.38843708355	1.00	3	Manf
**0.000198115113754598**	-0.83249774051	1.00	3	Gpr39
**0.000198572044420887**	-0.70329716482	1.00	3	Sgcd
**0.000199660341617292**	-0.25086770272	1.00	3	Ran
**0.000200132981687333**	-0.28226662937	1.00	3	Nup153
**0.000204899255710568**	-0.77619341088	1.00	3	Prkcd
**0.000205179459928778**	-0.68814121696	1.00	3	Mef2c
**0.000205719918851135**	-1.26	1.00	3	Vldlr
**0.000214111183412405**	0.26457903369[illegible]	1.00	3	Cfh
**0.000214154821464862**	0.81527238543[illegible]	1.00	3	Tmem141
**0.000217131357455898**	0.66399319670[illegible]	1.00	3	Ptk2b
**0.000218486479085665**	-0.54627374530	1.00	3	Myg1
**0.0002185786799333**	1.20	1.00	3	Ggta1
**0.000219006142462014**	-0.40805829036	1.00	3	Zeb1
**0.000219313227985689**	-0.89152969316	1.00	3	Gstt3
**0.000220217321676429**	0.79031143933[illegible]	1.00	3	Bcap29
**0.000222599627219837**	-0.40	1.00	3	Phldb1
**0.000222830646010652**	-1.19	1.00	3	Ednra
**0.000224088399256623**	0.65991545537[illegible]	1.00	3	Itga3
**0.000225374322124448**	-0.30079741304	1.00	3	Arl4a
**0.000230826838807714**	-0.34251376895	1.00	3	Pbx1
**0.000231702580185506**	-0.28	1.00	3	Epdr1
**0.000234439541273937**	-0.47447672935	1.00	3	Tprkb
**0.000237285139473398**	-0.27584257982	1.00	3	Atp5a1
**0.000237889009276499**	-0.25973849289	1.00	3	Tomm22
**0.000238368200515626**	-0.61350156330	1.00	3	Mta3
**0.000241013132816707**	-0.73584806330	1.00	3	Smim8
**0.000242445611581231**	-0.95123239607	1.00	3	Mzt2
**0.000243055817714742**	-0.64434540035	1.00	3	Flt3l
**0.000243083884268888**	-0.40527006909	1.00	3	Sema3b
**0.000244792536400334**	-0.36204957038	1.00	3	Lgals3
**0.000246464191922711**	-0.83364952340	1.00	3	Apip
**0.00024651739589993**	-0.69523260499	1.00	3	Gypc
**0.000246746321012433**	-0.85054973211	1.00	3	Rasa3
**0.000247032211937627**	-0.60262280315	1.00	3	Clcc1
**0.000248176284559771**	-0.82014203217	1.00	3	Acbd6
**0.000248531766770662**	-0.49177180220	1.00	3	Pim1
**0.000249025951794345**	-0.94456913458	1.00	3	Map3k5
**0.00024916829193779**	-0.63746321485	1.00	3	Dnajc25
**0.000249405939956841**	-0.97267568014	1.00	3	Traf3
**0.000250051050530335**	-1.45	1.00	3	Gfra2
**0.000254024600949833**	-0.39751805087	1.00	3	Filip1l
**0.000254501792219362**	-0.81	1.00	3	Kiz
**0.000254515173192478**	-1.19	1.00	3	Mark1
**0.000258365993040161**	1.76	1.00	3	Bpgm
**0.000260724116952748**	-0.28250421635	1.00	3	Psmd1
**0.000262457215018094**	-0.33460604996	1.00	3	Klf9
**0.000265219646644779**	-0.42209386516	1.00	3	Il1r1
**0.000265836134916458**	-0.60456418745	1.00	3	Gusb

**0.000269359846602015**	-0.66144539767	1.00	3	Dcbld2
**0.000270791451714891**	-0.60527542960	1.00	3	Txnrd1
**0.000271378448747009**	-1.03	1.00	3	Tmem38b
**0.000279116195870911**	1.01	1.00	3	Tmpo
**0.000282460298414056**	-1.33	1.00	3	Trim41
**0.000282500927145288**	-0.31884610253	1.00	3	Rbms1
**0.00028510492845261**	-0.46628040547	1.00	3	Qsox1
**0.000285814616989472**	-0.56236729862	1.00	3	Acadvl
**0.000286383715760998**	-0.28047799562	1.00	3	Rrbp1
**0.000286511739489425**	-0.80841350227	1.00	3	Rab7l1
**0.000288376477092334**	0.962546171896	1.00	3	Gng11
**0.00028860181846532**	-0.32560163253	1.00	3	Cdo1
**0.000289438731008767**	-0.33106137969	1.00	3	Pkn2
**0.000290484433377521**	-0.56579851281	1.00	3	Sssca1
**0.000291540877372676**	0.92	1.00	3	Klf7
**0.000292017282936179**	1.15	1.00	3	Fam43a
**0.000294803770366668**	-0.99767594860	1.00	3	Gcdh
**0.000295034097674053**	-0.36894069888	1.00	3	Asap3
**0.000298423321538412**	-0.52348302765	1.00	3	9030624J02F
**0.000298917022026648**	-0.80523965679	1.00	3	Sh3pxd2b
**0.000306205582274119**	-0.37968519489	1.00	3	Lonrf3
**0.000311943007858143**	-0.33670890376	1.00	3	Mtmr6
**0.000313662159433929**	1.11	1.00	3	Fam135a
**0.000318843591371173**	-1.43	1.00	3	Ntrk2
**0.000324548606187228**	-1.56	1.00	3	AI507597
**0.000324591503982162**	-0.27224514619	1.00	3	Aga
**0.000329312426337434**	-0.46875341541	1.00	3	Smtn
**0.000331286531237785**	0.879732511197	1.00	3	Anp32a
**0.000332172449042625**	-0.85	1.00	3	S1pr2
**0.00033284919982895**	-0.27308889423	1.00	3	Jmjd1c
**0.000336769576937106**	-0.31357624878	1.00	3	Pkia
**0.000339425692317713**	-0.39686852809	1.00	3	Snap47
**0.000355268895993099**	-0.36992387778	1.00	3	Csf1
**0.000358121019163694**	-0.71091461711	1.00	3	Setd7
**0.000359570251561384**	0.894994087921	1.00	3	Dusp6
**0.00036696684123518**	-0.45324353227	1.00	3	Nqo2
**0.000367438699578619**	-0.74834184124	1.00	3	Prdm5
**0.000369882143870445**	-0.80559599416	1.00	3	Aptx
**0.000376915942315192**	-0.86942403570	1.00	3	Casc4
**0.000376993124913819**	-0.35549873203	1.00	3	Tmem204
**0.00037723284026512**	0.576384708295	1.00	3	Ndrg1
**0.000379963622738705**	-0.66363501630	1.00	3	Postn
**0.000381120589337017**	-1.26	1.00	3	Angptl1
**0.000381589700687448**	1.39	1.00	3	Map3k1
**0.000382825445413038**	-0.72559560757	1.00	3	Psmd10
**0.000393888040150009**	-0.51993975173	1.00	3	Tnfrsf11b
**0.000394768527103943**	0.433769138035	1.00	3	Hes1
**0.000395482291861004**	-1.01	1.00	3	Dlg5
**0.000397054532803605**	1.19	1.00	3	Ppm1f
**0.000402561489121556**	-0.66791585670	1.00	3	Nbea
**0.00040476836218978**	-0.69389085621	1.00	3	Snta1
**0.000406053628211015**	-0.42411874041	1.00	3	Zfp131
**0.000409225476804105**	-0.27258753527	1.00	3	Eif1a
**0.000409501888543523**	-0.36269852319	1.00	3	Poglut1
**0.000415312385281527**	-0.61774939849	1.00	3	Mpp5
**0.000421424417338807**	-0.59921608363	1.00	3	Itm2a

**0.000432388831042263**	-0.83	1.00	3	Coq10b
**0.000433654224460479**	-0.64925645188	1.00	3	2310011J03R
**0.000434420727257684**	-1.01	1.00	3	Mfsd11
**0.000443118116422631**	-0.70	1.00	3	Siva1
**0.000446609759222372**	-0.30	1.00	3	Gaa
**0.000450182061799103**	-0.56241060830	1.00	3	Ndufaf2
**0.000453855588049841**	0.714295115364	1.00	3	Txnrd3
**0.000455193589481452**	-0.62411841885	1.00	3	Herpud2
**0.000456192818633925**	-0.88989337413	1.00	3	Farp1
**0.000460331258181023**	-0.39550219402	1.00	3	Dlgap4
**0.000463315411693763**	-0.62922029232	1.00	3	Slain2
**0.000465081973699608**	-0.57186721533	1.00	3	Abat
**0.000466165247760366**	-0.33743565016	1.00	3	Ptgis
**0.000485978449687659**	-0.87484482418	1.00	3	Rgs10
**0.000495750584781785**	-1.37	1.00	3	Lhfpl2
**0.000505728303909111**	-0.43702334001	1.00	3	Pik3r1
**0.000507831078011606**	-0.87360158746	1.00	3	Rftn2
**0.000507977765277323**	-0.60598633625	1.00	3	Mfsd5
**0.000510718920436506**	-1.06	1.00	3	Kif1a
**0.000510771041260888**	-0.60130954828	1.00	3	Yif1a
**0.000513523524954359**	-0.59586890274	1.00	3	Ndn
**0.000514662722678558**	-0.53283529309	1.00	3	Carhsp1
**0.000522143839533704**	-0.52304386109	1.00	3	Cisd2
**0.000529745380823759**	-0.83239992738	1.00	3	A4galt
**0.000533850949854699**	-0.49282569347	1.00	3	Nasp
**0.000545845113816364**	-0.65251539081	1.00	3	Chmp4b
**0.00054624198714085**	0.419619537187	1.00	3	Rac1
**0.000546680579762002**	-0.47411129905	1.00	3	Rasa2
**0.000550399177948951**	-0.65820179471	1.00	3	Dock7
**0.000556067164548957**	-0.62647018482	1.00	3	Cnpy4
**0.00055758957683726**	-0.87636331837	1.00	3	Sap18
**0.000560219240709333**	-1.08	1.00	3	Shisa4
**0.000560302289709924**	-0.94411492830	1.00	3	Echdc2
**0.000566122383263672**	-0.30644879647	1.00	3	Eif2s1
**0.000573313097823268**	0.718862045646	1.00	3	Tnfaip1
**0.000575136590071527**	-0.82966751822	1.00	3	Mrps6
**0.000577522182784081**	-0.62482200301	1.00	3	Bambi
**0.000581962982484387**	-0.26483060365	1.00	3	Mpv17
**0.000581963645804021**	-0.51642299706	1.00	3	Rhbdf1
**0.000582995154793644**	-0.25007971780	1.00	3	Mgst1
**0.000583252453417713**	0.31	1.00	3	Epn2
**0.000588182934218596**	-0.28110092415	1.00	3	Kdelr2
**0.000591682065248314**	0.630051793588	1.00	3	Tmbim6
**0.000592213412642463**	-0.37	1.00	3	Crtap
**0.000613931724257454**	-0.30028870234	1.00	3	Atp1b1
**0.00061480240759144**	-0.79	1.00	3	Sgsm2
**0.000616487984833184**	-0.46299124801	1.00	3	Med13
**0.000619358569281439**	-0.31596766031	1.00	3	Arih1
**0.000624633432225517**	-0.54300592424	1.00	3	Prdm6
**0.000627582911831764**	-0.44529238461	1.00	3	Sh3bgrl
**0.000631371229855395**	-0.33837611269	1.00	3	Nagk
**0.000633039439320864**	-0.75781722312	1.00	3	Dram1
**0.000640045169060923**	-0.39085788478	1.00	3	Scara3
**0.000643158066453273**	-0.28256787956	1.00	3	Gadd45b
**0.000650387226051222**	-0.63447844422	1.00	3	Pcbd2
**0.000650557560093078**	-0.61555532761	1.00	3	Plin4

**0.000654940650180469**	-0.38248974441	1.00	3	Myd88
**0.000655290556704734**	-0.86686144675	1.00	3	Rfc1
**0.000663672970129994**	-0.43035092709	1.00	3	Rad23a
**0.000664774223710494**	-0.45447532454	1.00	3	Jkamp
**0.000668552820895961**	-0.31701944063	1.00	3	Anpep
**0.000679595872887808**	-0.68931009508	1.00	3	Vgll3
**0.000683291549255472**	-0.30140470744	1.00	3	Clta
**0.000686587171086877**	-0.30422022204	1.00	3	Clcn4-2
**0.000687299817057898**	-0.45621476688	1.00	3	Nov
**0.000690917351760283**	-0.81303301638	1.00	3	Prrg3
**0.000694293460348937**	-1.33	1.00	3	Gm13889
**0.000695546591554468**	-0.79366548242	1.00	3	Rpp30
**0.000696199683782991**	-0.71297279325	1.00	3	Zbtb38
**0.000696986022176848**	-0.39475422733	1.00	3	Yars
**0.000699196459704128**	-0.25367324904	1.00	3	Gabarap
**0.000720639449524591**	-0.42386876985	1.00	3	Sdhc
**0.000721231171540782**	1.02	1.00	3	Wdfy3
**0.000729401379995893**	-0.54239439693	1.00	3	Tep1
**0.000730940129489623**	-0.35873081623	1.00	3	Gng12
**0.000734638868830579**	-0.51477001494	1.00	3	Fmnl2
**0.000738199400946344**	0.285568261542	1.00	3	Rps26
**0.000742145092879816**	-0.70986062276	1.00	3	Mast2
**0.000745530862216945**	-0.63561848095	1.00	3	Smim3
**0.000747268026357087**	-0.38093087104	1.00	3	Coq2
**0.000753570415479698**	-0.90881322735	1.00	3	Shisa3
**0.000755548881449712**	0.297316800333	1.00	3	Pkp4
**0.000755863930833909**	0.359052672656	1.00	3	Tpm4
**0.000768493280781639**	-0.58865643929	1.00	3	D19Bwg1357
**0.000770997869383654**	-0.63641653051	1.00	3	Dhrs1
**0.000772223652963836**	-0.69637495590	1.00	3	Mtfr1l
**0.000780958918196405**	-0.86015269170	1.00	3	Ebpl
**0.000801496545274846**	-0.76515095720	1.00	3	Gli3
**0.000804781540709915**	-0.29815665593	1.00	3	Rabgef1
**0.000807586482662935**	-0.63153762255	1.00	3	Ifngr2
**0.000814011269408273**	-0.62658384457	1.00	3	Ptpn9
**0.000822829731849931**	-0.76562374554	1.00	3	Il13ra1
**0.000830467052185923**	-0.26478456639	1.00	3	Ppl
**0.000841776075574279**	-0.50098019107	1.00	3	Csgalnact2
**0.000847157525786308**	-0.29393797080	1.00	3	Mrps17
**0.000847372042831328**	-0.26264860735	1.00	3	Slc11a2
**0.000858610632076355**	-0.69004428840	1.00	3	Cyp27a1
**0.00087361523266413**	-0.60856003877	1.00	3	Mpnd
**0.000876987115914291**	-0.35	1.00	3	Ggnbp2
**0.000880312042200467**	-1.04	1.00	3	B3gnt9
**0.00088037644655837**	-0.27869229575	1.00	3	Mbd1
**0.000883062525085895**	-0.33	1.00	3	Ppp2ca
**0.000883274991542524**	-0.58334029149	1.00	3	Bok
**0.000883965918877237**	-0.79673142287	1.00	3	Lnp
**0.000888572744523896**	-1.11	1.00	3	Slc16a9
**0.00089943267739252**	-0.40959991091	1.00	3	Samd4b
**0.000899500460952892**	-0.95712516503	1.00	3	Acyp2
**0.000899965368129373**	-0.89694226158	1.00	3	Zfp651
**0.000900514438847192**	-0.30803362440	1.00	3	Psma2
**0.000901989703187688**	1.16	1.00	3	Map3k11
**0.00090938818996968**	-0.38805457607	1.00	3	Rnf19b
**0.000914117136248781**	-0.29321120661	1.00	3	Lynx1

**0.000921261263601807**	-0.90436497459	1.00	3	2700029M09
**0.000921593794194384**	-0.41444942518	1.00	3	Cisd1
**0.000925762135271432**	-0.42036004353	1.00	3	Sec63
**0.000932269660019741**	-0.44245001715	1.00	3	Man2b1
**0.000943175753647894**	-0.46535512670	1.00	3	Yipf5
**0.000950435473384166**	-0.33518112031	1.00	3	Kpna4
**0.000950795077661242**	-0.28457149881	1.00	3	Rap1gds1
**0.000954484701723573**	0.25589704438	1.00	3	Rpl32
**0.000961263102502636**	1.28	1.00	3	Ppp1r13b
**0.000964674601220943**	-0.46610135307	1.00	3	Peak1
**0.000965899992847592**	0.57484539916	1.00	3	Pdgfd
**0.000970982262962411**	-0.27436010712	1.00	3	Txn2
**0.00097199454899293**	-1.52	1.00	3	Hhip
**0.000972885190931564**	-0.45178182265	1.00	3	Eef2k
**0.000973473574397640**	-0.36960743348	1.00	3	Auts2
**0.000974041279131489**	0.59	1.00	3	Hs6st1
**0.000974512166576442**	-0.61466606283	1.00	3	Tuba1c
**0.000978380575859640**	-0.52	1.00	3	Dnajc19
**0.000980640172199126**	-0.52330462253	1.00	3	Tspan31
**0.000981044971827142**	-0.94411492830	1.00	3	Id4
**0.000981384119750667**	-0.30974854306	1.00	3	Glt25d1
**0.000993165926508592**	-0.25016948234	1.00	3	Cst3
**0.000995139483404225**	0.78084706000	1.00	3	Gnaq
**0.00100287793101873**	-0.47733840956	1.00	3	Tmem64
**0.00100631201932037**	-0.69332557223	1.00	3	Cnst
**0.00100778263326194**	-0.25688155586	1.00	3	Cuta
**0.00101540147487504**	-0.78877645881	1.00	3	Lcn2
**0.00101793372783415**	-0.36901830102	1.00	3	Bbx
**0.00102261772580103**	-0.33226695224	1.00	3	Selt
**0.00103790403733545**	-0.52645409881	1.00	3	Pls3
**0.00104952954406835**	-0.40283562457	1.00	3	Tmem127
**0.00106270126584993**	1.28	1.00	3	Calml4
**0.00106289035970836**	-0.37	1.00	3	Trp53inp2
**0.0010640871002258**	-0.53341295985	1.00	3	Stk39
**0.00107602760374878**	-0.30227337676	1.00	3	Serpine1
**0.00108438485069941**	-0.60784592306	1.00	3	Sec22b
**0.00110141314661059**	-0.52960591557	1.00	3	Ppp1r15b
**0.00110620628955804**	0.61613273993	1.00	3	Taok2
**0.00110768255546455**	-0.44496314373	1.00	3	Nhp2
**0.00111254126457631**	-0.56512794546	1.00	3	Tmem126a
**0.0011147718614547**	-0.41448418355	1.00	3	Psme4
**0.00112014357467863**	-0.28424060526	1.00	3	Fam20b
**0.00112548428374365**	-0.49526309599	1.00	3	Pcsk5
**0.00113679200585241**	-0.67	1.00	3	B3gat3
**0.00113695093063368**	-0.70164669504	1.00	3	Hddc2
**0.0011390808054965**	-0.95599092712	1.00	3	Ramp1
**0.00117651334881377**	-0.76148807460	1.00	3	Sord
**0.00117816992448866**	-0.30309912770	1.00	3	Fam210a
**0.001180809193179**	-0.96803294751	1.00	3	Tlr2
**0.00118331866616386**	-0.30602683214	1.00	3	Sep15
**0.00119019425928507**	-0.34050997880	1.00	3	Prdx3
**0.00119025827699474**	-0.47	1.00	3	Pdha1
**0.0012038526917731**	-0.52235971754	1.00	3	Nop56
**0.00120796965586883**	-0.40963399428	1.00	3	Atp6v1b2
**0.00121363559067328**	-0.73633543881	1.00	3	Plgrkt
**0.00121488857318649**	0.28222127631	1.00	3	2810428I15R

**0.00121563438960386**	-0.71289636402	1.00	3	Crot
**0.00122056354645586**	0.613490969473	1.00	3	Sec14l1
**0.00124358274582558**	-0.54197994333	1.00	3	Kat2b
**0.00124371021135053**	-0.30447490179	1.00	3	Fem1b
**0.00124571979042799**	0.858959859670	1.00	3	Lysmd2
**0.00124647965158971**	-0.54304868280	1.00	3	Ehmt2
**0.00125294297265777**	0.458084152061	1.00	3	Utrn
**0.00127730276801854**	-0.48227044682	1.00	3	Praf2
**0.00129348194641263**	-0.95040245973	1.00	3	Tmem29
**0.00130030898055229**	-0.78261469932	1.00	3	1810026J23R
**0.00130460099139612**	-0.78199607882	1.00	3	Cyp2j6
**0.00134106733488877**	-0.58794644170	1.00	3	Derl2
**0.00135100274615396**	-0.47663336849	1.00	3	Scfd1
**0.00135160630470714**	-0.32395902519	1.00	3	Med15
**0.00135397256656656**	0.582886593536	1.00	3	Sh3bgrl3
**0.00135770461696842**	0.885716488873	1.00	3	Brms1
**0.00136049915074973**	-0.31897370562	1.00	3	Glg1
**0.0013753884632893**	-0.82915427117	1.00	3	Aoc3
**0.00138744194815124**	-0.38363070783	1.00	3	Myo5a
**0.00141251060694952**	-0.60797730771	1.00	3	Bccip
**0.00141500601072693**	0.733358809828	1.00	3	Hspa12b
**0.00141645147948131**	-0.41348667724	1.00	3	Ssbp1
**0.00142536756538941**	-0.49039218466	1.00	3	Mrpl27
**0.00144949466275616**	-0.31361391010	1.00	3	Srgap3
**0.00148906507711248**	-0.79	1.00	3	Slc35b2
**0.00150096549189886**	0.274863450383	1.00	3	Tmem176b
**0.00151894721854623**	-0.36340332549	1.00	3	Elof1
**0.00153202294433379**	0.807169226941	1.00	3	Crlf2
**0.00153572985314884**	-0.26608994569	1.00	3	Park7
**0.00154225863457397**	-1.02	1.00	3	Metrnl
**0.00155741862273403**	-0.37136614076	1.00	3	Nfkb2
**0.00156088209066566**	-0.37756409071	1.00	3	Fdx1
**0.00157049041052159**	0.519326192218	1.00	3	Sat1
**0.00157854840681048**	-0.41082487864	1.00	3	Snx7
**0.0015951128332027**	-0.60601693941	1.00	3	Gga2
**0.00159702894744642**	-0.63383302393	1.00	3	Dap3
**0.00160431298796024**	-0.49382802609	1.00	3	Heatr5b
**0.00160840808457787**	-0.42596892917	1.00	3	Setbp1
**0.00161965977324061**	-0.45231328295	1.00	3	Rxra
**0.00163248028543741**	-0.27323150168	1.00	3	Kdm6b
**0.00163255186541053**	-0.92563718054	1.00	3	Cluap1
**0.00163953824960246**	0.289993077256	1.00	3	App
**0.0016683765378209**	-1.28	1.00	3	Dync2li1
**0.00167336771245788**	-0.27483921982	1.00	3	Yipf3
**0.00168657331201707**	-0.36830976905	1.00	3	Mocs2
**0.00168996784565893**	-0.49753843195	1.00	3	Mid1ip1
**0.00169700985099109**	-0.27	1.00	3	Cd55
**0.00169819320626577**	0.896479221754	1.00	3	Thbs1
**0.00169971707349984**	-0.36586502346	1.00	3	Socs2
**0.00170206707818162**	-0.38802903111	1.00	3	Plekhj1
**0.00172248966266348**	0.569272231662	1.00	3	Eif4ebp3
**0.00172454396329295**	-0.32933681485	1.00	3	Mrpl54
**0.00175707772719459**	0.875155133945	1.00	3	Skap2
**0.00177015219463027**	-1.31	1.00	3	Mtus2
**0.00177125699008684**	-0.42153348297	1.00	3	Mif
**0.00177989353451598**	0.358534284542	1.00	3	Pdlim1

**0.0017822675913726**	0.483994637431	1.00	3	Nck2
**0.00178314388902579**	-0.41	1.00	3	Wdr60
**0.00178518436949291**	-0.55783472991	1.00	3	0610037L13R
**0.00179049116375669**	-0.78682596772	1.00	3	Map6
**0.00179596914065251**	-0.45443853345	1.00	3	Ibtk
**0.00180875108298597**	-0.43960460364	1.00	3	Dopey1
**0.00181828027824399**	-0.26433631776	1.00	3	Calu
**0.00182180368307384**	-0.43999854458	1.00	3	Stox2
**0.00183937375290784**	0.474184180293	1.00	3	Cabin1
**0.00184539925007033**	-0.73806530081	1.00	3	Max
**0.00186149617625256**	-0.30405191591	1.00	3	Parp3
**0.00186208768189421**	-0.27	1.00	3	Sepn1
**0.00187971331390307**	0.866833321575	1.00	3	Thsd7a
**0.00188207918213815**	-0.76439415320	1.00	3	Tmem175
**0.00189655443097431**	-0.41613555783	1.00	3	Nf1
**0.00189762446611344**	-0.30125336757	1.00	3	Rer1
**0.0018981792100518**	-0.26360548280	1.00	3	Timm17a
**0.001909281804873**	-0.67254402267	1.00	3	Pkdcc
**0.00193663118449626**	0.77	1.00	3	Casd1
**0.00193856113849688**	-0.27406027828	1.00	3	Golt1b
**0.00195973346041223**	-0.99023686295	1.00	3	Cacna1c
**0.00197357380146186**	-0.69639315277	1.00	3	Aimp2
**0.00199309964348561**	-0.70522302002	1.00	3	Osbpl1a
**0.00199977987602229**	-0.37611844909	1.00	3	Ctps2
**0.0020059644425803**	-0.32540635306	1.00	3	Asun
**0.00201866392012959**	-0.29039988797	1.00	3	Dtx3
**0.00204139846553015**	0.901989495642	1.00	3	Etv6
**0.00204592493596914**	-0.94365314117	1.00	3	Laptm4b
**0.0020555673150074**	-0.68442472380	1.00	3	Nicn1
**0.00208389744899523**	-0.29968290030	1.00	3	Hdgfrp3
**0.00208650068370574**	0.458543797196	1.00	3	Fam206a
**0.00209610721176805**	-0.30702170933	1.00	3	Sema5a
**0.00210014337141992**	-1.34	1.00	3	Zfp354c
**0.00210316196163502**	-0.81858672717	1.00	3	Cpox
**0.00213179524571749**	-0.80092811967	1.00	3	Wdr6
**0.00213478094205171**	-0.46955251551	1.00	3	Htra2
**0.00214712381498179**	-0.27068643695	1.00	3	Ank
**0.00215026342026809**	-0.46513112325	1.00	3	Zc2hc1a
**0.00217883160290092**	-0.25802029971	1.00	3	1700123O20R
**0.00218050187642981**	-0.47376509860	1.00	3	Fzd7
**0.00219561514912652**	-0.37485875039	1.00	3	Pygb
**0.00220045143873818**	-0.69093480635	1.00	3	Mrps18a
**0.00221248637904629**	-0.39969247828	1.00	3	Surf1
**0.00221932528004633**	-0.82800460003	1.00	3	Micu2
**0.00224833545684047**	-0.26275779252	1.00	3	Hmgn1
**0.0022564463060518**	-0.53	1.00	3	2310039H08R
**0.00226274803697878**	1.42	1.00	3	Stxbp6
**0.00227406156630501**	-0.31481576949	1.00	3	Snx12
**0.00228819532752283**	-0.31830995207	1.00	3	Alkbh5
**0.00229227946833081**	-0.40999197725	1.00	3	Ctr9
**0.00231763901900122**	-0.95	1.00	3	Slc20a2
**0.00232499978446008**	-0.61674320951	1.00	3	Ten1
**0.00232728026970143**	-0.42695616114	1.00	3	Rufy2
**0.00232793045882375**	-0.39796459021	1.00	3	Gab1
**0.0023293833515267**	-0.88249022745	1.00	3	Arsk
**0.00238029309948155**	0.868473176052	1.00	3	Rfk

**0.00240102857476239**	-0.48920781170	1.00	3	Ccdc88a
**0.00242521269211062**	-0.25218800414	1.00	3	Slc37a3
**0.00243347532576429**	-0.85569467516	1.00	3	Socs5
**0.00246352196200978**	0.314865406942	1.00	3	AI987944
**0.00248631633355719**	-0.42893777535	1.00	3	Tmem87b
**0.00250583776334637**	0.29	1.00	3	Tbl1xr1
**0.00251966763496181**	-0.42392680849	1.00	3	Ssh2
**0.0025240335128053**	-0.51153502105	1.00	3	Tkt
**0.00253775067486852**	-0.59999154494	1.00	3	Fxr2
**0.00254495118780471**	-0.88279184401	1.00	3	Tbxa2r
**0.00254743527107465**	-0.26588635131	1.00	3	Ncbp2
**0.00256110809252465**	-0.60010686099	1.00	3	Gsk3a
**0.00256474483654304**	-0.38227240331	1.00	3	Pgd
**0.00260432042233165**	-0.25221075598	1.00	3	Snx22
**0.00260905856290536**	-0.66252003475	1.00	3	Slc7a6
**0.00262245244433732**	0.379459199507	1.00	3	Wipi1
**0.00262683179912637**	-0.27184773243	1.00	3	Cc2d2a
**0.00264812946947764**	-0.37189828777	1.00	3	Sars
**0.00266839639326078**	-0.68069334178	1.00	3	Arhgef40
**0.00269662389316344**	-0.36784470546	1.00	3	Kdm6a
**0.00273961069205804**	-0.65056773639	1.00	3	AW549877
**0.00273991443094693**	-0.44935510854	1.00	3	Ndufs4
**0.00274754301766542**	-0.52466357822	1.00	3	Fech
**0.00275855445887591**	-0.61739104957	1.00	3	Naga
**0.00277115197022695**	0.93	1.00	3	Efna1
**0.00279463665478305**	-0.30662173121	1.00	3	Ube2j1
**0.00279530750533368**	-0.54578179133	1.00	3	Plekhg2
**0.00280774470819812**	-0.60	1.00	3	Dbn1
**0.00280801967630645**	-0.66095901294	1.00	3	Emd
**0.00281036058902446**	-0.41461370286	1.00	3	Chp1
**0.00282468225352464**	-0.77699259124	1.00	3	Gucy1a2
**0.00282801839557971**	0.608998400149	1.00	3	Icam1
**0.00284207379355833**	-0.27249464479	1.00	3	Man1c1
**0.00284498841161435**	-0.33129127773	1.00	3	Enah
**0.00285186213532203**	-0.94655997829	1.00	3	Spsb1
**0.00285280537677752**	-0.58889232263	1.00	3	Stim1
**0.00289148544649203**	0.711956646503	1.00	3	Jam2
**0.00289659389935028**	-0.39784206009	1.00	3	Srxn1
**0.00290292212451361**	-0.61492288989	1.00	3	Shmt2
**0.00290504219004399**	-1.04	1.00	3	Sorl1
**0.00290562462538464**	-0.26808037797	1.00	3	Cyb5
**0.00293754434743901**	-0.50292032825	1.00	3	Impad1
**0.00296684620339551**	0.782505238753	1.00	3	Gltp
**0.00296699349827942**	-0.50	1.00	3	Slc25a12
**0.00297397130287594**	0.786375737426	1.00	3	2810025M15
**0.00297483168067547**	-0.59356557779	1.00	3	Tbc1d10b
**0.00303722746812241**	0.893852725864	1.00	3	Ets1
**0.00305272228289632**	-1.15	1.00	3	Rasl12
**0.00305327912600436**	-0.57828627812	1.00	3	Tbc1d9b
**0.00307964026504814**	0.271147618167	1.00	3	Dusp1
**0.00308751172742172**	-0.29025246294	1.00	3	Pygm
**0.00310616010102369**	-1.02	1.00	3	Hspb6
**0.00312237546234813**	-1.00	1.00	3	Irs2
**0.00312494981668615**	-0.96332831790	1.00	3	Rab3il1
**0.00313370845108941**	-0.77465846730	1.00	3	Spry1
**0.00315128123280747**	-0.28939644235	1.00	3	Zfp36l2

**0.00315285106988343**	-0.29395793267	1.00	3	Gas2l1
**0.00316798229885768**	-0.82954560725	1.00	3	Itih4
**0.00318025473001353**	0.429542084720	1.00	3	G6pc3
**0.00318565972159885**	-0.51	1.00	3	Igbp1
**0.0032042690525212**	0.609487302770	1.00	3	Shroom3
**0.00321555984613436**	-0.39446113309	1.00	3	Vps16
**0.00324446813278456**	-0.28652000326	1.00	3	Gba
**0.00330782388054305**	-0.32248778762	1.00	3	Plod3
**0.00331243933740023**	-0.82002613353	1.00	3	Thsd4
**0.00331369990871283**	-0.76589555887	1.00	3	Gclc
**0.00332404233075077**	-0.95753794863	1.00	3	Ephb3
**0.00335561040037398**	0.336273214552	1.00	3	Fads1
**0.00337557930087685**	-0.68958663573	1.00	3	Ergic1
**0.00337691791336174**	-0.38087168906	1.00	3	Rdm1
**0.00337972690915974**	0.252463999266	1.00	3	2700081O15I
**0.00339246918337478**	-0.69217373723	1.00	3	Map2k7
**0.00340060269722365**	-0.68	1.00	3	Spred1
**0.0034081281140528**	0.396685827598	1.00	3	Ctnna1
**0.00343102140073554**	-0.59531207922	1.00	3	Ddt
**0.00344958198767529**	0.901359144001	1.00	3	Mrps5
**0.0035178110526229**	-0.38755756703	1.00	3	Upf3a
**0.00354097720861243**	-0.58873114012	1.00	3	Ppp1r7
**0.00354699725944066**	-0.54360490188	1.00	3	Med8
**0.00359975745546764**	-1.09	1.00	3	Agpat2
**0.00360609055077663**	0.799041712836	1.00	3	Ktn1
**0.00363651267442947**	-0.38528154138	1.00	3	Cdk13
**0.00366500358127916**	-0.40239961380	1.00	3	Plxnb2
**0.00367568183283931**	-0.27984472018	1.00	3	Cdc37
**0.00369288653659376**	-0.51399388533	1.00	3	Map2k1
**0.00370521166958985**	-1.20	1.00	3	Prss12
**0.00370825396962697**	-0.68367781802	1.00	3	Lrig3
**0.00371526342573501**	-0.51765210734	1.00	3	Dpp9
**0.00377107773668519**	0.267653498004	1.00	3	Abhd14a
**0.00379344663056786**	0.788740153343	1.00	3	Ptplad2
**0.00385675807882308**	-0.74493698118	1.00	3	Slc25a46
**0.00388794154342415**	-0.42387332806	1.00	3	Pla2g15
**0.00391101920549177**	-0.61823333247	1.00	3	Osbpl5
**0.00392993013043176**	-0.52093674214	1.00	3	Fnbp1
**0.00396015611355115**	-0.46	1.00	3	Bet1
**0.00400707534250519**	-0.50623957604	1.00	3	Psd3
**0.00401596914504085**	0.523067547723	1.00	3	Usp10
**0.00401807189021075**	-0.25185559638	1.00	3	Exosc4
**0.00402548518091798**	0.720267894702	1.00	3	Smap2
**0.00405762303866701**	-0.41986070930	1.00	3	Tmem248
**0.00405976693668643**	-0.67045185871	1.00	3	Ift43
**0.00410013620841573**	-0.53847904058	1.00	3	Dcun1d3
**0.00410355243408252**	-0.75629129435	1.00	3	Ormdl3
**0.00415095385555902**	-0.72901076366	1.00	3	Zfc3h1
**0.00417434935294892**	0.782571705817	1.00	3	Med4
**0.00418891834349084**	-0.44298038046	1.00	3	2510009E07F
**0.00422142242321369**	-0.44708262666	1.00	3	Fam53c
**0.00427307028897805**	-0.55979304681	1.00	3	Aup1
**0.00427344386840574**	-0.53235031864	1.00	3	Hibadh
**0.00432773290851492**	-0.31026289655	1.00	3	Ddx50
**0.00433144891560049**	-0.51093206752	1.00	3	Rcbtb2
**0.00433699361197971**	-0.26482692650	1.00	3	Hand2

**0.00434215932217948**	-0.49	1.00	3	Xpnpep1
**0.00440885341229981**	-0.42933018192	1.00	3	Dmpk
**0.00445512312135551**	-0.93119264681	1.00	3	Npr3
**0.0044745412433838**	-0.32044499802	1.00	3	Ncoa6
**0.00447771545300215**	-0.78	1.00	3	Ehbp1
**0.00449757254394315**	-0.67872677281	1.00	3	P2rx4
**0.00453329320286347**	0.742922635672	1.00	3	Ptbp3
**0.00464127156712237**	-0.40521680919	1.00	3	Trafd1
**0.00465373988740681**	0.664915331910	1.00	3	Fam63b
**0.00468028058612932**	-0.50460146581	1.00	3	Txndc16
**0.00468080552587223**	-0.67440934685	1.00	3	Prkaa1
**0.00478508893319896**	-0.28654507253	1.00	3	G6pdx
**0.00479092406128508**	-0.31506290819	1.00	3	Rab21
**0.00483891145968873**	0.855696396083	1.00	3	Pla2g16
**0.00488924260689043**	0.583814291728	1.00	3	Nmt2
**0.00489068474399441**	-0.49	1.00	3	Cc2d1b
**0.00491766359266673**	0.87	1.00	3	Nsf
**0.00495611928739978**	-0.31	1.00	3	Dctn6
**0.00499840509807491**	0.795484903892	1.00	3	Sorbs2
**0.00501036409480304**	0.656356155851	1.00	3	Jup
**0.00502617608391887**	-0.28092007002	1.00	3	Atf1
**0.00504242560293486**	-0.39846807054	1.00	3	Lrp3
**0.00505904200223622**	-0.87777298016	1.00	3	Exoc4
**0.0050692167923437**	-0.47543003853	1.00	3	Mpzl1
**0.00507243674691977**	-0.58	1.00	3	Tspan17
**0.00510097317232555**	-0.84524905177	1.00	3	Sphk1
**0.00511148570140386**	-0.51537763928	1.00	3	Fat4
**0.00511651354818999**	0.702844461530	1.00	3	Adss
**0.00515091507281254**	-0.55804960963	1.00	3	Ube2k
**0.00515187420228374**	-0.37209766675	1.00	3	Palm
**0.00517461974521792**	0.25	1.00	3	Uso1
**0.00519024395898526**	-0.57073813474	1.00	3	Hsd17b4
**0.00521718669117006**	0.983820964937	1.00	3	Epb4.1
**0.00524617790877024**	-0.30089330800	1.00	3	Ints6
**0.00526148343871076**	-0.53446355263	1.00	3	Idh3a
**0.00527163588957434**	0.762748927154	1.00	3	Tmx1
**0.0052820395326848**	-0.39970990828	1.00	3	1810055G02I
**0.00529301399465154**	-0.40947707040	1.00	3	Chmp1a
**0.00529435596624848**	-0.36025754154	1.00	3	Emc2
**0.00532867824726224**	-0.50191358036	1.00	3	Pde4a
**0.00535123817144312**	-0.54432526510	1.00	3	Kif3a
**0.00543208189506258**	-0.93251753948	1.00	3	Rnf150
**0.00543231619156001**	0.999528965532	1.00	3	Tmtc2
**0.00543270174015697**	0.619806992907	1.00	3	Dazap2
**0.005491898461596**	-0.35	1.00	3	Tm9sf4
**0.00549808189606293**	-1.27	1.00	3	Wif1
**0.00552647315168334**	0.35	1.00	3	Iqgap1
**0.005526499713266**	-0.30208712606	1.00	3	Cops4
**0.00554697049096923**	-0.50385091565	1.00	3	Slmo2
**0.00563304896103397**	-0.32045377668	1.00	3	Ndufs2
**0.00563549131963463**	-0.40	1.00	3	Edem1
**0.00568249527448463**	-0.60314481670	1.00	3	Twf1
**0.00576268327447923**	-0.96511816636	1.00	3	Zfp592
**0.005773718331149**	-0.39704715982	1.00	3	Tshz2
**0.00579864233586419**	-1.12	1.00	3	Epha3
**0.0058121701852729**	-0.58939545790	1.00	3	Rab2b

**0.00581541147752247**	-0.26056343480	1.00	3	Copz1
**0.00589530351664405**	-1.31	1.00	3	Saa3
**0.005908390341263**	-0.60	1.00	3	Dut
**0.00590853673270929**	-1.06	1.00	3	Rgs5
**0.00591611906671101**	-0.81076389459	1.00	3	Fbxw5
**0.00593506405074152**	1.01	1.00	3	Orai1
**0.00595440282310368**	-0.48403693885	1.00	3	Egr2
**0.00598071746983834**	-0.45626593873	1.00	3	Grik5
**0.0060194784707988**	-0.89479853142	1.00	3	Ifi30
**0.00602062907020204**	-0.35008627507	1.00	3	Galnt2
**0.00604912474027451**	-0.49080617762	1.00	3	Jdp2
**0.00607295296254372**	-0.36081166198	1.00	3	Tmem263
**0.00607403031392602**	-0.57744770877	1.00	3	Kctd17
**0.00611098017383417**	-0.70497541217	1.00	3	Lmod1
**0.00611372908601453**	-0.27586880223	1.00	3	Decr1
**0.0061169480486605**	0.89407214152	1.00	3	Lbr
**0.00611899583069082**	-0.27726164170	1.00	3	Araf
**0.00612007536599813**	-0.34424253733	1.00	3	Mon2
**0.00613881272028516**	-0.74261231265	1.00	3	Akr7a5
**0.00615423292242871**	-0.44351347785	1.00	3	Rabep1
**0.00616588867126574**	-0.28	1.00	3	Mrpl43
**0.00618709560227835**	0.78096086344	1.00	3	Slc20a1
**0.00621743899557592**	-0.33838582955	1.00	3	Pla2g4a
**0.00622915629063593**	0.78476749851	1.00	3	Hint2
**0.00638888558840638**	-0.28790780177	1.00	3	Ltbr
**0.00642565620914807**	-0.35455782918	1.00	3	Dmd
**0.00649967486219277**	0.97076223067	1.00	3	Zfp326
**0.00650633305375266**	-1.06	1.00	3	Hmgcs1
**0.00652317409922106**	-0.53740319630	1.00	3	Cdc37l1
**0.00655275918597461**	-0.51407665765	1.00	3	Rprd1b
**0.00659804085323965**	-0.59596826353	1.00	3	Srprb
**0.00662087417434213**	0.62	1.00	3	Plxna4
**0.00665167167594995**	-0.42629874763	1.00	3	Naa60
**0.00665820764102757**	-0.62137015284	1.00	3	Ubp1
**0.00667227750643149**	-0.25244077323	1.00	3	Exoc3
**0.00667690825335899**	-0.31103042164	1.00	3	Lonp2
**0.0066928799870494**	-0.57149304898	1.00	3	Tmem214
**0.00669560815829849**	-0.26724863334	1.00	3	Rusc1
**0.00672527275982414**	-0.55145656766	1.00	3	Pawr
**0.00674444891610755**	-0.59916854346	1.00	3	Pcdh7
**0.00676452861787549**	-0.62283602959	1.00	3	Mrps11
**0.00677475986739856**	-0.57791418200	1.00	3	Fgf1
**0.00679561414552065**	-0.65575215372	1.00	3	Dtymk
**0.00681180946734067**	-0.80944910560	1.00	3	Mrps12
**0.00681996990387683**	-0.61334287812	1.00	3	Klhl21
**0.00682521289653532**	1.27	1.00	3	Hscb
**0.00693987831377881**	-0.40802615218	1.00	3	Meis1
**0.00693993219982462**	-0.42260855190	1.00	3	Rp9
**0.00695306987500089**	-0.77624665076	1.00	3	Itih2
**0.00696258288596939**	-0.99065822980	1.00	3	Clybl
**0.00700205910626062**	-0.47502537590	1.00	3	D3Ertd254e
**0.00701727541351556**	-0.31317423644	1.00	3	Tm2d2
**0.00705722734971685**	-0.40	1.00	3	Mif4gd
**0.00712114567434629**	-0.36464916346	1.00	3	Ppm1b
**0.00712853094552243**	-0.40752811488	1.00	3	Tcerg1
**0.00719336391543332**	1.36	1.00	3	Hrct1

**0.00725285227789368**	-0.42833671767	1.00	3	Rab22a
**0.00728253527350085**	-0.59439487492	1.00	3	Poldip2
**0.00736535893451423**	-0.36	1.00	3	Slc4a3
**0.00737407457569077**	0.69828237782[illegible]	1.00	3	Tanc1
**0.0074076721251052**	-0.71739965903	1.00	3	Oard1
**0.00742770921209149**	-1.07	1.00	3	Fbxl22
**0.00748986214029701**	-0.48	1.00	3	Golga5
**0.0075183123946231**	-1.13	1.00	3	Itga7
**0.00754806338155722**	-0.90191756710	1.00	3	Aff2
**0.00755691557218516**	-0.65311797465	1.00	3	Slc22a17
**0.00755853730198156**	-1.47	1.00	3	Kcnmb1
**0.0075609404383702**	-0.58625625996	1.00	3	Acp2
**0.00757406166984329**	-0.33781474567	1.00	3	Rabgap1
**0.00767086477383854**	-0.36113761653	1.00	3	Fam193a
**0.00768081818439171**	-0.46392568899	1.00	3	Kcnj15
**0.00771325359625916**	-0.67407151491	1.00	3	Rnf123
**0.00774771371454046**	-0.98057181067	1.00	3	Tpm2
**0.0077660247956383**	-0.40405842579	1.00	3	Fkbp14
**0.0078106506500731**	-0.37686875150	1.00	3	Dnal4
**0.00786306453450128**	-0.40200492378	1.00	3	Slc9a1
**0.00787591253346691**	-0.25677234320	1.00	3	Pld3
**0.00791896472907802**	-0.60029314252	1.00	3	Ifi35
**0.00792381671108719**	-0.26818758081	1.00	3	Akr1e1
**0.00794699787200844**	-0.41550930488	1.00	3	Senp7
**0.00821469736060115**	-0.25685109712	1.00	3	Mfsd10
**0.00825468889717129**	-0.32518296425	1.00	3	Lrrk1
**0.00827908055408836**	-0.47	1.00	3	Cep120
**0.00828358327851842**	-0.66352587018	1.00	3	Rbl2
**0.00833291917443489**	0.25772091079[illegible]	1.00	3	Ncl
**0.00836451149983724**	-0.47631845745	1.00	3	Nrbp2
**0.00837064634016622**	-0.84504721386	1.00	3	Phf10
**0.00838320169491874**	-0.73567248343	1.00	3	Bace1
**0.00843555698045484**	-0.96	1.00	3	Dock10
**0.00849155036021851**	0.29255597745[illegible]	1.00	3	Eln
**0.0084919710641678**	-0.32941326613	1.00	3	Ociad1
**0.00853522540390955**	-0.26594851107	1.00	3	Optn
**0.00854924087040052**	-0.28801920184	1.00	3	Aamdc
**0.00863978366788804**	-0.38939993688	1.00	3	Gnl3
**0.0086798379085127**	-0.36136605743	1.00	3	Abl1
**0.00870477473227593**	-0.61086171142	1.00	3	Inhba
**0.00874863361650804**	-0.88948336356	1.00	3	Ercc6l2
**0.00884612624425909**	-0.27	1.00	3	Pgm2
**0.00889641139035595**	-0.45327020859	1.00	3	Ube2a
**0.00889912666528128**	0.82984826110[illegible]	1.00	3	Phactr2
**0.00902015250739256**	0.70	1.00	3	Atl2
**0.0090470506926111**	-0.46047710778	1.00	3	Cic
**0.00904957801360491**	-0.70369333801	1.00	3	Fbxo18
**0.00906628898469686**	-0.86451064532	1.00	3	Gpr64
**0.00907660251376535**	0.44531695853[illegible]	1.00	3	Sh3glb1
**0.00909463360468502**	-0.35140733958	1.00	3	Rere
**0.00909689329210117**	0.61	1.00	3	Txnip
**0.00919413831404261**	0.62450098961[illegible]	1.00	3	Fzd4
**0.00924236074553584**	0.56	1.00	3	Rab11fip2
**0.00924411919731549**	-1.23	1.00	3	Lmbr1
**0.00925619873805289**	0.64215520666[illegible]	1.00	3	Emp2
**0.00926621058709022**	0.69265960075[illegible]	1.00	3	Phc2

**0.00926837779307036**	-0.55735400946	1.00	3	Hnrnpul1
**0.00931652256531837**	-0.32474903267	1.00	3	Rnaseh2c
**0.00933477242288187**	-0.92930522683	1.00	3	Gpc1
**0.00936167802178327**	-0.81046766753	1.00	3	Extl3
**0.00937509199442569**	-0.72295353819	1.00	3	Fam129a
**0.0094447374895224**	-0.88479399178	1.00	3	Cd2bp2
**0.00950426275435446**	-0.30503496901	1.00	3	Ppp1r14a
**0.00957614234151687**	-0.25742537556	1.00	3	Cbx5
**0.00958184356703945**	0.31668045173	1.00	3	Npat
**0.00958611549687317**	-0.38	1.00	3	Tubb2a
**0.00960387349975286**	-0.40714498684	1.00	3	Zfp827
**0.0097004224923722**	-0.57899551082	1.00	3	Zkscan17
**0.00971340048198849**	-0.55692657639	1.00	3	Hyi
**0.009734019262288**	-0.27616287891	1.00	3	Gorasp2
**0.00975135480739576**	-0.49896442301	1.00	3	Pinx1
**0.00980133269265549**	-0.31921482918	1.00	3	Ap4s1
**0.00983638205116853**	-0.28738885498	1.00	3	Snx1
**0.00992776956646623**	-0.28142171412	1.00	3	Smarcd3

p_val	avg_logFC	p_val_adj	cluster	gene
1.27419059030556e-36	1.62	1.75978462427102e-32	4	Tpm2
1.75005994649201e-35	1.51	2.41700779210011e-31	4	Tagln
1.20822469967782e-33	1.67	1.66867913272504e-29	4	Cnn1
3.06275547432466e-33	1.15	4.22997158558979e-29	4	Myh11
2.97607141833422e-32	1.39	4.11025223586139e-28	4	Myl9
3.11915574417685e-32	1.49	4.30786599828265e-28	4	Itga8
3.90165398122307e-31	1.32	5.38857431346718e-27	4	Mylk
9.64940447481215e-31	1.26	1.33267925201631e-26	4	Acta2
1.853973053977E-30	1.47	2.56052218484764e-26	4	Cbr2
1.47521616365946e-29	1.95	2.03742104363008e-25	4	Tes
5.88764491743851e-28	1.83	8.13142639547432e-24	4	Myom1
1.63904752213999e-27	1.56	2.26368853282754e-23	4	Sh3bgr
1.67862236971609e-27	0.97	2.3183453548149e-23	4	Flna
4.76608130324368e-27	-0.70	6.58243488790984e-23	4	Itm2b
4.77135313044731e-27	1.56	6.58971580846078e-23	4	Npnt
1.50869456088167e-26	-0.920852451580314	2.08365805803368e-22	4	Ifitm3
1.62235585211598e-26	1.25	2.24063566735737e-22	4	Susd5
1.66342116989613e-26	-0.73	2.29735097774354e-22	4	Ftl1
1.84074397300665e-26	-1.74	2.54225150111948e-22	4	Igfbp4
1.08774444625323e-25	1.49	1.50228385472033e-21	4	Lmod1
1.18373875799138e-25	1.97	1.63486159866189e-21	4	Ptprz1
2.63145035609033e-25	-2.08	3.63429608679635e-21	4	Serpinf1
3.21064926500784e-25	1.32	4.43422769990232e-21	4	Nov
1.13291679634242e-24	1.49	1.56467138742851e-20	4	Synpo2
1.21429726820114e-24	1.66	1.67706595711259e-20	4	Sost
1.32106389609167e-24	-1.99	1.82452134689221e-20	4	Pcolce
2.59252527687966e-24	1.38	3.5805366598985e-20	4	Rasl12
2.73502341323598e-24	1.19	3.77734083602022e-20	4	Ncam1
1.34130287108954e-23	-0.800095049584556	1.85247339526176e-19	4	Cfh
1.37662104684587e-23	1.55	1.90125132779884e-19	4	Thsd4
1.39429592526364e-23	-2.08	1.92566210238161e-19	4	Dpt
1.5215580540014e-23	1.21	2.10142382838134e-19	4	Col18a1
2.82254265821407e-23	1.24	3.89821366525946e-19	4	Smtn
5.52167929428906e-23	1.76	7.62599127334262e-19	4	Speg
7.49106868161634e-23	-1.37	1.03459149561803e-18	4	Serping1
8.302838634511E-23	-1.56	1.14670504381231e-18	4	Mmp2
2.10165616656446e-22	-1.53	2.90259733164218e-18	4	Rnase4
2.53725132090401e-22	1.25	3.50419779930052e-18	4	Cspg4
2.74846604270162e-22	1.17	3.79590645157521e-18	4	Pbxip1
3.43594071764035e-22	1.62	4.74537772513309e-18	4	Dsp
3.45027387492958e-22	1.62	4.76517324866524e-18	4	Ramp1
1.27317726003399e-21	-1.24	1.75838511383294e-17	4	Dcn
1.58006310415949e-21	1.13	2.18222515315467e-17	4	Tpm1
2.21537423594788e-21	0.920438388318979	3.05965335726762e-17	4	Ccdc3
4.39773505490874e-21	1.03	6.07371188433446e-17	4	Cald1
4.968525128228E-21	-1.80	6.8620300545957e-17	4	Fbln1
6.28793385845536e-21	1.25	8.6842654519127e-17	4	Mylk4
7.05428667368283e-21	-1.83	9.74267532502336e-17	4	Col14a1
7.28812878277664e-21	-2.04	1.00656346618928e-16	4	Cygb
7.44566530602823e-21	1.37	1.02832083541556e-16	4	Hspb6
9.15260886958521e-21	1.09	1.26406681097841e-16	4	Kcnmb1
9.62432243380366e-21	1.32	1.32921517133262e-16	4	Fblim1
2.3146584060559e-20	1.38	3.19677472460381e-16	4	Pkp4
3.4651799833423e-20	0.888751178936024	4.78576007499406e-16	4	Map1b
3.56037964059894e-20	-0.850335230079342	4.91724032163119e-16	4	Sepp1

**4.02850479558436e-20**	1.10	5.56376797318156e-16	4	Itga9
**4.707195694871E-20**	0.885380146347071	6.50110797418633e-16	4	Dmd
**6.22467518084016e-20**	-0.736567127615464	8.59689889225835e-16	4	Sptbn1
**1.11510327472503e-19**	0.903509159686011	1.54006913272274e-15	4	Tns1
**1.31993726589319e-19**	1.24	1.82296535792508e-15	4	Mcam
**1.58655654694892e-19**	0.967215141828337	2.19119324699115e-15	4	Ppp1r12a
**3.47808847504228e-19**	0.962276260477803	4.80358799288089e-15	4	Wwp2
**4.24292980892366e-19**	-1.45	5.85991035910446e-15	4	C1ra
**6.07007608119825e-19**	1.31	8.38338207574291e-15	4	Actg2
**1.56498731675983e-18**	-1.06	0.00	4	Gsn
**2.29537260032688e-18**	1.30	3.17013909831146e-14	4	Entpd1
**2.48567216042939e-18**	1.22	3.43296182076903e-14	4	E030013I19R
**4.17077464521174e-18**	0.90	5.76025686250193e-14	4	Serpine2
**4.19034224824027e-18**	-1.42	5.78728167904464e-14	4	C3
**5.86143073940823e-18**	1.34	8.09522199419671e-14	4	Pcp4l1
**6.33319631796474e-18**	0.925240884095843	8.7467774347411e-14	4	Dstn
**6.99944582937295e-18**	1.25	9.66693463494698e-14	4	Sgcg
**8.78170246832207e-18**	0.790434464870033	1.21284092789996e-13	4	Pgm5
**9.84437948201148e-18**	1.01	1.35960725026061e-13	4	Aoc3
**1.0550202416832e-17**	-1.88	1.45708845578867e-13	4	Gpx3
**1.19684510406809e-17**	-0.940532916913993	1.65296277322843e-13	4	Ctsl
**1.5474294216763e-17**	-1.12	2.13715477427714e-13	4	Ly6e
**2.70199480170873e-17**	-0.980217896519157	3.73172502063993e-13	4	Cd34
**2.77930996491657e-17**	-0.68	3.83850499254627e-13	4	Ppib
**2.83785163728874e-17**	1.11	3.91935689625949e-13	4	Csrp1
**4.07220465640938e-17**	0.862981155511904	5.62412185096699e-13	4	Lpp
**4.47827758509807e-17**	1.09	6.18494917277895e-13	4	Slmap
**4.75265196811946e-17**	-0.947611256343615	6.56388763316978e-13	4	Lrp1
**4.77361272027984e-17**	-1.16	6.59283652797849e-13	4	Lum
**5.53532909324215e-17**	1.15	7.64484301067673e-13	4	Dmpk
**1.53871930080454e-16**	1.68	2.12512522634115e-12	4	Itgb3
**1.65435392117813e-16**	-0.843937849053841	2.28482820053912e-12	4	Ccdc80
**2.18635208877245e-16**	1.18	3.01957086980364e-12	4	Osbpl3
**2.45681045719207e-16**	1.27	3.39310092242796e-12	4	Itih4
**2.97052666093175e-16**	-1.67	4.10259437141284e-12	4	Pdgfra
**3.25778922211286e-16**	1.84	4.49933269466007e-12	4	Net1
**3.71678859351504e-16**	1.07	5.13325672650363e-12	4	Mustn1
**4.04201950889471e-16**	1.09	5.58243314373448e-12	4	Pdlim3
**5.15902862995779e-16**	1.74	7.1251344408347e-12	4	Trabd2b
**6.12597790475225e-16**	-1.99	8.46058808425333e-12	4	Abca8a
**6.37012101036218e-16**	-0.371469078658309	8.79777412741121e-12	4	B2m
**7.6973810561037e-16**	1.26	1.06308529765848e-11	4	Rcan2
**9.57939422794401e-16**	-1.01	1.32301013682135e-11	4	Npc2
**9.60707137634962e-16**	-0.483708640435184	1.32683262778765e-11	4	H2-D1
**1.05329501389179e-15**	-0.89	1.45470574368596e-11	4	Lamp2
**1.1531640108194e-15**	-1.90	1.59263481534267e-11	4	Entpd2
**1.22189667212339e-15**	-0.473317068049283	1.68756149386961e-11	4	Cst3
**1.51498998128521e-15**	1.35	2.09235266315301e-11	4	Mrvi1
**1.54119692311196e-15**	0.889932234742431	2.12854707050992e-11	4	Actn1
**1.86592025003727e-15**	-1.47	2.57702245732647e-11	4	Slc43a3
**2.32921729523204e-15**	1.09	3.21688200644497e-11	4	Xirp1
**3.0145445980109e-15**	0.964551972296041	4.16338754431285e-11	4	Rassf3
**4.1987965143005e-15**	1.08	5.79895786590042e-11	4	Enah
**6.19161644154933e-15**	-0.667124207759602	8.55124146742378e-11	4	Anxa5
**8.64239383468443e-15**	0.694656279120412	1.19360101250827e-10	4	Tspan2
**1.42124037816792e-14**	1.24	1.96287508628771e-10	4	Jph2

**1.56320624295703e-14**	-2.07	2.15894414214795e-10	4	Smoc2
**1.57338883699791e-14**	-0.941041393288764	2.17300732277782e-10	4	S100a10
**2.27725151390697e-14**	0.918324637888187	3.14511206585691e-10	4	Actn4
**2.68106020511929e-14**	-0.48	3.70281224929025e-10	4	Rps5
**2.69532108921333e-14**	-1.97	3.72250795631254e-10	4	Il33
**2.75738320685823e-14**	0.721126537524936	3.80822194699191e-10	4	Cpe
**2.86079882042916e-14**	1.54	3.95104925089471e-10	4	5930403L14F
**4.27808133898564e-14**	-0.891994869506175	5.90845813727306e-10	4	Ly6a
**4.950488780077E-14**	0.851198419068353	6.83712005416434e-10	4	Pcdh7
**5.07791823262446e-14**	1.15	7.01311287107764e-10	4	Ppp1r14a
**5.77606602236509e-14**	-0.413001552782163	7.97732478348843e-10	4	Ltbp4
**8.25536179493028e-14**	1.01	1.14014801749782e-09	4	Lims2
**9.45034453595555e-14**	-1.63	1.30518708386082e-09	4	Cxcl1
**9.94412809879989e-14**	1.64	1.37338353172525e-09	4	Grip2
**1.2559954861457e-13**	-1.64	1.73465536591582e-09	4	Add3
**1.31375284056273e-13**	-0.252467378404349	1.81442404810119e-09	4	Timp2
**1.8808703714347e-13**	0.797830704848691	2.59767006998846e-09	4	Gucy1b3
**1.88643607419436e-13**	-0.822626295499777	2.60535686206983e-09	4	Mfap5
**2.42846233755576e-13**	-0.916103587717425	3.35394933439825e-09	4	Ppap2b
**2.76124124025934e-13**	0.956233417771009	3.81355027692218e-09	4	Cdh13
**2.77583603072237e-13**	-1.45	3.83370714203067e-09	4	C1s1
**2.80515661461895e-13**	-1.44	3.87420180045022e-09	4	Igf1
**2.82620144353169e-13**	-0.510202220219208	3.90326681366162e-09	4	Nfkbia
**3.55753262462831e-13**	2.21	4.91330830787416e-09	4	Rgs7bp
**6.0693048237334e-13**	0.850133991825046	8.3823168920582e-09	4	Pls3
**6.42798307100382e-13**	-1.75	8.87768741936337e-09	4	Cpxm1
**6.57922260595271e-13**	-0.453165064882906	9.08656434108128e-09	4	Emp1
**7.46306366819017e-13**	-1.27	1.03072372321374e-08	4	Cxcl12
**7.66912942626273e-13**	-1.88	1.05918346506115e-08	4	Cfb
**7.99011141910063e-13**	-1.33	1.10351428809199e-08	4	Mmp3
**8.6158373010207e-13**	-1.50	1.18993328964397e-08	4	Marcks
**1.10179938654102e-12**	-1.55	1.52169513275181e-08	4	Bicc1
**1.18226581086681e-12**	-0.916458456135863	1.63282731138815e-08	4	Tcf4
**1.18554220675488e-12**	0.91	1.63735234174916e-08	4	Nes
**1.28353427803061e-12**	-1.62	1.77268919138807e-08	4	Pi16
**1.42781034888913e-12**	1.09	1.97194887285078e-08	4	Cdo1
**1.47543815366604e-12**	0.817652527108113	2.03772763402817e-08	4	Lrrfip1
**1.55972879006229e-12**	-0.503513886907136	2.15414143195503e-08	4	Rplp0
**1.84696356697353e-12**	-0.468324837581166	2.55084138234714e-08	4	Rps18
**1.90496882615424e-12**	-0.337765650660974	2.63095244580162e-08	4	Selm
**1.90733795847905e-12**	-1.73	2.63422445445541e-08	4	Olfml3
**1.9201458653778e-12**	1.07	2.65191345467328e-08	4	Limch1
**1.99783618259939e-12**	1.00	2.75921155178801e-08	4	Plin4
**2.12663210654161e-12**	-1.96	2.93709160234461e-08	4	Dpep1
**2.28810619381067e-12**	1.59	3.16010346427192e-08	4	Ivd
**2.54961490111286e-12**	0.982291417206202	3.52127313992698e-08	4	Adcy5
**3.04330706586837e-12**	0.853831622506735	4.20311138867081e-08	4	Sun2
**3.57149642073675e-12**	2.32	4.93259370667953e-08	4	Fbxo32
**3.99741257075239e-12**	-0.342940339988498	5.52082650146613e-08	4	Ifitm2
**4.54588019348661e-12**	0.977538463729247	6.27831513522435e-08	4	Mfge8
**4.70675730991947e-12**	0.753930244485465	6.50050252072978e-08	4	Vcl
**5.05263825135872e-12**	1.40	6.97819868895154e-08	4	2900026A02F
**5.55567310253219e-12**	-0.446563522889901	7.67294012190721e-08	4	Rps3
**6.24401227829593e-12**	0.549911202613995	8.62360535755451e-08	4	Utrn
**6.98063464532724e-12**	0.670383729973608	9.64095450866145e-08	4	Mef2c
**8.39869018170945e-12**	1.27	1.15994310099589e-07	4	Gm13889

**8.94288846415527e-12**	-1.64	1.23510232578448e-07	4	C7
**9.37126328952527e-12**	1.42	1.29426517291634e-07	4	Mtus2
**1.04160857739895e-11**	-0.608532055840049	1.43856560624569e-07	4	Col3a1
**1.13768229224246e-11**	-1.78	1.57125301381606e-07	4	Gfpt2
**1.25214455855403e-11**	0.508701630558147	1.72933684981898e-07	4	Kcnma1
**1.35155698485325e-11**	-1.76	1.86663535178083e-07	4	Scn7a
**1.48882269628808e-11**	1.37	2.05621302584347e-07	4	Mmp17
**1.54831316490779e-11**	0.946616797783108	2.13837531205414e-07	4	Myh10
**1.58643120170443e-11**	-0.260119074412124	2.19102013267399e-07	4	Rps4x
**1.73157686322673e-11**	-0.549069121190739	2.39148080580243e-07	4	Bsg
**1.81193864623805e-11**	1.25	2.50246846431938e-07	4	Rgs4
**1.86216975402305e-11**	1.32	2.57184264728124e-07	4	Pawr
**2.4544560295277e-11**	0.837293943206202	3.38984922238071e-07	4	Plcb4
**2.6697339323121e-11**	-1.49	3.68716953391625e-07	4	C4b
**3.03311705830569e-11**	0.893361231354697	4.18903796922598e-07	4	Nexn
**3.3165880107084e-11**	-0.40	4.58053970158937e-07	4	Cd81
**4.0126131118131e-11**	-0.586313414120636	5.54181996872507e-07	4	Zfp36
**4.72234682667025e-11**	0.87	6.52203320231428e-07	4	Rrad
**4.73354222653403e-11**	-1.19	6.53749516906615e-07	4	Fbln2
**5.0986369729736e-11**	1.59	7.04172752337384e-07	4	Fam13c
**5.13455226115673e-11**	-1.79	7.09133012788356e-07	4	Tgfbi
**5.19421268652542e-11**	-1.21	7.17372714136025e-07	4	Mgst1
**5.25961717201888e-11**	0.844173207807563	7.26405727627528e-07	4	Nox4
**6.34198293883195e-11**	0.865323951979183	8.75891263682081e-07	4	Pdlim7
**6.39283164555942e-11**	-1.07	8.82913978568211e-07	4	Nucb2
**6.99675839565644e-11**	0.699536978658716	9.6632230202411e-07	4	Tbc1d1
**8.40386446700944e-11**	1.56	1.16065772153867e-06	4	Kcnab1
**1.13480138836213e-10**	-0.799961378362012	1.56727419746694e-06	4	Cd302
**1.14419470193976e-10**	-1.52	1.58024730284901e-06	4	Ifi27l2a
**1.20623920183881e-10**	1.05	1.66593696165958e-06	4	Sorl1
**1.21675430005933e-10**	0.637605809291217	1.68045936381194e-06	4	Samd4
**1.25625259697332e-10**	-0.923431320800654	1.73501046167985e-06	4	Tgfbr2
**1.40098764096155e-10**	0.741684453817826	0.00	4	Hspb2
**1.41246166707932e-10**	-0.912901933875391	1.95075080840325e-06	4	Mtch1
**1.56491774756664e-10**	-0.839283918611282	2.16130790116429e-06	4	Ctsz
**1.64773106420832e-10**	-0.454939076181638	2.2756813727781e-06	4	Rpl14-ps1
**1.75516788644481e-10**	0.909712669957527	2.42406236796893e-06	4	Acsl1
**1.85061215651828e-10**	0.690212839922092	2.55588044936739e-06	4	Perp
**1.92933034378678e-10**	-0.311927604862711	2.66459813780393e-06	4	Cd63
**2.02753882014683e-10**	1.08	2.80023386450478e-06	4	Prdm16
**2.12708385899384e-10**	-0.330552970919783	2.93771551765639e-06	4	Rps14
**2.18068076900988e-10**	-1.79	3.01173821007955e-06	4	Clec3b
**2.2373490153823e-10**	-1.57	3.0900027251445e-06	4	Lpar1
**2.41483863644938e-10**	-0.922300037037999	3.33513364080023e-06	4	Ccnl1
**2.52334703377061e-10**	0.820559913361105	3.48499458834059e-06	4	Rock1
**3.19904775935945e-10**	-0.50	4.41820486045133e-06	4	Gabarap
**3.37514172037592e-10**	1.70	4.66140823001118e-06	4	Gpsm2
**3.44468910342867e-10**	-0.418666820434651	4.75746012074534e-06	4	Fosb
**3.46968225650775e-10**	-0.778398964089853	4.79197816446285e-06	4	Il6st
**3.51424564794484e-10**	1.27	4.85352466437661e-06	4	1190005I06R
**3.53641781471066e-10**	0.631849345294794	4.88414664389689e-06	4	Syne1
**3.56607981333563e-10**	-1.40	4.92511283019784e-06	4	Gda
**3.79229114403356e-10**	-0.692624195348506	5.23753329902474e-06	4	Rplp2
**3.83549170564463e-10**	0.741754879625322	5.2971975946658e-06	4	Map1a
**4.00855286500498e-10**	-1.98	5.53621236185838e-06	4	Thy1
**4.06068625153343e-10**	-0.447033073507551	5.60821378199282e-06	4	Col1a2

**4.23289165952581e-10**	-1.12	5.84604667097109e-06	4	Fuca1
**4.56260743486686e-10**	-0.845978975780085	6.30141712829462e-06	4	Pdia6
**4.62656420921527e-10**	1.03	6.38974782934721e-06	4	Fmo3
**4.73542512274687e-10**	0.603266475178073	6.5400956370257e-06	4	Pde4dip
**5.10164617733296e-10**	-1.75	7.04588353551455e-06	4	Aldh1a1
**5.3745445648126e-10**	-1.97	7.42278349846268e-06	4	Scara5
**5.61280004106288e-10**	0.797129068607452	7.75183813671194e-06	4	Uchl1
**5.73045127278225e-10**	-0.551650448137212	7.91432625283956e-06	4	Anxa2
**5.78038356975828e-10**	-0.362152382724374	7.98328774819316e-06	4	Hspa5
**5.97879392686548e-10**	-0.808344200441042	8.25731229239392e-06	4	Nrp1
**6.18721852511008e-10**	1.74	8.54516750502954e-06	4	Filip1
**7.30659412394653e-10**	0.936971453678694	1.00911371445825e-05	4	Smarcd3
**8.54389599292059e-10**	-1.16	1.17999747558226e-05	4	Prg4
**8.5852377273847e-10**	-0.887593059006502	1.1857071825291e-05	4	Cd47
**8.9011200054336e-10**	0.554585671347093	1.22933368395043e-05	4	Actb
**1.0054956674641e-09**	-0.813867054487435	1.38869006633467e-05	4	Cp
**1.03873883643983e-09**	1.35	1.43460220700706e-05	4	Efhd1
**1.05478276829813e-09**	-0.572876683799429	1.45676048129654e-05	4	Mt1
**1.178371165925E-09**	-0.504661630116368	1.62744841725902e-05	4	Tmem59
**1.26873540908375e-09**	-0.667182634620867	1.75225047348557e-05	4	Cyb5
**1.36151794376311e-09**	0.553930112085403	1.88039243213123e-05	4	Fn1
**1.47247622938458e-09**	1.21	2.03363692040304e-05	4	Bcam
**1.48866966310505e-09**	-0.272268267060929	2.05600167171438e-05	4	Rps11
**1.54557238754227e-09**	0.622882019664133	2.13459002443462e-05	4	Tmsb4x
**1.71908884991167e-09**	0.572933659752502	2.37423361061301e-05	4	Csrp2
**1.74198152348457e-09**	0.834241345566168	2.40585068208454e-05	4	Pdlim1
**1.78514268786157e-09**	-0.436437923734414	2.46546056620561e-05	4	Nfib
**1.84494763140447e-09**	-1.78	2.54805717373271e-05	4	Fxyd6
**1.97522180893511e-09**	0.875535562172662	2.72797884032027e-05	4	Ldb3
**2.05267501139311e-09**	1.27	2.83494945823502e-05	4	Kcnc4
**2.29740375840769e-09**	-1.85	3.17294433073686e-05	4	Fmod
**2.71683193840987e-09**	-0.844161832728856	3.75221659013788e-05	4	Htra3
**2.74008744073269e-09**	-0.547359407592075	3.78433476439592e-05	4	Mt2
**2.81559063552954e-09**	1.03	3.88861222672984e-05	4	Fgf1
**2.84131474027559e-09**	0.792964225280639	3.92413978779461e-05	4	Lmcd1
**2.85077444804711e-09**	0.721153939942337	3.93720459019787e-05	4	Hdac11
**2.8872053392294e-09**	-0.738316616099037	3.98751929400972e-05	4	Nid1
**2.8886310823708e-09**	-1.12	3.98948838786231e-05	4	Abi3bp
**3.58847134874374e-09**	-1.02	4.95603777974997e-05	4	Hexa
**3.66229055997961e-09**	-0.487126013434756	5.05798949238784e-05	4	Icam1
**3.84210920450167e-09**	0.744680830675128	5.30633702233726e-05	4	Phtf2
**3.94447071861137e-09**	1.14	5.44770850947416e-05	4	Ryr2
**3.94588552101668e-09**	0.451774067621021	5.44966249307614e-05	4	Endod1
**4.82632355350622e-09**	-0.287851525683094	6.66563545974745e-05	4	Hsp90b1
**5.14750948400965e-09**	1.22	7.10922534836573e-05	4	Rgs2
**5.25859807025278e-09**	1.15	7.26264979482612e-05	4	Pdgfa
**5.29835864502047e-09**	-0.325888661699531	7.31756312463777e-05	4	Rpl32
**5.35317317936696e-09**	0.973127645342121	7.3932674780237e-05	4	Cacna1d
**6.26082485618554e-09**	-1.21	8.64682520887785e-05	4	Thbd
**7.29720660423885e-09**	-0.579388523164224	0.000100781720411143	4	Sh3glb1
**8.07298210166524e-09**	-0.321528033938322	0.000111495955806099	4	Fgfr1
**9.53992773319851e-09**	1.03	0.000131755941923205	4	Msrb1
**9.90423222639712e-09**	0.548355973415712	0.000136787351278771	4	Eln
**9.91428713298629e-09**	-0.491401183896229	0.000136926219593674	4	Arf4
**1.13628632589675e-08**	-0.727503776720763	0.0001569325044696	4	Fcgrt
**1.14734854492946e-08**	0.466258892534348	0.000158460307540208	4	Atp2b4

**1.23490459054598e-08**	0.594536203407714	0.000170552673000306	4	Ltbp1
**1.26905246811405e-08**	-1.63	0.000175268836371232	4	Gpm6b
**1.32937374410091e-08**	-0.411287829272727	0.000183599807797777	4	Grn
**1.34117879668444e-08**	1.28	0.000185230203610088	4	Inpp5a
**1.40008302392907e-08**	-1.48	0.000193365466434844	4	Osmr
**1.41254457138727e-08**	0.82	0.000195086530754296	4	Postn
**1.62689866392824e-08**	1.05	0.000224690974475129	4	Pde5a
**1.63743993392714e-08**	-0.401496978002948	0.000226146829274678	4	Lmo4
**1.75600621940548e-08**	-0.650135070252538	0.000242522018962091	4	Adamts2
**1.84921593105891e-08**	0.463956230191331	0.000255395212238546	4	Prpsap1
**1.86567173034598e-08**	0.606619594532861	0.000257667922678084	4	Cacna2d1
**1.89277240224993e-08**	0.785414315397524	0.000261410796474737	4	Msrb3
**1.90011017364644e-08**	-0.796815402359636	0.00026242421608231	4	Sri
**1.95678124523048e-08**	1.12	0.000270251057778782	4	Stom
**1.95792897828071e-08**	-0.643353932568778	0.000270409571190349	4	Nfkbiz
**2.08653581304105e-08**	0.748166897530638	0.000288171461139099	4	Rbpms
**2.13863909939597e-08**	-0.488330288780187	0.000295367446017577	4	Rps19
**2.15915695098772e-08**	-0.433149303298974	0.000298201166500914	4	Gm6251
**2.26911394195381e-08**	-1.54	0.000313387326523241	4	Clec11a
**2.294732931783E-08**	0.660765693605758	0.00031692556520855	4	Ctgf
**2.30413938077603e-08**	1.11	0.000318224689878978	4	Gid4
**2.37121168513953e-08**	-1.69	0.000327488045834621	4	Ctsk
**2.44582257394239e-08**	1.02	0.000337792555687183	4	Zak
**2.53373464045518e-08**	-0.304469805726306	0.000349934091193265	4	Gm1821
**2.70138591841574e-08**	0.896202515339266	0.000373088409192398	4	Mgll
**2.72756086719681e-08**	-0.519697678061945	0.000376703431368551	4	Col1a1
**2.9958424317463e-08**	-1.75	0.000413755798248482	4	Lpl
**3.25994332570832e-08**	-1.54	0.000450230772713576	4	Dab2
**3.33928984255677e-08**	-0.54	0.000461189320155515	4	Vkorc1
**3.7116016046967 1e-08**	-1.91	0.000512609297624662	4	Inmt
**3.83793612786584e-08**	0.641234439098672	0.000530057358619552	4	Kank1
**3.97145768712897e-08**	0.739219529554839	0.000548498021169382	4	Xpr1
**4.01297395324422e-08**	-0.95	0.000554231832682559	4	Crispld2
**4.0759873695514e-08**	0.771071110301862	0.000562934615608744	4	Sh3bgrl
**4.17136176062206e-08**	1.00	0.000576106772759513	4	Myocd
**4.47124864537914e-08**	-0.282015872998337	0.000617524150413313	4	Rps26
**4.54877757136975e-08**	1.43	0.000628231670381876	4	Tnk2
**4.65606978565279e-08**	-0.730560543983892	0.000643049798096507	4	Adamts5
**4.69562713264931e-08**	-1.15	0.000648513063290196	4	Serpina3n
**4.73667448459882e-08**	1.02	0.000654182113067942	4	Filip1l
**4.7825451802707e-08**	-1.14	0.000660517314847187	4	Timp1
**5.16554721166169e-08**	1.34	0.000713413725402596	4	Gcnt2
**5.19009419667267e-08**	0.621782694351974	0.000716803909502462	4	Marveld1
**5.54733530801084e-08**	-0.519088734343057	0.000766142479389378	4	Rarres2
**5.6838330824879e-08**	-0.414762309200361	0.000784994187022404	4	Cpxm2
**5.82263130769628e-08**	-1.35	0.000804163609905934	4	Ly6c1
**5.83257383490865e-08**	-1.57	0.000805536772339234	4	Lgals3bp
**6.62212856445953e-08**	0.693439815473379	0.000914582176037505	4	Arhgef17
**6.80201542255475e-08**	-1.73	0.000939426350009037	4	Bace2
**7.01574595677645e-08**	-0.406008604234949	0.000968944674090396	4	Calr
**7.21176997428009e-08**	-0.790868002238373	0.000996017551147823	4	Cyp1b1
**7.31972961725884e-08**	0.796838090240764	0.00101092785743962	4	Phldb2
**7.66187376785351e-08**	-0.849181480424402	0.00105818138607825	4	Ergic3
**8.9937376342239e-08**	-0.96	0.00124212510466266	4	Igfbp5
**9.00720193336711e-08**	0.680002846960473	0.00124398465901733	4	Gne
**9.16085737397089e-08**	0.931767665101584	0.00126520601191912	4	Itih2

**9.76239633249302e-08**	0.50	0.00134828455748061	4	Slc22a1
**9.78022849031741e-08**	0.425865344316241	0.00135074735679774	4	Fam71a
**9.86965378849781e-08**	-1.05	0.00136309788472943	4	Cebpd
**1.0240087165713e-07**	0.638318070179746	0.00141425843845662	4	Synm
**1.1088892133773e-07**	0.419395463471273	0.0015314868925954	4	Cav1
**1.12173673019392e-07**	-1.80	0.00154923059807082	4	Tmem100
**1.14180124191999e-07**	1.14	0.0015769416952157	4	Rbpms2
**1.16616490727316e-07**	-0.258017273319734	0.00161059035343496	4	Rpl4
**1.22456135532814e-07**	-1.46	0.00169124168784369	4	Pltp
**1.24797290198973e-07**	0.821437586395644	0.00172357537493801	4	Oxct1
**1.25069172195661e-07**	-1.16	0.00172733033719427	4	Ackr3
**1.30329249651125e-07**	0.693006666812382	0.00179997726693169	4	Pi15
**1.30408680415137e-07**	-0.25	0.00180107428521346	4	Sec62
**1.34177588814005e-07**	0.606353265787112	0.00185312667911023	4	Pdlim4
**1.35826321517986e-07**	-0.624097670765212	0.0018758973264849	4	Shisa5
**1.43291509371433e-07**	0.716263710258066	0.00197899903592887	4	Unc5c
**1.44398983765902e-07**	-1.71	0.00199429436479087	4	Pdpn
**1.45091522872618e-07**	-1.63	0.00200385902239373	4	Efemp1
**1.66001276895513e-07**	-0.758801623098967	0.00229264363520393	4	Lama2
**1.91231218124851e-07**	0.511171027027729	0.00264109435352231	4	Hip1
**1.92293505961405e-07**	-0.334803509538698	0.00265576561083296	4	Rpl14
**1.92359910524125e-07**	0.981785428383382	0.00265668272424869	4	Tnc
**2.05738104893375e-07**	-0.264073736269595	0.0028414489666824	4	Ddx5
**2.10879755135735e-07**	0.444729789088815	0.00291246029817964	4	Dpysl3
**2.13888134347867e-07**	-0.684266907516258	0.00295400902347839	4	Col5a2
**2.1647992923139e-07**	1.16	0.00298980430261472	4	Zbtb38
**2.49020638211236e-07**	-1.81	0.00343922403433539	4	Gdf10
**2.54987222428747e-07**	-1.86	0.00352162852896343	4	Igfbp6
**2.56168927808482e-07**	-1.06	0.00353794906196295	4	Man2a1
**2.56224081136101e-07**	1.08	0.00353871078457069	4	Hyal1
**2.56544538111827e-07**	0.532556346790098	0.00354313661586244	4	Ehbp1l1
**2.63902933683424e-07**	-1.49	0.00364476341710177	4	Medag
**2.70135678970562e-07**	1.29	0.00373084386226243	4	Fgfr2
**2.8256018461667e-07**	-1.64	0.00390243870974082	4	Dnm1
**3.12717704414724e-07**	0.309241053094489	0.00431894421567175	4	Tbc1d10b
**3.15369241601938e-07**	-0.34	0.00435556459576436	4	Rpl13a
**3.26986529578215e-07**	-1.67	0.00451601096000472	4	Ccl7
**3.27663333748331e-07**	0.636602369216597	0.0045253583023982	4	Cyb5r3
**3.31454345448113e-07**	-1.73	0.00457771596498388	4	Ms4a4d
**3.53685354010715e-07**	1.33	0.00488474842424198	4	Kcnh2
**3.63720775905584e-07**	0.616574065474982	0.00502334763603202	4	Tgfb1i1
**3.68635975435593e-07**	0.417863621800497	0.00509123145674097	4	St5
**3.75713932815057e-07**	0.537722489451737	0.00518898512610876	4	Gucy1a3
**3.81107036437868e-07**	-0.692526885618016	0.0052634692802434	4	Ace
**4.17919001221601e-07**	0.463698218555983	0.00577187932587153	4	Sorbs2
**4.21681840077134e-07**	0.313713243925477	0.0058238478933053	4	Emb
**4.44794797235637e-07**	-0.783823769900103	0.00614306094462138	4	Bhlhe40
**4.58263329433817e-07**	0.629950458815392	0.00632907484281044	4	Ptgis
**4.7598809454856e-07**	-0.488454896664567	0.00657387157381016	4	Plxdc2
**4.83339377273725e-07**	-0.678837043472771	0.00667540013952742	4	Itm2c
**5.05224715525962e-07**	0.833042200643354	0.00697765854612906	4	Tacc2
**5.14987308124727e-07**	-1.40	0.00711248971251061	4	Srpx
**5.29099241592399e-07**	1.19	0.00730738962563262	4	Spint2
**5.49555867056813e-07**	-0.25	0.00758991607992164	4	App
**5.6294166183355e-07**	-1.31	0.00777478729158316	4	Slfn5
**5.75641987592313e-07**	0.546967955072414	0.00795019149063743	4	Nacc2

**5.91890146623454e-07**	-0.798980681362855	0.00817459481501653	4	Pcolce2
**6.01038640388493e-07**	-0.588731645017277	0.00830094466240548	4	Hsd17b11
**6.25800296271982e-07**	1.11	0.00864292789181235	4	Dysf
**6.27008244055372e-07**	-1.04	0.00865961085864874	4	Pkd2
**6.32450762882505e-07**	0.449472208836997	0.00873477748617028	4	Tinagl1
**6.56580913923153e-07**	1.08	0.00906803900219267	4	Itga7
**6.68003634251958e-07**	0.367739751417865	0.0092257981926538	4	Trak2
**6.93202908694081e-07**	-1.36	0.00957382537197396	4	Litaf
**7.33956361271522e-07**	-0.585615459994119	0.010136671305521	4	Ssr3
**7.7482201410233e-07**	0.819744391345938	0.0107010668367673	4	Npy1r
**7.95125127311387e-07**	0.531477495183474	0.0109814731332976	4	Fhl1
**8.38601234968168e-07**	-0.776468793698684	0.0115819216561454	4	Stat3
**8.56505538703322e-07**	0.590059122278281	0.0118291979950316	4	Sema3f
**9.18779215862359e-07**	-0.515279718663028	0.012689259750275	4	Rpl7
**9.98809264858153e-07**	-1.90	0.013794554756956	4	Hsd11b1
**1.00536281283359e-06**	-0.582986663113584	0.0138850658080447	4	Myl12b
**1.08363531750313e-06**	0.468988721505641	0.0149660873700357	4	Hdac5
**1.11100274074109e-06**	-1.20	0.0153440588523752	4	Matn2
**1.1332979979847e-06**	-0.797716940503969	0.0156519786501666	4	Epb4.1l2
**1.15650723194227e-06**	0.761778021335417	0.0159725213803547	4	Stk38l
**1.29632575824572e-06**	-0.306493121273288	0.0179035550471317	4	Tuba1a
**1.32106618693855e-06**	-0.282010334077726	0.0182452451078083	4	Tapbp
**1.33863676485589e-06**	-0.282544985094465	0.0184879123594247	4	Mettl7a1
**1.33962527401626e-06**	1.00	0.0185015646594385	4	Neo1
**1.36595808023551e-06**	0.688784538255243	0.0188652470461326	4	Nudt4
**1.3899589982085e-06**	-0.337715830971405	0.0191967237242575	4	Cox4i1
**1.39933555245627e-06**	-1.73	0.0193262233149735	4	Il6
**1.45136716678751e-06**	1.25	0.0200448319405023	4	6330403A02F
**1.54784419968039e-06**	1.14	0.0213772762417859	4	Plcd1
**1.5524730370057e-06**	-1.05	0.0214412051140857	4	Pdlim2
**1.59844047623909e-06**	1.14	0.0220760614173381	4	Prkg1
**1.60911594294853e-06**	1.06	0.0222235002880621	4	2200002D01I
**1.63677479566878e-06**	-0.343203568945458	0.0226054967029815	4	H3f3b
**1.64234377303624e-06**	0.535113299723411	0.0226824098494036	4	Slc25a23
**1.64443221210731e-06**	0.776089856457934	0.0227112532814141	4	Serpini1
**1.68953351644732e-06**	0.905345767845123	0.023334147395654	4	Cd97
**1.76840470053903e-06**	0.436761854681174	0.0244234373191446	4	Clu
**1.77209622031079e-06**	-1.55	0.0244744208987123	4	Igsf10
**1.80090502418022e-06**	-0.638669679387152	0.024872299288953	4	Sdf4
**1.81177689823372e-06**	0.807745201814247	0.0250224507415059	4	Rgs5
**1.85056386194088e-06**	0.439333061053479	0.0255581374972655	4	Mob2
**1.8768753653102e-06**	-0.872419954504541	0.0259215256702992	4	Gas1
**1.93202776387961e-06**	-0.521111789873616	0.0266832354469413	4	Ssr4
**2.01366448741599e-06**	-0.801729202042549	0.0278107202357023	4	B4galt1
**2.1527824009987e-06**	0.585850685125911	0.0297320777401931	4	Plekha3
**2.17435032662526e-06**	-0.457115694228212	0.0300299523610215	4	Tmsb10
**2.21965933843099e-06**	-0.597825166015925	0.0306557151230704	4	Dnajc3
**2.22326918402838e-06**	-0.405141908712404	0.030705570700616	4	Zeb2
**2.25303801824082e-06**	-0.56	0.031116708069924	4	Lgals9
**2.27921206825573e-06**	0.685291440706146	0.0314781978746799	4	Cnn2
**2.45634748119036e-06**	0.374835085237409	0.03	4	Ptplad2
**2.45899732173975e-06**	-1.49	0.0339612120105476	4	Apod
**2.51813394010461e-06**	0.716408810793407	0.0347779478467847	4	Zc2hc1a
**2.67931940101186e-06**	-0.369736420058774	0.0370040802473749	4	Tmem50a
**2.70606095410331e-06**	-0.335307968919841	0.0373734078371209	4	Nos3
**2.76876267856535e-06**	0.996624942246156	0.038239381353666	4	Tfrc

**2.83996343277663e-06**	-0.254883997407911	0.039222734970078	4	Prdx1
**2.88198880014016e-06**	-1.03	0.0398031473187358	4	Aspn
**2.94896622522781e-06**	0.498613724747595	0.0407281725366213	4	Aplp2
**2.95553063413087e-06**	1.31	0.0408188335879814	4	Mark1
**3.03014618796299e-06**	0.354119128069539	0.0418493490019569	4	Slc4a3
**3.10179689028923e-06**	-0.25	0.0428389168517845	4	Tram1
**3.14173615702956e-06**	-0.849726268012911	0.0433905180647353	4	Sash1
**3.16019701160784e-06**	0.82	0.0436454809273158	4	Ethe1
**3.18451783913827e-06**	0.456532206197047	0.0439813758763386	4	Zyx
**3.28278313591166e-06**	-0.546338994740191	0.045338517890076	4	Gnb1
**3.28447809810375e-06**	-0.425506217209954	0.045361927012911	4	Emp3
**3.45590251030398e-06**	-0.705649884086447	0.0477294695698083	4	Eif3f
**3.53413849156856e-06**	0.583646519160979	0.0488099867070534	4	Rasal2
**3.54671149402659e-06**	0.913439582275703	0.0489836324440013	4	Ptpla
**3.61028932936962e-06**	-0.411475808946395	0.0498617059279239	4	Ywhae
**3.70889694338153e-06**	-1.48	0.0512235756850423	4	Comp
**3.72217056255812e-06**	-1.63	0.0514068976394902	4	Pdgfrl
**3.72769691635558e-06**	0.69	0.0514832221117869	4	Fry
**3.73226947227053e-06**	-0.458389343625644	0.0515463736815282	4	Col6a1
**3.89655897512112e-06**	-1.63	0.0538153760053978	4	Lbp
**3.99705221998563e-06**	1.27	0.0552032882102215	4	Tpd52
**4.1205401657131e-06**	1.33	0.0569087802286637	4	Adck3
**4.12699803355196e-06**	0.357260953294247	0.0569979698413861	4	Nf2
**4.16711813877707e-06**	0.785642188306193	0.0575520686146501	4	Ptp4a3
**4.20797773399108e-06**	0.675503729268717	0.0581163804841508	4	Nrtn
**4.33031680067573e-06**	-0.554728078914835	0.0598060053341325	4	Smdt1
**4.78988227128162e-06**	-1.42	0.0661530640486705	4	Tnfaip6
**5.1116057068235e-06**	-0.37	0.0705963864169394	4	Vcan
**5.31078815202576e-06**	-0.757432314831212	0.073347295167627	4	Fkbp7
**5.52275372986456e-06**	-1.46	0.0762747517631595	4	Tmem119
**6.12101221542147e-06**	0.836415831397256	0.0845372997071859	4	Npr1
**6.27060435307219e-06**	0.55	0.09	4	Parm1
**6.28655223983877e-06**	0.698335655498763	0.0868235729844132	4	Nqo1
**6.47945252380124e-06**	-0.484078023040964	0.0894877188062189	4	Cnbp
**6.70040578853E-06**	-1.63	0.0925393043453878	4	Rftn1
**6.94075997231601e-06**	-0.636708597017112	0.0958588359776564	4	Tmem176b
**7.06781866253098e-06**	-1.55	0.0976136435482154	4	Dio2
**7.15505193997885e-06**	0.350663699614119	0.098818422343048	4	Boc
**7.17997362992032e-06**	0.421035857389594	0.0991626158028296	4	Hsph1
**7.65851369013323e-06**	0.683287613751428	0.11	4	Chd3
**7.69637799600268e-06**	-0.312192280451684	0.106294676502793	4	Serpinb6a
**7.69651867273332e-06**	-0.410412697420828	0.11	4	Ppap2a
**8.10821075651563e-06**	1.32	0.111982498758237	4	Plekho1
**8.16551633459039e-06**	-1.09	0.112773946097028	4	Csf1
**8.36627161892616e-06**	-0.649034995783987	0.115546577328989	4	Tmed3
**8.42522644075778e-06**	-1.33	0.116360802373306	4	Col16a1
**8.44533059903172e-06**	0.336157482601994	0.116638460903227	4	Tgm2
**9.44804581469724e-06**	0.570689500015116	0.130486960746784	4	Ttll7
**9.94750268746862e-06**	-0.38	0.137384959616629	4	Rpl18a
**9.98508854696635e-06**	0.410212588083613	0.137904057922152	4	Sorbs1
**1.00661256502088e-05**	0.713322566607896	0.139023261355034	4	Dusp3
**1.01237146540637e-05**	0.790084026350634	0.139818623087273	4	Dhrs3
**1.02355584536364e-05**	0.388901967759906	0.141363297803173	4	Gripap1
**1.06570081442202e-05**	-1.44	0.147183939479825	4	H2-T23
**1.09456928502834e-05**	0.34	0.151170963955263	4	Esam
**1.10570401567009e-05**	-1.03	0.152708781604196	4	Akap12

**1.1224221432163e-05**	-1.69	0.155017722199604	4	Svep1
**1.17065119849041e-05**	0.348082151772605	0.161678637023511	4	Ndrg2
**1.17756849195086e-05**	-0.48	0.162633984423333	4	Pcbp2
**1.23590163810366e-05**	0.420541304053389	0.170690375238497	4	Pkdcc
**1.26489467613405e-05**	0.823389083101463	0.174694603720873	4	Kif1c
**1.28886017066413e-05**	1.01	0.178004478170423	4	Fyco1
**1.31620526843619e-05**	-0.414002919062389	0.181781109623722	4	Wwtr1
**1.33426533080654e-05**	-0.929645541660831	0.184275384837691	4	Gaa
**1.3377740658269e-05**	0.271442055546281	0.184759976231353	4	Tln1
**1.3452882481659e-05**	-0.622766383798146	0.185797759954193	4	Hexb
**1.36335149239274e-05**	-0.969547142961402	0.188292474614361	4	Ptgs2
**1.37645696099082e-05**	-0.837724439403292	0.190102470882442	4	Herpud1
**1.42147096797936e-05**	0.50	0.196319355387629	4	Gstk1
**1.47746333273425e-05**	0.577447887770949	0.204052460883928	4	Farsb
**1.51273881123958e-05**	-0.363715123087711	0.208924357220298	4	Gas6
**1.51834959639577e-05**	-1.38	0.21	4	Zfp521
**1.52535744493746e-05**	0.77	0.210667116720312	4	Hspa2
**1.53665406502272e-05**	0.595291821317266	0.212227292920288	4	Nkd1
**1.54831289054993e-05**	-1.73	0.213837493313851	4	Gpnmb
**1.56503396877056e-05**	-1.55	0.216146841426903	4	Aff3
**1.62456243524446e-05**	0.840025505727736	0.224368317931613	4	2310022B05R
**1.65048361782555e-05**	-0.761106406647662	0.227948292457887	4	Ramp2
**1.70600951185206e-05**	-0.830944874130524	0.235616973681889	4	Swi5
**1.70829230800641e-05**	-0.937491684572665	0.235932250658766	4	Ddah2
**1.73009865473768e-05**	0.502204326076916	0.238943925205821	4	Lonp1
**1.73384283250131e-05**	1.42	0.239461033596756	4	Slc24a3
**1.74942044648371e-05**	0.684442365905838	0.241612457863865	4	Xk
**1.75695416697282e-05**	0.799108971572522	0.242652940000617	4	Ptms
**1.79125129215045e-05**	0.816648124343119	0.247389715958899	4	Glipr1
**1.81592274150672e-05**	-0.390406306280597	0.250797089829493	4	Sqstm1
**1.84794200832751e-05**	-0.378599031611055	0.255219270770113	4	Hint1
**1.92963083212442e-05**	-0.340981336191799	0.266501314224704	4	Rac1
**1.94308268559894e-05**	-0.653879897508176	0.27	4	Sdc2
**1.99961348780714e-05**	-0.417820747600261	0.276166618801044	4	Tmed10
**2.23197405822071e-05**	-0.925510977701371	0.308257937180863	4	Spon1
**2.23742739818729e-05**	-1.02	0.309011097963647	4	Anpep
**2.2443600776083e-05**	-0.574118742481528	0.309968570318482	4	Vcam1
**2.34769036045847e-05**	-0.270574943757923	0.324239515682919	4	Rabac1
**2.35375788944314e-05**	-1.17	0.325077502110993	4	Fam102b
**2.49774212432282e-05**	0.717535352580472	0.344963164790224	4	Capzb
**2.52364255577031e-05**	0.582117437959543	0.348540273377437	4	Zbtb6
**2.6651623034993e-05**	-1.08	0.368085565736289	4	Nfkb1
**2.69036909421511e-05**	-0.375498926730228	0.371566875602048	4	Pdia3
**2.70065508892742e-05**	1.09	0.372987474331766	4	Inhba
**2.7328534950051e-05**	0.825206014148078	0.377434396195155	4	Sncg
**2.74873340966872e-05**	0.904793846314766	0.379627571209347	4	Cdc42ep3
**2.75614987505938e-05**	0.650384944212711	0.380651859244451	4	Mcm6
**2.76043033628194e-05**	-0.310738654548415	0.381243033743899	4	Prelp
**2.94602429916318e-05**	-0.707181445314812	0.406875415957427	4	Itgbl1
**3.07393116173023e-05**	-0.692081953033163	0.424540632746562	4	Bmp1
**3.07499440902323e-05**	0.273651366498842	0.424687477830198	4	Flnb
**3.10944396853165e-05**	-1.28	0.429445306493906	4	Fbln7
**3.18851094451999e-05**	-0.795926789564823	0.440365246547656	4	Pid1
**3.20313762537486e-05**	0.400126132159343	0.442385337440522	4	Tbx18
**3.23681631691043e-05**	-0.921662856126327	0.447036701528499	4	Fnip1
**3.2642465242086e-05**	1.27	0.45	4	Lphn2

**3.28848494134494e-05**	-0.965420235201889	0.454172655249149	4	Arpc5
**3.32304220789783e-05**	0.520108790420441	0.458945359332769	4	Alcam
**3.32616368463243e-05**	-1.20	0.459376466484585	4	Jam2
**3.38485013883482e-05**	0.777378113173607	0.467481652674477	4	Me1
**3.42034715837254e-05**	1.03	0.472384146042832	4	Map3k7cl
**3.43171781886175e-05**	0.278270284696499	0.473954547962997	4	Col4a2
**3.70092707851775e-05**	1.06	0.511135038814087	4	Fam129a
**3.75967509541929e-05**	0.775343229576682	0.519248727428358	4	Serpine1
**3.81929779203646e-05**	1.28	0.527483218058155	4	Tmem47
**3.85132309715166e-05**	0.598598257148228	0.531906232947615	4	Sparcl1
**3.85799186867646e-05**	-0.834060969689052	0.532827256982907	4	Cmtm3
**4.08941760923632e-05**	0.312398182110894	0.564789466011629	4	Zfp322a
**4.12440534889509e-05**	1.43	0.569621622735901	4	Tcap
**4.14430468400599e-05**	-0.510004631582455	0.572369919908068	4	Pomp
**4.18986190783851e-05**	0.550619447623473	0.578661828091576	4	Mgat4a
**4.31664771023905e-05**	1.29	0.596172215261115	4	Tmem38b
**4.34725924328056e-05**	0.497611744487015	0.600399974089478	4	Cfl2
**4.38699513562679e-05**	-1.58	0.605887898181416	4	Tcf21
**4.43495427133427e-05**	0.622557096868763	0.612511534413976	4	Myl6
**4.748287383491E-05**	-1.21	0.655785970533942	4	Aldh3a1
**4.98752623596605e-05**	-1.70	0.688827248449272	4	Ptx3
**4.99576553785839e-05**	0.45	0.689965178433623	4	Asap2
**5.55901271550397e-05**	0.601674064836867	0.767755246138254	4	Iars2
**5.61333323591054e-05**	-1.45	0.775257453211605	4	Xdh
**5.62878862400584e-05**	-0.899920088727519	0.777391996861446	4	Mrc2
**5.70370932267707e-05**	-1.23	0.79	4	Tmem204
**5.79448707367373e-05**	-1.66	0.800276609745079	4	Gpr133
**5.83461219141481e-05**	-1.10	0.81	4	Steap4
**5.84833883806858e-05**	0.914223805511984	0.807714076925651	4	Thrb
**5.91232042548773e-05**	0.883890515684325	0.816550573964111	4	Erich1
**6.45074498337596e-05**	0.657553145313103	0.890912389654054	4	Zfp266
**6.71398625842586e-05**	-1.64	0.927268642151195	4	Gsta3
**6.75196878164973e-05**	0.546655203277302	0.932514408433644	4	Hbegf
**6.83951786947221e-05**	0.41	0.944605812952807	4	Fstl3
**6.84732200942901e-05**	-1.93	0.945683642722241	4	Angptl7
**6.89358014396575e-05**	-0.479715851622194	0.95	4	Ddost
**6.92741965071986e-05**	1.79	0.96	4	Pcgf5
**7.27855211601994e-05**	-1.56	1.00	4	Wnt5a
**7.42514467794577e-05**	-1.61	1.00	4	Ccl2
**7.51423696817212e-05**	-1.87	1.00	4	G0s2
**7.78699290360514e-05**	0.54	1.00	4	Fat1
**7.82456753177053e-05**	-0.369465550304395	1.00	4	Jmjd1c
**8.16428007697519e-05**	-0.271549567966273	1.00	4	Ifitm7
**8.61987050316765e-05**	-0.94	1.00	4	Dclk1
**8.62491867209241e-05**	-1.32	1.00	4	Dtx4
**8.68730878170185e-05**	-0.842353681772653	1.00	4	Mmp14
**8.87288996244983e-05**	-0.835479847539122	1.00	4	Leprel2
**8.90266238213087e-05**	0.533670408864805	1.00	4	Smoc1
**8.91078333481117e-05**	0.731915043674709	1.00	4	Zfp568
**8.94759857403262e-05**	0.319187346772464	1.00	4	Nxn
**8.97579588481054e-05**	1.03	1.00	4	Rab11fip3
**9.01789207367049e-05**	-0.829957806393632	1.00	4	Sec11a
**9.1034195272428e-05**	0.307858917365906	1.00	4	2610002J02F
**9.5462457506206e-05**	-0.602302315458757	1.00	4	Meg3
**9.55624209837405e-05**	0.605265008338082	1.00	4	Anxa6
**9.74295579878537e-05**	0.332542306010465	1.00	4	Fam118a

**9.85164119025991e-05**	0.449121429772066	1.00	4	Iqgap1
**0.000101532414700002**	-0.949203697147764	1.00	4	Abca9
**0.000102513530736633**	0.28	1.00	4	Myo1e
**0.000103011988577244**	-0.919460306635434	1.00	4	Fxyd5
**0.000103964180475416**	0.662958784220237	1.00	4	Ptprj
**0.000105036469171709**	-0.263966627813129	1.00	4	H2-K1
**0.00011144725976723**	-0.475417744394354	1.00	4	Capg
**0.000112892960396046**	-1.35	1.00	4	Fgf7
**0.00011359746790723**	-1.31	1.00	4	Mdk
**0.000113798529786202**	0.547080415406028	1.00	4	Pitrm1
**0.000114613694530039**	0.351193525413342	1.00	4	Mycbp
**0.000117182634544781**	-0.859574690251989	1.00	4	Cnot6
**0.000117433634799883**	0.319028302146281	1.00	4	Zfhx3
**0.000119294637224328**	0.536348446704501	1.00	4	Nrbp1
**0.000121514135854873**	1.10	1.00	4	Mepce
**0.00012552491545887**	-0.260139194736343	1.00	4	Rps19-ps3
**0.000125906987343974**	0.646076204916595	1.00	4	Ppp1r12c
**0.000126246636238271**	-0.988114119902842	1.00	4	Ifnar2
**0.000127692236329367**	-0.257323393773779	1.00	4	Atf4
**0.000130002908566521**	0.429406680516874	1.00	4	Rnf123
**0.00013157149509692**	1.35	1.00	4	Wtip
**0.00013204477598815**	-1.03	1.00	4	Gpx7
**0.000133685465381438**	0.84	1.00	4	Nt5c3b
**0.000135290479661798**	-0.568253203313128	1.00	4	Mmp23
**0.000136616507208821**	-1.50	1.00	4	Meox2
**0.000136695070285685**	0.791304521599109	1.00	4	Kpna3
**0.00014139697713998**	0.297306985046937	1.00	4	Efnb2
**0.000148754034703882**	-0.54	1.00	4	Atp6ap2
**0.000149368162587245**	1.53	1.00	4	Tmem110
**0.00015385155462071**	-0.944963123038428	1.00	4	Ece1
**0.000154433544445652**	0.376956050549811	1.00	4	Kalrn
**0.000158415224791823**	0.706940502692281	1.00	4	Adarb1
**0.000160585259979881**	1.22	1.00	4	Dtna
**0.000162332784958479**	-1.32	1.00	4	Naalad2
**0.00016561614129977**	-0.897882460680095	1.00	4	Irf1
**0.000170508804520823**	0.525669516645829	1.00	4	Agl
**0.000173793175070729**	-1.38	1.00	4	Cd55
**0.000175887961866542**	-0.533151118701961	1.00	4	Ier3ip1
**0.00018169727663968**	0.479749656472131	1.00	4	Tmem57
**0.000185063774300052**	0.413286386716996	1.00	4	Strbp
**0.000185420629934079**	0.311606726925886	1.00	4	Gps2
**0.000185944374019162**	0.381395800539162	1.00	4	Itgb1
**0.0001876599420434**	-0.443908436207105	1.00	4	Gstp1
**0.000188095355545193**	0.450921567057702	1.00	4	Fxr2
**0.000190693568013717**	-0.490043278243911	1.00	4	Atp5o
**0.000192300536021579**	-0.891007209878442	1.00	4	Mfap4
**0.00019360274741031**	-0.70	1.00	4	Gm15421
**0.000197756520906439**	0.298265118953869	1.00	4	Fgd4
**0.00019824994735367**	0.552846312061479	1.00	4	Itpr1
**0.000198364602100318**	-1.73	1.00	4	Hhip
**0.00020200470870882**	-1.20	1.00	4	Phlda1
**0.00020331461100375**	-0.492118957506069	1.00	4	Fndc3b
**0.000205144265027536**	0.605236298237565	1.00	4	Cpeb3
**0.000211872784730092**	-0.41	1.00	4	Spcs1
**0.000220713113127218**	-1.03	1.00	4	Plekha6
**0.000223954372944476**	0.467903256134842	1.00	4	Fbxo30

**0.000227444179282866**	0.29	1.00	4	Mbnl1
**0.000232392968997083**	0.425463649680552	1.00	4	Ctps
**0.000235583875999545**	-1.23	1.00	4	Fmo1
**0.00023943230815133**	-1.26	1.00	4	Nsg1
**0.00023944322760655**	0.638503341139053	1.00	4	Ncaph2
**0.000239447514990533**	0.362294455444524	1.00	4	Lynx1
**0.000243320172643975**	-1.45	1.00	4	Lhfpl2
**0.000243420911834719**	1.43	1.00	4	Grb14
**0.000244265757341213**	-0.543590322019146	1.00	4	Npdc1
**0.000244723271478718**	-0.51	1.00	4	Hnrnph1
**0.000247698616246379**	1.09	1.00	4	Isyna1
**0.000247858216356125**	-0.285141177073588	1.00	4	Laptm4a
**0.000255343562483138**	-0.464250812109275	1.00	4	Rps16
**0.000262830196993611**	0.320832111923249	1.00	4	Gtf2a1
**0.000263209864147886**	0.42	1.00	4	Hiat1
**0.000266650413929794**	-1.34	1.00	4	Ahsg
**0.000272814460545465**	-0.779701832261891	1.00	4	Snrpg
**0.00027329609356765**	-1.12	1.00	4	Tnfaip3
**0.000274154869934106**	0.298581036557752	1.00	4	Spag7
**0.000274563666108148**	-0.99	1.00	4	Gstm5
**0.000282036686856997**	0.513135945043329	1.00	4	Sertad1
**0.000284012938110128**	-1.48	1.00	4	AI507597
**0.000284954424005827**	0.483796951378071	1.00	4	Mcur1
**0.000290735632143319**	0.558617428015089	1.00	4	Ttc28
**0.000291598007944069**	0.673264092937368	1.00	4	Cd59a
**0.000295973515135775**	-1.31	1.00	4	Ntrk2
**0.000296827919949217**	0.502111797081466	1.00	4	Ano1
**0.000299647082654579**	1.33	1.00	4	Ntrk3
**0.000302854209885522**	-0.311023753186031	1.00	4	Nav1
**0.000303628980174081**	-0.73	1.00	4	Rpl22l1
**0.000306151107762363**	-1.50	1.00	4	C2
**0.000307453830969087**	-0.325999570742121	1.00	4	Hdlbp
**0.00031319850698707**	1.73	1.00	4	B3galt1
**0.000317524704827032**	-1.35	1.00	4	Mfap2
**0.000320030227867775**	-0.808042987334969	1.00	4	Glul
**0.000339560785688861**	-1.32	1.00	4	Ssh2
**0.00034297504264959**	0.574303870515885	1.00	4	Btbd7
**0.000361147281648453**	-0.842188719162099	1.00	4	Os9
**0.000362164844009965**	-1.01	1.00	4	0610031J06F
**0.00037375998716639**	0.843764016347036	1.00	4	Ttll1
**0.000374386783809994**	1.24	1.00	4	Phf2
**0.000379668456014121**	0.728609176755926	1.00	4	Fam214b
**0.000380265383077398**	-0.602343205855326	1.00	4	Tgoln1
**0.000381810932228614**	0.427781735498756	1.00	4	Nek9
**0.000385772266019229**	0.465478370854387	1.00	4	Chd6
**0.000389137677049604**	-1.20	1.00	4	Ccl19
**0.000390460598446453**	0.750615566194942	1.00	4	Sept9
**0.000393647698550763**	0.337897330813706	1.00	4	Cd24a
**0.000393864211375521**	1.90	1.00	4	3830406C13I
**0.000411767106556155**	-1.01	1.00	4	Egfr
**0.000415472737641666**	0.633021512869274	1.00	4	Tgfbr1
**0.000416967426892532**	0.650006580939929	1.00	4	Specc1
**0.000417220186754572**	-1.41	1.00	4	Bdh2
**0.000417535714863743**	0.60	1.00	4	Serpinb6b
**0.000425595519416563**	0.595584979816711	1.00	4	Pkm
**0.000427761595543843**	-1.41	1.00	4	Myo6

**0.000428553798148918**	-1.06	1.00	4	Slc16a2
**0.00043096178279973**	-0.746176354673011	1.00	4	Cyba
**0.000436249104776817**	-0.865101230853741	1.00	4	Sgce
**0.000440376273245801**	0.544184353409264	1.00	4	Qsox1
**0.000442767450805042**	-0.83	1.00	4	Raph1
**0.000454765404040143**	-0.441986049753087	1.00	4	Fkbp9
**0.000457444268085309**	0.34	1.00	4	Pygm
**0.000459919548394826**	0.59	1.00	4	Prdm6
**0.000470422635051169**	0.48	1.00	4	Fermt2
**0.000480898288339646**	-0.336370629236478	1.00	4	Tmem256
**0.000488265751358919**	-0.365600201827359	1.00	4	Lrpap1
**0.000489102559770715**	-1.31	1.00	4	Dapk1
**0.000489988730389095**	1.77	1.00	4	D16Ertd472e
**0.000495425811917435**	-0.423226931862714	1.00	4	Rpl39
**0.000500827021139822**	-1.24	1.00	4	Fndc1
**0.000508658698807176**	-0.285463658715521	1.00	4	Rps15a
**0.000521343528238153**	0.621713750231157	1.00	4	Ppp1r12b
**0.000525780285586845**	1.07	1.00	4	Myo9b
**0.000537834223188166**	-1.23	1.00	4	Sfrp4
**0.000539615382421339**	-0.77	1.00	4	Tgif1
**0.000544933859666883**	-1.22	1.00	4	Lamb1
**0.000547737309085078**	-0.659851974858136	1.00	4	Colec12
**0.000548092528866936**	0.51	1.00	4	Eef2k
**0.000551314505287254**	1.05	1.00	4	Tead3
**0.000553704278766567**	-0.633910118643541	1.00	4	Jak1
**0.000555977646779584**	0.980899265497315	1.00	4	Mfhas1
**0.00056242659464458**	1.48	1.00	4	Mmd
**0.000579387793076602**	1.18	1.00	4	Plekhg3
**0.000580084592255123**	0.314449456155312	1.00	4	Ddb1
**0.000586029170047794**	-1.13	1.00	4	Tmem176a
**0.000598790527679177**	-0.527956872267677	1.00	4	Islr
**0.000608811472003728**	-0.868088630056277	1.00	4	Tgfb3
**0.000616131221492115**	-0.337743326258913	1.00	4	Ndufa13
**0.000619688292903589**	-0.444698544724495	1.00	4	Ddr2
**0.000623348330613196**	0.542433885871775	1.00	4	Ldlrad4
**0.000626369509482458**	-1.14	1.00	4	Rasa3
**0.000637005045757464**	-0.446461707284787	1.00	4	Acap2
**0.0006417499388607**	0.443154883088547	1.00	4	Dcaf8
**0.00064712404382841**	-1.26	1.00	4	Ildr2
**0.000672268032192542**	-0.85	1.00	4	Tiparp
**0.000684448829752962**	-0.963571438426246	1.00	4	Birc3
**0.000697784068466049**	-1.29	1.00	4	Ccbe1
**0.000700671734717499**	0.335124753078485	1.00	4	Cib1
**0.000703162707597119**	-0.351135804450288	1.00	4	Anxa1
**0.000710003280243759**	1.09	1.00	4	Akt3
**0.000717332000161755**	-0.991952282143925	1.00	4	Ndufa4l2
**0.000727291697789526**	-0.740333081195051	1.00	4	Cebpg
**0.000736910940753187**	-0.885727706073277	1.00	4	Ggh
**0.00075308168981209**	-0.820273147452022	1.00	4	Rhou
**0.000763103058981471**	0.445908908231994	1.00	4	Ubxn2a
**0.00076764077286114**	-1.36	1.00	4	Abca8b
**0.000769254638109264**	0.566508599652251	1.00	4	Stx17
**0.000772049816536876**	0.36	1.00	4	Bnip2
**0.00078383729596355**	-0.266445866412367	1.00	4	Mrfap1
**0.00080418473326216**	0.782386105008361	1.00	4	Pdgfc
**0.000805618145884741**	-1.51	1.00	4	Deptor

**0.000807955443957472**	0.364251800728288	1.00	4	Tcta
**0.000810467154330712**	-0.856309647032218	1.00	4	Sox9
**0.000812574129488468**	1.52	1.00	4	Art3
**0.000820692479766307**	0.313006034684092	1.00	4	Igf1r
**0.000821972114172655**	0.37	1.00	4	Kif13a
**0.000823638021661516**	0.323961263559911	1.00	4	Hcfc1r1
**0.000830566056278977**	-1.65	1.00	4	S1pr3
**0.000843051964934839**	0.823225859470545	1.00	4	Nat6
**0.000847005748841771**	0.661921143876243	1.00	4	Atrip
**0.000848271570800825**	-1.28	1.00	4	Ssc5d
**0.000852106262114037**	-1.48	1.00	4	Itm2a
**0.000853910668059401**	-1.71	1.00	4	Has1
**0.000867165619674816**	0.700626353853318	1.00	4	Pip5k1c
**0.000867444802013965**	0.760716899473086	1.00	4	Coro1c
**0.00087824178202755**	0.407068874715915	1.00	4	Tbxa2r
**0.000878849491308035**	-0.715771381641815	1.00	4	Ptgfrn
**0.000879129110799906**	0.940840918579929	1.00	4	Itga3
**0.000881689966198416**	0.802928878521245	1.00	4	Prkar2a
**0.000888376591580592**	-1.24	1.00	4	Gbp7
**0.000922000436563401**	-0.663371696618645	1.00	4	Nop58
**0.000924488341188512**	0.933677422683135	1.00	4	Id4
**0.000925581396885058**	-1.53	1.00	4	Ebf2
**0.000953287128703449**	-0.874159789052259	1.00	4	Sdc3
**0.000955555106151143**	-1.24	1.00	4	Podn
**0.000958954162584348**	1.03	1.00	4	Cog4
**0.000963862061847047**	-1.33	1.00	4	Prrx2
**0.000969438835282092**	-1.18	1.00	4	Man1c1
**0.000979684594510729**	-0.952763924187924	1.00	4	Cdon
**0.000991737977377919**	0.498259083484051	1.00	4	2010111I01R
**0.00103656818337363**	-0.97	1.00	4	3632451O06
**0.00106265254449455**	0.740909361952431	1.00	4	Smarca4
**0.00106528573252416**	0.923127504213131	1.00	4	Rnf220
**0.00106859447049662**	0.544196373328674	1.00	4	Ptpn21
**0.00107124277466922**	0.85	1.00	4	Tesk1
**0.00109026399637354**	-1.46	1.00	4	Gas7
**0.00110521509251071**	-0.800534519017298	1.00	4	Prss12
**0.00110804125330566**	-1.22	1.00	4	Cpt1a
**0.00110868492832495**	0.418956907548634	1.00	4	Cped1
**0.00110942239131243**	0.650266850754438	1.00	4	S100a4
**0.00112240057441214**	-1.09	1.00	4	Mxra7
**0.00112532966671257**	0.634279374670345	1.00	4	Fam210a
**0.00113022134261844**	-0.912478777699848	1.00	4	Smim3
**0.00113414018732639**	0.926382059959057	1.00	4	Sema5a
**0.00115098824940552**	-0.40	1.00	4	Vwf
**0.00115888349287999**	0.628563074337806	1.00	4	Flnc
**0.00117145514873193**	0.955930388129416	1.00	4	Eml1
**0.00118583667746163**	-0.409449674454127	1.00	4	Prdx4
**0.00118819427827307**	-0.541533190976133	1.00	4	Sfrp1
**0.0012051077233619**	-0.743090112470741	1.00	4	Fam63b
**0.00121432517672178**	0.679131785234937	1.00	4	Polr2b
**0.00122265382108219**	-1.40	1.00	4	C1qtnf7
**0.0012261314058896**	1.08	1.00	4	Cap2
**0.00126928327955428**	-0.917416510505211	1.00	4	Gba
**0.00129110168834528**	-1.03	1.00	4	Lrp6
**0.00130317453775671**	0.53	1.00	4	Tsc22d1
**0.00131072142439357**	-0.916483651716559	1.00	4	Gpr64

**0.00131302417972457**	-0.693103829524933	1.00	4	Cdkn1a
**0.00131447296390082**	0.674035066249877	1.00	4	Myo1c
**0.00131949144014641**	0.275643208566876	1.00	4	Ppp1cb
**0.00132582177549558**	0.303584655015984	1.00	4	Sgk1
**0.00133080114097669**	0.521727230923842	1.00	4	Agrn
**0.00134125419028482**	-1.04	1.00	4	Itih5
**0.00134447553160267**	0.843412124764751	1.00	4	Serf2
**0.0013609664465522**	-0.59	1.00	4	Dpysl2
**0.0013817541933219**	-0.895967782730261	1.00	4	Cpne8
**0.00139023187153929**	0.887484732625073	1.00	4	Mrps15
**0.00139877505611816**	-1.04	1.00	4	Gpr153
**0.00140980849205875**	-1.41	1.00	4	Vit
**0.00142944719774945**	-0.419968536188333	1.00	4	Gtf2h5
**0.00144975373927673**	-0.838649143880268	1.00	4	Gpc3
**0.00145703648876414**	0.721349973912433	1.00	4	Asb2
**0.00146765766708146**	-0.597203939724225	1.00	4	Map4k4
**0.00148940848482611**	-1.20	1.00	4	Angptl1
**0.00148970749762148**	-0.49	1.00	4	Ntn1
**0.0015053395064277**	-0.445505061754337	1.00	4	Scarb2
**0.00151314693359621**	-0.439061014585783	1.00	4	Olfml2b
**0.00153677444233786**	-1.04	1.00	4	Hand2
**0.00155293818337302**	0.535809296649477	1.00	4	Prkag2
**0.00155810130341445**	-1.02	1.00	4	Snrpa1
**0.00160340965065291**	0.297943070176572	1.00	4	Sipa1l1
**0.00161212993498553**	-0.253204841508514	1.00	4	Ost4
**0.0016164683363373**	-1.06	1.00	4	Gprc5b
**0.00162335857358158**	-0.755855749878476	1.00	4	Cnpy2
**0.00163096079956787**	0.847645059429217	1.00	4	Pomgnt1
**0.00163719847519831**	-0.647250613043192	1.00	4	Tmcc1
**0.00164196240172296**	1.67	1.00	4	Msra
**0.00167842484961704**	-0.545417115169243	1.00	4	Atp5l
**0.00168857319528906**	-0.630343297788527	1.00	4	Prrx1
**0.00171030181364665**	0.597591118966363	1.00	4	Arl4a
**0.00172658498230585**	-1.15	1.00	4	Epyc
**0.00172763774977438**	-1.15	1.00	4	Trf
**0.00173623115330723**	-0.900177430116437	1.00	4	Papss2
**0.00175851383825431**	0.414549194117353	1.00	4	Lrrc58
**0.00177145448774134**	0.901582543874276	1.00	4	Ttc39b
**0.00178670580303811**	1.02	1.00	4	Gnb4
**0.00180169421855208**	-1.32	1.00	4	Asap3
**0.00180325604840175**	0.390836580753977	1.00	4	Olfr1033
**0.00180487035709531**	1.28	1.00	4	Sgca
**0.00180932897216656**	-0.70	1.00	4	Pgs1
**0.00181185335878988**	0.59	1.00	4	Camk2d
**0.00181471932814099**	-1.54	1.00	4	Lsamp
**0.00181886610489781**	-1.04	1.00	4	Fmnl2
**0.00182690030098313**	1.01	1.00	4	Adcy9
**0.00184504780358099**	0.938758443680062	1.00	4	Naa50
**0.00185553567519397**	-1.31	1.00	4	Enc1
**0.00187472162550724**	0.38	1.00	4	Ccdc107
**0.00189879829474481**	-0.846720872284408	1.00	4	Tgfbr3
**0.00190753398669163**	-0.34	1.00	4	Xbp1
**0.00191264702891143**	-1.41	1.00	4	Cxcl14
**0.00191514059326378**	0.306166568159997	1.00	4	Ankrd1
**0.00191633603173986**	0.537766066104562	1.00	4	Rasl11b
**0.00193602988248872**	0.823337152434104	1.00	4	Crbn

**0.0019474024426948**	0.498190133106721	1.00	4	Kpna1
**0.00196160777198306**	1.37	1.00	4	Fh1
**0.00196502696946731**	-0.622632157601371	1.00	4	Tcn2
**0.00199495311483849**	-1.29	1.00	4	Emilin2
**0.00199841518820657**	-0.253236705766776	1.00	4	Ppp1r15a
**0.00200242243527483**	-0.418625432387099	1.00	4	Rap1b
**0.00201224197968555**	0.435967256728364	1.00	4	Itga1
**0.00202377162309437**	-0.620893191506111	1.00	4	Nbl1
**0.00202993841221719**	0.675379697780063	1.00	4	1810055G02I
**0.00208456001152249**	0.287064035512534	1.00	4	Pgpep1
**0.00210578364137082**	-0.67	1.00	4	Il1r1
**0.00211621347948123**	0.72	1.00	4	Mmrn2
**0.00213004087022175**	-1.27	1.00	4	Sorcs2
**0.00213912064322015**	-1.12	1.00	4	Tbx20
**0.00216059844703346**	-0.705126468037251	1.00	4	Baiap2
**0.00217630751723501**	0.424861768251708	1.00	4	Atp1a2
**0.00218298177094331**	-1.25	1.00	4	Fst
**0.00218563342407824**	0.372678211721871	1.00	4	Dnajc15
**0.00219126693530124**	0.625671051867559	1.00	4	Cog2
**0.00219280103587394**	-1.63	1.00	4	Slc10a6
**0.00219440956455066**	0.544556946147197	1.00	4	Rab3gap2
**0.00220549028644433**	-1.27	1.00	4	1700025G04I
**0.00221458293170824**	-0.261969658082235	1.00	4	Rpl10
**0.00223003710511183**	1.02	1.00	4	Abcc1
**0.00223949394512843**	-1.25	1.00	4	F3
**0.00225094791567305**	1.23	1.00	4	Mical3
**0.00226894287718573**	-0.546430553386434	1.00	4	Scd2
**0.00227950142422927**	-0.526140385791726	1.00	4	Nfe2l2
**0.00228750335307226**	-1.07	1.00	4	Tshz2
**0.00230832682300186**	0.38	1.00	4	Enpp4
**0.00235329604863425**	-1.07	1.00	4	Enpp3
**0.00242112843411184**	-0.545670559377153	1.00	4	Gpx8
**0.00243586576077139**	0.548453057323821	1.00	4	Synpo
**0.00246997962925173**	0.633534171839021	1.00	4	Atp8b1
**0.0024708015699309**	0.59	1.00	4	Zbtb7a
**0.00249365032435185**	1.01	1.00	4	Actr1a
**0.00249779185256419**	0.983178339425644	1.00	4	Sharpin
**0.00252754445538096**	-1.24	1.00	4	Farp1
**0.00255615130895988**	0.346759090115863	1.00	4	Sypl
**0.0025878454965414**	-0.453314575104899	1.00	4	Mllt4
**0.00259198766989997**	-0.619424493094005	1.00	4	Erp44
**0.00259275297125174**	0.414671266233594	1.00	4	Anln
**0.00265155153413543**	-0.798405929787298	1.00	4	Birc2
**0.00266320025405961**	1.85	1.00	4	D5Ertd579e
**0.00266347814368588**	0.860536243548583	1.00	4	Spsb1
**0.00266482962499751**	0.427547406059455	1.00	4	Rexo2
**0.00267030773277569**	-0.641584034964943	1.00	4	Zfp655
**0.00268371146521653**	0.74	1.00	4	Mrpl35
**0.00269042314605612**	0.761428687976351	1.00	4	Lancl3
**0.002701378414483**	-0.557955909036941	1.00	4	Ablim1
**0.00270210173280562**	1.41	1.00	4	Nit1
**0.00271438472030718**	0.732254094105023	1.00	4	Mknk2
**0.00272002870619326**	-0.445975466661575	1.00	4	Kmt2e
**0.00275021397314278**	-0.58	1.00	4	R3hcc1l
**0.0027581502237369**	0.85	1.00	4	Fert2
**0.00278165977280244**	0.480429429444753	1.00	4	Unc45a

**0.00282417929987912**	1.10	1.00	4	Heyl
**0.00282986723477848**	-0.580049682399966	1.00	4	Ikbip
**0.00284200521637142**	0.835647473842175	1.00	4	Svil
**0.00290728992790682**	0.429595508021999	1.00	4	Ext1
**0.00291017738683793**	-0.434984403398001	1.00	4	Ctsh
**0.00292845147851381**	-1.03	1.00	4	Adcy7
**0.00292897467448615**	-0.358350217641884	1.00	4	Hivep2
**0.00293588839022681**	0.38	1.00	4	Zcchc14
**0.00294100183750689**	-0.791388481173129	1.00	4	Col4a3
**0.00294987441488182**	-1.06	1.00	4	5031426D15I
**0.00299368575016378**	1.30	1.00	4	2310035C23I
**0.00300114346751092**	0.382375948214047	1.00	4	Snx17
**0.00300122979791172**	-0.835001918120372	1.00	4	Steap3
**0.00301257578700807**	1.20	1.00	4	Ier5l
**0.00302202299334991**	0.346937768660874	1.00	4	Man2c1
**0.00302964439462712**	0.379793288031535	1.00	4	Deb1
**0.00304938226120181**	0.442396400849369	1.00	4	B3galnt2
**0.00314888994479481**	-0.615079224347909	1.00	4	Tnfrsf1a
**0.00315546563312137**	0.601266064570725	1.00	4	Atp1b2
**0.00319131744338452**	-1.05	1.00	4	Ifit3
**0.0032106432824661**	-1.01	1.00	4	Prickle1
**0.00321232464390976**	-1.15	1.00	4	Epha7
**0.00326535096649446**	0.76	1.00	4	Ppp2r5a
**0.0033282296687909**	0.390136890016964	1.00	4	Arl6ip5
**0.00334495719475853**	-1.04	1.00	4	Kcnj8
**0.00339469154638773**	0.63	1.00	4	Trappc12
**0.00355125054294182**	-0.83	1.00	4	Man2b1
**0.00355216568105134**	-0.876938131686571	1.00	4	Tsc22d3
**0.00357467468447832**	-1.38	1.00	4	Gfra2
**0.00358725771716273**	-1.16	1.00	4	Psd3
**0.00359327252699673**	0.324354176969048	1.00	4	Gjc1
**0.00364585951852795**	0.754741057676547	1.00	4	5430417L22I
**0.00369777592183949**	-1.08	1.00	4	Tmem9b
**0.00369838421420916**	-1.39	1.00	4	Penk
**0.00370954556168636**	-1.43	1.00	4	AI607873
**0.00374363404016115**	0.295035402049728	1.00	4	Rras
**0.00379337551158991**	-1.29	1.00	4	Shisa3
**0.00379499978640907**	-0.486215120630727	1.00	4	Tceb1
**0.00380116885362658**	0.464898416739376	1.00	4	Ncoa6
**0.00380333784753061**	0.354161424780976	1.00	4	Dek
**0.0038161212373865**	0.709420355172064	1.00	4	Tspyl1
**0.00381894581824661**	-0.533072238116852	1.00	4	Fosl2
**0.00395708704099134**	0.387150766031004	1.00	4	Uhrf1bp1l
**0.00399956762902981**	1.27	1.00	4	Klhdc3
**0.00400893023628992**	0.481350965649811	1.00	4	Tom1l2
**0.00403690870518463**	0.295419249610771	1.00	4	Ifngr1
**0.00405025364498041**	-0.614370270326295	1.00	4	Lcn2
**0.00406594675910825**	-0.265741617288857	1.00	4	Rpl37a
**0.00408869066006344**	-0.636006997363156	1.00	4	Scand1
**0.00413213577950815**	-0.677636136848656	1.00	4	Anapc13
**0.00414195128665952**	0.369027135975851	1.00	4	Vim
**0.00421383179954882**	-1.14	1.00	4	Glb1
**0.00422348302023371**	-0.924392378071323	1.00	4	Pmm1
**0.00436578374149975**	-0.956507730389018	1.00	4	Trps1
**0.0044211058156843**	0.361910855329336	1.00	4	Cyld
**0.0044261534223637**	-0.632951821580267	1.00	4	Atraid

**0.00443561908622447**	0.417974208477405	1.00	4	1110008P14F
**0.00450194767129649**	-0.258048849277348	1.00	4	Scara3
**0.00452681929004959**	-1.45	1.00	4	Gpr39
**0.00457433215823948**	-0.88	1.00	4	Lifr
**0.00465952940514591**	-0.668548333958272	1.00	4	Lipa
**0.00467236718340617**	-0.925602405408128	1.00	4	Ptp4a1
**0.00467605674499723**	1.05	1.00	4	Tspan9
**0.00469842967392776**	0.271622431434436	1.00	4	Gpr107
**0.00478311598267841**	-0.380194343192788	1.00	4	Manf
**0.00478454578344127**	0.736429167112979	1.00	4	Stbd1
**0.00479906024756175**	0.38	1.00	4	Cyhr1
**0.00487752361184612**	0.345956563730263	1.00	4	Agtpbp1
**0.0049117820611651**	-1.16	1.00	4	Rab43
**0.00491468243610285**	-0.820572851196315	1.00	4	Nr2f2
**0.00500462609842632**	0.596926651508814	1.00	4	Thoc6
**0.00500862797898616**	0.81	1.00	4	Ifngr2
**0.00507032145689696**	0.260309193201445	1.00	4	Col4a1
**0.00513605447251966**	-0.251125540440833	1.00	4	Comt
**0.00514245835143823**	0.551672360248202	1.00	4	Rabgap1
**0.00516414409673549**	0.320621498225681	1.00	4	Kansl1
**0.00520240741245595**	0.714584737275147	1.00	4	Rrp1
**0.00521527589637339**	0.285756161991626	1.00	4	Ola1
**0.00521563503906367**	0.640018190199434	1.00	4	Ano10
**0.00526054812272761**	-0.532839209959722	1.00	4	Tnfsf12
**0.00527279912296571**	0.377874908351087	1.00	4	Bmpr1a
**0.00534062749492915**	0.348676662701797	1.00	4	Bbx
**0.00534195881522697**	0.46	1.00	4	Large
**0.00535900884632799**	1.06	1.00	4	Lmf2
**0.00537006191793184**	-0.416028815099571	1.00	4	Fbn1
**0.00538311358039442**	-1.24	1.00	4	Runx1
**0.00540107219692746**	0.562320218246417	1.00	4	Cd200
**0.00548901638961558**	-1.43	1.00	4	Sult5a1
**0.00551305235773896**	-0.307972767099181	1.00	4	Gm12060
**0.0055228492035203**	-1.21	1.00	4	Psmb8
**0.00553998475461064**	-0.300949615560501	1.00	4	Ier3
**0.00559792102185082**	-1.08	1.00	4	Arl5b
**0.00563899528943216**	-0.47	1.00	4	Atp6v1g1
**0.00565372749665921**	0.383130982183842	1.00	4	Nsmf
**0.00574410165583395**	-1.39	1.00	4	Bmper
**0.00577273201151819**	0.424749932234175	1.00	4	Slc6a8
**0.00581848406999882**	-0.936612028519224	1.00	4	Nop16
**0.00582590240839138**	-0.591201189371619	1.00	4	Tmod3
**0.0058335687526492**	-0.73	1.00	4	Pcsk6
**0.00593176411779494**	0.268094939176476	1.00	4	Ddx47
**0.00593628385449147**	-0.902031100570395	1.00	4	Pikfyve
**0.00593962015541247**	-1.46	1.00	4	Ccl11
**0.00604336547450977**	0.269567328277122	1.00	4	Camsap1
**0.00606838776236792**	0.499910485321838	1.00	4	Akap9
**0.00610541005222788**	-1.11	1.00	4	Cilp
**0.00612100733744203**	0.80	1.00	4	Mrpl50
**0.00613645359835919**	-1.00	1.00	4	Meis2
**0.00614321567049284**	0.975141875799995	1.00	4	2210407C18F
**0.00615072003256369**	0.624477951127709	1.00	4	Xrcc5
**0.00618517425113064**	-0.441247287089573	1.00	4	Dap
**0.00626977670859391**	-0.395637235160244	1.00	4	Tmed5
**0.00628496263781494**	0.634097250161599	1.00	4	Mcm3ap

**0.00633582909529083**	-1.00	1.00	4	Mecom
**0.00634711645329504**	-0.925183851740528	1.00	4	Nin
**0.00638806172795615**	-0.842889872279868	1.00	4	Ncstn
**0.0064050170644753**	-1.15	1.00	4	Tsta3
**0.00641077777467877**	0.36	1.00	4	Ezh1
**0.00647238957905377**	-0.390804201060765	1.00	4	Hsbp1
**0.00654439020152501**	0.416731261849763	1.00	4	Sertad4
**0.00655612158943509**	-0.778280678702123	1.00	4	Smarcd2
**0.00658793265290894**	0.573311190851034	1.00	4	Mak16
**0.00658996625695004**	-0.848363652658617	1.00	4	Bin3
**0.00663820339438507**	0.273094797069281	1.00	4	Sepw1
**0.00665261502341763**	0.292496444203505	1.00	4	0610011F06F
**0.00674399437312443**	1.11	1.00	4	Wfdc1
**0.00677457469614957**	-0.287410775651419	1.00	4	Rps7
**0.00678471152646206**	-0.465683488574723	1.00	4	Socs2
**0.00687476283628637**	0.423080577841726	1.00	4	Stip1
**0.00691497563491843**	0.669054255344216	1.00	4	Senp7
**0.00692848698128984**	-1.19	1.00	4	Tenm3
**0.00695243702267588**	-1.18	1.00	4	Mmp19
**0.0069597818518769**	-0.697990642718385	1.00	4	Itpr2
**0.00696386267370573**	0.738792399116364	1.00	4	Rab3a
**0.00697219802399855**	0.738876983855328	1.00	4	Ggta1
**0.00698999315651298**	-1.11	1.00	4	Cxcl16
**0.00704142768797111**	-1.06	1.00	4	Col8a1
**0.00704700477461018**	0.621365391658709	1.00	4	Oxsr1
**0.00711308608476888**	-0.840133224862459	1.00	4	Rbms1
**0.00711420502992747**	0.523294597167388	1.00	4	Sema3c
**0.007118842028847**	-0.669717631677736	1.00	4	Adipor1
**0.00712195688469519**	-0.521837305345246	1.00	4	Kcne4
**0.00718648040529854**	0.31	1.00	4	St3gal2
**0.00721583610878852**	0.368702307060209	1.00	4	Itgb5
**0.00725526426480391**	0.446266329358276	1.00	4	Zfand3
**0.00733228514585653**	-0.573679420306053	1.00	4	Nlgn2
**0.00735403069076289**	-1.18	1.00	4	Olfml1
**0.00737097014554418**	-0.47	1.00	4	Acvrl1
**0.00737202430642289**	-0.646669706349771	1.00	4	Trip12
**0.00737993750816181**	-0.561289568374773	1.00	4	Kdm6b
**0.00741779348945431**	-0.415483967129736	1.00	4	Trim8
**0.0074420402330688**	-0.639072912644401	1.00	4	C1qtnf1
**0.00745528071279114**	-1.08	1.00	4	Rgs10
**0.00745550406191516**	-0.305862783821068	1.00	4	Wnk1
**0.00765377351408786**	0.490224158586941	1.00	4	Setd3
**0.00771873919254945**	-0.476347908144884	1.00	4	Mrpl33
**0.00771980963746069**	0.559720363098917	1.00	4	Wdr6
**0.00772313375337402**	1.55	1.00	4	Cdkn2aip
**0.00772813305789483**	0.619073144481972	1.00	4	Cacna1c
**0.00776894190697166**	-0.482990513581446	1.00	4	Ppic
**0.00781094115206817**	0.714667138458053	1.00	4	Srf
**0.00787834217043466**	0.269135142083224	1.00	4	Zfand6
**0.00788167525926567**	0.625783588775341	1.00	4	Nr1d1
**0.00791353169296566**	-0.490735595848603	1.00	4	Btg2
**0.00793050883435035**	-0.330408390582899	1.00	4	Sat1
**0.00793447212441755**	0.510095089744242	1.00	4	Nrep
**0.0079777000124913**	-0.672691608602193	1.00	4	Prnp
**0.00798139318425136**	0.616225014266267	1.00	4	Zfp651
**0.00800541922156242**	-1.23	1.00	4	Glt8d2

**0.00801515623274856**	0.446657404428614	1.00	4	Zadh2
**0.00807831642829553**	1.32	1.00	4	Arhgap35
**0.00808778459999003**	0.451848846577191	1.00	4	Nptn
**0.00821392127494908**	-1.54	1.00	4	S1pr2
**0.00822774750546314**	-0.887712025750669	1.00	4	Slc25a51
**0.00825177998732625**	-0.365137992675382	1.00	4	Ssr1
**0.00827933326975552**	0.567801142273462	1.00	4	Peli2
**0.00828065449857898**	1.18	1.00	4	Lemd2
**0.00828719523749337**	-1.23	1.00	4	Pcdh18
**0.00832162344600992**	-0.30	1.00	4	Cd93
**0.00837750505741281**	0.536341609020855	1.00	4	Trak1
**0.00845127790877439**	-0.277980223045321	1.00	4	Pbx1
**0.00850892421275142**	0.25	1.00	4	Taf3
**0.00858896529684868**	0.712050375844552	1.00	4	Bcl2l11
**0.00865953151730529**	0.440513215508823	1.00	4	Acadsb
**0.00868071392763843**	-0.344825399580859	1.00	4	Flt3l
**0.00878477099152619**	-0.460989738912679	1.00	4	Prr13
**0.00884065128454337**	-0.321181908221284	1.00	4	BC048507
**0.00886824646270059**	0.792353584189829	1.00	4	Dhrs1
**0.00887909745679617**	-0.973493693112543	1.00	4	Taf10
**0.00889635071544083**	0.476782274378687	1.00	4	Ralgapa2
**0.00891864104685959**	0.499546554755772	1.00	4	Armc1
**0.00893092465791068**	-0.53	1.00	4	Pdgfrb
**0.00899941077161461**	-0.559221649240264	1.00	4	Slc9a6
**0.00901320341421773**	-0.957423553117724	1.00	4	Spry1
**0.0090428662883885**	-0.730976422769389	1.00	4	B4galt5
**0.00908638701105711**	-0.266707390910781	1.00	4	Uqcrb
**0.00908736203553602**	-0.37	1.00	4	Rpl29
**0.00920944497090594**	0.265799233672326	1.00	4	Cdadc1
**0.0092619037496328**	-1.34	1.00	4	Fam43a
**0.00927278349086753**	0.320465948600301	1.00	4	Dst
**0.0092732704506889**	-0.301494965508861	1.00	4	Nadk
**0.00928322634760817**	0.29	1.00	4	Scmh1
**0.00930627450615091**	-0.511749644160183	1.00	4	Ostc
**0.00935760167795579**	0.31	1.00	4	Ypel3
**0.00936513357816392**	0.288526039145099	1.00	4	Fam168a
**0.00936555164242282**	-0.849252895223007	1.00	4	Dpp9
**0.00937749117634814**	-1.24	1.00	4	Fyn
**0.00941105868170973**	-0.645120686785367	1.00	4	Sh3d19
**0.00942980141277212**	0.684618147254383	1.00	4	Traf5
**0.00947043252267258**	0.259838180051511	1.00	4	Stk24
**0.00962197936893925**	-0.401056762912491	1.00	4	Daam1
**0.0096222599420694**	-1.10	1.00	4	Amy1
**0.00972252236958324**	0.491861511214802	1.00	4	Kpna6
**0.00977090224657269**	-1.55	1.00	4	Tspan11
**0.00982682293495174**	0.452326447977725	1.00	4	Cript
**0.00983417512916549**	-0.77	1.00	4	Ptpdc1
**0.00985116860166596**	0.773561102276385	1.00	4	Cdk10
**0.00987889503923835**	0.372995713905729	1.00	4	Wdr1

p_val	avg_logFC	p_val_adj	cluster	gene
3.02079169957185e-26	1.378030376	4.17201541627869e-22	5	Gfpt2
1.11757573555314e-24	0.970703554235397	1.54348384837245e-20	5	C3
2.15426562349249e-24	1.070284821	2.97525625260548e-20	5	C1s1
9.54821268114821e-23	1.185744082	1.31870365339338e-18	5	Mnda
9.65416705289982e-23	1.749243134	1.33333701167599e-18	5	Gpr133
1.43172630582678e-22	1.798123695	1.97735720097736e-18	5	Ccl7
4.0554672899961e-22	0.726797242134409	5.60100587421362e-18	5	Serping1
4.53444944104453e-22	1.330405008	6.26252812302661e-18	5	Tnfaip6
2.30468742258449e-21	0.962327714479351	3.18300379933144e-17	5	C1ra
6.16425758394968e-21	0.903677787030323	8.5134561491929e-17	5	Mt2
1.7729757321161e-20	0.754056614	2.44865678362554e-16	5	Igfbp4
1.33960353271774e-19	0.676371002357964	1.85012643903647e-15	5	Mt1
2.55837011478704e-19	1.082432015	3.53336496553238e-15	5	Clec3b
4.64483553825342e-19	1.14152448	6.4149823618818e-15	5	Cxcl1
1.403484589334E-18	1.036474467	1.93835256632918e-14	5	Scn7a
2.09210622905356e-18	0.700162420225252	2.88940791294587e-14	5	Gsn
5.20748338923433e-18	1.514603257	7.19205530887153e-14	5	Emilin2
1.03603078629061e-17	1.727316143	1.43086211894596e-13	5	AI607873
2.5407321494992e-17	1.571620036	3.50900517167334e-13	5	Ifi204
2.58854641239782e-17	-1.118896751	3.57504145016263e-13	5	Cpe
4.66810418414265e-17	0.615275166683644	6.44711868871941e-13	5	Rarres2
6.09852170518294e-17	1.015733442	8.42266832702815e-13	5	Cfb
6.88964671892308e-17	2.050756427	9.51529108350467e-13	5	Ptx3
1.07110875964424e-16	2.027006974	1.47930830794466e-12	5	Ccl11
1.58576607470866e-16	1.151626433	2.19010152578013e-12	5	Srpx
1.73499289517602e-16	0.689413470969424	2.3961986875276e-12	5	Pcolce
3.11474512176008e-16	1.109055265	4.30177448766284e-12	5	Phlda1
4.90164287112662e-16	1.698631218	6.76965896931297e-12	5	Has1
1.13958448276245e-15	1.045810153	1.57388012914322e-11	5	Scara5
2.48337256017602e-15	-1.08419663	3.4297858428591e-11	5	Tm4sf1
2.55979054147827e-15	1.139147573	3.53532671683564e-11	5	Hk2
3.29117542791942e-15	1.849478751	4.54544238349951e-11	5	Lrrn4cl
4.47458149983146e-15	-1.482024814	6.17984450941723e-11	5	Acta2
5.77619569076079e-15	0.643149464263268	7.97750386850973e-11	5	Ly6a
6.984730288313E-15	1.558711494	9.64661100118909e-11	5	Ifi205
7.2390782301414e-15	1.733551357	9.99789094364829e-11	5	Opcml
7.30407573295344e-15	1.782823609	1.0087658994782e-10	5	Ccl2
8.6299013964636e-15	1.043429861	1.19187568186559e-10	5	Fndc1
1.05435832051376e-14	0.694514172106746	1.45617427646155e-10	5	Dpt
1.07008797261138e-14	1.367555006	1.47789849897357e-10	5	Cd248
1.0771500364126e-14	1.094944917	1.48765191528944e-10	5	Pi16
1.54652512814865e-14	0.865436869441051	2.13590585448611e-10	5	Dpep1
2.33160486417271e-14	-1.390868154	3.22017947790893e-10	5	Fblim1
2.53950129092433e-14	0.744379719760503	3.5073052328956e-10	5	Col14a1
3.64860175803918e-14	0.960030957744028	5.03908388802791e-10	5	Gpx3
7.45476806364543e-14	-1.127654307	1.02957801727007e-09	5	Clu
1.32434486834627e-13	0.438785521159299	1.82905269767303e-09	5	Col1a2
1.40623255441356e-13	0.441806003	1.94214778090057e-09	5	Ifitm3
2.10227417071873e-13	0.906211526036038	2.90345085717964e-09	5	Ly6c1
2.81097487789736e-13	0.867502632390151	3.88223740386404e-09	5	Nid1
2.92075546763392e-13	0.603048155559082	4.03385537634921e-09	5	Rnase4
5.28580768149408e-13	-2.308333141	7.30022898891147e-09	5	Cnn1
6.74593269523634e-13	1.323359793	9.31680764539091e-09	5	Il6
1.14577010397692e-12	-0.997585664566546	1.58242309060253e-08	5	Eln
1.16167162090516e-12	0.407065942879309	1.60438467563212e-08	5	Timp2

**1.28253766117982e-12**	1.202027062	1.77131276385544e-08	5	Pcsk6
**2.24309316432026e-12**	0.922489637	3.09793596924272e-08	5	Csf1
**5.37800004463093e-12**	-1.196126764	7.42755586163978e-08	5	Mylk
**7.05749198109813e-12**	0.754794901818484	9.74710217509463e-08	5	Cxcl12
**8.0623617257286e-12**	1.037616121	1.11349277794038e-07	5	C2
**8.13213559756674e-12**	0.489427926	1.12312924737994e-07	5	Dcn
**1.6346180906175e-11**	-1.789477035	2.25757104495182e-07	5	Lmod1
**1.66047979500943e-11**	-0.756260759272131	2.29328864488752e-07	5	Aplp2
**1.67415277512373e-11**	0.636981992742783	2.31217239772339e-07	5	Cygb
**1.74975561532251e-11**	0.998608128457524	2.41658748032192e-07	5	Dapk1
**1.82092548515548e-11**	0.680908358862279	2.51488018754824e-07	5	Ppap2b
**1.90243534924848e-11**	0.591787029184591	2.62745346084707e-07	5	Serpinf1
**2.76705203302215e-11**	0.597832874230967	3.8215755628069e-07	5	Htra3
**2.82069649438458e-11**	0.522448995599132	3.89566392839454e-07	5	Ecm1
**4.00944928750033e-11**	-1.331582333	5.5374504109667e-07	5	Ccdc3
**4.21196627722398e-11**	-0.840072711568992	5.81714662547404e-07	5	Ctgf
**4.63835281484878e-11**	1.012148556	6.40602907258765e-07	5	Igfbp6
**4.74081185125888e-11**	-1.630522062	6.54753524777364e-07	5	Cdc42ep3
**5.14490990530887e-11**	1.475200469	7.10563507022208e-07	5	Apod
**5.80574845987075e-11**	1.097631425	8.01831919792749e-07	5	Ugdh
**6.55782869981018e-11**	-1.498839559	9.05701721730784e-07	5	Myl9
**7.39043239520648e-11**	0.771589845266108	1.02069261810197e-06	5	C4b
**7.55405986232451e-11**	1.245923763	1.04329120758564e-06	5	Gpc3
**7.96544951328269e-11**	1.613641274	1.10010823227947e-06	5	Plac8
**8.61158818713868e-11**	0.638153688142304	1.18934644452572e-06	5	Add3
**1.08426452460554e-10**	0.531679772813055	1.49747773493272e-06	5	Ccnl1
**1.18997969147931e-10**	1.061901507	1.64348095190207e-06	5	Steap4
**1.42424033780184e-10**	0.549429021904142	1.96701833053812e-06	5	Lrp1
**1.59609168313435e-10**	0.916888811953616	2.20436222357685e-06	5	Igfbp5
**1.68736935453444e-10**	0.482811756280073	2.33042581554752e-06	5	Ctsl
**1.70542637674749e-10**	0.548562794164874	2.35536436892596e-06	5	Fbln1
**1.8988222858688e-10**	0.797334054348088	2.6224634590134e-06	5	C7
**2.06513625703001e-10**	-1.203373071	2.85215968458414e-06	5	Pkp4
**2.12304870800173e-10**	0.381320993840951	2.93214257062118e-06	5	Ftl1
**2.39833803986641e-10**	0.599763295339628	3.3123446668595e-06	5	Entpd2
**2.53833415320018e-10**	0.925120085534022	3.50569329898476e-06	5	Efemp1
**2.73038256536369e-10**	0.550447049077221	3.77093136102379e-06	5	Abca8a
**2.7541664776584e-10**	1.143632147	3.80377932229401e-06	5	Inmt
**2.8072716055377e-10**	0.726570895465319	3.87712281440812e-06	5	Kdm6b
**3.1606588844949e-10**	0.673743823610887	4.36518598537591e-06	5	Anxa1
**4.4136971496535e-10**	-1.313363739	6.09575713338645e-06	5	Itga8
**4.63354876749455e-10**	1.343975594	6.39939420278672e-06	5	Lgi2
**5.58244837078973e-10**	-0.543933567677639	7.70991944489769e-06	5	Lmcd1
**6.00036700134902e-10**	-1.463302441	8.28710686556313e-06	5	Wwp2
**6.46891649736773e-10**	0.884174474605224	8.93422057451457e-06	5	Bcl3
**7.69473810025521e-10**	0.732583468185215	1.06272027902625e-05	5	Marcks
**9.57808656855889e-10**	-1.436147396	1.32282953598367e-05	5	Tagln
**1.14228369504848e-09**	-0.640594144439173	1.57760801123146e-05	5	Cd200
**1.20224997390348e-09**	0.604453366402215	1.6604274389581e-05	5	Smoc2
**1.31079430017278e-09**	1.165716093	1.81033800796863e-05	5	Slc4a4
**1.45368278944804e-09**	0.726351957728122	2.00768130050668e-05	5	Cpxm1
**1.48381482637107e-09**	0.572890960426067	2.04929665670109e-05	5	Cebpd
**1.51141840955207e-09**	0.568328961625748	2.08741996543237e-05	5	Pdgfra
**1.70209656229259e-09**	1.054458193	2.35076556218229e-05	5	Myc
**1.75879473421085e-09**	1.156749547	2.4290714074186e-05	5	Tnxb
**2.1134509605286e-09**	1.019911842	2.91888712158605e-05	5	Akap12

**2.61520843123143e-09**	0.869696049	3.61186436437373e-05	5	Ptgs2
**2.90339036810501e-09**	1.217758184	4.00987243738983e-05	5	Cxcl2
**3.48128457722536e-09**	-0.816139260564755	4.80800212960595e-05	5	Actn4
**3.9313326285636e-09**	-0.892174248	5.42956349330918e-05	5	Pls3
**4.16123808972472e-09**	0.643635062160869	5.74708592571881e-05	5	Bicc1
**4.50510001476437e-09**	-1.219793444	6.22199363039108e-05	5	Pgm5
**4.68728351512443e-09**	1.175326378	6.47360726273835e-05	5	Metrnl
**6.5891078877696e-09**	0.501550953200101	9.1002169037986e-05	5	S100a10
**6.95394667462712e-09**	1.575667323	9.60409575232751e-05	5	Amy1
**7.21989567341329e-09**	0.493301250584481	9.97139791455109e-05	5	Mfap5
**7.54946640768292e-09**	0.828568870309903	0.000104265680556509	5	Slit3
**9.59446054187719e-09**	1.176189501	0.000132509094543866	5	Arhgap24
**9.87781577889906e-09**	0.312934735033868	0.000136422513722375	5	Hspa5
**1.14520725643801e-08**	0.373748633	0.000158164574186654	5	Nfkbia
**1.15323633650272e-08**	0.898988734792969	0.00015927347043439	5	Ces2g
**1.16622783107407e-08**	0.519358252707745	0.00016106772574964	5	Ebf1
**1.23183366223947e-08**	0.701829022212397	0.000170128547091893	5	Slfn5
**1.45419321808071e-08**	-1.546337722	0.000200838625349127	5	Ncam1
**1.46670120095915e-08**	0.421871831549179	0.000202566102864469	5	Slc43a3
**1.57340349741816e-08**	0.865445346045046	0.000217302757028422	5	Pim1
**1.67859379317581e-08**	0.965387980970251	0.000231830588775512	5	Ildr2
**1.71964516797567e-08**	1.203582492	0.00023750019414912	5	Adm
**1.99101902854533e-08**	-1.64619931	0.000274979638032396	5	Tpm2
**2.10094907448356e-08**	0.681884483422782	0.000290162076676924	5	Steap3
**2.12602480778606e-08**	-1.076780538	0.000293625286203333	5	Smoc1
**2.24795208214158e-08**	0.448804145793231	0.000310464662064573	5	Fosl2
**2.38258650791266e-08**	-1.138685371	0.000329059022607818	5	Mcam
**2.49467595732531e-08**	-0.965209827181334	0.000344539696466198	5	Actn1
**2.52491715067625e-08**	-0.460395020601318	0.000348716307679897	5	Itgb1
**3.0240750081855e-08**	-1.171958652	0.000417654999380499	5	Susd5
**3.19983168842088e-08**	-1.111243142	0.000441928754487808	5	Csrp1
**3.2073422193279e-08**	0.999148209	0.000442966033911376	5	Tnfaip2
**3.93420975127185e-08**	-0.769861223916434	0.000543353708748155	5	Dmd
**3.97757021536508e-08**	-0.891734821480162	0.000549342222444072	5	Myh11
**3.99787700137618e-08**	0.877944743321992	0.000552146792660064	5	S1pr3
**5.91797265717315e-08**	-0.884367049115752	0.000817331203682184	5	Tpm1
**6.13484224654297e-08**	0.443797014275252	0.00084728306267005	5	Cd34
**6.24276445039179e-08**	-1.868600234	0.00086218819824361	5	Entpd1
**6.90084652387556e-08**	0.394269238574745	0.000953075913412453	5	Adamts1
**6.99963328310618e-08**	0.751834362024945	0.000966719352729794	5	Apbb1ip
**8.00461594420176e-08**	0.774556795816792	0.00110551750805371	5	Trib1
**9.36475600029964e-08**	0.548304089234397	0.00129336645120138	5	Abcc9
**9.92384407814314e-08**	-1.296464446	0.00137058210563235	5	Slmap
**1.14979090710644e-07**	-0.458675385713082	0.0015879762218047	5	Fhl1
**1.16959216029917e-07**	0.749961151448489	0.00161532373258919	5	Anpep
**1.17465450360802e-07**	0.410384378470249	0.00162231533493303	5	Atf4
**1.19404743645604e-07**	0.506384399572509	0.00164909891448944	5	Errfi1
**1.21443900078658e-07**	0.647325783375312	0.00167726170398634	5	Maff
**1.29733422838842e-07**	0.705622643589407	0.00179174830282725	5	Lgals3bp
**1.41397660021076e-07**	-1.528978078	0.00195284308255109	5	Tspan2
**1.44351956977402e-07**	-1.075618055	0.0019936448778149	5	Epas1
**1.47014207389576e-07**	0.830705396188188	0.00203041321825743	5	Nfil3
**1.47796872742441e-07**	0.834852439711112	0.00204122260944585	5	Hmox1
**1.50595158971384e-07**	-1.912084036	0.00207986974055378	5	Myom1
**1.60429722981173e-07**	0.615045635	0.00221569490409298	5	Mgst1
**1.6447868660406e-07**	1.023753806	0.00227161514068867	5	Lbp

**1.67502218169886e-07**	0.840563380982296	0.0023133731351443	5	Egfr
**1.69762594469453e-07**	-1.202003422	0.00234459119221761	5	Fam3c
**1.86885045290566e-07**	0.451881461468406	0.00258106936050801	5	Cd302
**1.8911475759335e-07**	0.798944083071867	0.00261186391712176	5	Uap1
**1.92085574749248e-07**	0.685971420315148	0.00265289387286186	5	Cebpb
**2.04777087985398e-07**	-2.153135799	0.00282817636216633	5	Cbr2
**2.06448167453629e-07**	-0.745419739485096	0.00285125564070207	5	Tns1
**2.50141508202108e-07**	1.421280371	0.00345470436977931	5	Ebf2
**2.5089995279715e-07**	0.613602677437729	0.00346517924808144	5	Gda
**2.54268990312656e-07**	1.069870479	0.00351170902520809	5	Cotl1
**2.61725886342655e-07**	-0.453602488866274	0.00361469621627841	5	Vcl
**2.63114394309634e-07**	0.709882458416357	0.00363387289981035	5	Gdf10
**3.07388347822794e-07**	0.557571813324578	0.0042453404717806	5	Fbln2
**3.17417954020977e-07**	1.075342026	0.00438385936298371	5	Wnt11
**3.44732838438206e-07**	0.375800271273332	0.00476110523167006	5	Adamts2
**3.59848224128031e-07**	0.485875801066442	0.00496986382343224	5	Pcolce2
**5.00221551487374e-07**	0.739506276828756	0.00690855984759213	5	Ccbe1
**5.20576983202183e-07**	0.504324218545076	0.00718968871500535	5	Islr
**5.44788566613737e-07**	-1.251992844	0.00752407489350232	5	Synpo2
**5.61657718949193e-07**	0.437502647658319	0.00775705475640731	5	Axl
**6.72949071114819e-07**	0.548273170105425	0.00929409962116677	5	Sash1
**6.82587002648667e-07**	-0.416035869120725	0.00942720909358074	5	Endod1
**7.04768454948988e-07**	0.614016524625716	0.00973355713130047	5	Map4k4
**7.82328464754051e-07**	0.458515283593627	0.0108047384267182	5	Adamts5
**8.16021942990758e-07**	0.475531536082324	0.0112700790546454	5	Litaf
**8.37181011693689e-07**	0.318461486	0.0115623069525015	5	Aebp1
**9.03118520746832e-07**	0.796925843298362	0.0124729698900345	5	Enpp3
**9.07773884593394e-07**	-0.949249821520739	0.0125372651201194	5	Syne1
**9.21734541435999e-07**	0.843667074592436	0.0127300757517726	5	Penk
**1.10893309311765e-06**	0.539242889282844	0.0153154749490479	5	Socs3
**1.16473789377539e-06**	0.945176246385872	0.0160861950509319	5	Baiap2
**1.17062885137266e-06**	0.533197834725051	0.0161675550663078	5	Serpina3n
**1.18386172841578e-06**	0.763804979665725	0.0163503143311504	5	Ube2e2
**1.22504479662438e-06**	-1.15569405	0.0169190936861793	5	Hbegf
**1.23076021897847e-06**	-1.255370405	0.0169980293843117	5	Cspg4
**1.25273123006712e-06**	0.723624852081541	0.017301471018457	5	Ackr3
**1.28645248441384e-06**	-0.829800992839495	0.0177671952622395	5	Kank1
**1.29887799172242e-06**	0.378486325937322	0.0179388039436784	5	Il33
**1.44666707912058e-06**	-2.030598028	0.0199799190297343	5	Fbxo32
**1.49039073264218e-06**	0.530862812247737	0.0205837864085212	5	Ddah2
**1.49835173878112e-06**	0.451542373133932	0.020693735864306	5	Zfp36
**1.51445270191182e-06**	0.571235650017599	0.0209161062661042	5	Tgfbr3
**1.53849601830279e-06**	0.4322465	0.0212481685087798	5	Col3a1
**1.55262406735699e-06**	-1.884685604	0.0214432909942673	5	Pcp4l1
**1.64879721472216e-06**	-1.30379961	0.0227715383325278	5	Nov
**1.66212759546209e-06**	0.609153629843274	0.0229556442209269	5	Tgif1
**1.68099209156594e-06**	0.349265459294059	0.0232161817766172	5	Celf2
**1.90621393279666e-06**	-1.920533975	0.0263267206258547	5	Npnt
**1.95536213500993e-06**	0.480333788	0.0270055064466222	5	Rbms1
**1.97392693518481e-06**	0.386538611	0.0272619049018374	5	Mmp2
**2.01432529330076e-06**	0.749604632	0.0278198466257768	5	Ripk1
**2.024202505267E-06**	0.405848786098348	0.0279562608002426	5	Npc2
**2.05836739757501e-06**	0.508109530302714	0.0284281121279084	5	Olfml3
**2.11505234901542e-06**	0.933058808850633	0.029210987992252	5	Il17ra
**2.1446465689486e-06**	0.668366978758098	0.0296197137637491	5	Abca9
**2.2014562887262e-06**	0.473325721912153	0.0304043128035976	5	Prg4

**2.23724893327532e-06**	0.661292054607189	0.0308986450174654	5	Irf1
**2.27982913829357e-06**	0.883290082493336	0.0314867202289725	5	Fam102b
**2.55452313875893e-06**	-1.044344766	0.0352805190693996	5	Dmpk
**2.67702415117167e-06**	0.892028363166816	0.0369723805518319	5	Sfrp4
**2.70619536654987e-06**	-0.627800027245482	0.0373752642074203	5	Ctnnb1
**2.7282068935193e-06**	-1.042433409	0.037679265406395	5	Itga9
**2.80947383773813e-06**	0.686637244747722	0.0388016431730014	5	Lpl
**2.83803983039769e-06**	0.792485883403742	0.0391961680976225	5	Tiparp
**3.04503450638858e-06**	0.942174542690291	0.0420549715677326	5	Tnfaip3
**3.15690817718618e-06**	0.444071337684688	0.0436000588351184	5	Csrnp1
**3.22042702647559e-06**	0.372183613111588	0.0444773176626544	5	Gadd45b
**3.2489199901347e-06**	0.553772132259966	0.0448708339837503	5	Lpar1
**3.33724765119346e-06**	-0.565249843065738	0.0460907273106329	5	Gpx1
**3.41918655094368e-06**	-0.805031903465005	0.0472223854550831	5	Lrrfip1
**3.61976115356987e-06**	0.462703277527978	0.0499925212919535	5	Il1r1
**4.01674067073769e-06**	-0.639674069884165	0.0554752054035583	5	Crip2
**4.03170456937322e-06**	0.516216942182956	0.0556818718076135	5	Man2b2
**4.04341268577149e-06**	-0.962664937647542	0.0558435726031901	5	Synpo
**4.13616889774155e-06**	-1.642585269	0.0571246286467085	5	2200002D01
**4.38835916180087e-06**	0.621490725082071	0.0606076283836318	5	Sbno2
**4.51224731104956e-06**	-0.629506948354673	0.0623186476129054	5	Flna
**4.9025986061517e-06**	-1.675337837	0.0677097893495612	5	Tinagl1
**4.93657539897752e-06**	0.498479950785132	0.0681790428352785	5	Igsf10
**4.95741515816544e-06**	-1.167588944	0.068466860749423	5	Limch1
**4.96954280093649e-06**	-0.543243081083565	0.0686343556237339	5	Dusp3
**5.02229986454296e-06**	-2.023536067	0.0693629834292028	5	Sost
**5.02805560300289e-06**	0.647553253089485	0.0694424759330729	5	Plat
**5.46850195210279e-06**	0.449589612580884	0.0755254804604917	5	Ddr2
**5.73974949110141e-06**	0.686171473085233	0.0792716802216015	5	Ms4a4d
**5.78735476770941e-06**	0.319650711042068	0.0799291566968347	5	Il11ra1
**5.8879098310214e-06**	-1.267943847	0.0813179226762365	5	Lims2
**6.1637754576569e-06**	0.722129765414135	0.0851279028456994	5	Adamtsl1
**7.05384664774145e-06**	0.388400929801703	0.0974206760519571	5	Lima1
**7.48468209622344e-06**	-0.632446467021476	0.103370944430942	5	Cald1
**7.53195446038316e-06**	0.301920668900893	0.104023823052352	5	Calr
**7.8414280728666e-06**	-1.34068677	0.108297963114361	5	Mtus2
**7.97807961090718e-06**	0.598096453019084	0.110185257506239	5	Plscr1
**8.05194790008223e-06**	0.672834234664612	0.111205452448036	5	Fnip1
**8.20673981710006e-06**	0.611376953149912	0.113343283613969	5	Birc3
**8.50632877815931e-06**	0.568088241400236	0.117480906755158	5	Tshz2
**8.77071497360407e-06**	0.574693342270511	0.121132344500446	5	Nsg1
**8.77734664560623e-06**	0.307935249052212	0.121223934522468	5	Fosb
**8.81011320332148e-06**	0.483906309	0.121676473451073	5	Tmem100
**8.8397490486567e-06**	-1.698755049	0.122085774110998	5	Pecam1
**9.08994448500353e-06**	0.391120513	0.125541223282384	5	Lamp2
**9.19705298225903e-06**	0.800914682968701	0.127020498737979	5	Tmeff2
**9.24234553079155e-06**	-1.158963086	0.127646034125762	5	Bcam
**9.37533614402056e-06**	0.480993499496969	0.129482767485068	5	Timp1
**9.47454354186035e-06**	0.321389111908522	0.130852920856633	5	C4a
**9.48036024435303e-06**	0.523459311817283	0.130933255	5	Ifrd1
**9.50801755218587e-06**	-1.663285947	0.131315230413239	5	Thsd4
**9.83922320195117e-06**	-1.427499403	0.135889511642148	5	Pdlim3
**1.06487929424828e-05**	0.504703981659967	0.147070479328629	5	Mmp14
**1.09552740527306e-05**	0.360753611372222	0.151303289942262	5	Hnrnpa2b1
**1.11065407698417e-05**	-1.221363088	0.153392434572284	5	Smtn
**1.1443386259065e-05**	-0.518742921400936	0.158044607623946	5	Tmsb4x

**1.22610405023086e-05**	0.64630438	0.169337230377384	5	Ngf
**1.26485809670996e-05**	-0.761032280246101	0.174689551736613	5	Oxct1
**1.50152472049565e-05**	0.779016402355342	0.207375579147654	5	Lrrc49
**1.50832602324693e-05**	1.812297015	0.208314907070634	5	Hp
**1.54944641944142e-05**	-1.827433401	0.213994044989054	5	Kcnmb1
**1.58104592950973e-05**	0.646882880880436	0.218358253324589	5	Slc16a2
**1.61884649038987e-05**	0.666363583547915	0.223578888787745	5	Thy1
**1.62000391768846e-05**	-0.808145356491467	0.223738741071954	5	Ppp1r12a
**1.67945107510402e-05**	0.311969368988789	0.231948987982616	5	Junb
**1.6957944769526e-05**	-0.828614652559691	0.234206175211924	5	Pcdh7
**1.77197582026264e-05**	0.873247041211707	0.244727580536473	5	Bmper
**1.82637167993999e-05**	1.625342368	0.252240192716513	5	Kazn
**1.846168842652E-05**	0.441177673261424	0.254974378858668	5	Mmp23
**1.85822649348587e-05**	-0.792591043862431	0.256639661015334	5	Kctd10
**1.86005723548579e-05**	0.428319586056258	0.256892504792942	5	Meg3
**1.87005656846343e-05**	-1.054556824	0.258273512670485	5	Nexn
**1.89393975313804e-05**	-1.471457368	0.261572019305895	5	Gucy1b3
**1.90360184536231e-05**	-1.205216423	0.262906450862989	5	Stom
**1.94033564808023e-05**	-0.960533757508383	0.267979756	5	Myo1e
**2.01513069406615e-05**	-0.486268618455036	0.278309700157475	5	Pnpla2
**2.02255943202186e-05**	0.346420267407744	0.279335683156538	5	Sfpq
**2.13819181018976e-05**	0.556083532209085	0.295305670905308	5	B4galt5
**2.29567996125083e-05**	0.307236725548073	0.317056359448352	5	Rps15a-ps6
**2.33025723392965e-05**	-0.341021790187892	0.321831826578024	5	Hes1
**2.45563760510385e-05**	-0.859300841419344	0.339148109640893	5	Postn
**2.51596267799398e-05**	0.789443523345976	0.347479605457748	5	Sqrdl
**2.58422458830687e-05**	-0.938359402691247	0.356907257891062	5	Cav2
**2.60300275381254e-05**	0.528115077949645	0.359500710329049	5	Thbd
**2.71124262216146e-05**	-1.808637736	0.374449718546719	5	Rgs4
**2.75525559280532e-05**	-1.50251477	0.380528349922343	5	Ppp1r14a
**2.78876245932338e-05**	0.722029184889502	0.385155983257152	5	Trex1
**2.87562806729417e-05**	0.492085493923628	0.397152992373997	5	Fbn1
**2.89058997068039e-05**	0.553826278691814	0.399219380850669	5	Olfml2b
**2.93408171582679e-05**	0.265898524792154	0.405226025772837	5	Egr1
**3.08340396092161e-05**	0.634178511011278	0.425848921042884	5	Pdia4
**3.39265184734496e-05**	-1.355430936	0.468559146636812	5	Ptprb
**3.44187374531803e-05**	-0.573303529655708	0.475357182965873	5	Serpine2
**3.4440377611814e-05**	0.646681897964665	0.475656055196764	5	Cyp27a1
**3.46563832779154e-05**	-1.506282469	0.478639309451289	5	Speg
**3.47598543815249e-05**	0.925129238012994	0.480068349	5	Irak3
**3.48431361116916e-05**	0.283475416836017	0.481218552838572	5	Ogn
**3.74099350877036e-05**	0.504586276685273	0.516668613496274	5	Lgals9
**3.79966784044961e-05**	0.477852121330324	0.524772125444496	5	Swi5
**3.80764980799053e-05**	0.530699684779278	0.525874514981572	5	Medag
**3.86007275252478e-05**	-1.182947422	0.533114647851197	5	Plekhg3
**3.90255648656615e-05**	-1.283309346	0.538982076359651	5	Cdh13
**3.95440277065273e-05**	-1.580691452	0.546142566654849	5	Nes
**4.04907429842382e-05**	0.357158750057938	0.559217651355314	5	Trip10
**4.12959139053292e-05**	0.592910891494601	0.570337866946501	5	Hivep2
**4.1513752283346e-05**	0.774171276	0.573346432785291	5	Tubb6
**4.27401290745763e-05**	0.363993839053592	0.590283922648974	5	Serpinh1
**4.36550511830958e-05**	1.728000371	0.602919911889736	5	Ccl8
**4.37067584704556e-05**	0.706687781614316	0.603634041235462	5	Thbs3
**4.43913046236585e-05**	-1.222149089	0.613088308157348	5	Gpr116
**4.59989337059494e-05**	0.373020602802455	0.635291273412867	5	Pmp22
**4.93425218346553e-05**	-0.950692839289843	0.681469569058425	5	Cav1

**5.11407071325043e-05**	0.508653202095596	0.706304306207017	5	Ier5
**5.30958139556681e-05**	0.330831751260808	0.733306286541733	5	Eif4a1
**5.5493554099265e-05**	0.472605464145545	0.766421475664949	5	Fam46a
**5.70372779737804e-05**	0.512223653527742	0.787741846	5	Ctsk
**5.76392687271065e-05**	-1.128958779	0.796055940390069	5	Mgll
**5.79210944907141e-05**	0.718177899669736	0.799948236011253	5	Ptp4a1
**5.80287006410632e-05**	-0.870599973553932	0.801434384553724	5	Cytl1
**5.99688739184022e-05**	0.282931299535643	0.828230117687053	5	Klf4
**6.03985022636031e-05**	-0.541102143482628	0.834163714762623	5	Ccnd2
**6.18644624945946e-05**	-0.400369030437805	0.854410091512846	5	Dst
**6.68636088041753e-05**	-1.200817313	0.923453301194465	5	Fgf1
**6.79159914538057e-05**	1.178593889	0.937987757968511	5	Sult5a1
**7.01196378019707e-05**	-0.511192406306074	0.968422317683018	5	Hspa1a
**7.01506297504235e-05**	-0.931601774	0.968850347483099	5	Bmp4
**7.10860980843017e-05**	0.884684788696813	0.981770100642291	5	Ssbp2
**7.19728926828538e-05**	0.356759670554237	0.994017620842893	5	Daam1
**7.37692053308672e-05**	0.771874229716183	1	5	Pdpn
**7.56578163651803e-05**	-0.532550650839762	1	5	Col4a6
**7.92551491516333e-05**	0.676971133792162	1	5	C1qtnf1
**8.000091042605E-05**	0.406556604417282	1	5	Serp1
**8.13246359027607e-05**	-0.324653029024429	1	5	Pam
**8.23801376372059e-05**	-1.649561289	1	5	Sh3bgr
**8.33865912988941e-05**	-1.933669282	1	5	Mmrn2
**8.55878737532906e-05**	0.273344810364679	1	5	Svep1
**8.64671119775291e-05**	0.316716452467813	1	5	Ppib
**8.69744189799837e-05**	0.347265725202141	1	5	Col5a1
**8.76784577229746e-05**	-1.971931026	1	5	Hspb6
**8.84670703457102e-05**	1.025722281	1	5	Arsa
**9.11140031110876e-05**	0.617147698508769	1	5	Podn
**9.30479540120673e-05**	-1.173492377	1	5	Kif1c
**9.41048350234666e-05**	-1.386984757	1	5	Slc9a3r2
**9.52550697560714e-05**	-0.623483723421111	1	5	Atp2b4
**9.929256275357E-05**	0.256507120962371	1	5	Sepp1
**0.000101002005932164**	1.203732887	1	5	Lif
**0.000101555476465797**	-1.3240068	1	5	Tspan7
**0.000105666051423638**	-0.504012085286861	1	5	Rock1
**0.000107300557709298**	0.672985724307066	1	5	Pamr1
**0.000109044657789033**	-0.975979580960615	1	5	Uchl1
**0.000109586930236479**	-0.747911266	1	5	Mical2
**0.000112493107091599**	0.364988023622853	1	5	Napa
**0.000113857797264968**	0.399500758810343	1	5	Lsp1
**0.00011401285646193**	0.373563854767144	1	5	Nav1
**0.000114607195397335**	-0.550792001641143	1	5	Wwtr1
**0.000116698661294244**	0.540750424644536	1	5	Gpnmb
**0.000119199016314242**	-1.235320542	1	5	Clec14a
**0.000119905648537078**	0.823811558705165	1	5	Tnnt3
**0.000122184731406302**	-0.267829492445085	1	5	Mgp
**0.000122782999526539**	0.820656425472405	1	5	Pcdh19
**0.000124215727871601**	-0.772476623240904	1	5	Col18a1
**0.000125609595026478**	0.683759685621414	1	5	Xdh
**0.000126857012730632**	0.555770640589724	1	5	Man2b1
**0.000130054655515471**	0.429328098329881	1	5	Clip4
**0.000132945645159485**	0.286776019423006	1	5	Serpinb6a
**0.000134282348654379**	-0.342207053585455	1	5	Pmepa1
**0.000135999302103149**	-1.801714735	1	5	Bmx
**0.000136586372891232**	-1.395947447	1	5	Asap2

**0.000141579477876777**	0.589958905437087	1	5	Prrx1
**0.000141723100576286**	0.527712921041852	1	5	Dennd2a
**0.000144956358823675**	-0.545100794	1	5	Dkk3
**0.000148457035566503**	0.378497867464613	1	5	Vkorc1
**0.000149805958199249**	0.700276689340495	1	5	Dram1
**0.000150436035427524**	0.501747851522277	1	5	Hspb8
**0.000155041160807289**	-1.127567618	1	5	Crim1
**0.000155924255216353**	-0.774329807841656	1	5	Myl6
**0.000157909164012743**	-0.859998286715809	1	5	Fbxo22
**0.000164795434241837**	-0.736967874127523	1	5	Lama5
**0.000164961561159029**	-0.735713364914061	1	5	Samd4
**0.000165606249301076**	0.455898423616983	1	5	Pnp
**0.000167753258060759**	0.401469050518513	1	5	Fcgrt
**0.000168424319651954**	0.659444566750802	1	5	Ifi203
**0.000171669266999048**	-0.866198822500207	1	5	Gm13889
**0.000172969474452313**	-0.828456055927902	1	5	1500015O10I
**0.000174525801659206**	0.612892623	1	5	Gja1
**0.000179583738991542**	0.276573250049627	1	5	Col1a1
**0.00018579721064658**	-1.633325292	1	5	Fmo3
**0.00018622432767786**	-0.785205393027465	1	5	Msrb3
**0.000186604300100269**	-1.377021696	1	5	Serpinb6b
**0.000192772298765199**	-1.549227993	1	5	Tnk2
**0.000195311426045637**	0.570433189094505	1	5	Rftn1
**0.000197168299408594**	0.339991664061106	1	5	Nbl1
**0.000197841057779151**	0.387196507192843	1	5	Bag3
**0.000197948348998478**	0.361133138380217	1	5	Lamc1
**0.000203094772608296**	0.624511105609141	1	5	Ston2
**0.000203750113101228**	0.483281275603071	1	5	Nfkb1
**0.000205102140728826**	0.274008408855759	1	5	Rpn2
**0.000208890212699989**	-1.443986706	1	5	Rbpms2
**0.000211607354457169**	0.314588235	1	5	Vcan
**0.000212919542004283**	-0.861582388339245	1	5	Smarcd3
**0.000229348473427006**	0.448687627929454	1	5	Ugp2
**0.000233075220505496**	-0.843138401	1	5	Frzb
**0.000235236922268455**	0.412047412099803	1	5	Rad23a
**0.000239256742069287**	-1.753624259	1	5	Nos3
**0.000242045878291362**	-0.613848216454358	1	5	Tgm2
**0.000244755766172328**	0.258199723809866	1	5	Sqstm1
**0.000250937848494012**	-1.202315803	1	5	Nxn
**0.000253092486026924**	0.704043172135972	1	5	Flrt2
**0.000254735233545829**	-0.764693703070644	1	5	Adh5
**0.000258991782096041**	0.654387252445553	1	5	Irgm1
**0.000261473766491601**	-0.866726564477965	1	5	Pi15
**0.000262036066791576**	0.509165886903776	1	5	Adamtsl3
**0.000270685830334799**	0.994724320326671	1	5	Galnt16
**0.000270975763900246**	0.556202735888691	1	5	Gaa
**0.000275705027150369**	0.619030829	1	5	Usp16
**0.000275758648382963**	-0.756386148554838	1	5	Ptdss1
**0.00027604718214683**	0.277655889597401	1	5	Cd47
**0.000276139309106021**	-1.191568735	1	5	Heyl
**0.000281028855255218**	-0.656777993756491	1	5	Chtop
**0.000289603573724285**	-1.116561379	1	5	Fam13c
**0.000298706989433285**	1.205730722	1	5	Isg20
**0.000304035399180457**	-1.225308148	1	5	St3gal5
**0.000307058764092121**	0.336131563652339	1	5	Sntb2
**0.000308447814436057**	0.625420947987262	1	5	Mndal

**0.000308866805834763**	0.527628073632154	1	5	Olfml1
**0.000310330762897048**	-1.373613116	1	5	Tom1l2
**0.000310819926814209**	-0.836836519141834	1	5	Vasp
**0.000316274457195218**	-1.166594102	1	5	Mrvi1
**0.000320180325155281**	0.356768516	1	5	Zcchc24
**0.000323293242119517**	-1.076352203	1	5	Ano10
**0.000328803353399914**	-0.703549493500236	1	5	Cd97
**0.000330811144469158**	-1.727132644	1	5	Tes
**0.000331293847330952**	0.323641002839225	1	5	Rpsa
**0.00033385721350331**	0.608439094960897	1	5	Pgap2
**0.000334033472308145**	0.347097064744911	1	5	Lama2
**0.000338192009409846**	0.477807505	1	5	Jmjd1c
**0.000341331421035541**	0.346719050881818	1	5	Epb4.1l2
**0.000343253467877964**	0.626630843192758	1	5	Shmt2
**0.000348332119340794**	-0.702344919738361	1	5	Id1
**0.00035136096833386**	0.827079944664021	1	5	Mms19
**0.000353542850145798**	0.428392153354799	1	5	Fkbp2
**0.00035555187978584**	-0.702160410996681	1	5	Tmem47
**0.000357204509284907**	-0.804503053354126	1	5	Pbxip1
**0.000358141122592225**	0.634342300489419	1	5	Lamb1
**0.000361207720900411**	0.324411184680738	1	5	Tubb4b
**0.000362855962421431**	1.430236349	1	5	Arhgap28
**0.000366081230247131**	-0.961793200655511	1	5	Map1a
**0.000370330096741633**	-1.453205053	1	5	Npy1r
**0.000371362071582822**	0.684296776794578	1	5	Abcb1b
**0.000377006620559795**	0.337166520898032	1	5	Zeb2
**0.000383099528853723**	0.259205030027642	1	5	Ifi27l2a
**0.000388898768662935**	0.279503181073264	1	5	Pdia6
**0.000391092134256982**	0.586924244243806	1	5	Ssc5d
**0.000395618799796898**	-0.656037566130983	1	5	Phldb2
**0.000397294215306484**	-0.595544087061294	1	5	Ndufb10
**0.000412992140080735**	-0.734814196584128	1	5	S100a4
**0.000424862326315575**	0.445640970560947	1	5	Clec11a
**0.000438366519564022**	-0.527035440565845	1	5	Ptgis
**0.00044699792214496**	0.467600223149723	1	5	Runx1
**0.000461903284783185**	0.669763556454213	1	5	Tsta3
**0.000466388536379904**	-0.361185651056496	1	5	Fnta
**0.000470028819970171**	-0.644000608864897	1	5	Actg2
**0.000474089574684484**	-0.354929625983028	1	5	Col4a1
**0.00047694052441512**	0.346466346435049	1	5	Igf1
**0.00047809693274436**	0.562689689488643	1	5	Tnfsf13b
**0.000478571271240529**	0.825250871914077	1	5	Arid5a
**0.000482257508845199**	-1.012928514	1	5	Itga7
**0.000484357017202518**	-1.037010928	1	5	Pomgnt1
**0.000488418366639915**	-1.53081987	1	5	Icam2
**0.000492015715883272**	0.567468543242253	1	5	Slc10a6
**0.000508275668240853**	-0.618753805494656	1	5	Tnfaip1
**0.000516144758120659**	-0.529120442593618	1	5	Iqgap1
**0.000522368301726821**	0.342973058781326	1	5	Efemp2
**0.00052585680253545**	0.307043563941377	1	5	Gpc6
**0.000526791279639949**	0.687418970046203	1	5	Pim3
**0.000535587240841652**	0.255034679482328	1	5	Usp2
**0.000556183139250149**	0.988885726780348	1	5	Smg7
**0.000566708208540252**	-1.687842329	1	5	Podxl
**0.00057503898830598**	0.453191920043877	1	5	Scd1
**0.000577388208188401**	0.929807190002403	1	5	Itga11

**0.000578026762530315**	0.613907312888622	1	5	C1galt1
**0.000588065889861372**	-0.524033221873084	1	5	Trp53inp2
**0.00059213023607862**	0.282562522300288	1	5	Gabarap
**0.000593639181671507**	0.250653132611315	1	5	Ubc
**0.000596011178331422**	0.382407227031717	1	5	Sf3b1
**0.000599338196942466**	0.263070967	1	5	Arf4
**0.000599847818296277**	-1.122487154	1	5	Itgb3
**0.000610577818177194**	0.269936594	1	5	Col6a1
**0.000611864927887162**	0.901391245028108	1	5	Zc3h12a
**0.000612312567360286**	0.717113660399027	1	5	Sobp
**0.000619321018197031**	-1.185497548	1	5	Inpp5a
**0.000621398592825171**	-0.482142329704908	1	5	Col4a2
**0.000621655652411159**	-0.399049087383692	1	5	Serinc3
**0.000623600646311854**	0.423349989729068	1	5	Pltp
**0.000637093341303553**	0.448221321046905	1	5	Gosr2
**0.000639561269438628**	0.652697912	1	5	Loxl2
**0.000641271030367214**	-0.659860546	1	5	Filip1l
**0.000643837950196017**	0.502842432296936	1	5	Ssh2
**0.000668090034018884**	-0.573908817589931	1	5	Fert2
**0.000675977936752022**	0.272248523	1	5	Rps15a
**0.000682375176464996**	-1.228591994	1	5	Ltbp2
**0.000683303870730507**	0.534423589	1	5	Emc2
**0.000692288308321263**	-0.388602901124913	1	5	Cyb5r3
**0.000701803037643213**	-0.551630198772409	1	5	Nol7
**0.000709511573364034**	0.471788738590657	1	5	Gna13
**0.000712268806286771**	0.623955735356773	1	5	Prrx2
**0.000714811055869185**	-0.579897962294029	1	5	Rabif
**0.000735504624727368**	0.460966890416503	1	5	Asap3
**0.000736943839808867**	1.122297245	1	5	Smpd3
**0.000747935477637597**	-0.536769270640474	1	5	Psmc3
**0.000754701381475503**	-1.471552478	1	5	Cd93
**0.000755302973962271**	0.535042593992394	1	5	Igsf8
**0.000756570382927577**	0.283627391415904	1	5	Ppp1r15b
**0.000761739056023612**	0.803987593388689	1	5	Gpr39
**0.000762284705385601**	-0.724609839532823	1	5	Nuak1
**0.000765154062154574**	0.669779636671526	1	5	Cish
**0.000777834760315207**	0.657930407687842	1	5	Sema3b
**0.000783601841619906**	0.383896163748981	1	5	Camk2n1
**0.000791721631061698**	0.514035810298898	1	5	Creb5
**0.000792148990355779**	0.278379414336698	1	5	Gadd45g
**0.000805996239901499**	0.379242480396495	1	5	Grn
**0.000809028256065496**	-0.736376463721454	1	5	Rasl12
**0.000810900983254539**	-1.028154839	1	5	Rassf3
**0.000813238002499526**	0.579551802848632	1	5	Adam33
**0.000824287633058073**	0.686098568189637	1	5	Nubp2
**0.000828085253995038**	0.402360375515936	1	5	Plekhh2
**0.000828960979251685**	0.561244628458182	1	5	Ppapdc1b
**0.000831091662977296**	0.415029581914899	1	5	Hexa
**0.00083351596279271**	-1.036906679	1	5	Sort1
**0.000836761855260016**	0.281672579688018	1	5	Dnajb9
**0.000839806607962389**	-0.411214003331627	1	5	Myh10
**0.000848263574026371**	-0.386353016969187	1	5	Tsc22d1
**0.000851607631396391**	-0.640268985617308	1	5	Inhba
**0.000857137995360326**	-0.887686796624735	1	5	Agrn
**0.000866481548306894**	0.258362779227218	1	5	Kcnq1ot1
**0.000873494835581108**	0.327921817088596	1	5	Ddost

**0.000893166570449105**	-0.737149381796721	1	5	Fry
**0.000895983469901709**	0.572498990057297	1	5	Rfx5
**0.00089941042219239**	0.275271757512354	1	5	Lum
**0.000899711497462847**	-0.604891052032056	1	5	Rbpms
**0.000900598549696416**	-0.758744705256122	1	5	Arhgef2
**0.000908878896698018**	0.729106110947389	1	5	Lrrc61
**0.000911589666654501**	0.857336495359816	1	5	Fgf7
**0.000920452206505473**	-0.340354106889532	1	5	Arhgef17
**0.000931641088467776**	-0.977653529743577	1	5	Fam135a
**0.000943640357629076**	0.284048054660586	1	5	Fbln7
**0.000948554483338151**	-0.339808118497721	1	5	Ltbp3
**0.000949046435044617**	0.597339369234376	1	5	Abca8b
**0.000961283289884589**	1.054563717	1	5	Has2
**0.000983951598906779**	-1.850079082	1	5	Ptprz1
**0.000994986594865264**	0.753968462050573	1	5	Ccl19
**0.000995238833691447**	-1.011721314	1	5	Fam126b
**0.000995519561634434**	-1.557675196	1	5	Gja4
**0.00099801482320739**	-0.740608319910388	1	5	F2r
**0.00102441442549744**	0.344749016933775	1	5	Scara3
**0.00103234279883904**	0.678578326879416	1	5	Slc44a1
**0.00103328662934195**	-1.562139249	1	5	Efcab4a
**0.00103710075733919**	-0.721370328604463	1	5	Cep290
**0.00104992517238172**	-0.549861917248617	1	5	Csrp2
**0.00106375254595909**	-1.104608926	1	5	Syde1
**0.00106638919711396**	-0.802291917739549	1	5	Ano1
**0.00108246157700658**	-0.528878203379864	1	5	Cox5a
**0.00108395137771293**	0.277847518	1	5	Dpp9
**0.001086004601216**	0.279055558403718	1	5	Abi3bp
**0.00108711255557821**	0.423134563028658	1	5	Zfp945
**0.00109286255002273**	-0.424845518785915	1	5	Dgka
**0.00111302046491861**	-1.214840687	1	5	Hrct1
**0.00114437542188467**	-0.676315561538028	1	5	Polr2g
**0.00115071527088439**	-0.682987695733832	1	5	Zc2hc1a
**0.0011541570836963**	0.361739291437883	1	5	Tgfbi
**0.00120497184155092**	0.417388107665506	1	5	Naga
**0.00121687644560742**	-1.71666378	1	5	E030013I19R
**0.00124128310378926**	-0.887195151247233	1	5	Cdh5
**0.00125025029730782**	0.267269428869747	1	5	Nfkbiz
**0.00125702129042439**	-0.922515405372782	1	5	Arhgef18
**0.00126405494664957**	0.345210699928642	1	5	Gba
**0.00126617720707019**	0.259774422107498	1	5	Gm6654
**0.00126760740604957**	0.559112207135149	1	5	Shc2
**0.00129914622134743**	0.564721631277025	1	5	Mmp19
**0.00130127487923135**	-0.540415104978865	1	5	Cdc42bpa
**0.00131775553797515**	-0.452791858797451	1	5	Nsmce4a
**0.00132187382101157**	0.539214698740906	1	5	Casp4
**0.0013430692499414**	0.299091297355717	1	5	Fgfr1
**0.00134617052351878**	-0.466991935515422	1	5	Atp5f1
**0.00135264934686406**	0.372117490720012	1	5	H3f3b
**0.00136279433542898**	0.273124746082334	1	5	Ier3
**0.00137289235473321**	0.552685632702364	1	5	Dtx4
**0.00137788684456239**	-0.570560763349837	1	5	2900026A02F
**0.00138008494580484**	-0.767860719347456	1	5	Rnf220
**0.00138809159665347**	-0.610036389529225	1	5	Hip1
**0.00140280809307456**	-1.436795005	1	5	Sorbs2
**0.00140978554290544**	-1.106567069	1	5	Adck3

**0.00141466976375068**	-0.991533348627671	1	5	Rcan2
**0.00142936097101561**	-0.565769011298559	1	5	Bnip2
**0.00144050004732709**	-0.529625688221171	1	5	Bbip1
**0.00144223785489501**	0.407409297001162	1	5	Ifnar2
**0.00146852800569533**	-0.861209417081166	1	5	Ptpn14
**0.0015081503555359**	0.516543124608681	1	5	Tnfrsf1a
**0.00153472691536435**	-0.270966287810579	1	5	Calm1
**0.00155784609314298**	-0.871909502495095	1	5	Ppp1r11
**0.00156577464090233**	-0.514979708289548	1	5	Mrps12
**0.00156792997908405**	0.390194813256164	1	5	Eif4g1
**0.00158021649731294**	0.353900077559136	1	5	Rpl39
**0.00162295947341527**	-0.469778551667785	1	5	Cacna2d1
**0.00165160598866868**	0.672521021174089	1	5	Ccng2
**0.00165565844087475**	1.102737714	1	5	Ang
**0.00166880155496927**	0.842686237180709	1	5	Slc16a9
**0.00170832896025586**	-0.767347360718226	1	5	2210013O21I
**0.00171899973289486**	-0.689961474934146	1	5	Mef2c
**0.00173157132214689**	-0.851530558467147	1	5	Slc4a2
**0.00174308768284445**	-0.787159802371382	1	5	Marveld1
**0.00174955674216949**	0.476390140741195	1	5	Rhog
**0.00175036447396751**	0.395978223197192	1	5	Jak1
**0.0017663924591582**	-0.411522756557342	1	5	Prkag1
**0.00177332765565638**	0.336489145317074	1	5	Fuca1
**0.00179280769840537**	-0.538780525784941	1	5	Mfge8
**0.0018288677792923**	0.441871709017586	1	5	Arhgap10
**0.00185422223783058**	0.400793596509221	1	5	Ndel1
**0.00185548705187992**	0.519437546696566	1	5	Gramd1a
**0.00185778100590224**	-0.929306439138603	1	5	Herc1
**0.00186567168947613**	0.330393801960439	1	5	Rpl22l1
**0.00186579373881002**	-0.759980252631376	1	5	Rhobtb1
**0.00190291147576987**	0.447821268803343	1	5	Naalad2
**0.0019066064628591**	0.493761086	1	5	A4galt
**0.00190859370006701**	0.430669322689269	1	5	Arhgap21
**0.00193827458120514**	-1.058898191	1	5	Tmtc2
**0.001952993794463**	-0.662177705927333	1	5	Chpt1
**0.00198859625972688**	0.264325475	1	5	Cstb
**0.00198957654085592**	0.410015071796322	1	5	Plekhf1
**0.00200810159240904**	-0.734746484518376	1	5	Sema5a
**0.00204490438379081**	0.288226879997076	1	5	Eif2s2
**0.00207772755867429**	-1.6122145	1	5	Net1
**0.00208199823957036**	-0.469175471848507	1	5	Map4
**0.00208753396625111**	0.429982712361543	1	5	Osmr
**0.0021064076823161**	-1.148417448	1	5	Sorbs1
**0.00210891379500048**	-0.867680683933153	1	5	Spag7
**0.00213324205316983**	-0.648412930429428	1	5	Cers4
**0.00215229173524841**	-1.420999146	1	5	Gcnt1
**0.00216887942475748**	0.306900245486694	1	5	Tcn2
**0.00217917684635698**	-0.948095581	1	5	Tspan6
**0.00218440735143339**	-0.502576020040888	1	5	Smarca4
**0.00220579234220756**	-1.417069806	1	5	Selp
**0.002205908386712**	0.334849968505866	1	5	Cxxc1
**0.00222120375256888**	-0.661477270274337	1	5	Plxna4
**0.00223011536572505**	-0.556469967125799	1	5	Atp1a2
**0.002231038298857**	-0.564960231925866	1	5	Ppp1cb
**0.00224468093341453**	-0.634154305550341	1	5	2310036O22I
**0.00224834546354096**	-0.628413819320301	1	5	Slc48a1

**0.00225566893843515**	-0.440232557526153		1	5	Flnb
**0.00229099583954172**	-0.655883703643481		1	5	Hook3
**0.00230349088515508**	0.463402945214383		1	5	Aff3
**0.00239851761108786**	0.286180149351412		1	5	Ctsz
**0.00241346537675974**	0.263088819554548		1	5	Nme2
**0.00243786370447324**	0.483706521687882		1	5	Naglu
**0.00243791966029327**	-0.339426608499545		1	5	Hspb1
**0.00245943421245439**	0.349709894809525		1	5	Dhx32
**0.00252987906925261**	0.736126177987442		1	5	Mta3
**0.00253442933826221**	0.506295333848947		1	5	Qpct
**0.00253671733044107**	-0.686410940001775		1	5	Rhob
**0.0025739927095525**	-0.827416989036729		1	5	2010111I01R
**0.00257944950391487**	-0.399970746258984		1	5	Akap9
**0.00259879484023137**	0.337834205012878		1	5	Ubtf
**0.00261029624544346**	-0.731901693485003		1	5	Mrpl15
**0.00264495822358921**	0.289317045031211		1	5	Icam1
**0.00264772253422935**	-0.907108172579675		1	5	Atp2a3
**0.00265426979247835**	0.527038531753363		1	5	Txnrd1
**0.00266306466825442**	-0.752198413208933		1	5	Tgfb1i1
**0.00267731875530367**	-1.475072225		1	5	5930403L14F
**0.0026863790111717**	0.299269986997618		1	5	Hnrnph1
**0.00269915222140758**	0.616898102238751		1	5	Cpeb2
**0.00270138886956081**	0.592785503711098		1	5	Galm
**0.00270419781819792**	0.723573771330246		1	5	Procr
**0.00270730953664545**	-0.338875721834751		1	5	Rras
**0.00271848050137209**	0.741806165770575		1	5	Pvr
**0.00272299705436455**	-1.457042865		1	5	Ankrd1
**0.00272536512983038**	-0.261621460104635		1	5	Jmy
**0.00272639550265967**	-0.946395782846266		1	5	Wdfy3
**0.00273065385148858**	0.323163333854743		1	5	Bzw1
**0.00274754036038264**	-0.598409359		1	5	1110001J03F
**0.00275326974307628**	0.313084119506603		1	5	Rpl38
**0.00275486535642335**	0.540467312721708		1	5	Bdh2
**0.00276874226652197**	-0.749414557762226		1	5	Zak
**0.00278006730845941**	0.263601383156832		1	5	Epb4.1l3
**0.00279177319072564**	-0.452022027382954		1	5	Wls
**0.00280588809598074**	0.277881385689758		1	5	Hfe
**0.00284070249493887**	0.343819729053852		1	5	Eif4b
**0.00288060104854676**	0.264799897143133		1	5	Zc3h11a
**0.00288874926968715**	0.408343168031356		1	5	Cluap1
**0.00291076032559576**	-1.714087968		1	5	F11r
**0.00295147384128474**	0.953012729830102		1	5	Dot1l
**0.00297443625323403**	0.672647399062966		1	5	Stk19
**0.00298901214497821**	0.266546051346294		1	5	H6pd
**0.00301201753480334**	0.474095445051449		1	5	Ncstn
**0.00303406239997853**	-1.081730337		1	5	Fryl
**0.00304501168604299**	0.461132796679844		1	5	Bscl2
**0.00307011099391086**	0.358215547		1	5	Sema3c
**0.00307728585949186**	0.516611207490084		1	5	Hn1
**0.00309738085977597**	-0.849747531558443		1	5	Dusp8
**0.00312338881459144**	0.271644221		1	5	Grina
**0.00313696433276717**	0.386480166212139		1	5	Gem
**0.00314991645876728**	-1.607579002		1	5	Gja5
**0.00317966207914261**	-0.981704443023591		1	5	Phtf2
**0.00319005675113269**	0.431327338569531		1	5	Pcdh18
**0.00322365424566824**	0.629472996531472		1	5	Ube2j1

**0.00333981704270035**	-1.319182148	1	5	Esam
**0.00338791506983068**	0.412534770693418	1	5	Sh3kbp1
**0.00345141830820011**	-0.556575261774389	1	5	Strbp
**0.0034730369903777**	0.551261025222437	1	5	Gas7
**0.00355008348682414**	0.284819710531035	1	5	Stk40
**0.00357191819441261**	-0.288571497553352	1	5	Rbp1
**0.00359641876399269**	0.490193999216311	1	5	Nop58
**0.00359980301012757**	-0.613593752977786	1	5	Coq2
**0.00366175446376868**	-0.555400878758155	1	5	0610012G03I
**0.00371942723365678**	0.703395569072918	1	5	Fam102a
**0.00374519452830272**	-1.09994386	1	5	Atp1b2
**0.00375389865388737**	-1.202205239	1	5	Kcnab1
**0.00376816268106967**	-1.11481584	1	5	Tnc
**0.00377099218904539**	-0.372845078986235	1	5	Cd9
**0.00377655532457994**	0.697265505	1	5	Cxcl10
**0.0037858860893543**	0.732492184589548	1	5	Ttc7
**0.00378968791968482**	0.603743494915423	1	5	Gnl3
**0.00382072076261409**	-0.645866637927714	1	5	Rab11a
**0.00382665760611606**	0.322686444752224	1	5	Ssr2
**0.00382703658898017**	-0.751988290952882	1	5	Rasal2
**0.00386429591410728**	-0.413627072706469	1	5	Scp2
**0.00386909499084658**	0.516822643238365	1	5	Ism1
**0.00389132350352105**	-0.451491117636394	1	5	Psmb6
**0.00389598961893779**	-0.675502563836341	1	5	Smim12
**0.0039017644908746**	0.763320258785567	1	5	Man1a
**0.00391012246480614**	0.419065043818435	1	5	Dlc1
**0.00393082832558432**	-0.679529138179017	1	5	Rsu1
**0.00393229307488721**	-0.700405543010268	1	5	Dusp6
**0.00393512599402072**	0.349783942007243	1	5	Zhx2
**0.00397328728428927**	0.812491750511126	1	5	Taf4b
**0.00403132056674465**	-0.777566218519326	1	5	Nat6
**0.0040411515153139**	0.358548224406489	1	5	Sri
**0.00404607551365612**	-0.705880595697622	1	5	Ankrd46
**0.00404880991981383**	-0.273385599213068	1	5	Fbln5
**0.00405455849299423**	-1.027114922	1	5	Etl4
**0.00405836714361927**	0.527239958080464	1	5	Endou
**0.00407345214225742**	-0.396561605603551	1	5	Ddx42
**0.00408678767981566**	0.308196400439732	1	5	Gpx7
**0.00410802991715268**	-0.455386995301573	1	5	Pomp
**0.00414134073711026**	0.480507461302474	1	5	Hilpda
**0.0041560859227025**	-1.375305632	1	5	Rgs7bp
**0.00418110602209208**	-0.790885576589822	1	5	Rbm10
**0.00420286984818383**	-0.652407541750947	1	5	Cdc37l1
**0.00420905650538915**	-0.402528994717713	1	5	Banf1
**0.00422797924739832**	-0.508239946789849	1	5	Esyt2
**0.00424110552379437**	0.612918905	1	5	Hebp2
**0.00427120879289165**	0.312192083268149	1	5	Ergic3
**0.00427806199000769**	-0.371126611	1	5	Xpr1
**0.00435405296415628**	0.266451179103272	1	5	Dnm1
**0.00436535664824183**	0.825650760484781	1	5	Fosl1
**0.00440901324289013**	-0.698767760621827	1	5	Gdi1
**0.00442851069486497**	0.291671861227663	1	5	Tbc1d2b
**0.00446775632762098**	-0.924741555193669	1	5	Acsl1
**0.00447814533068764**	-0.560328329341552	1	5	Hras
**0.00447868169420124**	0.586489552269447	1	5	Tle3
**0.00450588775614423**	0.404772417028608	1	5	Gstm5

**0.00453842648629525**	-0.617541748785313	1	5	Smad4
**0.00459397624863355**	0.357338585970998	1	5	Arf6
**0.00461067982358625**	0.401834636795964	1	5	Tspan31
**0.00462158530465966**	-0.688769143680513	1	5	Cds2
**0.00463635576120066**	-0.616647325593666	1	5	Ptk2
**0.00472521504136374**	-0.293369060918525	1	5	Prrc2b
**0.00472957260401672**	0.295787535810124	1	5	Nr2f1
**0.0047477821455608**	-1.027650485	1	5	Spint2
**0.00474855956994401**	0.307527961865731	1	5	Vasn
**0.00478517266813958**	0.313038010286427	1	5	Cd44
**0.00478601471708904**	0.446336297737078	1	5	Papss1
**0.00487098342644571**	-1.049089648	1	5	Edn1
**0.00490720293554219**	0.343668013	1	5	Magt1
**0.00492274360363838**	0.278659280003006	1	5	Sh3gl1
**0.00494065623447937**	1.054325638	1	5	Eml4
**0.00494387148343649**	0.442036595942021	1	5	Aqr
**0.00500051013503409**	-0.659700363462022	1	5	Ehbp1l1
**0.00502736150237154**	0.311675014	1	5	Loxl1
**0.00512109761956395**	-0.497450963636604	1	5	Utp11l
**0.00516187217066221**	-0.738124123	1	5	Zkscan17
**0.00517594963164086**	-0.622367380662629	1	5	Emc8
**0.00518960020420892**	-0.761413069227955	1	5	Pex19
**0.00520720479103227**	-2.049875179	1	5	Egfl7
**0.00521644839083458**	0.340104729	1	5	Traf3
**0.0052187830832556**	-0.468393767	1	5	Dynlt3
**0.00523342412780107**	-0.485622465971761	1	5	Prkacb
**0.00527063086260395**	0.377946707357183	1	5	Vat1
**0.00528399119574125**	-0.480237619591334	1	5	Ctsc
**0.00529261147425227**	-0.974960271536638	1	5	Osbpl3
**0.00531826565398667**	0.683073409435386	1	5	Plaur
**0.0053337097330198**	-1.506150587	1	5	Vwf
**0.00533797518453853**	0.451281584682039	1	5	Lhfpl2
**0.00534227976012427**	0.279774027794945	1	5	Ewsr1
**0.00536191512604209**	0.261699211283365	1	5	Ecm2
**0.00537976079690468**	-0.823095206366672	1	5	Itih2
**0.00538805259664488**	0.271510209260331	1	5	Prdx5
**0.00543905544803026**	-0.532537489808698	1	5	Epn2
**0.00543999792632012**	0.319260136433329	1	5	Il6st
**0.00544783973142234**	-1.072946305	1	5	Dsp
**0.00544907030963862**	-1.165088923	1	5	Serpini1
**0.00546116883878711**	-0.652256199112686	1	5	Zfyve16
**0.0054768584079625**	-0.893903879032255	1	5	Camk2g
**0.00547775236513029**	-1.023534942	1	5	Mkl2
**0.0054957101922482**	-1.010943917	1	5	Hey2
**0.00549602320855487**	-0.822038430569496	1	5	Pak2
**0.00552535019272375**	-0.702300366267205	1	5	Keap1
**0.00555637200587274**	0.481242283632737	1	5	Antxr2
**0.00558094270744721**	0.589010631	1	5	Rab43
**0.005613765241292**	0.630987009	1	5	Gprc5b
**0.00561453056123874**	0.462782405324785	1	5	Man2a1
**0.00561828195416779**	-0.853473283940108	1	5	Tmem164
**0.0056323620309048**	-0.794085282	1	5	Dennd5b
**0.00564506402385793**	-1.617788657	1	5	Stab1
**0.00571206156180063**	-0.598725040393312	1	5	Ndufs2
**0.0057214635802511**	-0.401254876035301	1	5	Gm20300
**0.00583094450740648**	-0.637556073776821	1	5	Gid4

**0.00588643956706239**	1.137211101	1	5	Irs2
**0.00591549502122768**	-0.305933373703108	1	5	Chrac1
**0.00592293182746081**	0.320191732388674	1	5	Mybbp1a
**0.00595956206454881**	-0.692984850802921	1	5	Brwd1
**0.00600124591203333**	-0.978711486003197	1	5	Tef
**0.00601351284976531**	-0.712591734651235	1	5	Gucy1a3
**0.00601850679100513**	0.500562890128124	1	5	Zfp217
**0.0060480785237075**	-0.616416844104358	1	5	Arl2
**0.006050531004958**	0.413481706839014	1	5	Appl2
**0.00605142405643944**	-0.488500476565467	1	5	Rexo2
**0.00609967301682273**	-0.514713653547215	1	5	Acadm
**0.00610184550107202**	-0.958466022073345	1	5	Imp3
**0.00617038488980069**	-0.383775362	1	5	Zfp318
**0.00617156717160513**	-1.31291534	1	5	Bpgm
**0.00619391976650937**	0.825878369453557	1	5	Gadd45a
**0.00629428254447158**	-0.426438782428074	1	5	Atpif1
**0.00637277728876047**	-0.650197743	1	5	Hnrnpl
**0.00641489478745356**	0.268067221631495	1	5	Mcm6
**0.00651791091055088**	0.352130734570279	1	5	Angptl4
**0.00654164556578862**	-1.342205757	1	5	Sept6
**0.00654291579591653**	0.348056133117313	1	5	Gas1
**0.00654920121008113**	0.308714886542786	1	5	Scarb2
**0.00655754719438258**	-0.623431776718415	1	5	Sdc1
**0.00655797213318879**	-1.309308329	1	5	Pde3a
**0.00657885692014109**	0.501679163367057	1	5	Psme2b
**0.00662658990723026**	-0.817025722161665	1	5	Unc5b
**0.00667029003645534**	-0.252407428412293	1	5	Nupr1
**0.00673826904829504**	0.642466059387503	1	5	Dgat1
**0.00675129988580587**	0.677329841423669	1	5	Csf2rb2
**0.0068004888310053**	-0.734657679728324	1	5	Myo1d
**0.0068156313987965**	-1.032156512	1	5	Akt3
**0.00685978981051664**	0.948283717485594	1	5	E330033B04R
**0.00696649018106317**	-1.304162691	1	5	Fli1
**0.00704901888065073**	-0.391446504936781	1	5	Gtf3c6
**0.00707625551044821**	-0.359936028	1	5	Efna5
**0.00707832175000368**	0.373594154628945	1	5	Prnp
**0.00709557237202966**	0.638186393649523	1	5	Antxr1
**0.00710670020295842**	-0.422428579175063	1	5	Clk4
**0.00710736092245549**	-0.482314102220765	1	5	Mlf2
**0.00712242986976204**	-0.833882479712086	1	5	S1pr1
**0.00715343571266913**	0.468963663899081	1	5	Rnf19b
**0.00718535229076322**	-0.342348720258813	1	5	Pitrm1
**0.00723597463906968**	-0.492285318839241	1	5	Hint2
**0.007247045217353**	-1.352446727	1	5	Myocd
**0.00726278567419367**	-0.820400342675633	1	5	Cep170b
**0.00727254983571688**	0.729309776935873	1	5	Myd88
**0.00729501080479444**	0.471665364699916	1	5	Pid1
**0.00735099728133963**	-0.335868578813967	1	5	Gtf2e2
**0.00736698239356465**	0.650598237840793	1	5	Abca6
**0.00737446133567412**	0.662231021468868	1	5	Acly
**0.0074468689288781**	0.363285834186465	1	5	Lonrf3
**0.00745208315293299**	-0.440259363111592	1	5	Eno1b
**0.00749181791196859**	0.415796113107639	1	5	Glul
**0.00752843446694476**	0.518606416314489	1	5	Ubfd1
**0.00755764965309855**	-0.710912163091291	1	5	Adam10
**0.00768643551132864**	0.616739292749084	1	5	Rnh1

**0.00769614331443865**	0.622243647010905	1	5	Ibtk
**0.00773786608343661**	0.560932567291662	1	5	5031426D15I
**0.00774631543907339**	0.441932091140789	1	5	Os9
**0.00781769638515446**	-0.563337497549944	1	5	Phc3
**0.00784764099251302**	0.457233548192707	1	5	Arl5b
**0.0079569330942229**	-0.871235079219542	1	5	Zfp467
**0.00796138535301499**	-0.433244051	1	5	Brcc3
**0.00796429224059076**	-1.070252179	1	5	Pawr
**0.00797017679011027**	-0.538791130295039	1	5	Ezh1
**0.00805689118736812**	-0.51700992	1	5	Cnn2
**0.00809220010099469**	0.329053216941098	1	5	Eif5
**0.00820530192638482**	-0.743565368091351	1	5	Poldip2
**0.00822557567524235**	-1.305972443	1	5	Adcy4
**0.00822887556051268**	0.366604448421533	1	5	Mmp3
**0.00834204984558976**	0.292549709873304	1	5	Atp2b1
**0.00841285248911352**	-0.613908820678491	1	5	Dock10
**0.00845193568162557**	0.507468218898625	1	5	Coq10b
**0.00845238436879142**	0.334775996	1	5	Dnajb1
**0.00853637876715864**	0.370374424404728	1	5	Phrf1
**0.00857174907569224**	0.653241672051542	1	5	Sirpa
**0.0085988823944592**	0.298800166933054	1	5	Ppp1r14b
**0.00862466137868416**	0.352002614728775	1	5	Akt2
**0.00863218660923475**	-0.689110782230254	1	5	Zc3h14
**0.00863517601332922**	-0.813950749963108	1	5	Cx3cl1
**0.00871223912543574**	0.395317878238018	1	5	Plbd2
**0.00882495615234257**	-0.811681219922664	1	5	Plvap
**0.00888624352467449**	0.388939743786034	1	5	H2-T23
**0.00888680720224122**	-0.442109447396182	1	5	Tmem87b
**0.00898388991249196**	0.484588965	1	5	Negr1
**0.00900710126753423**	0.858110812643052	1	5	Fam46c
**0.00905554816848252**	-0.624719736782707	1	5	Agtpbp1
**0.00907673004876003**	-1.238954781	1	5	Arl16
**0.00935162515302028**	-0.563627473756869	1	5	Zcchc8
**0.00937443101505707**	0.665208870095198	1	5	Cmtm3
**0.00941331993804875**	-0.547750965480309	1	5	Txnip
**0.00943601459768289**	0.266076135354469	1	5	Tubb2a
**0.009585488**	0.622007975391022	1	5	Sphk1
**0.00964972416841405**	-1.195392615	1	5	Nhsl2
**0.00970699590774138**	0.546227295849797	1	5	Mme
**0.00974275600415371**	-1.040674452	1	5	Ldb3
**0.00975082618646903**	-0.434628794931147	1	5	Dcaf8
**0.00987799397216483**	0.879042759551186	1	5	Ormdl3
**0.0099202164147301**	0.278733304268023	1	5	Nucb2
**0.00997779918823177**	0.739761021094038	1	5	Cxcl5

p_val	avg_logFC	p_val_adj	cluster	gene
**3.07779105716802e-45**	1.56	4.25073722905475e-41	6	Ppp1r14a
**9.61207310769564e-37**	0.928660812710678	1.32752341690384e-32	6	Acta1
**5.82870912487804e-33**	0.417719521834501	8.05003017236906e-29	6	Myl6b
**1.04196632738366e-30**	1.79	1.43905969474957e-26	6	Ramp1
**9.05705573569011e-30**	0.375826240331055	1.25086996765616e-25	6	Actbl2
**1.07766824511582e-29**	0.896594269001084	1.48836761332946e-25	6	Tmsb4x
**2.33202004060107e-28**	-2.58	3.22075287807414e-24	6	Ly6e
**5.56014076830855e-27**	1.62	7.67911041511094e-23	6	Asb2
**9.19926646755711e-27**	1.26	1.27051069183431e-22	6	Tpm2
**1.05828457055559e-26**	1.37	1.46159682039433e-22	6	Cnn1
**1.75003172005933e-26**	0.418223613886452	2.41696880857394e-22	6	Actc1
**3.39606387139443e-26**	-1.98	4.69030381278285e-22	6	Dcn
**3.63236393477053e-26**	1.02	5.01665783031158e-22	6	Dstn
**4.84608041910274e-26**	1.52	6.6929216668228e-22	6	Efhd1
**9.80831871479213e-26**	1.47	1.35462689769994e-21	6	Sh3bgr
**1.09481731344282e-25**	1.15	1.51205219159588e-21	6	Myl9
**1.28721728028138e-25**	1.18	1.77777578579661e-21	6	Itga8
**1.512962580186E-25**	-2.53	2.08955261949488e-21	6	Cd34
**1.85897553989903e-25**	1.19	2.56743111815456e-21	6	Tagln
**3.17833520993362e-25**	0.838659429258325	4.38959875843932e-21	6	Flna
**5.35117045246583e-25**	1.92	7.39050151190056e-21	6	E030013I19R
**1.53797477039881e-24**	0.976482293226515	2.12409695539779e-20	6	Myh11
**1.62579451218083e-24**	1.04	2.24538480077294e-20	6	Csrp2
**2.25029295694877e-24**	1.06	3.10787960284195e-20	6	Acta2
**2.47025373580012e-24**	1.08	3.41166743451354e-20	6	Smtn
**3.65686056093275e-24**	1.59	5.05049012070422e-20	6	Kcnmb1
**6.17174205856543e-24**	1.35	8.52379295708472e-20	6	Cbr2
**7.876210124271 9e-24**	1.29	1.08778338026319e-19	6	Tspan2
**2.7976143320817e-23**	-2.64	3.86378515403803e-19	6	Serpinf1
**3.08245246404735e-23**	1.09	4.2571750980958e-19	6	Mylk
**3.13961703219845e-23**	0.97	4.33612508316928e-19	6	Rcan2
**3.63291459289892e-23**	2.04	5.0174183442527e-19	6	1190005I06R
**5.85487339538848e-23**	1.21	8.08616564637102e-19	6	Gm13889
**6.57693200766278e-23**	1.50	9.08340079578307e-19	6	Mustn1
**9.70921006065979e-23**	1.08	1.34093900147772e-18	6	Actn1
**1.03012608050207e-22**	-2.12	1.42270712978141e-18	6	Igfbp4
**1.11397206616153e-22**	1.06	1.53850682057569e-18	6	Col18a1
**1.348884532359E-22**	-2.06	1.86294442764101e-18	6	Lum
**1.3644127551266e-22**	1.07	1.88439045610535e-18	6	Synpo2
**3.09016869640454e-22**	1.28	4.26783198660431e-18	6	Npnt
**3.48348229951987e-22**	1.33	4.8110374038669e-18	6	Hspb7
**4.11710808676766e-22**	0.855184669275052	5.68613797863482e-18	6	Tpm1
**4.78553203297101e-22**	0.868670994212233	6.60929829073626e-18	6	Sost
**5.18120974379144e-22**	-2.28	7.15576877715035e-18	6	Icam1
**5.77759671273113e-22**	0.849454472993801	7.97943881995296e-18	6	Pls3
**8.50434964915796e-22**	1.69	1.17453573004521e-17	6	Sgcg
**2.42253716635569e-21**	-1.90	3.34576608045385e-17	6	Mmp2
**2.43321688589974e-21**	1.06	3.36051584111613e-17	6	Mcam
**2.9204857224524e-21**	-2.38	4.03348283127901e-17	6	Fbln2
**3.11586245941041e-21**	-2.80	4.30331764269171e-17	6	Abca8a
**3.44378769192933e-21**	-2.58	4.7562151813236e-17	6	Dpt
**3.59695662992978e-21**	1.44	4.96775680159601e-17	6	Hspb6
**4.7033905392429e-21**	1.31	6.49585267374837e-17	6	Susd5
**5.09126128793609e-21**	-2.09	7.03154096476853e-17	6	Ly6a
**6.52929094681748e-21**	-2.21	9.01760372664962e-17	6	Emp1

**1.84610944761628e-20**	1.17	2.54966175810284e-16	6	Mylk4
**1.93600062803281e-20**	-1.93	2.67381046737612e-16	6	C1ra
**1.94842360222274e-20**	-2.17	2.69096783702983e-16	6	Vcam1
**1.98036138708898e-20**	1.11	2.73507711170859e-16	6	Dmpk
**3.11102835978469e-20**	-2.30	4.29664126769863e-16	6	Pdgfra
**3.44778814406993e-20**	-1.20	4.76174020577499e-16	6	Cfh
**4.66424998255893e-20**	-2.72	6.44179565091213e-16	6	Cpxm1
**6.60423040343814e-20**	1.13	9.12110261018841e-16	6	Nov
**8.23431531123112e-20**	-2.81	1.13724128763413e-15	6	Smoc2
**8.88951661166331e-20**	1.04	1.22773113923682e-15	6	Pdlim3
**9.10659032447378e-20**	1.07	1.25771118971307e-15	6	Ccdc3
**9.5366320100264e-20**	0.875341867243604	1.31710424690475e-15	6	Ncam1
**9.80793241601026e-20**	0.882864743272134	1.35457354597518e-15	6	Myl6
**1.37456506537047e-19**	0.922212248647594	1.89841181178316e-15	6	Dmd
**1.49576941260762e-19**	-2.36	2.06580713575239e-15	6	Cygb
**1.75676941745847e-19**	0.80	2.42627424245189e-15	6	Csrp1
**1.75712354953327e-19**	0.548530687731948	2.4267633342604e-15	6	Lgals1
**1.77064024321076e-19**	0.571183737359971	2.44543123989838e-15	6	Cald1
**1.88003772975917e-19**	-1.82	2.59652010857039e-15	6	Cp
**2.03577105257377e-19**	-2.54	2.81160340070964e-15	6	Entpd2
**2.12554723306717e-19**	1.48	2.93559328358906e-15	6	Pcp4l1
**2.18720549190913e-19**	0.668257954215111	3.0207495048757e-15	6	Myom1
**2.19264474294101e-19**	1.49	3.02826165447583e-15	6	Ptpla
**2.60177106008418e-19**	-2.18	3.59330601108227e-15	6	Add3
**2.67301882744402e-19**	-1.75	3.69170630258294e-15	6	Slc43a3
**2.68290819592624e-19**	-2.21	3.70536450939373e-15	6	Pcolce
**3.76589937493107e-19**	1.67	5.20108362671731e-15	6	Actg2
**4.207197534256E-19**	1.09	5.81056051456096e-15	6	Art3
**4.5348486189319e-19**	-2.25	6.26307942760684e-15	6	Ackr3
**4.87560922535728e-19**	0.93	6.73370390114093e-15	6	Lmod1
**5.111117444227E-19**	-1.96	7.05896430222191e-15	6	C3
**6.05812450956218e-19**	0.675635933289407	8.36687576015633e-15	6	Lpp
**9.55190857781014e-19**	1.34	1.31921409368136e-14	6	Aoc3
**1.41864272198854e-18**	-2.19	1.95928746333838e-14	6	Fbln1
**1.51566549163878e-18**	1.54	2.09328561050232e-14	6	Hspb2
**1.58588585288379e-18**	1.44	2.1902669514178e-14	6	Fgf1
**2.58979697286331e-18**	-2.64	3.57676859922152e-14	6	Tgfbi
**3.21999223785832e-18**	-2.19	4.44713127970613e-14	6	Cxcl1
**4.64587705949159e-18**	1.16	6.41642080686384e-14	6	Pdlim1
**5.01288002882412e-18**	0.901733812087025	6.923288607809E-14	6	Tns1
**6.30134023457583e-18**	1.36	8.70278099797268e-14	6	Map3k7cl
**8.80355326493606e-18**	-2.56	1.21585874142032e-13	6	Il33
**1.03306026949887e-17**	-2.19	1.42675953820489e-13	6	Serpina3n
**1.07628035043713e-17**	1.34	1.48645079198872e-13	6	Spint2
**1.10640119072781e-17**	0.414243194885015	1.52805068451418e-13	6	NP-904328.1
**1.228966136059E-17**	-2.44	1.69732513051109e-13	6	Sfrp1
**2.874567303568E-17**	0.98	3.97006490295776e-13	6	Postn
**2.8922757607856e-17**	2.02	3.99452205322099e-13	6	Slc22a1
**3.81516546025949e-17**	0.897460251690616	5.26912501716439e-13	6	Ppp1cb
**3.99285592310945e-17**	1.19	5.51453331540645e-13	6	Lims2
**4.39480233411666e-17**	0.69	6.06966150364852e-13	6	Serpine2
**6.0307726740523e-17**	0.361422147712788	8.32910014013363e-13	6	Vim
**7.1337061309166e-17**	-2.22	9.85236153740891e-13	6	Gpx3
**8.80925215095095e-17**	0.879383600774532	1.21664581456784e-12	6	Plekho1
**8.96534972216865e-17**	1.87	1.23820445012871e-12	6	Rasgrp2
**1.03347033160343e-16**	0.756095662458108	1.4273258749775e-12	6	Enah

**1.07071165165825e-16**	-1.60	1.47875986210521e-12	6	Bhlhe40
**1.13718214883564e-16**	-1.80	1.5705622657569e-12	6	Col14a1
**1.14100866501095e-16**	0.401482722594687	1.57584706724663e-12	6	Fth1
**1.19844936794471e-16**	1.24	1.65517842206844e-12	6	Rasl12
**1.58256042642773e-16**	0.372662907388705	2.18567420493934e-12	6	NP-904340.1
**1.90712483153094e-16**	1.14	2.63393010482738e-12	6	Pgm5
**1.90759702996947e-16**	-1.45	2.63458225809083e-12	6	Rnase4
**2.7144143597719e-16**	-1.50	3.74887767228097e-12	6	Serping1
**3.08439171162607e-16**	-2.51	4.25985339292676e-12	6	Dpep1
**3.14838980691261e-16**	0.707125256853968	4.34824116232701e-12	6	Oxct1
**3.4454667053441e-16**	0.812793637131805	4.75853406675073e-12	6	Pbxip1
**4.41858132932701e-16**	-2.51	6.10250267393354e-12	6	Cfb
**4.49759421112614e-16**	0.770801018746929	6.21162736498632e-12	6	Ptgis
**4.65166496377936e-16**	0.639741116338547	6.42441448147568e-12	6	Bcam
**6.35926399411982e-16**	1.10	8.78277950227888e-12	6	Thsd4
**6.78530545043949e-16**	0.742777019953332	9.37118535760197e-12	6	Cox6c
**7.0030017420671e-16**	-2.14	9.67184570596888e-12	6	Marcks
**7.34494992614337e-16**	-1.84	1.01441103429966e-11	6	Tmem176b
**8.06754514212056e-16**	-2.26	1.11420865957827e-11	6	C4b
**8.46450647557204e-16**	0.628666320678509	1.16903298934125e-11	6	Map1b
**8.48808842216681e-16**	0.966655732832878	1.17228989198546e-11	6	Cryab
**8.85542045566649e-16**	0.800573324605422	1.2230221191321e-11	6	Slmap
**9.71989553932655e-16**	0.567018289016532	1.34241477293639e-11	6	Kcnab1
**9.98972510516768e-16**	-2.41	1.37968093427471e-11	6	Igf1
**1.10277955130989e-15**	1.25	1.52304883831409e-11	6	Serpini1
**1.15467776304387e-15**	0.98	1.59472545853989e-11	6	Higd1a
**1.19606343137175e-15**	1.27	1.65188320506753e-11	6	Gucy1b3
**1.37563032078714e-15**	-1.55	1.89988303603912e-11	6	Mgst1
**1.59812354348942e-15**	1.55	2.20716842591324e-11	6	Glipr1
**2.01458752535865e-15**	0.490230135208725	2.78234683127284e-11	6	Itgb1
**2.14614623538767e-15**	1.31	2.96404256569391e-11	6	Fbxo30
**2.20237544868794e-15**	1.47	3.04170073218291e-11	6	Cap2
**2.22199076431092e-15**	-2.41	3.06879144458981e-11	6	Ramp2
**2.63057297747304e-15**	-2.53	3.63308433918801e-11	6	Gda
**2.64673638201313e-15**	0.557820193239123	3.65540761719833e-11	6	Cpe
**3.00557739146514e-15**	0.701840360995886	4.1510029353525e-11	6	Sh3bgrl
**3.50738439945304e-15**	-1.28	4.8440485940846e-11	6	Gsn
**3.94589352190794e-15**	-2.37	5.44967354310706e-11	6	Scn7a
**3.99520921542381e-15**	-2.39	5.51778344742182e-11	6	Pi16
**4.27860597624011e-15**	-0.926124189057958	5.90918271378522e-11	6	Ifitm3
**5.12743157052863e-15**	1.20	7.0814957420571e-11	6	6330403A02R
**5.58686052844066e-15**	-1.78	7.7160130758294e-11	6	Nid1
**5.8247928226594e-15**	0.725959983908335	8.04462136737489e-11	6	Slc25a4
**7.93732522821363e-15**	0.81	1.09622398726858e-10	6	Vcl
**1.10136544963477e-14**	-2.49	1.52109582249058e-10	6	Efemp1
**1.44646979631519e-14**	1.03	1.99771943569091e-10	6	Samd4
**1.53081779000875e-14**	0.89	2.11421244978109e-10	6	Msrb1
**1.87583491623716e-14**	-2.40	2.59071560281515e-10	6	Bmp4
**2.23317630221118e-14**	1.12	3.08423979098386e-10	6	Cspg4
**2.45949069536119e-14**	-1.96	3.39680259936334e-10	6	Pltp
**2.91563611416851e-14**	-1.76	4.02678503727813e-10	6	C1s1
**2.9537442748211e-14**	0.954602925175644	4.07941621795543e-10	6	Pdcl3
**5.03054365738831e-14**	-1.65	6.947683845219E-10	6	Lima1
**5.07870891141465e-14**	-2.11	7.01420487755477e-10	6	Bicc1
**6.24126911282973e-14**	1.06	8.61981677172914e-10	6	Perp
**6.45601674335089e-14**	2.00	8.91640472424191e-10	6	Fbxl22

**6.9141700147596e-14**	-2.19	9.54916020738449e-10	6	Clec3b
**7.15388831766338e-14**	-1.39	9.88023515552489e-10	6	Adamts2
**7.79677329856956e-14**	-1.45	1.07681236026544e-09	6	Meg3
**8.06823819956257e-14**	0.952111066319227	1.11430437774159e-09	6	Kank1
**8.25211145784155e-14**	0.363220926375075	1.1396991134425e-09	6	NP-904329.1
**9.36046719477239e-14**	0.859242180920342	1.29277412427002e-09	6	Entpd1
**9.50449449870948e-14**	-1.58	1.31266573521677e-09	6	Litaf
**9.69577972723009e-14**	-1.87	1.33908413812775e-09	6	Mmp3
**9.8812044019201e-14**	0.597922198447254	1.36469313994919e-09	6	Rock1
**1.13768091300427e-13**	0.659475140390087	1.5712511089502e-09	6	Myh10
**1.25577810456842e-13**	0.728559269227465	1.73435514021945e-09	6	Pkp4
**1.32356014901069e-13**	0.530039066137373	1.82796892179866e-09	6	Fblim1
**1.40690234410715e-13**	0.546804985127935	1.94307282744639e-09	6	Aplp2
**1.50544326739981e-13**	0.525085684553908	2.07916769660587e-09	6	Cox4i1
**1.61793523485187e-13**	0.844635008424626	2.23453035285391e-09	6	Nox4
**1.83323113116741e-13**	0.775715338239421	2.53187551525531e-09	6	Rbpms
**1.85018419861846e-13**	-1.45	2.55528939671195e-09	6	Ablim1
**2.5141947146326e-13**	-1.24	3.47235432037909e-09	6	Nrp1
**2.68875003605126e-13**	-1.97	3.71343267479039e-09	6	Gfpt2
**2.72952738695183e-13**	0.698034888355654	3.76975027411917e-09	6	Itga9
**2.81643290402805e-13**	-2.20	3.88977548375313e-09	6	C7
**3.09742278003784e-13**	-2.06	4.27785060151027e-09	6	Ifi27l2a
**3.7099744391087e-13**	-1.78	5.12384569785303e-09	6	Olfml3
**3.82497606992401e-13**	1.48	5.28267445017205e-09	6	Anln
**3.95532511211491e-13**	-2.35	5.46269951234190e-09	6	Dio2
**4.20944790911688e-13**	1.70	5.81366850728132e-09	6	Gm11627
**4.29911204153001e-13**	-2.28	5.9375036405571e-09	6	Fxyd6
**4.41283368296358e-13**	0.868464181484983	6.09456459954099e-09	6	Mrvi1
**4.4768304866315e-13**	-1.92	6.18295058508676e-09	6	Thbd
**4.54903904011308e-13**	0.782057338518803	6.28267781830017e-09	6	Cped1
**4.5930405545881e-13**	1.04	6.34344830994163e-09	6	Itih4
**5.287620866754E-13**	0.390358509992695	7.30273317907395e-09	6	Igfbp7
**5.44696306637386e-13**	0.607853650728102	7.52280069096894e-09	6	Pfn1
**5.93422043155943e-13**	0.379877501741296	8.19575183802673e-09	6	NP-904337.1
**6.26328023896515e-13**	-2.33	8.65021633803476e-09	6	Ctsk
**6.3453477651413e-13**	0.697712388157855	8.76355979843665e-09	6	Nexn
**7.57747757219949e-13**	0.261122417752583	1.04652542749647e-08	6	Actb
**7.8313582697623e-13**	0.711808852823328	1.08158889063687e-08	6	Ndufa6
**9.3781014554039e-13**	0.872784793513503	1.29520959200583e-08	6	2310022B05R
**9.42820158741214e-13**	-2.63	1.30212892123749e-08	6	Scara5
**1.04924082937203e-12**	0.411452095528131	1.44910650944572e-08	6	Fbxo32
**1.10214223936659e-12**	0.696692886586295	1.5221686467892e-08	6	Uqcr10
**1.11862566767E-12**	0.547742690921083	1.54493390961904e-08	6	Ogn
**1.17026352635026e-12**	0.691701658324571	1.61625095624234e-08	6	Fxyd1
**1.26458101970821e-12**	0.497143160281753	1.746512846319E-08	6	Fhl1
**1.27359797145597e-12**	1.15	1.75896615837784e-08	6	Suclg2
**1.33109112132161e-12**	1.23	1.83836994765727e-08	6	Acsl1
**1.40748758669645e-12**	1.32	1.94388110598647e-08	6	Kcnh2
**1.69674607972955e-12**	-1.72	2.34337601071448e-08	6	Lpl
**1.77442240334892e-12**	0.742144246451178	2.45065478126519e-08	6	Lrrfip1
**1.85793251183889e-12**	0.849535631904789	2.5659905921007e-08	6	Dsp
**2.30442308847862e-12**	-1.53	3.18263872749782e-08	6	Timp1
**2.42954246728065e-12**	-1.68	3.3554411015613e-08	6	Phlda1
**2.44473579419895e-12**	-1.75	3.37642460536817e-08	6	Clec11a
**2.47064752215341e-12**	0.604475447988136	3.41221129284608e-08	6	Zyx
**2.52326487767696e-12**	-2.00	3.48488112255965e-08	6	Akap12

**2.58121582962519e-12**	-1.23	3.56491718229535e-08	6	Ppap2b
**2.8069627006394e-12**	0.447448795763079	3.87669618585307e-08	6	Ptrf
**2.83939504472753e-12**	0.683958841893197	3.92148849627319e-08	6	Cav1
**2.97079869711819e-12**	1.10	4.10297008058994e-08	6	1700120C14I
**3.13025476924415e-12**	0.845473682515187	4.3231948618031e-08	6	Rras
**3.40907132694961e-12**	-2.18	4.70826840965011e-08	6	Bace2
**3.52680783891519e-12**	0.835133597962525	4.87087430632578e-08	6	Itga3
**3.57946050231864e-12**	0.847241842237309	4.94359289975228e-08	6	Zak
**3.60872159013456e-12**	0.549910599350201	4.98400538813485e-08	6	Ppp1r12a
**3.79656207190724e-12**	-0.945891750294651	5.24343187751109e-08	6	H2-D1
**4.48169130876015e-12**	0.796674044806344	6.18966386652864e-08	6	Msrb3
**4.4950044384205e-12**	0.934551931851081	6.20805062990255e-08	6	Usmg5
**4.8841669844909e-12**	0.851170670163703	6.74552302228038e-08	6	Marveld1
**4.98750579202825e-12**	-1.90	6.88824424937022e-08	6	Lgals9
**5.057132790535E-12**	-2.00	6.98440609700789e-08	6	Lpar1
**5.07389602031271e-12**	-1.30	7.00755779365388e-08	6	Cyp1b1
**5.17541406925348e-12**	1.08	7.14776437104599e-08	6	Stk38l
**5.23105285563823e-12**	-2.18	7.22460709892196e-08	6	Acvrl1
**5.42751043681453e-12**	-1.94	7.49593466428454e-08	6	Spon1
**5.97673291753156e-12**	0.758779653649457	8.25446583240284e-08	6	Mark1
**6.07556866980645e-12**	-1.25	8.39096788986969e-08	6	Cxcl12
**6.42015076423953e-12**	0.70	8.86687022049122e-08	6	Atp5j2
**6.43754399614123e-12**	-1.00	8.89089201307065e-08	6	Ccdc80
**6.83600254333613e-12**	0.733505297936637	9.44120311260153e-08	6	Uqcrb
**6.84026646883104e-12**	0.796420242519164	9.44709202010254e-08	6	Tmem38b
**7.38344995639512e-12**	-2.03	1.01972827347773e-07	6	Dnm1
**8.21290129111223e-12**	0.865722906363164	1.13428379731551e-07	6	Hcfc1r1
**8.54847716583562e-12**	0.966422679037745	1.18063018137356e-07	6	Rassf3
**9.7714019554846e-12**	0.930872483422915	1.34952832407198e-07	6	Rbpms2
**1.06252565279143e-11**	-2.01	1.46745417907025e-07	6	Ly6c1
**1.06984315924365e-11**	-1.73	1.47756038723141e-07	6	Kcnq1ot1
**1.18381900527143e-11**	-1.53	1.63497242818038e-07	6	Irf1
**1.24792811268581e-11**	-1.65	1.72351351643037e-07	6	Lgals3bp
**1.25780078201293e-11**	-2.06	1.73714866003806e-07	6	Comp
**1.30219572590447e-11**	-2.36	1.79846251704666e-07	6	Igfbp6
**1.35985299066503e-11**	-1.75	1.87809296540748e-07	6	Slfn5
**1.37391712740522e-11**	-2.14	1.89751694465934e-07	6	Tnfaip3
**1.45184146734738e-11**	-0.91	2.00513825055347e-07	6	Col1a1
**1.51096806785428e-11**	0.781477796340889	2.08679799851354e-07	6	Capzb
**1.77761436529221e-11**	0.79	2.45506319990508e-07	6	Lmcd1
**1.96813848403582e-11**	-1.34	2.71819606030187e-07	6	Cebpd
**2.23977395756427e-11**	-1.98	3.09335181279201e-07	6	Cmip
**2.54051112949087e-11**	-1.28	3.50869992093984e-07	6	Osmr
**2.67042239879717e-11**	0.909492065415077	3.68812037497878e-07	6	Adck3
**2.79459846699692e-11**	1.00	3.85961994276944e-07	6	Osbpl3
**2.80548073141663e-11**	0.780814470724216	3.87464943815951e-07	6	Rsu1
**2.88660614942104e-11**	-2.06	3.9866917529654e-07	6	Srpx
**2.92923722667788e-11**	0.900497888567831	4.04556953376482e-07	6	Plin4
**3.27205939579286e-11**	0.88	4.51904123152952e-07	6	Snhg18
**3.34621914826667e-11**	0.80	4.6214632656711e-07	6	Speg
**3.53030578807344e-11**	0.698517326284632	4.87570532390823e-07	6	Atp5g3
**3.94884057280228e-11**	-1.85	5.45374371509722e-07	6	Trf
**4.38988889709084e-11**	0.628238939804421	6.06287555577216e-07	6	Cox8a
**4.51146514362558e-11**	0.952425465830864	6.23078450986129e-07	6	Pcdh7
**4.53936286004706e-11**	-1.86	6.26931404601099e-07	6	Prg4
**4.78640900563335e-11**	0.875689537607548	6.61050947768022e-07	6	Tgfb1i1

**4.79287269018815e-11**	0.762001369770112	6.61943647241885e-07	6	Wfdc1
**4.93095153662777e-11**	0.78	6.81013716723661e-07	6	Ndufa11
**5.04405892735011e-11**	1.31	6.96634978456323e-07	6	Mmp17
**5.17871090012341e-11**	-2.28	7.15231762416044e-07	6	Aldh1a1
**5.20351357277069e-11**	-1.35	7.1865725953536e-07	6	Pcolce2
**5.21856713362691e-11**	-2.08	7.20736306825213e-07	6	Ms4a4d
**5.60408667818101e-11**	-2.15	7.73980411123579e-07	6	Gpm6b
**6.13374394276514e-11**	0.437134955498801	8.47131375935294e-07	6	S100a6
**6.33827454264991e-11**	-0.90	8.75379097085379e-07	6	Nfkbia
**6.74900552418928e-11**	-1.88	9.32105152945781e-07	6	H2-T23
**6.94408091638949e-11**	1.05	9.59047015362552e-07	6	Gpsm2
**7.0853307047605e-11**	-2.12	9.78555023634473e-07	6	Steap4
**7.49830001053725e-11**	0.66	1.0355902144553e-06	6	Dynll1
**7.59813735524237e-11**	1.56	1.04937875013252e-06	6	Kcnma1
**8.88032969449935e-11**	0.794619660226745	1.22646233410731e-06	6	Cox7c
**8.91226319670158e-11**	0.575216164550529	1.23087267009645e-06	6	Gm12338
**9.15198386409422e-11**	-1.34	1.26398049147005e-06	6	Csf1
**1.01160366953714e-10**	1.05	1.39712582799774e-06	6	Fabp4
**1.07218078939337e-10**	0.708614039038272	1.48078888823118e-06	6	Unc5c
**1.13809550514937e-10**	1.16	1.57182370216179e-06	6	Dtna
**1.16008785028278e-10**	-1.16	1.60219733002555e-06	6	Sash1
**1.17529872699714e-10**	0.869769680285986	1.62320507185576e-06	6	Hdac9
**1.27166847233981e-10**	0.539146562882773	1.75630132714851e-06	6	Cd200
**1.34190502683444e-10**	1.51	1.85330503256104e-06	6	Sytl5
**1.39744233583484e-10**	0.98	1.93000761002149e-06	6	St5
**1.40462669259147e-10**	0.389845263715908	1.93992992513808e-06	6	Nupr1
**1.40713196110334e-10**	1.00	1.94338995147982e-06	6	Fgf13
**1.41059438934078e-10**	-0.70	1.94817191111856e-06	6	Timp2
**1.45493493108751e-10**	0.837694418824198	2.00941063332496e-06	6	Pkdcc
**1.57344314477271e-10**	-1.10	2.17308232724559e-06	6	Ccnl1
**1.59941557541082e-10**	0.917649312956293	2.20895285119989e-06	6	Pde4dip
**1.70513550563736e-10**	-1.85	2.35496264683576e-06	6	Cytl1
**1.75608305532579e-10**	0.57	2.42532630771045e-06	6	Rpl7
**1.80905202682821e-10**	0.979798134000582	2.49848175425244e-06	6	Neo1
**1.94592447579312e-10**	0.531398665718754	2.68751629351788e-06	6	Rpl14-ps1
**1.96791044622357e-10**	0.68	2.71788111727937e-06	6	Anapc11
**2.17146489604538e-10**	1.14	2.99901016792827e-06	6	Bcar3
**2.18548606492412e-10**	0.694003076307039	3.01837480426671e-06	6	Hspb1
**2.24650131913742e-10**	0.571727237264887	3.10264297186069e-06	6	Atp5j
**2.26459763653894e-10**	0.678463361370673	3.12763579582393e-06	6	Tln1
**2.31898773990602e-10**	1.11	3.20275396758421e-06	6	Tnk2
**2.33393314407022e-10**	1.06	3.22339506527538e-06	6	Pawr
**2.56272793083463e-10**	-0.970942953937302	3.53938354527571e-06	6	Nav1
**2.7916498859101e-10**	0.47	3.85554765743044e-06	6	Clic4
**2.83255364577264e-10**	-2.35	3.91203984017659e-06	6	Inmt
**2.85090804913893e-10**	0.952396181130109	3.93738910666577e-06	6	Tes
**2.90824847983767e-10**	-1.67	4.01658197550381e-06	6	Medag
**3.01485842643586e-10**	0.539659215895717	4.16382097275057e-06	6	Utrn
**3.21202497897944e-10**	0.945726574891884	4.43612769846851e-06	6	Nop10
**3.27795845345243e-10**	-2.33	4.52718842006315e-06	6	Cpxm2
**3.28817824177717e-10**	1.34	4.54130296971845e-06	6	Phtf2
**3.38065188226793e-10**	-2.23	4.66901831460024e-06	6	Hsd11b1
**3.38844876886719e-10**	-1.20	4.67978659468248e-06	6	Adamts5
**3.66151890958312e-10**	-1.65	5.05692376602524e-06	6	Slc16a2
**3.79109609602425e-10**	0.48	5.23588281821909e-06	6	Calm2
**3.79131559159151e-10**	0.559198053089864	5.23618596354703e-06	6	Mfge8

**3.82474453985172e-10**	-2.44	5.2823546839892e-06	6	Fmod
**3.90548914627914e-10**	-1.47	5.39387105992612e-06	6	Uap1
**3.90993772242427e-10**	-0.969884113567996	5.40001498844016e-06	6	Tcf4
**4.00418186619934e-10**	-1.93	5.53017557540791e-06	6	Bst2
**4.05537029037683e-10**	0.497738056772211	5.60087190803944e-06	6	Net1
**4.29858150456804e-10**	0.77	5.93677091595892e-06	6	Fat1
**4.42225874688687e-10**	0.561622767189435	6.10758155532545e-06	6	Rgs4
**4.59988951556482e-10**	0.494105032313182	6.35290740994657e-06	6	Limch1
**4.90065839010754e-10**	-2.10	6.76829930257752e-06	6	Gpnmb
**4.95148789818991e-10**	1.27	6.83849993619008e-06	6	Sgca
**5.27595080919573e-10**	0.777864460258767	7.28661566258022e-06	6	Wwp2
**5.46902025781135e-10**	-2.22	7.55326387806326e-06	6	Apod
**5.70558942058401e-10**	0.40	7.87998954876858e-06	6	Mbnl1
**5.71622267156358e-10**	-1.98	7.89467513169646e-06	6	Svep1
**5.83579987780054e-10**	0.417021220406283	8.05982321123033e-06	6	Actn4
**5.96985892608132e-10**	-1.19	8.24497216281091e-06	6	Fxyd5
**6.57050044641069e-10**	0.591221139370595	9.0745181665378e-06	6	Rgs7bp
**6.91470096711702e-10**	0.834975413787392	9.54989350568532e-06	6	Col4a6
**7.17932009055906e-10**	0.335085046527152	9.91535897707111e-06	6	Gm13826
**7.29222066862334e-10**	0.751967075166303	1.00712859654357e-05	6	Mef2c
**7.35720885650112e-10**	-1.32	1.01610411517137e-05	6	Ifnar2
**7.3605186914967e-10**	-1.51	1.01656123648261e-05	6	Gas1
**7.60784574439298e-10**	0.661955556062916	1.05071957575811e-05	6	Pdlim7
**8.16863176296919e-10**	-1.08	1.12816973278368e-05	6	Stat3
**8.28357077900398e-10**	0.490484454757633	1.14404396028824e-05	6	Cox6b1
**8.4740902351893e-10**	0.759336066319671	1.17035660238199e-05	6	Sema3c
**9.19517626580689e-10**	-1.93	1.26994579407059e-05	6	Gdf10
**9.62376888537643e-10**	0.457062505412142	1.32913872075934e-05	6	Rpl14
**9.67697957649219e-10**	0.798687751426736	1.33648764930934e-05	6	Ldhb
**1.02880034422727e-09**	0.302121304901511	1.42087615541228e-05	6	Gpx1
**1.04226789646034e-09**	0.81	1.43947619180137e-05	6	Rrad
**1.06669995387534e-09**	0.648933637684937	1.47321930629723e-05	6	Mtss1l
**1.08451446002764e-09**	-1.79	1.49782292074417e-05	6	Xdh
**1.10675405147849e-09**	0.737886120205665	1.52853802049695e-05	6	Rpl10a
**1.14843986536623e-09**	0.450879966190412	1.5861102980573e-05	6	Gstm1
**1.28027568023255e-09**	-1.29	1.76818874196918e-05	6	Lama2
**1.33126524749218e-09**	-0.857899847855367	1.83861043331145e-05	6	Fuca1
**1.34291273525349e-09**	-1.29	1.85469677865859e-05	6	Jam2
**1.37953538600201e-09**	1.38	1.90527632160738e-05	6	Ldb3
**1.52980404312285e-09**	0.708245180595902	2.11281236395697e-05	6	Atp5g1
**1.58429807367471e-09**	0.608783136104533	2.18807406955214e-05	6	Ndufc1
**1.68714136970522e-09**	0.58	2.33011094569988e-05	6	Ndufa4
**1.69102273770198e-09**	-1.56	2.33547150304021e-05	6	Dab2
**1.71133866875722e-09**	-1.35	2.36352983542059e-05	6	Sfrp2
**1.7272568292087e-09**	0.92	2.38551440682014e-05	6	Smarcd3
**1.73355701047407e-09**	1.07	2.39421558716574e-05	6	Tnc
**1.73699736136071e-09**	0.641643434803589	2.39896705577527e-05	6	Atp5l
**1.8184170651143e-09**	-1.03	2.51141580862936e-05	6	Tgfbr2
**2.0348082394195e-09**	1.09	2.81027365946228e-05	6	Tnfrsf11a
**2.04051454781111e-09**	1.17	2.81815464198193e-05	6	Ttll7
**2.13736792707571e-09**	-0.821601193558883	2.95191884408426e-05	6	Ctsl
**2.25116039363188e-09**	-0.963790354778789	3.109077619645E-05	6	Ace
**2.32602945515245e-09**	-1.42	3.21247928051105e-05	6	Ece1
**2.41645650185322e-09**	1.23	3.33736807470948e-05	6	Emb
**2.50844131421912e-09**	-1.53	3.46440829906803e-05	6	Fam102b
**2.65039074288488e-09**	0.458618886791551	3.66045465499831e-05	6	Crip1

**2.70737283289417e-09**	0.91	3.73915261951014e-05	6	Grip2
**2.7082048490017e-09**	0.44	3.74030171695624e-05	6	S100a11
**2.73615665668902e-09**	-1.97	3.77890595855321e-05	6	Mdk
**2.804337953799E-09**	1.52	3.8730711479918e-05	6	Tcap
**2.91121086318291e-09**	0.829706349263335	4.02067332314192e-05	6	Dynlt3
**2.94090837023078e-09**	-0.956512171266531	4.06168855012574e-05	6	H2-K1
**2.95789194002334e-09**	0.636214454352431	4.08514455836623e-05	6	Atp6v0e
**3.58504315230029e-09**	0.658686812704972	4.95130309764194e-05	6	Mtus2
**3.72883908749716e-09**	0.68	5.14989966374233e-05	6	Jph2
**3.80436981275867e-09**	-1.92	5.254215148401E-05	6	Fndc1
**3.84825137886654e-09**	-1.43	5.31481997935257e-05	6	Boc
**4.01807922988776e-09**	-1.75	5.54936922439799e-05	6	Fmo1
**4.07105488582203e-09**	-1.55	5.62253390280881e-05	6	Tcn2
**4.51098300579024e-09**	-0.665707582476203	6.2301186292969e-05	6	Sptbn1
**5.01880621675041e-09**	0.447961952502581	6.93147326595399e-05	6	Arpc2
**5.24489442038133e-09**	0.479744247786736	7.24372368398865e-05	6	Fermt2
**5.3279138317506e-09**	-0.40	7.35838179303075e-05	6	Malat1
**5.36648633255119e-09**	-1.07	7.41165427388644e-05	6	Abi3bp
**6.06764920957268e-09**	0.830208531616006	8.38003032334083e-05	6	Itgb3
**6.27098653769374e-09**	0.909890710028688	8.66085950720883e-05	6	Arap2
**6.44529846876232e-09**	0.292400253734882	8.90160171520764e-05	6	Calm1
**6.50195456848521e-09**	-0.735220067852095	8.97984945453493e-05	6	Sepp1
**7.12607371463634e-09**	0.522682993260796	9.84182040728425e-05	6	Nptn
**7.16322388878299e-09**	0.748938405934737	9.89312851279819e-05	6	H3f3a
**7.17802299722998e-09**	-1.16	9.91356756147432e-05	6	Psmb10
**7.24571753632217e-09**	0.941710936543291	0.000100070604894146	6	Zc2hc1a
**7.54023167225418e-09**	0.911431593996851	0.000104138139625503	6	Fgfr2
**7.94190355303061e-09**	0.539671540982198	0.000109685629970906	6	Ubl7
**8.28998781117414e-09**	-0.773324394682057	0.000114493021660126	6	Tapbp
**8.35069193899169e-09**	1.21	0.000115331406369414	6	Xk
**8.51230429668125e-09**	0.622928554933623	0.000117563434641465	6	Cox6a1
**8.63230767570365e-09**	0.634208564053471	0.000119220801309143	6	Filip1l
**8.80271218974482e-09**	1.50	0.000121574258052566	6	Srpk3
**8.84892726887796e-09**	-1.13	0.000122212534510473	6	Fosl2
**8.93708476226197e-09**	-1.34	0.0001234300776516	6	Ptgs2
**8.99839506850056e-09**	-2.05	0.000124276834291061	6	1500015O10[illegible]
**9.43613378042344e-09**	-1.17	0.000130322443641428	6	Ifngr1
**9.79970191421648e-09**	-1.06	0.000135343683137244	6	Mllt4
**1.22474545620352e-08**	-1.42	0.000169149594956268	6	Sox9
**1.26205726638826e-08**	0.758220890364291	0.000174302729060883	6	Itpk1
**1.30376979037715e-08**	0.757066114469486	0.000180063645748988	6	Mertk
**1.37594432065766e-08**	0.767636678192365	0.00019003167012603	6	Nqo1
**1.44871410478364e-08**	-1.26	0.000200081905011669	6	Ptgfrn
**1.50599135429722e-08**	0.698249381246711	0.00020799246594199	6	Prdm16
**1.5198078250773e-08**	-2.08	0.000209900658721426	6	Il6
**1.56379818411568e-08**	0.746245585996073	0.000215976167208217	6	Cfl2
**1.58317016299791e-08**	1.31	0.000218651631211641	6	Btc
**1.59297284585498e-08**	-1.86	0.000220005479741032	6	Igsf10
**1.688603963977E-08**	-2.12	0.000233213093464863	6	C2
**1.69818598444206e-08**	-1.00	0.000234536466311292	6	Tgif1
**1.7187454269936e-08**	0.747663835700346	0.000237375930922086	6	Cox17
**1.74616863748388e-08**	1.23	0.000241163350522899	6	Sorl1
**1.89269483137922e-08**	0.800281465466988	0.000261400083161784	6	Mrps14
**1.93148640923599e-08**	1.15	0.000266757587979583	6	Atp1b2
**1.93575735991375e-08**	-0.488619403894116	0.000267347448977688	6	Ftl1
**1.94373147286247e-08**	-2.12	0.000268448753717035	6	Tmem100

**1.97587426897958e-08**	-1.04	0.00027288799528877	6	Pdlim2
**2.00100735557414e-08**	-1.97	0.000276359125878345	6	Cpne8
**2.11695008898436e-08**	-1.85	0.00029237197678963	6	Wnt5a
**2.17145401502516e-08**	-2.06	0.000299899514015124	6	Penk
**2.20389983322356e-08**	0.625931708722657	0.000304380605966506	6	Wdr1
**2.21868597229042e-08**	0.94	0.000306422719633029	6	Usp20
**2.2306338354749e-08**	-2.07	0.000308072839017439	6	Ccl7
**2.5525115306194e-08**	0.878568238899646	0.000352527367493845	6	Ppp2r5a
**2.55342107300851e-08**	1.15	0.000352652984393205	6	Ryr2
**2.66907439573693e-08**	0.472221658615732	0.000368625864795228	6	Pi15
**2.822036418858E-08**	-1.07	0.000389751449808478	6	Pnp
**2.95498651362274e-08**	0.405564412786942	0.000408113187396436	6	Tmem47
**3.12147031351228e-08**	0.26	0.000431106264999181	6	Rpl41
**3.27229730299423e-08**	0.672252169528309	0.000451936980516533	6	Fam126b
**3.39841644151627e-08**	-1.78	0.000469355294737813	6	Sdc1
**3.50022189344638e-08**	-2.19	0.00048341564570388	6	Srgn
**3.58918827942787e-08**	-1.99	0.000495702793271782	6	Arhgdib
**3.6158169824097e-08**	-1.06	0.000499380483440604	6	Rbms1
**3.70919037562923e-08**	1.46	0.000512276282778153	6	Timp4
**3.96148498493588e-08**	-1.61	0.000547120691269495	6	Gmfb
**3.96372134727718e-08**	1.37	0.000547429555272451	6	Pnck
**4.10789275796031e-08**	-0.827110267115436	0.000567341068801899	6	Scara3
**4.41272402869913e-08**	-1.74	0.000609441315603637	6	Pdgfrl
**4.62206730530726e-08**	-1.32	0.000638353715535986	6	Tmem176a
**4.66952263486538e-08**	0.71	0.000644907771101258	6	Inpp5a
**4.74018145303132e-08**	1.16	0.000654666460478156	6	Akap6
**4.75568554579753e-08**	0.975420016940364	0.000656807730730097	6	Lama5
**4.88335392032303e-08**	1.23	0.000674440009935813	6	Fabp3
**4.96136293923603e-08**	0.929449081692688	0.000685213835537888	6	Itga7
**5.17122730439342e-08**	0.708776154341075	0.000714198203009775	6	5930403L14F
**5.22797342021068e-08**	-1.13	0.000722035409065297	6	Gba
**5.22970085216042e-08**	0.615288194732333	0.000722273984691876	6	Ndufa2
**5.23315746484098e-08**	-1.39	0.000722751377469188	6	Raph1
**5.59907500699056e-08**	0.722589454830191	0.000773288249215466	6	Adcy5
**5.69929530530057e-08**	-0.90	0.000787129674615061	6	Mfap4
**5.71618204530893e-08**	0.609932519745363	0.000789461902277616	6	Bnip2
**5.76335999727948e-08**	0.784611618323899	0.000795977649224269	6	Nkd1
**5.88294924943826e-08**	-1.76	0.000812494120839919	6	Matn2
**6.02500088610674e-08**	0.565689940315131	0.000832112872380202	6	Id2
**6.13653378475948e-08**	-0.878548860489697	0.000847516681013132	6	Nucb2
**6.74098170012521e-08**	-1.70	0.000930996982604293	6	Fbln7
**6.80646342998691e-08**	0.393744226630397	0.000940040664315492	6	Chchd2
**6.8814466309254e-08**	0.532082382236667	0.000950396594197107	6	Ndufa7
**7.10166262989995e-08**	0.284848001143893	0.000980810625815482	6	NP-904330.1
**7.2965542947182e-08**	-1.52	0.00100772711364353	6	Sdc3
**7.32900043939843e-08**	-1.90	0.00101220825068532	6	Tmem119
**7.53642354727898e-08**	-0.812355143215602	0.0010408554561147	6	Shisa5
**7.53770784159982e-08**	0.690345778514958	0.00104103283000335	6	Endod1
**7.57650101996144e-08**	-1.72	0.00104639055586687	6	Egfr
**7.61412271728204e-08**	-1.43	0.00105158648848382	6	Dapk1
**8.02004245552752e-08**	0.63	0.00110764806353291	6	Slc25a23
**8.04857322567317e-08**	0.950959406995684	0.00111158844819772	6	Lancl3
**8.871905757831E-08**	-2.01	0.00122529890421404	6	Ptprb
**8.93102817468653e-08**	-1.17	0.00123346430120596	6	Kdm6b
**9.20539327280145e-08**	0.410693124257816	0.00127135686490661	6	Npr1
**9.2461969690532e-08**	0.839215752358751	0.00127699226339594	6	Gnai1

**9.43080798113839e-08**	0.68	0.00130248889027502	6	Cnn2
**9.83566814143605e-08**	0.766138400566928	0.00135840412701373	6	Emc8
**9.88049912713431e-08**	0.616522504157118	0.00136459573444852	6	Kctd10
**1.00080372238557e-07**	-1.18	0.00138221002098671	6	Abca1
**1.02983851493908e-07**	0.261546806484581	0.00142230997298237	6	Lphn2
**1.03402252058341e-07**	-1.25	0.00142808850317774	6	Birc3
**1.0438560292726e-07**	0.447242173509586	0.00144166956202839	6	Cyb5r3
**1.04674875738544e-07**	-1.91	0.00144566470882503	6	Thy1
**1.11495806040419e-07**	0.652406012646644	0.00153986857722423	6	Stom
**1.12534266080022e-07**	-1.65	0.00155421074883119	6	Mfap2
**1.13531603612747e-07**	0.987699568087155	0.00156798497749565	6	Macrod1
**1.16740850978951e-07**	0.381841586296782	0.00161230789287029	6	Scube3
**1.32661905560652e-07**	0.60	0.00183219357769817	6	Prkcdbp
**1.33194590983419e-07**	0.936486997284961	0.001839550496072	6	Ppp1r12b
**1.35486068959462e-07**	0.97	0.00187119809839913	6	Myo18b
**1.35503205695785e-07**	-2.01	0.00187143477386449	6	S1pr3
**1.3982124736796e-07**	0.378689666588088	0.0019310712473989	6	Id1
**1.45055831136194e-07**	0.70	0.00200336608382197	6	Camk2g
**1.4852529799217e-07**	0.337238769279198	0.00205128289056986	6	Pde5a
**1.49096801981106e-07**	-2.00	0.00205917593216105	6	Fyn
**1.55871319306496e-07**	0.396157385905478	0.00215273879094201	6	Ltbp1
**1.61308484072015e-07**	-1.79	0.0022278314735186	6	Lamb1
**1.70445938547656e-07**	-0.979962606594937	0.00235402885728168	6	Pmp22
**1.74292323826581e-07**	0.333416397212714	0.00240715128436891	6	Eif4g2
**1.74616824850385e-07**	-1.90	0.00241163296800867	6	Tmem204
**1.77934620550229e-07**	0.861526440243888	0.00245745504441921	6	Myocd
**1.78680077067936e-07**	0.767380359466878	0.00246775054438526	6	Sec24d
**1.79443976070996e-07**	0.629413353494264	0.00247830075351652	6	Txnip
**1.83976307664582e-07**	0.710482411919976	0.00254089678515555	6	S100a4
**1.85253835832537e-07**	0.480162235294676	0.00255854072668317	6	9530077C05[illegible]
**1.86265691787479e-07**	0.527470891038407	0.00257251546927688	6	Naca
**1.96833774372123e-07**	0.96	0.00271847125785339	6	Tub
**1.97757728812905e-07**	0.40	0.00273123199263503	6	Micall1
**2.01381123834703e-07**	0.908306342123985	0.00278127470128108	6	AI414108
**2.02275804238898e-07**	-2.12	0.00279363113234342	6	Adh1
**2.07999922937015e-07**	0.540895281701915	0.00287268693568311	6	Pip5k1b
**2.33313385935869e-07**	-2.09	0.00322229117316029	6	Cxcl2
**2.46102933458871e-07**	-2.20	0.00339892761400047	6	Angptl7
**2.49242391433189e-07**	-0.747961279896898	0.00344228666808378	6	Slc3a2
**2.52639219853757e-07**	-1.48	0.00348920026540024	6	Rhou
**2.55821480262139e-07**	-2.09	0.00353315046390041	6	Psmb8
**2.58489493359701e-07**	-1.31	0.00356999839279084	6	Mmp14
**2.65210951953387e-07**	-2.23	0.00366282845742822	6	Ccl2
**2.65659696028847e-07**	0.260106145861464	0.0036690260618544	6	Lrrc58
**2.66315423357485e-07**	0.516425097286696	0.00367808231199022	6	Kcnc4
**2.69425027127839e-07**	0.54	0.00372102904966259	6	Psmb7
**2.73196072951534e-07**	-0.984925126372343	0.00377311096353364	6	Ap2a2
**2.75805243169021e-07**	-1.71	0.00380914621340735	6	Plekha6
**2.81525325645128e-07**	0.766183989362512	0.00388814627248487	6	Nrtn
**2.88197994466752e-07**	0.727428545810429	0.00398030250158031	6	Inpp4a
**2.93606497678073e-07**	0.56	0.00405499933943186	6	Ndufv3
**2.98335941321101e-07**	0.909030741507866	0.00412031768558573	6	Ccdc107
**3.07710063913926e-07**	0.32	0.00424978369271524	6	Clu
**3.1125433122832e-07**	0.561936498082535	0.00429873356859432	6	Cox7a2
**3.12097293060286e-07**	0.684300122912949	0.00431037571445561	6	Nat6
**3.16812814606945e-07**	-1.89	0.00437550178253652	6	Smim3

3.19840546844738e-07	-1.39	0.00441731779247268	6	Lasp1
3.271053522169E-07	-1.86	0.00451765201946761	6	Podn
3.30846620978792e-07	-1.64	0.0045693226823381	6	Nr4a2
3.43151192994881e-07	-1.23	0.0047392611264523	6	Frzb
3.53274761544685e-07	-1.17	0.00487907773169364	6	Magt1
3.56718529981353e-07	0.582641348365548	0.00492663961757247	6	Carns1
3.59265337151195e-07	0.873826995348511	0.00496181357139516	6	Mthfd2l
3.7779927870256e-07	-0.424491595520551	0.00521778583816105	6	Hspa5
3.80385724403973e-07	-1.53	0.00525350723974327	6	Igfbp5
3.98541322068341e-07	0.437999368616005	0.00550425419908586	6	D8Ertd738e
4.02114554391888e-07	0.412384467919443	0.00555360411070637	6	Uqcrh
4.03081902750886e-07	-1.11	0.00556696415889249	6	Med13
4.1362266161938e-07	0.787425449569174	0.00571254257962526	6	Ptp4a3
4.4145631773162e-07	0.458877463604706	0.00609695320419141	6	Ndufa5
4.61468040810668e-07	0.423877251725469	0.00637333511163614	6	Zfhx3
4.73928606032454e-07	-1.63	0.00654542797791423	6	Tnfaip6
4.74254712747894e-07	0.961428705949563	0.00654993183776117	6	Lnpep
4.81668621524699e-07	-0.966637479945163	0.00665232533187762	6	Ppp3ca
4.82788844817355e-07	0.432783592990585	0.0066677967357725	6	Shfm1
4.88518660880959e-07	-1.73	0.00674693122542692	6	Itih5
4.92916223978812e-07	-1.88	0.00680766596937138	6	Ahsg
5.03582853531435e-07	0.480190631075065	0.00695498279012265	6	BC048507
5.18708619727688e-07	0.389920465135903	0.0071638847470591	6	Fn1
5.22715849907222e-07	-1.82	0.00721922860306865	6	Cmtm3
5.31777491150162e-07	0.412624616048329	0.00734437893027488	6	Cav3
5.33886026423457e-07	0.294628292559208	0.00737349991093436	6	Rps3a1
5.42645455153497e-07	-0.751897348460053	0.00749447638112495	6	Tpm4
5.44300080952515e-07	-1.26	0.00751732841803519	6	Cd248
5.58842555886375e-07	0.310304827596919	0.00771817453934673	6	NP-904338.1
5.63434866740923e-07	0.72	0.00778159894455889	6	Clybl
5.65032396202803e-07	0.816872292682018	0.00780366242395692	6	Slc24a3
5.69367819509383e-07	-1.20	0.00786353895524409	6	B4galt1
5.74078521157791e-07	-0.92	0.00792859845571025	6	Nop58
5.83919185525624e-07	-1.83	0.00806450787129439	6	Pdpn
5.86814452777426e-07	0.590120323025406	0.00810449440730903	6	Nav2
5.94731051772578e-07	-0.741618906230277	0.00821383055603108	6	Cdkn1a
5.99138537148786e-07	-1.32	0.00827470233656189	6	Oaf
5.99551858617894e-07	-0.901195721776648	0.00828041071937174	6	Crispld2
6.10032391193428e-07	1.01	0.00842515735477243	6	Fstl3
6.31774268299413e-07	0.679602218040764	0.00872543441948319	6	Cacna2d1
6.39544449121405e-07	0.536804860495061	0.00883274838681572	6	2900026A02F
6.91517323374397e-07	0.527588501200061	0.0095505457531238	6	Tecr
7.30536262313352e-07	0.434123242647916	0.0100894363188097	6	Ndufa13
7.39485123902997e-07	0.628941814256369	0.0102130290462243	6	Sema5a
7.47245763771856e-07	0.435927915421342	0.0103202112434531	6	Atp2a2
7.50908766214679e-07	-0.730383123881373	0.0103708009701909	6	Bmp1
7.52162256488291e-07	-1.85	0.0103881129243598	6	Gpr133
7.59467138863763e-07	0.494124727978228	0.0104890006548474	6	Cnn3
7.74769451184383e-07	0.723177156743518	0.0107003408903075	6	Napepld
7.75890682566005e-07	-0.709678703796071	0.0107158262169191	6	Gas6
7.8007934694826e-07	0.521826718542257	0.0107736758607024	6	Olfr1033
7.82144203621309e-07	-1.51	0.0108021935962139	6	Cxcl16
8.20138485508066e-07	-1.12	0.0113269326233519	6	Tgfbr3
8.91952411002965e-07	-0.581237824490224	0.012318754748362	6	Tuba1a
9.00814308555439e-07	-1.83	0.0124411464154592	6	Ppl
9.09783299699097e-07	-1.86	0.0125650171521442	6	Flt3l

**9.22675263688758e-07**	0.29	0.0127430680668054	6	Ptprz1
**9.36650179524653e-07**	-1.94	0.012936075629415	6	Lbp
**9.60330594086869e-07**	-0.494585677952366	0.0132631258349337	6	Hsp90b1
**9.84693060084647e-07**	-1.83	0.0135995958528291	6	Ecscr
**1.00624534143885e-06**	-1.97	0.0138972544106119	6	Cd55
**1.01846380612917e-06**	1.08	0.014066004	6	Tppp3
**1.10634580793754e-06**	0.41	0.0152797419534253	6	Hes1
**1.10785408842938e-06**	0.80	0.0153005728152981	6	Gucy1a3
**1.13952063233665e-06**	-0.706084230958987	0.0157379194532014	6	Zbtb20
**1.14832128832599e-06**	-1.54	0.0158594653130702	6	Dtx4
**1.17430286140445e-06**	0.827575350061844	0.0162182968188569	6	Gde1
**1.17862541968516e-06**	0.388817725484905	0.0162779956712717	6	Tbc1d9
**1.18285152081256e-06**	0.861010793816474	0.0163363623539423	6	Xpr1
**1.21496837496549e-06**	0.810111604209056	0.0167799282266484	6	Dctn6
**1.25703673696147e-06**	-0.813303460024461	0.0173609343741749	6	Xbp1
**1.27195304822897e-06**	0.80	0.0175669435490903	6	Camta1
**1.27917663153725e-06**	-1.68	0.017666708458161	6	St3gal4
**1.29127298051997e-06**	0.561720753353647	0.0178337711339613	6	Smim14
**1.30771000572378e-06**	-1.77	0.0180607828890511	6	Kcnt2
**1.3372728163066e-06**	0.441346389472104	0.0184690748660105	6	Gm6402
**1.34652974127357e-06**	0.499588530112515	0.0185969222567292	6	Ndufa3
**1.37720337795787e-06**	-0.717731038155637	0.0190205558529762	6	Ctsz
**1.43243115853889e-06**	-0.95	0.0197833067305806	6	Grina
**1.44169251371228e-06**	-1.15	0.0199112153068803	6	Cnot6
**1.44610945711649e-06**	0.524094766374784	0.0199722177122359	6	Sorbs2
**1.45300106174684e-06**	-1.76	0.0200673976637856	6	Deptor
**1.46383390552277e-06**	0.882330633902974	0.020217010069175	6	Fibin
**1.51574292159403e-06**	-1.69	0.0209339254901352	6	Gbp7
**1.52447675341426e-06**	-0.742907215970054	0.0210545484414043	6	Lrp1
**1.54439295231405e-06**	-1.93	0.0213296110644093	6	Selp
**1.55438309877093e-06**	0.716503790484137	0.0214675849771253	6	Ndufb10
**1.55575184459454e-06**	-1.57	0.0214864887256952	6	Uap1l1
**1.5564108770063e-06**	0.627824795484917	0.021495590622334	6	Mpc2
**1.58430214184484e-06**	-0.782649051935729	0.0218807968810191	6	F3
**1.61580594035768e-06**	-1.11	0.0223158958422799	6	Tmed5
**1.63178327087331e-06**	0.494895847642754	0.0225365587540313	6	Bmpr1a
**1.63256270124838e-06**	-1.77	0.0225473234669414	6	Rasa3
**1.65547845454982e-06**	-0.489545483428749	0.0228638129357876	6	Nfib
**1.70461570622395e-06**	0.754600492285763	0.0235424475186589	6	Trabd2b
**1.71995810323171e-06**	-1.01	0.0237543413637332	6	Sntb2
**1.7420697475978e-06**	-1.69	0.0240597252840733	6	Abca9
**1.76896787362483e-06**	-0.739699408254101	0.0244312153026325	6	Mxra8
**1.77080721384423e-06**	0.839896586893634	0.0244566184304026	6	Adamts8
**1.81282111273613e-06**	-1.56	0.0250368723879987	6	Cdh11
**1.83432501289989e-06**	0.524446185719675	0.0253338627531604	6	Npy1r
**1.84499636805853e-06**	0.59	0.0254812448392563	6	Sdhc
**1.86055352037465e-06**	0.907792702365681	0.0256961046698942	6	Ube2a
**1.89832199280087e-06**	0.391482247153225	0.0262177250425729	6	Uqcr11
**2.00158184572274e-06**	0.784977048622137	0.0276438468712768	6	Sema3a
**2.06160411797594e-06**	0.760466705926435	0.0284728144733657	6	Sept6
**2.10204253540555e-06**	0.778849637292033	0.029031309456486	6	Mtss1
**2.14070099032523e-06**	-1.18	0.0295652213773818	6	Lipa
**2.15507910376925e-06**	0.450268951210016	0.029763797502157	6	Tusc1
**2.16483730673036e-06**	-2.07	0.0298985680432531	6	Man1a
**2.25236380431377e-06**	-1.91	0.0311073965013775	6	Rftn1
**2.26691360912995e-06**	0.889879090959319	0.0313083438556938	6	Fmo3

**2.27468648417501e-06**	0.315500007916655	0.0314156950329411	6	Cd9
**2.30810488594053e-06**	0.660813404681532	0.0318772365797247	6	Sec61g
**2.32779332344389e-06**	-1.17	0.0321491535900836	6	Tmem9b
**2.3296130797912e-06**	-1.08	0.0321742862449963	6	H6pd
**2.33078380191003e-06**	-1.08	0.0321904550881794	6	Arhgap29
**2.34053226815167e-06**	0.950800612772813	0.0323250911554427	6	Trpv2
**2.3693059276726e-06**	1.32	0.0327224841670863	6	0610009L18R
**2.37538433675047e-06**	-0.83	0.0328064330748608	6	Jmjd1c
**2.44314276028004e-06**	0.447276379214807	0.0337422446622276	6	Nudt4
**2.44786331358827e-06**	-1.10	0.0338074402239676	6	Ptbp3
**2.46777564067829e-06**	0.498726034313292	0.0340824493734079	6	Flnc
**2.48854214386149e-06**	0.607098198339016	0.0343692555488711	6	Gng12
**2.49431889233891e-06**	0.733846753848481	0.0344490382220927	6	Ndufv2
**2.54810022646006e-06**	-0.47	0.0351918122276399	6	Col1a2
**2.58290341229405e-06**	0.800306517170922	0.0356724790271932	6	Sept9
**2.58463754540726e-06**	1.14	0.0356964291396196	6	Smim5
**2.64099740392245e-06**	0.456309065260565	0.036474815145573	6	Rcn2
**2.70276244855166e-06**	0.45	0.0373278521769469	6	Tagln2
**2.72892365488874e-06**	-0.890325469217591	0.0376891645976683	6	Rcn1
**2.74406921894939e-06**	-0.674082643568923	0.03789834	6	Grn
**2.7638639380668e-06**	-2.12	0.0381717248486406	6	G0s2
**2.78814251805788e-06**	-0.831002022325285	0.0385070363168973	6	Htra3
**2.79154771122657e-06**	-0.794373154198062	0.0385540654397502	6	Plce1
**2.80380887211042e-06**	-1.01	0.038723404332717	6	Sept11
**2.81345316487455e-06**	0.33	0.0388566016600824	6	Pkm
**2.8347221495313e-06**	0.513652971492925	0.0391503476071768	6	Rexo2
**2.83639559718259e-06**	0.511344556645956	0.0391734595926888	6	Psmb6
**2.90999317163701e-06**	-1.00	0.0401899156934788	6	Sik1
**2.93131105229971e-06**	0.567429601430371	0.0404843369433112	6	Ndufa12
**2.95057006369194e-06**	-0.939274445175848	0.0407503231496494	6	Igsf3
**2.98013439609754e-06**	-0.716979860295402	0.0411586361445032	6	Mt2
**3.01976950465549e-06**	0.483102929242689	0.041706036628797	6	Dpysl3
**3.25120884886156e-06**	1.14	0.044902445411627	6	Otud1
**3.28829899311215e-06**	-0.750753080384848	0.0454146973938719	6	Csrnp1
**3.36453507809835e-06**	-1.73	0.0464675939636163	6	C1qtnf7
**3.50136087431835e-06**	-0.833198710821703	0.0483572950352107	6	Map4k4
**3.51310168948599e-06**	-1.14	0.048519447433491	6	Tsc22d3
**3.51914676402431e-06**	0.890056927866115	0.0486029359579398	6	Uchl1
**3.55736697935766e-06**	-1.55	0.0491307953519086	6	Bok
**3.6033901769693e-06**	0.854543368101672	0.0497664217341231	6	Snrpd2
**3.82756722600742e-06**	0.61	0.0528625309583884	6	Sema3d
**3.99291906482296e-06**	-0.584789887665566	0.0551462052042699	6	Eif1a
**4.03260184321204e-06**	0.652816574616837	0.0556942640566014	6	Cds2
**4.07819817924976e-06**	1.55	0.0563239950536184	6	Rgs5
**4.09330947554976e-06**	0.641874998441232	0.0565326971668177	6	Tesk1
**4.09776311425524e-06**	0.873258325572994	0.0565942063709792	6	Arhgef17
**4.11220910634737e-06**	1.13	0.0567937199677635	6	Igsf9
**4.1611219201611e-06**	-0.690201922256096	0.057469254839345	6	Cd47
**4.18319252241345e-06**	-0.834878526287773	0.0577740719270522	6	Sri
**4.1865583470994e-06**	1.00	0.0578205573317898	6	Ift43
**4.21471216287125e-06**	-0.883338503869348	0.0582093896814149	6	Tnfrsf1a
**4.23713672055587e-06**	-1.56	0.0585190952475971	6	Nod1
**4.33624057039361e-06**	0.631390982774155	0.0598878185177062	6	Cox5a
**4.43270476488444e-06**	0.48	0.061220085507819	6	Ncs1
**4.55940190431392e-06**	-0.802140072567821	0.0629698997004796	6	Xpnpep1
**4.64551835499302e-06**	0.634906735221209	0.0641592540008086	6	Snapc2

**4.72025314858527e-06**	-0.494745821385365	0.0651914162351112	6	Npc2
**4.97566479564946e-06**	-0.702226971762528	0.0687189064927147	6	S100a10
**4.98344345734211e-06**	1.10	0.0688263375893519	6	Hspb11
**5.08493506512512e-06**	0.451815651648226	0.0702280381844431	6	Plcb4
**5.17179147357603e-06**	0.375658758185457	0.0714276120415586	6	Cdh13
**5.20381408196383e-06**	-0.939939258414256	0.0718698762860025	6	Igsf8
**5.27567600137306e-06**	0.454072944252744	0.0728623612549634	6	Dynlrb1
**5.28569906653712e-06**	-1.58	0.0730007898079442	6	Abca8b
**5.44318089492274e-06**	-1.82	0.0751757713397779	6	S1pr2
**5.58761404720318e-06**	-0.84	0.0771705376059231	6	Epb4.1l2
**5.61825500610847e-06**	0.493027427218514	0.0775937198893641	6	Ndrg2
**5.62739496010349e-06**	0.521382798817062	0.0777199517939893	6	Snrnp27
**5.66356690619156e-06**	-1.19	0.0782195225414117	6	Jade1
**5.68509255146504e-06**	0.436392109912283	0.0785168132282837	6	Atp2b4
**5.77105254518285e-06**	0.612041402081615	0.0797040067015204	6	Camk1
**5.85655809739774e-06**	-0.841442316395507	0.0808849238831602	6	Hexa
**5.89786935579968e-06**	0.666404229120244	0.0814554736729493	6	Ndufb9
**5.99626093157705e-06**	0.575202075856565	0.0828143597260106	6	Mrps21
**6.2530181170817e-06**	-1.40	0.0863604332150154	6	Slc4a4
**6.29173343274373e-06**	-0.742892974080098	0.0868951304396237	6	Pkd2
**6.43824176923396e-06**	-0.64	0.0889185570748903	6	Arl6ip1
**6.48383811753729e-06**	1.09	0.0895482882413075	6	Ppil1
**6.51013099426777e-06**	-1.64	0.0899114191618322	6	Gpr39
**6.75235315480049e-06**	-0.763867168192972	0.0932567494209495	6	Fcgrt
**6.79176956176238e-06**	0.653224466675834	0.0938011294175003	6	Mfhas1
**6.85616920007105e-06**	0.522102245607087	0.0946905528221813	6	Twsg1
**7.03118269358301e-06**	0.629897928400602	0.0971076641810749	6	Psma2
**7.06224719926845e-06**	-1.54	0.0975366960690965	6	Hfe
**7.15205353386446e-06**	0.845177575542121	0.0987770113562021	6	Snta1
**7.23774975186576e-06**	-1.71	0.099960561823018	6	Gpc3
**7.36505928582122e-06**	0.854757350488574	0.101718833796477	6	Akt3
**7.56216890197695e-06**	1.36	0.104441114705204	6	Lgr6
**7.56437600994333e-06**	0.615641202360963	0.104471597073327	6	Ilk
**7.60911174824855e-06**	-1.01	0.105089442355061	6	Gaa
**7.65508968583624e-06**	-0.68	0.105724443651084	6	Scd2
**7.72258453880023e-06**	-1.62	0.106656615	6	Runx1
**7.74967337791767e-06**	-0.740507008791488	0.107030739022421	6	Skil
**7.77784234398341e-06**	0.455545915984969	0.107419780612755	6	Asph
**7.79482240262356e-06**	-1.38	0.107654292202634	6	Psd3
**7.81036806699638e-06**	-1.02	0.107868993373287	6	F2r
**7.88397459103175e-06**	-0.825793950039401	0.108885573076739	6	Lmo4
**7.93365959045445e-06**	-1.37	0.109571772603766	6	Ubtd2
**7.98048651072874e-06**	0.659238175542185	0.110218499199675	6	Prdm6
**8.16394002061291e-06**	0.509676926816423	0.112752175624685	6	Rpl29
**8.24614396967038e-06**	-0.835552223064617	0.113887494365118	6	Mpv17
**8.49964389827868e-06**	0.397557358875683	0.117388581879127	6	Atp5e
**8.55972765702884e-06**	-1.38	0.118218398671225	6	Smc4
**8.75252549489608e-06**	0.63	0.12088113	6	Agl
**8.86365645446777e-06**	-2.01	0.122415959292654	6	Ptx3
**8.97731898322449e-06**	-0.483802168896411	0.123985752477313	6	Lman1
**9.40379231572512e-06**	0.320382133379549	0.129875776	6	Calcoco1
**9.50821409942662e-06**	0.741943559325599	0.131317944927181	6	Dbndd2
**9.76915337147792e-06**	1.05	0.134921777213482	6	Jazf1
**1.00161933757686e-05**	0.561818000351983	0.138333647	6	Vamp8
**1.00756531216109e-05**	1.00	0.139154845262569	6	Ethe1
**1.01448636858868e-05**	0.906195366057563	0.140110712365783	6	Prkaa2

**1.02090662372647e-05**	-1.48	0.140997413802863	6	Itpr2
**1.03122248650332e-05**	0.489063952312209	0.142422137610973	6	Stbd1
**1.05066272042142e-05**	0.385724713973911	0.145107028317403	6	Polr2l
**1.07853690164622e-05**	-1.44	0.148956731486359	6	1700025G04I
**1.09487749471981e-05**	0.393139854738838	0.151213530795753	6	Dag1
**1.0995381234265e-05**	-0.849661517225945	0.151857210226434	6	Chpf
**1.10823860826628e-05**	1.45	0.153058834187656	6	Tbxa2r
**1.11516866347477e-05**	0.469222566051925	0.154015944112501	6	Aldoart1
**1.11634544333287e-05**	-0.797029879262338	0.154178469178702	6	Rap1b
**1.12850462748607e-05**	-0.784521622485291	0.155857774	6	Olfml2b
**1.1926651796136e-05**	0.666051091624464	0.164718987956434	6	Ndufb7
**1.1928200187148e-05**	0.696114908718078	0.164740372784701	6	Ywhah
**1.24104738038253e-05**	0.968833245662138	0.171401053704632	6	Dnajb5
**1.24166767211193e-05**	0.359021113713495	0.171486722195379	6	Zcrb1
**1.25141522589158e-05**	-0.717573074689066	0.172832956847886	6	Klf4
**1.26227498849052e-05**	0.767797788742378	0.174332798660425	6	Sesn1
**1.26372114428945e-05**	-1.60	0.174532527237816	6	Zfp521
**1.28035787477925e-05**	-0.868355253873974	0.176830226085762	6	Cdkn1c
**1.32887067790509e-05**	-0.669890992822525	0.183530329325472	6	Mtch1
**1.33162636310485e-05**	-0.534047460439623	0.183910917008411	6	Pdia6
**1.33278452196537e-05**	-1.74	0.184070870328638	6	Tcf21
**1.33972071643521e-05**	0.417842830582443	0.185028828146866	6	Atp5k
**1.34133899904753e-05**	-0.854708034346449	0.185252329158454	6	Arih1
**1.34786967148811e-05**	0.351319590564751	0.186154280329223	6	Serf2
**1.3490188462947e-05**	0.561494680478719	0.186312992861761	6	Tead3
**1.39554046505115e-05**	0.825263124394334	0.192738093628215	6	Cdo1
**1.43739277365216e-05**	-1.34	0.198518316	6	Ltbp2
**1.46713924968036e-05**	0.471958166448724	0.202626601773354	6	Hist1h2bc
**1.47813280728614e-05**	-1.51	0.204144922014289	6	Plxna2
**1.52514035775172e-05**	-1.37	0.210637135	6	Prrg3
**1.54763123324967e-05**	-0.616028542443494	0.213743349624111	6	Gbp2
**1.54818396732812e-05**	0.431621053983276	0.213819687727687	6	Gstp1
**1.60433277190746e-05**	0.871485148420061	0.221574399128139	6	Siva1
**1.60844277209283e-05**	0.396522865899517	0.222142031253741	6	Rps13
**1.65770869779066e-05**	-0.731516568461472	0.228946148251869	6	Kdelr2
**1.6835545478258e-05**	-0.676150444898055	0.232515718600221	6	Zfp36
**1.78397172649412e-05**	-1.44	0.246384335146102	6	Fmnl2
**1.81136740194067e-05**	0.604002528270839	0.250167951882026	6	Mrpl33
**1.83052459652651e-05**	-1.19	0.252813752026276	6	Casp4
**1.83172978395993e-05**	0.610588937964238	0.252980200462705	6	1810026J23F
**1.84343989894376e-05**	0.712623792793145	0.254597484443123	6	Lrch1
**1.86535160439758e-05**	-1.74	0.25762371	6	Bcl3
**1.8698267791373e-05**	-0.354523481306995	0.258241776466653	6	B2m
**1.89357503641598e-05**	-1.27	0.261521648279411	6	Eng
**1.9672419088249e-05**	0.541948295745529	0.271695780027807	6	4930523C07F
**1.99675722695104e-05**	-0.922293060335976	0.275772140614208	6	Myh9
**2.03867176049008e-05**	0.255609695552186	0.281560956841285	6	Des
**2.10596657314106e-05**	0.765128276418354	0.290855043416512	6	Abracl
**2.13068259854052e-05**	-1.48	0.294268573684431	6	Hiatl1
**2.1931674583541e-05**	0.418248949520262	0.302898357673285	6	Gstm2
**2.20276573908222e-05**	0.652188304527384	0.304223976224645	6	Gstm7
**2.20948653224442e-05**	0.639925449943674	0.305152184968277	6	Sparcl1
**2.21810864898578e-05**	0.514134416047484	0.306342985511426	6	Dusp10
**2.22539194301616e-05**	-1.86	0.307348881249962	6	Hhip
**2.23938691946712e-05**	0.826682459720923	0.309281727447604	6	5430417L22F
**2.26110018297253e-05**	0.535706901301612	0.312280546270336	6	Eno1b

**2.31105858331913e-05**	-0.587351067909562	0.319180300942204	6	Clcn4-2
**2.42815085778296e-05**	0.654665443755782	0.335351914968404	6	Ncald
**2.52308662997468e-05**	0.371330319202607	0.348463494465803	6	Ost4
**2.52764044819447e-05**	0.333384787566851	0.349092422300138	6	Pfdn5
**2.54761754954101e-05**	-1.02	0.351851459767109	6	Nras
**2.55168094430932e-05**	0.791302396662854	0.352412655	6	Dhx32
**2.58581593831984e-05**	-0.677716254776219	0.357127039241353	6	Loxl1
**2.63184460934113e-05**	-1.11	0.363484058996104	6	Arhgap21
**2.64799323056115e-05**	0.826188767940425	0.365714345072801	6	Lypla1
**2.69288399888222e-05**	1.39	0.371914209085623	6	Cited2
**2.69879535635627e-05**	0.354861447258672	0.372730626666364	6	Cox5b
**2.73351143201759e-05**	-1.03	0.377525263875949	6	Vegfa
**2.78236237912138e-05**	-0.77	0.384272068180453	6	Baz2b
**2.78554025034223e-05**	-1.89	0.384710963974765	6	Sp100
**2.79287525311196e-05**	-1.65	0.385724001207293	6	LOC1026365
**2.82620296988401e-05**	-0.719547869112082	0.390326892170681	6	Top1
**2.83011261883902e-05**	-1.00	0.390866853787857	6	Lsp1
**2.87075087212507e-05**	-1.13	0.396479402949193	6	Ctdnep1
**2.89009258960899e-05**	0.498155988906928	0.399150687550898	6	Rbm24
**2.93543285745002e-05**	0.830045221564941	0.405412631942422	6	Hint3
**2.97974102033895e-05**	0.48	0.411532032319013	6	BC031181
**3.03563554648391e-05**	-0.964204087570053	0.419251625324893	6	Man2a1
**3.04089704249299e-05**	0.348210937872414	0.419978290538706	6	Rgs7
**3.04134841644988e-05**	0.486167285649192	0.420040629795893	6	Cwc15
**3.06995430748733e-05**	-0.765327355538887	0.423991389407075	6	Daam1
**3.07462398192567e-05**	0.262874133702505	0.424636318143754	6	Prdx1
**3.08657585521422e-05**	0.677241718371286	0.426286991363636	6	Arl4d
**3.11145331820378e-05**	0.88	0.429722817777124	6	B3galnt2
**3.14715535698898e-05**	-1.90	0.434653626353748	6	Ccl11
**3.16260213522879e-05**	-1.50	0.436786980896448	6	Col4a4
**3.16284011060658e-05**	0.412202308044384	0.436819847675874	6	Shroom3
**3.16896215954263e-05**	0.479170950634338	0.437665363854433	6	Nedd8
**3.19657895925084e-05**	0.319274979591435	0.441479520062133	6	Rock2
**3.23508622552554e-05**	0.319967826329058	0.446797758607332	6	Gng5
**3.23814952435327e-05**	-1.64	0.447220830808431	6	Meox2
**3.24954799872807e-05**	-1.23	0.448795074104333	6	Col16a1
**3.26700012799666e-05**	-0.747435394474828	0.451205387677619	6	Herpud1
**3.31973006392846e-05**	-0.869268666617551	0.458487919129159	6	Nfkb1
**3.35422424275761e-05**	0.397600847778492	0.463251910167254	6	Edf1
**3.35522626172178e-05**	0.445874328219201	0.463390299006395	6	Kif1c
**3.54181013969668e-05**	-0.74	0.489159398393508	6	Nfkbiz
**3.54669651480815e-05**	1.29	0.489834255660154	6	Csdc2
**3.65952109927695e-05**	-0.637642881120062	0.505416459021139	6	Tpm3
**3.68388711959554e-05**	-1.48	0.50878165	6	Rab3il1
**3.68885587437662e-05**	-1.59	0.509467884810155	6	Tnfaip2
**3.70461304231373e-05**	0.893730941120634	0.511644107273949	6	Pkig
**3.70722435176135e-05**	-1.51	0.512004755	6	Slc10a6
**3.74848744362807e-05**	0.402647344157971	0.517703600839473	6	Npm1
**3.76198748142828e-05**	0.330274659162628	0.519568091	6	Myl12a
**3.79769880726578e-05**	-1.27	0.524500182271477	6	Dclk1
**3.80293385481176e-05**	0.281282050606314	0.525223194688052	6	Pin1rt1
**3.80596371270776e-05**	0.48	0.525641648362068	6	Park7
**3.81704031065465e-05**	0.381933345317268	0.527171437304513	6	Atp5c1
**3.84711552805979e-05**	1.07	0.531325125580337	6	Anapc7
**3.85291808093819e-05**	-1.86	0.532126516158373	6	Gsta3
**3.8903287152235e-05**	0.524607663863638	0.537293298859518	6	B230219D22I

**3.89613225611784e-05**	0.390820503511762	0.538094825892435	6	Nes
**3.93578529895684e-05**	0.770081147472087	0.543571308	6	Rpl35
**3.96416422991151e-05**	0.344131146407618	0.547490721793078	6	Pdlim5
**4.02170556146427e-05**	0.728642402599894	0.555437755093831	6	Psmb5
**4.0218539034558e-05**	-0.567034392472088	0.555458243	6	Junb
**4.03498617441894e-05**	0.51	0.557271940548999	6	Pygm
**4.08625764577268e-05**	-0.749497368586004	0.564353043457665	6	Aspn
**4.08936282212967e-05**	0.423674992261845	0.564781899364328	6	Hoxb2
**4.14327289473403e-05**	0.355054376934138	0.572227419491717	6	Rps16
**4.15575424594207e-05**	0.980462171984328	0.573951218907059	6	2210407C18I
**4.37352415673928e-05**	0.292006777327776	0.604027421287262	6	Gm1821
**4.60238254866131e-05**	0.596106706975008	0.635635053795613	6	Rnf7
**4.60397668735451e-05**	0.553215766089955	0.635855220290531	6	Rdh14
**4.68437378315156e-05**	-1.38	0.646958863191062	6	Ifit3
**4.81490506235708e-05**	0.455667865275577	0.664986538162137	6	Dhrs3
**4.85103307348887e-05**	-0.807397285139525	0.669976177779547	6	Sepn1
**4.86143409601631e-05**	-1.05	0.671412663000813	6	Anpep
**5.00959164212338e-05**	0.666101592996266	0.691874701693659	6	Zfp771
**5.020582928178E-05**	-1.21	0.693392708210664	6	Flrt2
**5.06814018604547e-05**	0.384575079066634	0.699960841	6	Rps7
**5.07571106254529e-05**	0.899495606028347	0.701006455	6	Pfkm
**5.15177905176023e-05**	-0.756942035161867	0.711512204838606	6	Tubb2a
**5.19456119750166e-05**	-1.76	0.717420846986954	6	Aldh3a1
**5.21583962097824e-05**	0.919587137110021	0.720359610053304	6	Slc4a3
**5.22156308504789e-05**	-0.495020490189066	0.721150077675964	6	Calr
**5.22579993476174e-05**	-0.969031090978325	0.721735228989943	6	Pde3b
**5.37387821455323e-05**	-1.68	0.742186320211947	6	Ildr2
**5.37646055243472e-05**	-1.43	0.742542966896759	6	Ntrk2
**5.40902500983012e-05**	-1.60	0.747040444107638	6	Glt8d2
**5.47649841092052e-05**	0.58	0.756359195532232	6	Atpif1
**5.5074057109544e-05**	-1.80	0.760627802739912	6	Ebf2
**5.52614163037205e-05**	-0.775172811699009	0.763215420570684	6	Tiparp
**5.58807733125674e-05**	-1.62	0.771769360219868	6	Col8a1
**5.6223633336225e-05**	0.468320597278407	0.776504600006604	6	Iscu
**5.63848091031157e-05**	-1.57	0.778730598523131	6	Lhfpl2
**5.64948079986956e-05**	0.982866499780123	0.780249793269984	6	Dtd2
**5.70747361874887e-05**	0.318797628622551	0.788259181485406	6	Rbp1
**5.75863754090983e-05**	0.440477208801652	0.795325430775057	6	Sarnp
**5.78310274505651e-05**	0.856019725541362	0.798704320119754	6	Taf1a
**5.80475515067174e-05**	0.472633014119662	0.801694733859274	6	Chpt1
**5.816332587493E-05**	0.355339954750979	0.803293693658658	6	Tceb2
**5.8721662552655e-05**	0.372272569266122	0.811004881514718	6	Csde1
**5.96826304857955e-05**	0.598826690753148	0.824276809639321	6	Hspa2
**6.03199218883825e-05**	-1.46	0.833078441200451	6	Fgf7
**6.05999321263534e-05**	0.528757264820586	0.836945662597067	6	Grb14
**6.10621391492564e-05**	0.582245762364134	0.843329204	6	Gmppa
**6.12473226723725e-05**	0.584654705950002	0.845886773428137	6	Ndufb6
**6.12915142073957e-05**	-1.14	0.846497102718341	6	Unc5b
**6.1402580077884e-05**	0.718908671670286	0.848031033455656	6	Zfp790
**6.1868227524175e-05**	0.260642814273857	0.854462090336381	6	Tomm6
**6.20907677880424e-05**	-1.56	0.857535593920654	6	Mmp19
**6.26675944825556e-05**	-0.630672135729885	0.865502147398575	6	Cd302
**6.31255077807319e-05**	-1.28	0.871826387959689	6	Stat2
**6.49076655961094e-05**	-1.42	0.896439769547867	6	Prrx2
**6.59130018306531e-05**	-1.72	0.910324468	6	Sult5a1
**6.72999702726489e-05**	0.557116687752131	0.929479889435554	6	Ppm1b

**6.87438212380786e-05**	-0.568783264134197	0.949420915119104	6	Ier3
**7.07831976722466e-05**	-0.674429543565185	0.977586743051397	6	Slc39a7
**7.1674860372117e-05**	-0.837368550410046	0.989901496599308	6	Igf2r
**7.25213220308395e-05**	0.849464166850119	1	6	Dysf
**7.38157534696022e-05**	-1.22	1	6	Ndn
**7.45051236178101e-05**	1.13	1	6	Tmod1
**7.51478366522184e-05**	0.347009632526075	1	6	Cltc
**7.58846260535627e-05**	0.281941985956602	1	6	Rps3
**7.62061017252956e-05**	0.476643951835703	1	6	Col4a2
**7.72056144046589e-05**	1.18	1	6	Plxnb1
**7.96538410610608e-05**	-1.02	1	6	Nfatc1
**7.97414068247136e-05**	0.263086790988543	1	6	Skp1a
**7.97779448628944e-05**	-0.884610033188831	1	6	Wsb1
**8.1506268239159e-05**	-1.76	1	6	S100b
**8.2060558040499e-05**	-1.87	1	6	Ephx2
**8.24325920312866e-05**	0.384800210828468	1	6	Filip1
**8.40281723895479e-05**	-0.507224269831027	1	6	Fosb
**8.53556333502604e-05**	-0.765029897566607	1	6	Zeb2
**8.57429266032733e-05**	-0.499466452601095	1	6	Nbl1
**8.84464054056304e-05**	-1.98	1	6	Clec14a
**8.86538235319422e-05**	0.429429661675304	1	6	Fat4
**8.96492263990816e-05**	-0.834957430704854	1	6	Prr13
**9.03651078040655e-05**	0.574417973740558	1	6	Slc8a1
**9.38295119778306e-05**	-1.11	1	6	Trib1
**9.42858096606352e-05**	-1.14	1	6	Cbfb
**9.69169809116929e-05**	0.464575282033534	1	6	Fyco1
**9.71515484588951e-05**	0.40	1	6	Hint1
**9.71598578454631e-05**	0.543063394625937	1	6	Cobll1
**9.81183555634604e-05**	-0.842423805659453	1	6	Dpysl2
**9.93864677466849e-05**	-1.16	1	6	Lrrk1
**9.94059907649102e-05**	0.660023662708275	1	6	Prkg1
**9.95352419808209e-05**	-0.737768415225611	1	6	Dazap1
**0.000100072261376133**	-1.39	1	6	Sfrp4
**0.00010031846433979**	-0.573594793427798	1	6	Arf6
**0.000100326151938861**	-1.42	1	6	Abcg2
**0.000100675554968261**	0.605348006434303	1	6	Syne1
**0.000101701779894727**	-0.820004955945421	1	6	Rbbp6
**0.000103720375691208**	-0.68	1	6	Hivep2
**0.000105940091497852**	-1.12	1	6	Mettl9
**0.000106275963027808**	-1.65	1	6	Cdk14
**0.000106464235079381**	0.838039973269739	1	6	Rftn2
**0.00010737916105998**	1.14	1	6	Gldn
**0.000109307622691135**	0.354452900173431	1	6	Atp5h
**0.000109755811833194**	0.448601695437015	1	6	Fam118a
**0.000113411148460684**	0.507308928689821	1	6	Minos1
**0.000114510400473035**	-1.26	1	6	Prkcg
**0.000116759227337688**	-0.770054078723579	1	6	Mrc2
**0.0001180256949814**	-1.78	1	6	Bmper
**0.000118408185148924**	0.589387957469386	1	6	Glo1
**0.00011930892093666**	-1.55	1	6	Tshz2
**0.000121215456222947**	0.420201765260873	1	6	Ndufs6
**0.000126681559164876**	0.253459545007014	1	6	Pebp1
**0.000126821611462641**	0.659709573710305	1	6	Ccdc68
**0.000127341796201988**	-0.786053401309027	1	6	Med13l
**0.000127718541978472**	0.452667148915612	1	6	Itga1
**0.000128650277363083**	-0.42	1	6	Egr1

**0.000129062040171803**	-1.50	1	6	Fst
**0.000130419596418214**	0.407149271350274	1	6	Myeov2
**0.000132862539461113**	-0.594177739842992	1	6	Il6st
**0.00013407499350222**	-0.931303163048235	1	6	Agpat2
**0.000135842146689671**	-1.49	1	6	Plscr4
**0.000141140102312563**	-1.35	1	6	Sema6d
**0.000143271205390266**	-0.336614152174284	1	6	Jun
**0.000143380332210853**	0.573760295321587	1	6	Tmtc3
**0.000144271305440538**	-0.70	1	6	Jak1
**0.000144283113336276**	0.578731508954316	1	6	Kcnd3
**0.000145125355411435**	-0.898526610236271	1	6	Trim8
**0.000145355250908854**	-0.42	1	6	Islr
**0.000146467532763097**	-1.84	1	6	Bmx
**0.000146935932534483**	-1.05	1	6	Capg
**0.000148866368382849**	-0.734531242066388	1	6	Ndrg1
**0.000151365304836517**	-1.71	1	6	Stxbp6
**0.000151908377669911**	0.694753606608219	1	6	Uba52
**0.000153530192274998**	-0.775169932431034	1	6	Pid1
**0.000155386046866731**	-1.55	1	6	Sirpa
**0.000155431064125413**	-1.00	1	6	Fam63b
**0.000156908817389962**	0.469191035552192	1	6	2810428I15R
**0.000157998738998405**	-0.527848504766882	1	6	Mfap5
**0.000158571902943118**	-1.18	1	6	Stim2
**0.000159813232907527**	0.878122520246497	1	6	Zfp809
**0.000160278782275279**	-1.82	1	6	3632451O06I
**0.000160966020563969**	0.353234013128358	1	6	Tinagl1
**0.000164173313262107**	-0.934803825534629	1	6	Akap2
**0.000166481619085106**	0.628879776393929	1	6	Fzd7
**0.00016660721820067**	0.518148937174196	1	6	Nek9
**0.000168409936997308**	-1.59	1	6	Tbc1d9b
**0.00017418209570185**	0.351477901926491	1	6	Map1a
**0.000175532939163611**	-0.605774301919974	1	6	Vit
**0.000175652113237653**	-1.26	1	6	Heg1
**0.000176236577244394**	-0.667549302015415	1	6	Sorbs3
**0.000179790362242569**	-0.568799719234612	1	6	Lamp2
**0.000182785027554695**	-0.904196361220101	1	6	Tm7sf3
**0.000186394392853253**	0.430080915361625	1	6	Atp6v1a
**0.00018805077129372**	-1.08	1	6	Ncoa7
**0.000189033155367242**	0.569093782701712	1	6	Cd97
**0.000189034410078858**	0.459859953861425	1	6	Ptprs
**0.000190392435493824**	-0.824693260249945	1	6	C1qtnf1
**0.000193827256168582**	-0.904832051549731	1	6	Slc41a1
**0.000196468628863157**	-0.702695158835011	1	6	Mxra7
**0.000200402680840506**	-0.854411912966328	1	6	Pqlc1
**0.000204386314508188**	0.853061731210661	1	6	Nacc2
**0.000204549851601672**	-1.53	1	6	Farp1
**0.000205557092977338**	-0.671103340790206	1	6	Tram1
**0.000206064419081291**	0.250909536238226	1	6	Prkar1a
**0.000206244709661371**	-0.654009461261252	1	6	Mycbp2
**0.000208393282553555**	-1.28	1	6	Sphk1
**0.000209693058982174**	0.398494391426263	1	6	Atp8b1
**0.000209940700428386**	-1.23	1	6	Baiap2
**0.000211409361088785**	0.352260129751108	1	6	Naa20
**0.000214362601570542**	0.518472817646082	1	6	Cux1
**0.000215096819974018**	0.433430809140627	1	6	Tacc2
**0.00021708017039399**	-1.11	1	6	Dpy19l1

**0.000217225631302342**	-0.856504562033026	1	6	Abhd5
**0.000219638733316666**	0.400814867986485	1	6	Dact3
**0.000222519277215451**	0.680904558002813	1	6	Mtus1
**0.000223420211772631**	0.731681131193736	1	6	Wscd2
**0.000224198378364347**	0.389709270435861	1	6	Tgm2
**0.000226121623084668**	0.611300091806564	1	6	Pttg1
**0.00022657354061841**	0.799403173119028	1	6	Hspe1
**0.000227309639719386**	-0.728268303521411	1	6	Tubb6
**0.000230333926807581**	0.663528433484692	1	6	Pfdn1
**0.000230723411517353**	0.631706536601319	1	6	Acadm
**0.000232723899614054**	-2.06	1	6	Tek
**0.000233962092633561**	-0.759655513969881	1	6	Creld2
**0.000235861283770577**	-0.941870025458263	1	6	Zcchc6
**0.000236602428294538**	-0.572011243010432	1	6	Col6a1
**0.000241529178077221**	0.772952571525883	1	6	Tfrc
**0.000242551650562787**	0.454223653270453	1	6	Oat
**0.000243530177480702**	0.698557655369974	1	6	Zmym6
**0.000247735735667078**	-1.29	1	6	Ccbe1
**0.000252954643184629**	1.17	1	6	Trak2
**0.000254523057373596**	-1.39	1	6	Ets1
**0.00025791222488097**	0.801934436672974	1	6	Cacna1c
**0.000258523826417043**	0.587934842320152	1	6	Rpl15
**0.000260212005123295**	0.628127311825714	1	6	Synm
**0.000266233271833918**	0.713325939121195	1	6	Zfp367
**0.000268239577188777**	-0.761359472817504	1	6	Nin
**0.000271713804731995**	-0.37	1	6	Os9
**0.000273995571108665**	1.03	1	6	Bche
**0.000274602663612463**	-1.25	1	6	Twist1
**0.000274756355787142**	0.536808129740987	1	6	Vdac2
**0.000276587185029169**	0.877691644920552	1	6	Surf2
**0.000280474758777907**	0.700681593545001	1	6	Gamt
**0.000282810795685244**	-0.46	1	6	Rrbp1
**0.000293095738794008**	0.473764291796298	1	6	Sun2
**0.000293236272009877**	0.85	1	6	Manbal
**0.000298007603194735**	-1.61	1	6	AI607873
**0.000298627796146836**	0.331188093370797	1	6	Hsbp1
**0.000299291140661207**	-0.413487635707523	1	6	Smarca2
**0.000300676019477117**	0.826114960639156	1	6	Jam3
**0.000300785955851385**	0.449479030953399	1	6	Tax1bp1
**0.000303529502487136**	0.790727913069936	1	6	Fahd2a
**0.000309599885623829**	0.838224040157334	1	6	Bri3
**0.000309784216729635**	0.29	1	6	Rpl39
**0.000313373810116116**	-1.10	1	6	Tnxb
**0.000313507827743661**	0.346371518796759	1	6	Slc25a3
**0.000313994567123355**	0.607014893789574	1	6	Fam162a
**0.000314297078025042**	0.408127539362689	1	6	Psmb1
**0.000315910386652251**	0.559692300284007	1	6	Abtb2
**0.000316130524560968**	0.474901111921116	1	6	Anxa6
**0.000320919674161436**	0.501765327377107	1	6	Ndufb2
**0.000321636149890572**	-0.962489668681438	1	6	Irf9
**0.000321886007547054**	-1.63	1	6	Xaf1
**0.000321929931875878**	-1.96	1	6	Lmo2
**0.000321963248104255**	-0.594390166247729	1	6	Ddah2
**0.000326468034124602**	0.477440268138808	1	6	Cirbp
**0.000328035957620828**	-1.22	1	6	Gas7
**0.000332109937483641**	0.600351355767062	1	6	Tcp1

**0.000333336199761523**	-0.672942496471462	1	6	Sra1
**0.000333650174213258**	-0.682500864943683	1	6	Arid1b
**0.000334578735683065**	0.326881910586337	1	6	Fry
**0.000336693546276056**	0.376061162952591	1	6	Rheb
**0.000339933820496183**	-1.92	1	6	Lcn2
**0.000341349384834302**	0.588092121292785	1	6	Thoc7
**0.000346663226134331**	-1.38	1	6	Papss2
**0.000347645876334911**	0.694607461193271	1	6	Rhobtb1
**0.000347723617338093**	-1.05	1	6	Atp6v1b2
**0.000350220557894509**	-0.631194330940307	1	6	Acap2
**0.000355422523496636**	0.724013968868887	1	6	Gmpr2
**0.000360112432014239**	0.610455639357045	1	6	Cdk2ap2
**0.00036377669621556**	-1.26	1	6	Slc22a17
**0.000365828115576329**	-0.761678983512419	1	6	Zcchc11
**0.000371065012093796**	-0.996170124490676	1	6	Ndufa4l2
**0.000377628111423648**	-0.623065498423667	1	6	Ppp1r10
**0.000381180112268513**	-0.622921033423011	1	6	Kazald1
**0.000382438227099272**	0.888497185096305	1	6	Gbe1
**0.000384309898846438**	-0.661566564504482	1	6	Fam46a
**0.000386886388621924**	0.87	1	6	BC005624
**0.000388007069669542**	0.517406313393244	1	6	Esd
**0.000388426995280145**	-0.886523771734632	1	6	Zswim6
**0.000389824604590376**	-0.762431712189285	1	6	Pld1
**0.000391106460882877**	-1.88	1	6	Col2a1
**0.000397627428579937**	-0.984941581028601	1	6	Alkbh5
**0.000401238581730617**	-0.920740801850544	1	6	Art4
**0.000404839441232128**	-1.26	1	6	Kcne4
**0.000405049058848017**	-1.19	1	6	Sos1
**0.000406963855542411**	0.823354279616497	1	6	Rab3a
**0.000412558706484097**	-1.11	1	6	St3gal1
**0.000415733132140955**	0.391282945820642	1	6	Rps27l
**0.000416871469394799**	-1.26	1	6	Thbs1
**0.000418527031881928**	-0.669226163348237	1	6	Rsl1d1
**0.00041889557068194**	0.30	1	6	Ttc39b
**0.000421783360440227**	0.551274904651834	1	6	2010107E04R
**0.000429167283250961**	-0.735870062770104	1	6	Hand2
**0.000429682927241405**	0.630151491090843	1	6	St3gal5
**0.000430361992743766**	0.49	1	6	Prkag2
**0.000430928806484209**	-0.976738445456361	1	6	Epc2
**0.000432178422572323**	0.54	1	6	Spag7
**0.000432522583524241**	0.536691106869041	1	6	Mlf2
**0.000432765746466035**	0.389470286811177	1	6	Atp1a2
**0.000432832924107692**	-1.44	1	6	Fam19a5
**0.000437190757012079**	0.630845335309498	1	6	Ift46
**0.00044189807247795**	-1.44	1	6	Ssc5d
**0.000442584769315221**	-0.610375777299413	1	6	Arl5b
**0.0004426105340493**	0.443259765261591	1	6	9530068E07R
**0.000443393723433493**	0.426860409382944	1	6	Fra10ac1
**0.000447845488947989**	0.728272305730597	1	6	Sort1
**0.000450092130169939**	-0.66	1	6	Ripk1
**0.000451834987653593**	-1.32	1	6	Ccl19
**0.000451931484527733**	-0.506440839532188	1	6	Txnrd1
**0.000453483681634342**	0.336437592370423	1	6	Spcs1
**0.000457644838672519**	0.524394540163118	1	6	Stip1
**0.00046995210714588**	-1.00	1	6	Tcf12
**0.000470100199509542**	-0.871073240857857	1	6	Copz1

**0.000470532047019837**	-1.43	1	6	Isg20
**0.00048442876564987**	0.712021073663776	1	6	Ndufb3
**0.000493186380189935**	-1.19	1	6	Pcdh18
**0.000495637702398063**	0.495223082989361	1	6	Lamtor5
**0.000503427088542977**	-2.10	1	6	Icam2
**0.000503933873261032**	-1.32	1	6	Smarcd2
**0.00050469984297583**	0.25	1	6	Macf1
**0.000505962184434242**	-1.36	1	6	Naalad2
**0.000506176520529712**	0.433809765123228	1	6	Slc25a5
**0.000515485048627598**	-0.90	1	6	Elk3
**0.000515561554396495**	0.580506647591143	1	6	Ech1
**0.000516881897507091**	-0.519038970027357	1	6	Srrm2
**0.00051726416879015**	-0.616516972633192	1	6	Arpc5
**0.000520287277544671**	-1.26	1	6	Abcb1b
**0.000522243691034594**	0.335792153938801	1	6	Vdac1
**0.000537550100258193**	0.481576169148179	1	6	Pmvk
**0.000541451645653476**	-1.09	1	6	Ptgir
**0.000548490897954446**	-1.37	1	6	Angptl1
**0.000549976687243876**	0.269272345248984	1	6	Cav2
**0.000551108290736956**	-0.975800579033571	1	6	Dchs1
**0.000551190732642394**	0.44	1	6	Pgrmc1
**0.000553660105787248**	-0.655812703891946	1	6	Smad4
**0.000554144846817422**	-0.636098124670072	1	6	Eif4g3
**0.000555702400892751**	0.819536404349084	1	6	Pon3
**0.000572952508649484**	-1.18	1	6	Atp1b1
**0.000575427730685383**	-0.77	1	6	Scarf2
**0.000576490140467943**	-1.73	1	6	Procr
**0.000576519092037379**	-1.61	1	6	Enpp3
**0.000576782794500058**	-0.854727356620305	1	6	Sfxn1
**0.000577352562574584**	-1.08	1	6	Tenm3
**0.000581253249381571**	0.618845182021713	1	6	Ppp1r12c
**0.000582461830707683**	1.03	1	6	Tctex1d2
**0.000587904507214284**	-0.414267859368751	1	6	Serpinb6a
**0.000590324792022028**	-0.476170124448644	1	6	Ghr
**0.000591321021472997**	0.461037379481844	1	6	Vasp
**0.000598859600739637**	-1.04	1	6	Dnmt3a
**0.000601022224622058**	-0.665970906871809	1	6	Gpbp1
**0.000607738071542934**	0.577882180438227	1	6	Rps29
**0.000613879321737932**	0.389014750409195	1	6	Nkiras1
**0.00061732369002865**	0.48	1	6	Bloc1s1
**0.0006234253682006**	0.401170092547261	1	6	Paip2
**0.000630876646326724**	-0.634347305582358	1	6	Emilin1
**0.000633936123872627**	0.662168431803105	1	6	Arl4a
**0.000634461272377083**	0.360584127228367	1	6	Ano10
**0.000634535075085975**	-0.59	1	6	Atxn7l3b
**0.000635741897419194**	-1.09	1	6	Lsamp
**0.000639896870244942**	-0.928058141668742	1	6	Naglu
**0.000650838832581176**	-0.380561738204351	1	6	App
**0.000652889780447437**	0.550018757635725	1	6	Psmb3
**0.000653324576630561**	-0.877970918588878	1	6	Pla2g16
**0.000653952801347966**	0.467412848461894	1	6	Psmc1
**0.000659274744481896**	-0.451377498537449	1	6	Fkbp2
**0.000661403954986627**	-0.655456179149887	1	6	Nfe2l2
**0.000661431345583519**	0.285585495420233	1	6	Psma7
**0.000667456625033693**	-1.40	1	6	Shisa3
**0.0006759416999001**	-0.682094391978409	1	6	Lamc1

**0.000676253006510603**	0.496446132534267		1	6	Eif3g
**0.000676822198548891**	0.399456590623184		1	6	Sgcb
**0.000677499976238689**	-0.644613169945674		1	6	Acbd3
**0.000678397244152892**	0.718511373293983		1	6	Gne
**0.000678732169407546**	-1.06		1	6	Man2b2
**0.000679142121653784**	-0.662231117367405		1	6	Mpp6
**0.000688779081445082**	-0.623170721184018		1	6	Pde4b
**0.000695647491382204**	0.410940164167028		1	6	Cox7b
**0.000702795987109463**	-1.02		1	6	Sec14l1
**0.000710573224192994**	-0.893975802109582		1	6	Trove2
**0.000713345421698309**	0.541480428236219		1	6	Ran
**0.000720942963284401**	0.484127814580692		1	6	Cd59a
**0.000722185222939286**	-0.97		1	6	Birc2
**0.000722879443897679**	0.33		1	6	Pnpla2
**0.000748269118208113**	-0.420351743830951		1	6	Atp6ap2
**0.000752353171238722**	-0.698120159708725		1	6	Ankrd17
**0.000757712359718234**	0.46		1	6	Wtip
**0.000764029083478335**	0.369461406424789		1	6	Mob4
**0.00076583322578119**	-1.22		1	6	Myo6
**0.000766956376281528**	-1.51		1	6	Rtp4
**0.000768525202154249**	-0.663874750765471		1	6	Cebpb
**0.000769154691074487**	-0.300612729752579		1	6	Lpgat1
**0.000771169719185911**	-1.92		1	6	Cd93
**0.000776028561552727**	-1.18		1	6	Arhgap31
**0.000783837575512002**	-0.831672425996839		1	6	Zfc3h1
**0.000786478397462217**	0.968363431230193		1	6	Prps2
**0.000788016201348333**	-1.13		1	6	Atf5
**0.00079157718633877**	-0.720984077768752		1	6	Sh3d19
**0.000798291019368259**	-1.21		1	6	Kctd12
**0.000798799906638179**	-0.452344479873483		1	6	Prrc2c
**0.000802789948752398**	-1.34		1	6	Pecam1
**0.000808351854497182**	0.302901503890747		1	6	Tomm7
**0.00081546362314434**	-0.753801308125577		1	6	Dpagt1
**0.000819308743416604**	-1.38		1	6	Nsg1
**0.00083530224674319**	-1.52		1	6	Gprc5b
**0.00083720713977826**	0.88		1	6	Cisd1
**0.000837530077855315**	-0.381703041558725		1	6	Mbd1
**0.000852113133109959**	0.364003851320845		1	6	Srf
**0.000852189534823186**	0.332084728033207		1	6	Trappc8
**0.000854102125405419**	0.503235667226517		1	6	Dnajc8
**0.000856233874265991**	-1.23		1	6	Spry1
**0.000866520804636588**	0.292720023513221		1	6	Tbca
**0.000874500794060404**	0.52		1	6	Rps25
**0.000880874935158213**	-0.53		1	6	Mbtps1
**0.000883819718980682**	-0.452167508977654		1	6	Qpct
**0.000885208131550177**	-0.403161945539925		1	6	Pdia3
**0.000885330191477127**	-0.883111767438964		1	6	Rab23
**0.000886986732289775**	-0.70		1	6	Rbm28
**0.000893718429631101**	-0.943826954654887		1	6	Cebpg
**0.000898751357999827**	0.764357761419402		1	6	Higd2a
**0.000899955707607283**	-1.04		1	6	Trim2
**0.000902964541368591**	0.312813722483212		1	6	Grcc10
**0.000904024176334895**	0.536512850515426		1	6	Pdgfd
**0.000921582146987716**	-0.377566130768909		1	6	Dnajb11
**0.000923092229761494**	-1.03		1	6	Rasa1
**0.000926872767501546**	0.658848752747026		1	6	Xirp1

**0.000930477675822095**	0.95	1	6	Mif4gd
**0.000937724687233155**	0.524672135889036	1	6	Mgat4a
**0.000943526644056845**	1.07	1	6	Tmem80
**0.000949461257573404**	-0.447774435426549	1	6	Kmt2e
**0.000952697115404364**	-0.917971195131708	1	6	Prpf40a
**0.000961827994174035**	0.526981539302702	1	6	Deb1
**0.000963382681680552**	-0.845875156551462	1	6	Nnmt
**0.00096450827060335**	-0.960391469113584	1	6	Per1
**0.000969523894884298**	-1.24	1	6	Piezo1
**0.000970031387407205**	-0.99	1	6	Usp24
**0.000979766794097374**	0.650275942503181	1	6	Lxn
**0.000980362465580868**	-0.597476633929037	1	6	Adamts10
**0.000984719383130494**	0.26	1	6	Fau
**0.00098784416600125**	0.376143181424679	1	6	Sh3pxd2a
**0.000990639515970258**	0.76	1	6	Rnd3
**0.000997097101907073**	-0.832294588717727	1	6	Sh3kbp1
**0.00100541802867534**	0.419731723071256	1	6	Bnip3
**0.00101097047383424**	-1.18	1	6	Lamc3
**0.00101418261348082**	-0.991402373537819	1	6	Irgm1
**0.00101754370544357**	-1.05	1	6	Elmsan1
**0.00102061210979455**	-0.853026741267882	1	6	Lifr
**0.001023859696475**	-0.518437032797755	1	6	Shmt2
**0.00104013681779107**	0.315095536713552	1	6	Txn1
**0.00104961483509979**	-0.58	1	6	Klf9
**0.00105154077334714**	-0.286440969901524	1	6	Laptm4a
**0.00105560020912381**	0.505713814951072	1	6	Maf
**0.00105843089347569**	-1.49	1	6	Gfra2
**0.00106264198626619**	-1.46	1	6	Aff3
**0.00106475751391485**	0.306297660615514	1	6	Rpl37a
**0.00107256287059056**	-0.811431844140182	1	6	Stx5a
**0.00107274280537434**	-0.601761433384637	1	6	Hexb
**0.00107377043741153**	-0.775283743928064	1	6	Grb10
**0.00107820024249404**	0.485497592035004	1	6	Mdh1
**0.00107912632492618**	0.349062338018161	1	6	Bcas2
**0.00108644634224356**	-1.16	1	6	Pcdh9
**0.0010867852341184**	-0.403060246720889	1	6	Fgfr1
**0.00110555414045684**	-1.05	1	6	Iigp1
**0.0011244702110192**	-1.42	1	6	Slc16a9
**0.00112902981851758**	0.33	1	6	Ktn1
**0.00114146704470834**	-1.37	1	6	Slc9a3r2
**0.00114749958567455**	-0.822626562181038	1	6	Fkbp10
**0.00115143820002865**	0.691281334678593	1	6	Mrpl28
**0.00115392597716459**	0.555744212443314	1	6	Ube2d2a
**0.00115501598442282**	-0.865690540193241	1	6	Acsl5
**0.00115667462387196**	0.333201475896899	1	6	Afap1
**0.00118074690109646**	-0.964885542899691	1	6	Ppp1r14b
**0.00119540996102854**	0.48	1	6	Nxt2
**0.00120065265555956**	0.57	1	6	Srp14
**0.00120349066355601**	-0.341578383414261	1	6	Ltbp4
**0.00121099953488521**	-0.675623620511562	1	6	Glul
**0.0012127293858966**	0.456496058839454	1	6	Hipk1
**0.00121358975836849**	-0.673328899087873	1	6	Trim35
**0.00121667452651718**	-0.789997541271125	1	6	Pld3
**0.00122380825703676**	0.455365536263495	1	6	BC002163
**0.0012257352581729**	-0.698447999216968	1	6	Peak1
**0.00122617766057626**	-1.68	1	6	Enc1

**0.00124806044757212**	-1.52	1	6	Adcy4
**0.00124987734945323**	0.607163556872898	1	6	Mrpl51
**0.00125796427332197**	0.592848024493704	1	6	Ehbp1l1
**0.00126212120485007**	-0.396038984403706	1	6	Acvr1
**0.00126489212122302**	-0.536774743107474	1	6	Lrp6
**0.0012652133353535**	-1.52	1	6	Tmod2
**0.00127115073245961**	0.613997483553056	1	6	Mrps24
**0.00128333919737131**	0.715452938074244	1	6	Unc45a
**0.00130559711493383**	-0.541483848363618	1	6	Antxr1
**0.00134306304309273**	0.578569996648961	1	6	2700060E02F
**0.00134963224212789**	-0.755133488224217	1	6	Fndc3a
**0.00136868599028128**	0.808168482522488	1	6	Gm561
**0.00137307086746607**	-0.958536885492759	1	6	Tex261
**0.00138287777501272**	0.415692028042356	1	6	Chd6
**0.001392169072874**	0.372312900791264	1	6	Dek
**0.00139746215247723**	0.32	1	6	Psmb2
**0.00141418739962654**	-0.885524521089408	1	6	Tm9sf4
**0.00141436748344387**	-0.598254697116402	1	6	Mcfd2
**0.0014246411839223**	0.272275639095025	1	6	Rabac1
**0.00146324229432715**	-0.428989551253498	1	6	Tmed3
**0.00147138206573298**	0.557133995455755	1	6	Prkaca
**0.00148445630209519**	-0.423546787539217	1	6	Nucks1
**0.00148729659944999**	-0.962634619851267	1	6	Usp2
**0.00148798833615253**	0.534209305952828	1	6	Fam188a
**0.00149393971347111**	0.336437124878087	1	6	Fam199x
**0.00149673178451134**	1.30	1	6	Ubxn7
**0.00149817389006773**	-0.913830737348445	1	6	Fbxw8
**0.00151188021917754**	0.309283018473968	1	6	Mbnl2
**0.00151874254153047**	1.14	1	6	Vps37b
**0.00152349324347993**	-0.744387088404367	1	6	Ifih1
**0.00153301027673643**	-1.69	1	6	Tspan11
**0.00153486065615069**	0.357627896398524	1	6	Ano1
**0.00153590507952522**	-1.33	1	6	Terf2
**0.00155910345826036**	-1.14	1	6	Neat1
**0.0015883569219499**	0.365791045293291	1	6	Ivd
**0.00159585816525162**	-1.20	1	6	Ndst1
**0.00160629889620279**	0.492547668847998	1	6	Zbtb8os
**0.00161099336332217**	0.367189340285801	1	6	Tgfbr1
**0.00161827848928735**	-1.08	1	6	Cyp2j6
**0.00162364498403347**	-0.376521893546771	1	6	0610031J06F
**0.00162750027792792**	-0.610905734796126	1	6	Leprel2
**0.00164828664788721**	0.496195102784008	1	6	Tbc1d1
**0.00165040126297079**	0.370803213345257	1	6	Itpr1
**0.00165437312905872**	0.752989841748472	1	6	Trp53inp2
**0.00166524439507773**	0.386630834613907	1	6	Wbp2
**0.00166708209476725**	0.600033711117786	1	6	Ngfrap1
**0.00168868244035841**	-1.03	1	6	Setd5
**0.00169599139168525**	-0.466086684120271	1	6	Hn1
**0.00173023021474624**	-0.520826784019858	1	6	Col5a2
**0.00174237542239042**	0.291459040181878	1	6	Lats2
**0.00174249128540674**	0.526343782967502	1	6	Mrpl42
**0.00176641103060274**	-1.76	1	6	Tmem158
**0.00177380579025676**	0.646995825514582	1	6	Skap2
**0.00178013578018229**	0.77	1	6	Tmed4
**0.00179562703146526**	-0.820049418720629	1	6	Numb
**0.00180757915402375**	-0.967022292674386	1	6	Ptpn1

**0.00181748489046187**	-0.899270859113894	1	6	Plat
**0.00181894491278255**	-0.49	1	6	Phldb1
**0.00182652990914259**	-0.81	1	6	Zmym2
**0.00182698917204657**	-1.08	1	6	Phlda3
**0.00183097099168518**	0.259987745273699	1	6	Arpc4
**0.00185254027789489**	-1.61	1	6	Vwf
**0.00187552516312989**	-1.42	1	6	Bdh2
**0.0018800306523809**	-0.677096094903199	1	6	Mettl7a1
**0.00188115339603737**	-1.76	1	6	Ccnd1
**0.00189237215217924**	-0.607895244606057	1	6	Slc7a1
**0.00189495735540076**	-0.818406416196256	1	6	Acsl4
**0.00190755589998996**	-0.803331318682628	1	6	Bclaf1
**0.00190785012093223**	0.39	1	6	Hyal1
**0.0019098979973911**	-1.53	1	6	4632428N05R
**0.00191527427362976**	-0.902870810256203	1	6	Tmem55b
**0.00192550847956516**	-0.591211379583932	1	6	Vat1
**0.00192665044643129**	-0.673797634127727	1	6	Kcmf1
**0.00193455288509936**	-1.36	1	6	Tmem140
**0.00193942623932808**	0.506680886423788	1	6	Hsph1
**0.00194491853209381**	-0.559239656670352	1	6	Thbs3
**0.00195876965535962**	0.981643995706568	1	6	Stk16
**0.0019790451766975**	-0.744688858070184	1	6	Ibtk
**0.00199351801466623**	0.454330716535963	1	6	Commd3
**0.00202439811413576**	-0.711922228281359	1	6	Mia3
**0.00202795660849034**	-0.684866757001197	1	6	Fam149a
**0.00203205783612785**	-0.858511492510471	1	6	Lrrc32
**0.00204379595133733**	0.464308908945904	1	6	Med28
**0.00204616230839566**	0.311300072942273	1	6	Col4a5
**0.00205250593810225**	0.790134251944447	1	6	Snx4
**0.00206741620605034**	-0.682019976696453	1	6	Olfm1
**0.00209766048391577**	0.433969342993941	1	6	Ccm2
**0.002111480205959**	-0.708079993928142	1	6	Fam178a
**0.00214075195423291**	-0.575635524554708	1	6	Slc50a1
**0.00215712036253334**	-1.08	1	6	Loxl2
**0.00215891051268634**	0.58	1	6	Bmpr1b
**0.00215975466153581**	0.419382729503565	1	6	Cuedc1
**0.00217469888268513**	0.656943257259888	1	6	Eml1
**0.00219085675305728**	0.655165586664179	1	6	Pcif1
**0.00220455983603046**	0.404553705219727	1	6	Cep104
**0.00220798469228703**	-0.568133108899204	1	6	Zbed6
**0.00220820925481247**	-0.703799512143943	1	6	Stox2
**0.00221925342773239**	-0.713674581664623	1	6	Midn
**0.00223277571877807**	0.256825754628787	1	6	Hif1a
**0.00227224860970693**	-1.48	1	6	Sele
**0.00227303523901936**	-1.94	1	6	AW112010
**0.00228777322595646**	0.440757439726146	1	6	Selt
**0.00228924610695441**	-1.48	1	6	Arhgap20
**0.00230554320594371**	-0.47	1	6	Tmem205
**0.00230612637866718**	-0.988424732261967	1	6	Ephb3
**0.00232132437911238**	0.425990687660907	1	6	Psmd4
**0.00234399480476428**	-0.977683109710015	1	6	Usp33
**0.00235610454493052**	-0.524416936455973	1	6	Bptf
**0.00235799532844326**	-1.06	1	6	Mid1ip1
**0.00236620144436382**	0.349447461197837	1	6	Atp6v1f
**0.00236665116421691**	0.502159495415002	1	6	Vamp2
**0.00237325955846357**	0.484991210523118	1	6	Uqcrq

**0.00237887992064754**	0.504250455234726	1	6	Trmt112
**0.00239088601938844**	0.487910359624429	1	6	Pold4
**0.00240068986749186**	-1.76	1	6	Has1
**0.00240722211292431**	0.651315866796274	1	6	Mrps18c
**0.00241360725157496**	-0.804884526699702	1	6	Plod1
**0.00243184836279941**	-1.07	1	6	Slc7a2
**0.00244283558205295**	-0.867410676024137	1	6	Adam17
**0.00244305963524131**	-1.09	1	6	Arhgef40
**0.00245337564183107**	-0.661292256514996	1	6	Kcnj8
**0.00248184860253128**	0.438216169345043	1	6	Cct7
**0.00252034210013589**	1.01	1	6	Fem1a
**0.00254020997203172**	0.289733630138134	1	6	Slc48a1
**0.0025500957049595**	0.266454411863382	1	6	Aktip
**0.00255304961592104**	-1.36	1	6	Sbno2
**0.00255680894685324**	0.459283946255752	1	6	Plekha3
**0.00258728922006918**	0.299285108634267	1	6	Rnf44
**0.00262621274735338**	-0.96	1	6	Cln5
**0.00263487648617845**	0.553321973938815	1	6	Snhg9
**0.00263588067161487**	0.605040833954541	1	6	Med9
**0.00263717771329513**	-1.45	1	6	H2-Ab1
**0.00265995395772226**	-2.17	1	6	Egfl7
**0.00268875048875499**	0.768925548329507	1	6	Marf1
**0.00269115541093616**	0.718984735063727	1	6	Dpp3
**0.00269868868722609**	-1.50	1	6	Ifi203
**0.00270594075545345**	0.267336870491271	1	6	Rasl11a
**0.00270967460206983**	-1.58	1	6	AI507597
**0.00273657446235219**	-0.804183657500146	1	6	Arnt
**0.00275797024723498**	0.653840040718049	1	6	Mrpl21
**0.00277449931121312**	0.458637603456131	1	6	Ptges3
**0.0028189229404537**	-0.996714935182441	1	6	Sp1
**0.00282402165312065**	0.764151384367797	1	6	Zfp467
**0.00284340281984655**	0.287721608613536	1	6	Nsa2
**0.0028442507353923**	0.736084077620484	1	6	Arl2
**0.00284753826597096**	0.792465539846235	1	6	Thyn1
**0.00286653324787446**	-0.908147001540814	1	6	Cdk16
**0.00286969183883499**	-0.923107044574174	1	6	Cndp2
**0.00286994790346451**	-1.15	1	6	Chst11
**0.00287906350070843**	-1.27	1	6	Il4ra
**0.00296158626339964**	0.683205765017719	1	6	Plcb3
**0.00297556171292694**	0.321878349701585	1	6	Sharpin
**0.00298222861253378**	0.395120307971681	1	6	Tpt1
**0.00298553370343396**	0.996455459051983	1	6	2610002J02F
**0.00298706001152424**	0.287874802268599	1	6	Akr1a1
**0.00298938858228805**	-1.02	1	6	Cpeb1
**0.00302154336212689**	0.388088286758818	1	6	Mgll
**0.00307907223871081**	0.290774705226737	1	6	Pbrm1
**0.00309892908174178**	0.446584136247557	1	6	Ecm2
**0.00315437305532029**	-0.511198937744598	1	6	Zcchc24
**0.00317305944527798**	0.551374710031427	1	6	Parp8
**0.00318288811803808**	-0.798901957293918	1	6	Slc25a25
**0.00319138032985004**	-0.773596140939471	1	6	Ube2l6
**0.00319678143551945**	0.486599523393425	1	6	Cops6
**0.00321025339288414**	0.546397290674469	1	6	Me2
**0.00321058609504554**	-1.59	1	6	Cxcl14
**0.00321875678531788**	-0.426260081268238	1	6	Derl1
**0.00325510927122398**	-0.321120782043335	1	6	Atf4

**0.00328229694745544**	0.355596516490124		1	6	Dnajc10
**0.00333018493676837**	-0.50		1	6	Arglu1
**0.00333598995750896**	-0.682765343311807		1	6	Serpinb9
**0.00340313300747039**	0.462488660146955		1	6	Hspb8
**0.00340461499249459**	-0.520018943517071		1	6	Prrc1
**0.0034134305537123**	-0.861271285423824		1	6	Smad3
**0.00342519802390464**	0.519530114709307		1	6	Timm17a
**0.00344763076602882**	-0.55		1	6	Ckap4
**0.00346983021328087**	-0.510854561018274		1	6	Zmiz1
**0.00348171211449414**	-0.500376882318496		1	6	Dnttip2
**0.00351212800797506**	0.655109391419406		1	6	Nr1h2
**0.0035170987525664**	-0.29		1	6	Tecpr1
**0.00353867381907694**	-0.501423352052865		1	6	Fech
**0.00357150860756184**	-1.29		1	6	Fnbp1l
**0.0036169224792459**	0.293284561358493		1	6	Actr3
**0.00362605873855113**	-1.57		1	6	Itm2a
**0.00364080630964804**	-0.488147467451038		1	6	Rab6b
**0.00367159089291101**	0.412545247819389		1	6	Cdc37l1
**0.0036716128984547**	-1.20		1	6	Tmem173
**0.00368540777047627**	0.472697052100347		1	6	4930503E24F
**0.00372199443850755**	-0.663970692212096		1	6	Arpc1a
**0.00376518442686947**	0.61		1	6	Mycbp
**0.00376638643238482**	-1.44		1	6	Tbx20
**0.00378383634284425**	-0.485584775208895		1	6	Rela
**0.00379554590598214**	-0.395961727753718		1	6	Wdr26
**0.00381529379604339**	0.333958544661117		1	6	Wdr83os
**0.00382560077103037**	0.578610634971657		1	6	Hibadh
**0.00383530076372211**	-0.656987587986617		1	6	Rnf145
**0.00383811712152391**	-1.07		1	6	Dram1
**0.00386753038979684**	-1.23		1	6	Ptgs1
**0.00389589352963876**	-0.313077211708022		1	6	Timp3
**0.00391031563823835**	-0.408170643380502		1	6	Lrig1
**0.00391128873976283**	0.767394319208728		1	6	Crtc3
**0.00395854009487157**	-0.338815360097175		1	6	Ddost
**0.0039675616128289**	0.287651233160465		1	6	Atp5f1
**0.00398370333094117**	-0.279174852717906		1	6	Rhbdf1
**0.00398886882400151**	0.817602275938214		1	6	Rwdd4a
**0.0039987541928574**	0.314406576722069		1	6	S100a1
**0.00401525637098829**	-0.82		1	6	Notch1
**0.00402170103460274**	-1.05		1	6	Dact1
**0.0040379980408065**	0.329300642874121		1	6	Rpl10
**0.00405877502584773**	0.62		1	6	Tada3
**0.00406818682059378**	-0.470985702797329		1	6	Mmp23
**0.00412293217796149**	-1.22		1	6	Glb1
**0.0041519039863911**	0.534859682519143		1	6	Banf1
**0.00417248655655858**	0.549088793194354		1	6	Vps25
**0.00417572276188165**	-0.499592582877906		1	6	Nipbl
**0.00418900334610308**	-0.840277186403464		1	6	Snap47
**0.0043028304496053**	-1.15		1	6	Siah2
**0.00431632982496613**	0.639904956758345		1	6	Mrpl32
**0.00432995735477577**	0.476156588415628		1	6	Idh2
**0.00435762820803291**	0.868264533720019		1	6	Srrd
**0.00443238880055596**	-0.484228357705927		1	6	Dnajc3
**0.00445057163509788**	0.59		1	6	Cnot10
**0.00445927646756999**	-0.295114618911438		1	6	1300002E11F
**0.00447076744718999**	-0.490993522436299		1	6	Pnrc1

**0.00447522218708193**	-0.846999933548544	1	6	Mdfic
**0.00447588392614342**	-1.16	1	6	Map3k3
**0.00447762477586445**	-0.571534152519337	1	6	Map4k5
**0.00448995504628113**	0.537137125405103	1	6	Arc
**0.00454077523957365**	-0.914067824307503	1	6	Sdccag3
**0.00454782679228736**	0.438838536817386	1	6	Pias1
**0.00458232739312023**	-0.685539969886912	1	6	Sptlc2
**0.00460549451822498**	0.616265083676059	1	6	Rbbp9
**0.00464098927964555**	0.340448442761836	1	6	Pitpna
**0.00466734612587196**	-0.523173829131219	1	6	Srm
**0.00467901239143247**	-0.949092389734478	1	6	Zfp655
**0.00468945877989671**	0.74	1	6	Mrpl18
**0.00472815161184173**	-0.417976092724305	1	6	Usp7
**0.00474038628311171**	0.328790501628591	1	6	Acadl
**0.00475045317801224**	-0.558949752273749	1	6	Arsb
**0.00480312285409954**	0.736164478752694	1	6	Crat
**0.00482131338989223**	0.28	1	6	Gid4
**0.00488759122446789**	0.757921735913311	1	6	Pigyl
**0.00495764799024575**	0.342267487869564	1	6	Tnfaip8
**0.00497634021195799**	0.902558350242235	1	6	Arhgef10l
**0.00500589372497804**	0.72	1	6	Myo1e
**0.00501056830872662**	-0.39	1	6	Smc6
**0.00502219165156998**	-0.460118333749167	1	6	Fbn1
**0.00503050285980499**	-1.37	1	6	Myoc
**0.00503218914666391**	-1.39	1	6	Golm1
**0.0050411857174933**	-0.359172255211659	1	6	Nr4a1
**0.00504409114808784**	0.407330105864267	1	6	Btf3
**0.00507616181513832**	0.526424084431874	1	6	Cab39
**0.00507842188445553**	-0.42	1	6	Thoc2
**0.00508727464066337**	0.371514236183695	1	6	1110059E24R
**0.0051027499676063**	-0.420221724544573	1	6	Srsf11
**0.00512744883459789**	-0.405840707791613	1	6	Ldha
**0.00512780102839879**	0.55	1	6	Dnal1
**0.00517947779668827**	-1.10	1	6	Man1c1
**0.00519800630637612**	0.31	1	6	Cetn3
**0.00519879493295639**	-0.418698618465194	1	6	Elf2
**0.00521499362677614**	-0.553512995906706	1	6	Colec12
**0.00523812336662382**	0.554081192366384	1	6	Sap30bp
**0.00525461948887625**	0.809322222080609	1	6	Dnajc11
**0.00530716406936886**	-1.32	1	6	Metrnl
**0.00532124713254554**	-0.777974835703651	1	6	Ehd1
**0.00533144620164935**	0.466748379070849	1	6	Mif
**0.00533397297666865**	-1.15	1	6	Ppp1r15b
**0.00537643602046421**	-0.578261536197827	1	6	Cc2d2a
**0.00543265664605088**	0.722968422148796	1	6	Fbxo21
**0.00543562395112628**	-0.696092967537325	1	6	P2rx4
**0.00544319855720162**	-0.407062623424613	1	6	Sde2
**0.00547441589426576**	-1.19	1	6	Sav1
**0.00549639861893578**	0.26	1	6	Ndufa1
**0.00550054965894942**	0.382467194853659	1	6	Hsp90aa1
**0.00554740503397783**	0.684248198646839	1	6	Rps6ka4
**0.00555100892133122**	0.551632134175043	1	6	Ppp3cb
**0.00556906424090596**	0.266542236565333	1	6	Dad1
**0.00557443826703764**	0.942869158712843	1	6	Etv1
**0.00558300748708494**	-0.643078939899759	1	6	Azin1
**0.00559151822006167**	-1.29	1	6	Esam

**0.00560091262613108**	-0.704405511556489	1	6	Rpl27a
**0.00568437000902467**	-2.00	1	6	Cdh5
**0.00569439484787757**	-1.57	1	6	Ism1
**0.00569866363075961**	-1.07	1	6	Nrip1
**0.00571452097364504**	0.456021431227559	1	6	Ndufs7
**0.00574092655896024**	-0.599566635092476	1	6	Pak1ip1
**0.00577062778349256**	-0.363214529879237	1	6	Spag9
**0.00577572449951731**	-0.465932351223908	1	6	Camk2n1
**0.00578801640359427**	0.45	1	6	Ppt1
**0.00583924970854206**	-0.758602502128453	1	6	Cyp27a1
**0.00584025182576864**	-0.510710964590265	1	6	Gypc
**0.0058452024605872**	-0.625384381633862	1	6	Cpd
**0.00586568422147368**	-0.738074075028632	1	6	Rassf1
**0.00587934526836622**	0.378674118740662	1	6	Cct2
**0.00595411028235072**	-1.22	1	6	Gnptg
**0.00596226704281594**	-0.62	1	6	Socs2
**0.00597402617854992**	-0.303892335105075	1	6	Lztr1
**0.00598416545102669**	-0.707722872471542	1	6	Srek1
**0.0060180385301073**	0.456960830216764	1	6	Ptbp2
**0.00607048240364613**	-0.717510929895972	1	6	NP-904339.1
**0.00607493563780491**	0.469582522591195	1	6	Tmco1
**0.00611853648081958**	-0.381937952116143	1	6	Col3a1
**0.00611911144935103**	0.672920179580641	1	6	Mpv17l2
**0.00613580249590627**	0.438945461127691	1	6	Hebp1
**0.00617448541801295**	0.52	1	6	Gcsh
**0.00618021136383649**	0.330072214775745	1	6	1810013L24F
**0.0061881699480389**	-1.42	1	6	Rnf144a
**0.0062275136328221**	0.265847462880904	1	6	Tma7
**0.00625263086979743**	0.840808715953557	1	6	Pcbd2
**0.00625497709532199**	-0.481748026046787	1	6	Nktr
**0.00626746048565906**	0.647010411378387	1	6	Sncg
**0.00631226260484344**	-1.30	1	6	Bach1
**0.00631779064305656**	-0.584662630126925	1	6	Hdac7
**0.00633338531763113**	0.495974288182644	1	6	Atad1
**0.00634659133652592**	0.605473032114992	1	6	Aldh6a1
**0.00641161969021748**	-0.531177451731583	1	6	Carhsp1
**0.00642114479989468**	-0.930791822564955	1	6	Rap2b
**0.00645350212050368**	-0.486115694436128	1	6	Tceal8
**0.00646474795418536**	0.252834923710015	1	6	Hypk
**0.0065208210126438**	-0.411794981589814	1	6	Arpc1b
**0.00655751496583505**	-0.28	1	6	Nfix
**0.00658509818058639**	-0.883953320610404	1	6	Ncstn
**0.00664248848270722**	-0.643496821919826	1	6	Ptp4a1
**0.00665064270377943**	0.310472681275748	1	6	Serhl
**0.0066524012360032**	0.345501643340607	1	6	Tyw1
**0.00676979807760387**	-0.999702047374669	1	6	4931406P16F
**0.00677301437363731**	0.505541184330336	1	6	Lamtor2
**0.00682620822162269**	0.300197384382102	1	6	Arf1
**0.0068311506522193**	0.653185909485878	1	6	Snhg3
**0.00690249259932349**	0.464778465573392	1	6	Cabin1
**0.00694832623802859**	-0.667037923840119	1	6	Tgfb3
**0.00698114258616238**	-0.766149707763836	1	6	Cirh1a
**0.00698233122272312**	-0.757580925458406	1	6	Trib2
**0.00702165490029568**	0.48	1	6	Cars2
**0.00706923764096157**	-0.602753442856319	1	6	Por
**0.00710802417531784**	0.539053660555265	1	6	Csnk2a1

**0.0071176745638443**	0.304445134743103	1	6	Cnbp
**0.00713544380486288**	-0.757906817590996	1	6	Smad1
**0.00713816839182996**	-0.489892815004547	1	6	Ifrd1
**0.00715353390685013**	-0.62	1	6	Hbp1
**0.00716597084673883**	1.16	1	6	Plcl1
**0.00718369582939398**	-0.384049623874866	1	6	Vwa1
**0.00720170387855392**	-0.637386803672809	1	6	Hspa13
**0.00725010065014327**	0.464032226945758	1	6	Bcl2l2
**0.00726808954626692**	-0.943797847947397	1	6	Lonrf3
**0.00728618689980936**	0.407363889077392	1	6	Sntg2
**0.00734262607407251**	-0.664948786590032	1	6	Plk3
**0.00737183398533685**	-1.80	1	6	Fgd5
**0.00737553489529951**	-0.999170678656911	1	6	Steap3
**0.00739812632625175**	-0.420044557871321	1	6	Slc44a1
**0.00740172122599652**	0.253903723710823	1	6	Hnrnpk
**0.00741588136898538**	0.297903843641722	1	6	Prss23
**0.00743656771919348**	-0.446927730289008	1	6	Amotl1
**0.00744592839862284**	0.602271923079149	1	6	Mras
**0.0075021881286717**	0.337645452336456	1	6	Hbegf
**0.00753137624908947**	-0.312368177855258	1	6	Ubn2
**0.00757835577728313**	-0.899876075953451	1	6	Elmod2
**0.00759760724937743**	-1.40	1	6	Chl1
**0.00761943785749936**	-0.652573569162379	1	6	Zkscan17
**0.00767305059854885**	0.435034394068396	1	6	Ncoa3
**0.00774440691993106**	-0.575951279023118	1	6	Gpsm1
**0.00775550003811308**	0.36	1	6	Chd3
**0.00777596931977976**	0.342318827842878	1	6	Tnks
**0.00784992172363976**	-0.654825126539826	1	6	Creb3l1
**0.007894399639379**	-0.649477149760606	1	6	Naga
**0.00795461569827644**	-1.05	1	6	Phf11d
**0.00802403604766408**	0.46	1	6	Cacybp
**0.00808235377957593**	0.388514875367544	1	6	Rps27a
**0.00808276236399856**	0.447446953115006	1	6	Chchd3
**0.00809917537347324**	0.354191293721472	1	6	Rhoc
**0.00810123707099042**	-1.70	1	6	Car8
**0.008107319347262**	-0.30	1	6	Atp9a
**0.00813378104232408**	-0.895871669368692	1	6	Cdr2l
**0.00813570032778875**	-0.814305779376278	1	6	Slc20a1
**0.00814193960807918**	-0.495966293470997	1	6	Setbp1
**0.00814328640629092**	-0.373398849125836	1	6	Atrx
**0.00814730931075331**	0.480501367003817	1	6	Gfra4
**0.00816697984073502**	0.584473728605578	1	6	Dars
**0.00826876126543035**	0.99	1	6	Tmem261
**0.00828270769766177**	-0.631920451349505	1	6	Pafah1b2
**0.00831212671508312**	0.784504321795256	1	6	Mob2
**0.00831261429334249**	-0.516333309347422	1	6	Atp11a
**0.00837214422101295**	-0.512403283241463	1	6	Arf5
**0.00839200061666761**	0.397342195515716	1	6	Rasl11b
**0.00840413059469083**	-0.878733746749619	1	6	Pim1
**0.0084868783780202**	-1.07	1	6	Pcsk6
**0.0085735542886414**	0.51	1	6	Prpsap1
**0.00863520740033964**	0.347899841046203	1	6	Snap23
**0.008643813804221**	-0.683452258154592	1	6	Sdpr
**0.00865652262482112**	-1.01	1	6	Mapk8
**0.00869560171863148**	-0.86	1	6	Parp9
**0.00873199160200797**	0.620567050340954	1	6	Dnm1l

**0.00874913571094588**	0.311377805225706	1	6	Map7d1
**0.00878181643688353**	-0.493980536331055	1	6	Pdcd11
**0.0088913130227541**	-0.652724797896126	1	6	Ccnl2
**0.00892589701768925**	-1.47	1	6	Mysm1
**0.00893414101297008**	-1.33	1	6	Tlr2
**0.00895456904984314**	0.35	1	6	Emc2
**0.00897187496025955**	-0.661180513633317	1	6	Ugdh
**0.00897833571917455**	0.298673793318183	1	6	Prkra
**0.00900320338560473**	-0.721438916297196	1	6	9430020K01[illegible]
**0.00900890901269725**	-0.465716245145855	1	6	Gna13
**0.00903707284355915**	-1.07	1	6	Msx1
**0.00904761581867677**	0.468221250741601	1	6	Idh3a
**0.00907042477975321**	-0.616285361117262	1	6	Ppic
**0.00913416723224837**	-0.787690185264355	1	6	Gtf3c2
**0.00913783762017805**	0.344078155555753	1	6	Fam149b
**0.0092245208890353**	-0.455040716238961	1	6	Phip
**0.00925411497216753**	0.606424769875645	1	6	Med22
**0.00926402833867204**	-1.38	1	6	Angpt1
**0.00926848555584202**	-0.57	1	6	Sidt2
**0.00927909469342221**	-1.11	1	6	Pgap2
**0.00928387479060062**	0.509856373105657	1	6	Dhrs1
**0.00933914337957568**	-0.555970281631253	1	6	Ddx58
**0.00937955393799242**	0.374098808977514	1	6	Aqp1
**0.00945022072056592**	0.495638199153719	1	6	Mrpl4
**0.00946592333201767**	0.370571109130474	1	6	Capza2
**0.00949483784429132**	0.316821526066903	1	6	Ccdc82
**0.00949772446379001**	0.493620833741536	1	6	Tmem246
**0.00951953674654007**	0.558240613163689	1	6	Pigp
**0.00955335899383469**	0.584513005327859	1	6	N6amt1
**0.00956641033408595**	-0.452091906044224	1	6	Soga1
**0.00957057399361193**	-0.285264792156097	1	6	Usp16
**0.00962660987945945**	-0.566085256636712	1	6	Psmd3
**0.00964832869756862**	0.405339944751414	1	6	Usp53
**0.00973056659777927**	0.378866022602754	1	6	Mcm6
**0.00975562913326475**	-0.611260174771025	1	6	Stag1
**0.00979272572140206**	-0.515753201087958	1	6	Abhd11os
**0.00981801575238949**	0.410807051913878	1	6	Ckb
**0.00993222278074916**	0.389605834155689	1	6	Dgcr6
**0.00995063354641645**	0.395316181676926	1	6	Synj2bp
**0.00995805688930114**	0.62	1	6	Mzt1
**0.00996651118772677**	-0.778190329724526	1	6	Lrrc8a
